# A Single Molecule Scaffold for the Maize Genome

**DOI:** 10.1371/journal.pgen.1000711

**Published:** 2009-11-20

**Authors:** Shiguo Zhou, Fusheng Wei, John Nguyen, Mike Bechner, Konstantinos Potamousis, Steve Goldstein, Louise Pape, Michael R. Mehan, Chris Churas, Shiran Pasternak, Dan K. Forrest, Roger Wise, Doreen Ware, Rod A. Wing, Michael S. Waterman, Miron Livny, David C. Schwartz

**Affiliations:** 1 Laboratory for Molecular and Computational Genomics, Department of Chemistry, Laboratory of Genetics, UW Biotechnology Center, University of Wisconsin–Madison, Madison, Wisconsin, United States of America; 2 Department of Plant Sciences, Arizona Genomics Institute, University of Arizona, Tucson, Arizona, United States of America; 3 Departments of Mathematics, Biology, and Computer Science, University of Southern California, Los Angeles, California, United States of America; 4 Cold Spring Harbor Laboratory, Cold Spring Harbor, New York, United States of America; 5 Corn Insects and Crop Genetics Research, United States Department of Agriculture–Agricultural Research Service and Department of Plant Pathology, Iowa State University, Ames, Iowa, United States of America; 6 Plant, Soil, and Nutrition Research, United States Department of Agriculture–Agricultural Research Service, Ithaca, New York, United States of America; 7 Computer Sciences Department, University of Wisconsin-Madison, Madison, Wisconsin, United States of America; The Salk Institute for Biological Studies, United States of America

## Abstract

About 85% of the maize genome consists of highly repetitive sequences that are interspersed by low-copy, gene-coding sequences. The maize community has dealt with this genomic complexity by the construction of an integrated genetic and physical map (iMap), but this resource alone was not sufficient for ensuring the quality of the current sequence build. For this purpose, we constructed a genome-wide, high-resolution optical map of the maize inbred line B73 genome containing >91,000 restriction sites (averaging 1 site/∼23 kb) accrued from mapping genomic DNA molecules. Our optical map comprises 66 contigs, averaging 31.88 Mb in size and spanning 91.5% (2,103.93 Mb/∼2,300 Mb) of the maize genome. A new algorithm was created that considered both optical map and unfinished BAC sequence data for placing 60/66 (2,032.42 Mb) optical map contigs onto the maize iMap. The alignment of optical maps against numerous data sources yielded comprehensive results that proved revealing and productive. For example, gaps were uncovered and characterized within the iMap, the FPC (fingerprinted contigs) map, and the chromosome-wide pseudomolecules. Such alignments also suggested amended placements of FPC contigs on the maize genetic map and proactively guided the assembly of chromosome-wide pseudomolecules, especially within complex genomic regions. Lastly, we think that the full integration of B73 optical maps with the maize iMap would greatly facilitate maize sequence finishing efforts that would make it a valuable reference for comparative studies among cereals, or other maize inbred lines and cultivars.

## Author Summary

The maize genome contains abundant repeats interspersed by low-copy, gene-coding sequences that make it a challenge to sequence; consequently, current BAC sequence assemblies average 11 contigs per clone. The iMap deals with such complexity by the judicious integration of IBM genetic and B73 physical maps, but the B73 genome structure could differ from the IBM population because of genetic recombination and subsequent rearrangements. Accordingly, we report a genome-wide, high-resolution optical map of maize B73 genome that was constructed from the direct analysis of genomic DNA molecules without using genetic markers. The integration of optical and iMap resources with comparisons to FPC maps enabled a uniquely comprehensive and scalable assessment of a given BAC's sequence assembly, its placement within a FPC contig, and the location of this FPC contig within a chromosome-wide pseudomolecule. As such, the overall utility of the maize optical map for the validation of sequence assemblies has been significant and demonstrates the inherent advantages of single molecule platforms. Construction of the maize optical map represents the first physical map of a eukaryotic genome larger than 400 Mb that was created *de novo* from individual genomic DNA molecules.

## Introduction

Maize (*Zea mays* ssp. *mays* L.) is a pervasive, economically valuable crop supplying the world with food, animal feed, and with biofeedstocks used in the synthesis of a broad range of industrial products. It is also a model system for classical genetics and cytogenetics that has significantly contributed to our understanding of fundamental processes that include reproduction, photosynthesis, biosynthesis of primary metabolites, mobile elements, and chromosome structure-function relationships. Investigators have developed extensive genetic tools over the last two decades dealing with male sterility, QTLs, regeneration of crop species, wide hybridization, marker assisted selection, associative mapping, endosperm mutants, transgenic crops, genetic control of meiosis, transposable elements, chromosome elimination, *etc*. In addition, diverse germplasms have been accumulated that have leveraged the assessments of genomic modifications during domestication, molecular mechanisms of heterosis, and the roles played by mobile DNA elements affecting genome evolution. Such advances are now being rapidly exploited with paradigm shifting tools and resources that are fostering insights emerging from fully sequenced and annotated genomes. In 2005 three funding agencies – NSF, DOE and USDA – jointly pledged $32 million towards a 4-year program to sequence the maize genome. These agencies' goals were to ensure that cutting-edge genomic resources would be available for maize to accelerate translational research in the agriculture and bioenergy sectors.

The maize genome is estimated to be 2.3–2.5 gigabases (Gb) in size [1], and its architecture presents significant challenges for comprehensive sequencing. An intriguing attribute of the maize genome is its allotetraploidy nature that originated at least 5 million years ago (mya) from two progenitors, which had previously diverged from a common ancestor about 12 mya [2]–[3]. The maize genome underwent a whole genome duplication event in the hybridization of the two progenitors, and then gradually became diploid through loss of ∼50% of one of its progenitors' gene copies [4]–[8].

The architecture of the maize genome is also heavily punctuated by a complex motif of repetitive elements. About 85% of the genome is made up of a complex mix of repetitive DNA that mainly includes numerous families of retrotransposons such as *Tekay*, *Huck*, PREM-2, *Opie*, *Ji*, etc. [9]–[11]. These retroelements mostly appeared during the last 1 to 3 million years and thus show great similarity [9]. The chromosome “knobs” consist of megabase-sized satellite sequences interspersed with retrotransposons, while the euchromatic regions harbor repetitive insertions of transposons, with most retrotransposons tending to insert within each other, resulting in nested retrotransposons in the intergenic regions [9], [11]–[17]. Therefore, maize genes are like small islands surrounded by seas of nested retrotransposons, and such challenging attributes have necessitated development of multiple sequencing approaches.

Given the current need for a broadly informative representation that includes coding sequences and precise physical characterization of gaps between genes, accurate genetic and physical maps are required for guiding the large-scale sequencing of maize genome. The genetic, physical, and integrated maps available for maize are briefly described. The 1935 maize genetic map featured just 62 loci that relied on morphological variants [18]. Advancements in new technologies and genomic insights led to the addition of nearly 6,000 markers to create a high resolution genetic map using the intermated B73 X Mo17 (IBM) populations [19]–[20] (http://maize-mapping.plantgenomics.iastate.edu/). This augmentation drew new resources from the development of cytological markers based on B-A translocations, molecular markers based on isozymes, restriction fragment length polymorphisms (RFLPs), microsatellite or simple sequence repeat (SSR) markers, single nucleotide polymorphisms (SNPs), and cDNA or expressed sequence tags (EST) markers [19], [21]–[38]. In addition, FPC (fingerprinted contigs) [39] map contigs were also anchored to this genetic linkage map, and this highly integrated resource became known as the “iMap.”

Early physical mapping of maize used a YAC (yeast artificial chromosome) library constructed from an inbred line UE95 [34]. The YAC libraries proved to be of limited utility due to a significant level of clone chimerism, or issues surrounding YAC stability and faithful representation of genome copy number [40]. With the advent of stable large-insert cloning in bacteria, more reliable BAC (bacterial artificial chromosome) [41] libraries were constructed for the maize B73 inbred line. Clones were fingerprinted, hybridized with known molecular markers (genetic), and FPC mapped. These efforts integrated the genetic and physical maps through assignment of molecular markers from the genetic map to individual BAC clones within FPC contigs [35], [42]–[46]. FPC maps were later greatly refined by HICF (fluorescent-based high-information-content fingerprinting) mapping of these libraries which reduced the number of FPC contigs from 4,518 to 1,500 [47]. The number of FPC contigs was further reduced to 721 (2,150 Mb; May, 2006) by manual curation based on agarose-based fingerprinting and on knowledge gleaned from the HICF map and syntenic markers between the maize and rice genomes [37]. In addition to the ∼6,000 genetic markers, there are over 24,000 sequence markers integrated into the maize genetic-physical (FPC) map (also termed iMap), including expressed sequence tag (EST)-derived unigene markers, overgos derived from maize EST sequences, conserved genomic sequences, and end-sequence data from gene-containing BACs. The inclusion of these sequence markers into the integrated map (iMap) (IBM2; iMap; http://www.maizemap.org/iMapDB/iMap.html) has greatly increased the marker density across the entire maize genome and created a framework for directed clone-based sequencing and assembly of chromosome-wide pseudomolecules [37],[44],[48]. However, the maize genome is structurally highly polymorphic, as seen in the significant structural variation among different inbred lines and even between different haplotypes [49]–[52]. Because the maize iMap integrates the IBM genetic map with the B73 inbred line FPC physical map, structural differences between the IBM population and the B73 genome (targeted genome for sequencing http://ftp.maizesequence.org/release-3b.50/All Releases/ [53]) would be expected. The primary construction of high-resolution physical maps that are not dependent on the IBM genetic map would offer an essential resource for the comprehensive and accurate assembly of the maize B73 reference genome.

Sequencing efforts for the maize genome have progressed through three stages: the pilot, gene enrichment, and clone-by-clone full genome sequencing stages. **1)** The pilot sequencing effort considered large parts of chromosome arms and BAC-end sequence gathered from random clones; this provided an early glimpse into genome structure, organization, and sequence composition [48], [54]–[55]. **2)** The gene-enrichment approaches culled gene-rich templates for side-stepping notoriously complex sequence repeats and high copy number DNA elements present in the maize genome. Enrichment was accomplished by a variety of sequencing approaches that included ESTs, genome filtration (methylation filtration and high-Cot selection), RescueMu (RM), and hypomethylated partial restriction (HMPR) [56]–[63]. Sequence data enriched for genes, collectively termed as Genome Survey Sequences (GSSs), are scattered throughout the maize genome, typically comprising small sequence contigs a few kilobases in size [61]. **3)** In contrast, clone-by-clone sequencing used a comprehensive, hierarchical, map-based approach that allowed construction of a BAC minimal tiling path across the iMap. Tiled BACs were then individually shotgun-sequenced and assembled [12], [64]–[66]. Although this map-based approach simplified assembly, an individual BAC assembly typically contained multiple unordered sequence contigs. Sequencing of the maize genome is now in the finishing phase with more than 16,000 sequenced BACs [53]. But complete sequencing and creation of a highly accurate assembly of the maize genome still hold daunting challenges for the maize community.

A direct and encompassing way to deal with the formidable architecture of the maize genome is to analyze “chunks” of it, at high-resolution, that are as large as possible. In this way, nests of sequence repeats are largely bridged by chunks that offer a sufficient level of unique sequence information for supporting *de novo* genome assembly. With this concept in mind, we constructed a high-resolution optical map [67]–[76] that spans ∼91% of the maize genome by the *de novo* assembly of a large data set containing ordered restriction maps of individual genomic DNA molecules ∼500 kb in size. This ordered restriction map provides an independent resource that lays out an accurate physical metric across the entire maize genome. Because large molecules were analyzed, we were able to physically map repeat-rich regions and link sequence and map data within complex genomic regions. We show here that our maize optical map identifies gaps within and between sequence contigs and guides the assembly and validation of reference chromosomes.

## Results

### Data acquisition and map assembly

We constructed a whole-genome shotgun optical map for maize using the CpG methylation insensitive restriction enzyme SwaI. The optical map data set contains 2,116,074 genomic DNA molecules, ranging in size from 300 kb to 3,700 kb, and totaling ∼927,604 Mb, or ∼403× coverage of the maize genome. The maps in this raw data set—one optical restriction map per genomic DNA molecule—have a mean length of 438.4 kb with an average fragment size of 26.1 kb.

Because of the vast size of the maize optical data set, our *de novo* assembly of maps relied on a divide and conquer strategy that leveraged available cluster computing resources [77]. Briefly, we divided the raw map data set into 40 separate bins. Each bin was assembled into contigs and processed to remove redundant contigs and/or overlapping contigs, producing seed maps (consensus maps) for our iterative assembly scheme (Materials and Methods). After five initial cycles of iterative assembly, the terminal 40 restriction fragments of a seed map (Materials and Methods) were selected for augmentation of optical contigs that were >10 Mb. These optical consensus maps were lengthened and their depth of coverage was increased through an additional 15 cycles of iterative assembly using the entire map data set. In this way, we constructed 66 optical consensus maps spanning a total of 2,103.93 Mb.

The consensus maps were internally validated in an additional iterative assembly step. They were partitioned into a series of overlapping 1 Mb map intervals for use as new seed maps, with the overlaps covering ∼500 kb. Because this diagnostic assembly reproduced the original set of 66 parental contigs, the current optical assembly is apparently free of any chimeric maps. Statistics describing the 66 optical map contigs are shown in Table 1. In total, 339,280 of the 2,116,074 maps were assembled into 66 optical map contigs. The average depth of coverage is 72 restriction fragments per contig (Table 1). The breadth of these contigs range from 3.64 Mb–100.76 Mb, and the average contig size is 31.88 Mb. The average size of restriction fragments of each contig ranges from 21.32 kb to 28.53 kb, with the overall size averaging 23.56 kb (Table 1). Lastly, the rate of contig formation was 16.03%, and we attribute this modest value to the modest rate of restriction digestion caused by unknown inhibitors within our DNA preps (genomic; ∼500 kb sized molecules) that attenuated restriction enzyme action. We leveraged the assembly process for overcoming this problem by increasing the number of digested molecules within the raw data set for biasing those molecules with adequate restriction patterns supporting confident contig formation.

**Table 1:** Statistics of optical map contigs and the anchoring of FPC contigs.

Optical Map Contig Name	Contig Size (Mb)	Ave Frag Size (kb)	# of Sin Mol Maps	Coverage (X)	Ave. SD	Chr. Anchored	FPC Contigs Spanned
**OMcontig_0**	100.76	22.82	15532	71.75	2.37	1	ctg30–48
**OMcontig_1**	97.04	22.17	15903	76.15	2.34	4	ctg176–196
**OMcontig_2**	94.49	23.30	15048	75.14	2.38	2	ctg68–84
**OMcontig_3**	85.78	22.45	14382	77.58	2.37	1	ctg12–30
**OMcontig_4**	84.69	22.83	13331	73.39	2.34	8	ctg332–354
**OMcontig_5**	83.37	22.42	13010	72.46	2.32	2	ctg87–98
**OMcontig_6**	70.90	22.85	12456	81.35	2.40	6	ctg279–291
**OMcontig_7**	68.92	22.55	12094	80.65	2.39	5	ctg204–222
**OMcontig_8**	65.88	22.28	11397	80.45	2.33	10	ctg405–420
**OMcontig_9**	58.50	23.34	8773	70.35	2.42	4	ctg169–176
**OMcontig_10**	57.33	24.52	9081	73.21	2.36	7	ctg301–315
**OMcontig_11**	56.78	23.74	9156	75.18	2.43	3	ctg132–149
**OMcontig_12**	55.30	23.88	10278	86.38	2.44	3	ctg121–131
**OMcontig_13**	55.02	22.85	9120	77.25	2.39	3	ctg111–118
**OMcontig_14**	52.11	23.46	8026	71.65	2.43	5	ctg232–238
**OMcontig_15**	50.50	22.27	8426	78.10	2.35	1	ctg1–12
**OMcontig_16**	49.65	23.32	8204	77.72	2.43	8	ctg326–334
**OMcontig_17**	49.87	23.82	7795	73.13	2.49	4	ctg196–197/ctg376–381
**OMcontig_18**	46.99	23.05	7207	71.59	2.35	9	ctg371–376
**OMcontig_19**	47.25	22.50	8293	81.33	2.36	1	ctg51–64
**OMcontig_20**	43.23	23.17	6762	72.89	2.43	7	ctg292–300
**OMcontig_21**	40.29	22.25	7508	86.06	2.36	10	ctg392–398
**OMcontig_22**	38.60	23.87	5760	69.53	2.44	5	ctg223–231
**OMcontig_23**	38.23	23.18	6946	84.95	2.39	9	ctg383–391
**OMcontig_24**	33.63	22.48	5434	75.07	2.38	4	ctg160–164
**OMcontig_25**	29.78	22.80	4650	72.85	2.32	5	ctg238–247
**OMcontig_26**	27.71	23.76	4893	82.23	2.39	2	ctg99–104
**OMcontig_27**	26.19	22.83	4524	80.34	2.33	7	ctg317–321
**OMcontig_28**	25.16	23.70	3491	64.90	2.42	3	ctg121/255
**OMcontig_29**	24.54	23.53	3940	74.08	2.39	4	ctg170–171
**OMcontig_30**	24.16	21.59	4135	78.77	2.19	6	ctg267–269
**OMcontig_31**	23.93	21.69	4254	82.07	2.39	7	ctg322–325
**OMcontig_32**	23.15	22.88	4041	81.14	2.35	8	ctg354–358
**OMcontig_33**	22.99	22.08	3921	79.63	2.33	5	ctg248–254
**OMcontig_34**	21.07	23.46	3421	75.37	2.36		
**OMcontig_35**	20.54	23.89	3269	75.73	2.54	8	ctg359–366
**OMcontig_36**	20.51	22.48	3457	78.10	2.27	6	ctg265–269
**OMcontig_37**	20.32	23.52	2882	66.75	2.40	10	ctg399–401
**OMcontig_38**	16.98	22.37	2766	76.90	2.37	4	ctg199–203
**OMcontig_39**	16.13	21.85	3174	90.19	2.34	2	ctg108–110
**OMcontig_40**	15.61	24.98	2272	67.09	2.40	unknown	ctg430
**OMcontig_41**	14.53	24.17	1831	58.63	2.48	unknown	ctg449
**OMcontig_42**	14.31	24.02	1981	64.32	2.48	5	ctg231–232
**OMcontig_43**	13.48	23.48	1775	61.34	2.30	7	ctg300–301
**OMcontig_44**	13.34	22.01	1989	70.50	2.36	4	ctg156–159
**OMcontig_45**	13.45	22.91	2001	70.16	2.35	10	ctg401–404
**OMcontig_46**	13.29	22.99	2069	72.28	2.38	3	ctg118–120
**OMcontig_47**	12.73	21.32	2088	75.57	2.22	3	ctg150
**OMcontig_48**	11.86	27.91	1448	58.58	2.60	6	ctg256–260
**OMcontig_49**	11.49	25.64	1478	60.39	2.51	2	ctg84
**OMcontig_50**	10.34	25.22	1266	57.16	2.59	2	ctg120
**OMcontig_51**	10.33	23.05	1475	65.42	2.38	9	ctg368–370
**OMcontig_53**	8.70	24.51	1255	66.65	2.43	6	ctg271–274
**OMcontig_55**	8.82	25.28	1043	55.96	2.48	unknown	ctg448
**OMcontig_56**	7.89	26.03	965	58.37	2.43	1	ctg49
**OMcontig_57**	7.84	23.19	1103	65.17	2.39		
**OMcontig_58**	6.17	26.02	683	51.11	2.51	2	ctg106–108
**OMcontig_59**	5.68	22.81	939	77.11	2.40	1	ctg50
**OMcontig_60**	5.72	21.60	885	70.21	2.37	6	ctg267
**OMcontig_61**	5.69	23.81	911	76.34	2.45	1	ctg65–67
**OMcontig_64**	4.52	27.08	491	51.02	2.72	2	ctg106
**OMcontig_65**	4.16	24.47	521	58.40	2.45	6	ctg264
**OMcontig_66**	4.14	28.53	593	66.00	2.72	2	ctg105
**OMcontig_67**	3.89	22.90	565	64.92	2.30	6	ctg263
**OMcontig_68**	4.04	27.11	487	56.27	2.59	7	ctg321–322
**OMcontig_69**	3.64	26.37	426	54.63	2.62		
**Total/** ***Ave*** **.**	**2103.93/** ***31.88***	***23.56***	**339280**	***71.61***	***2.41***		

*Note: Ave Frag Size = average fragment size, Sin Mol Map = single molecule map, Ave. SD = average standard deviation, Chr. = chromosome.

**Figure 1:**
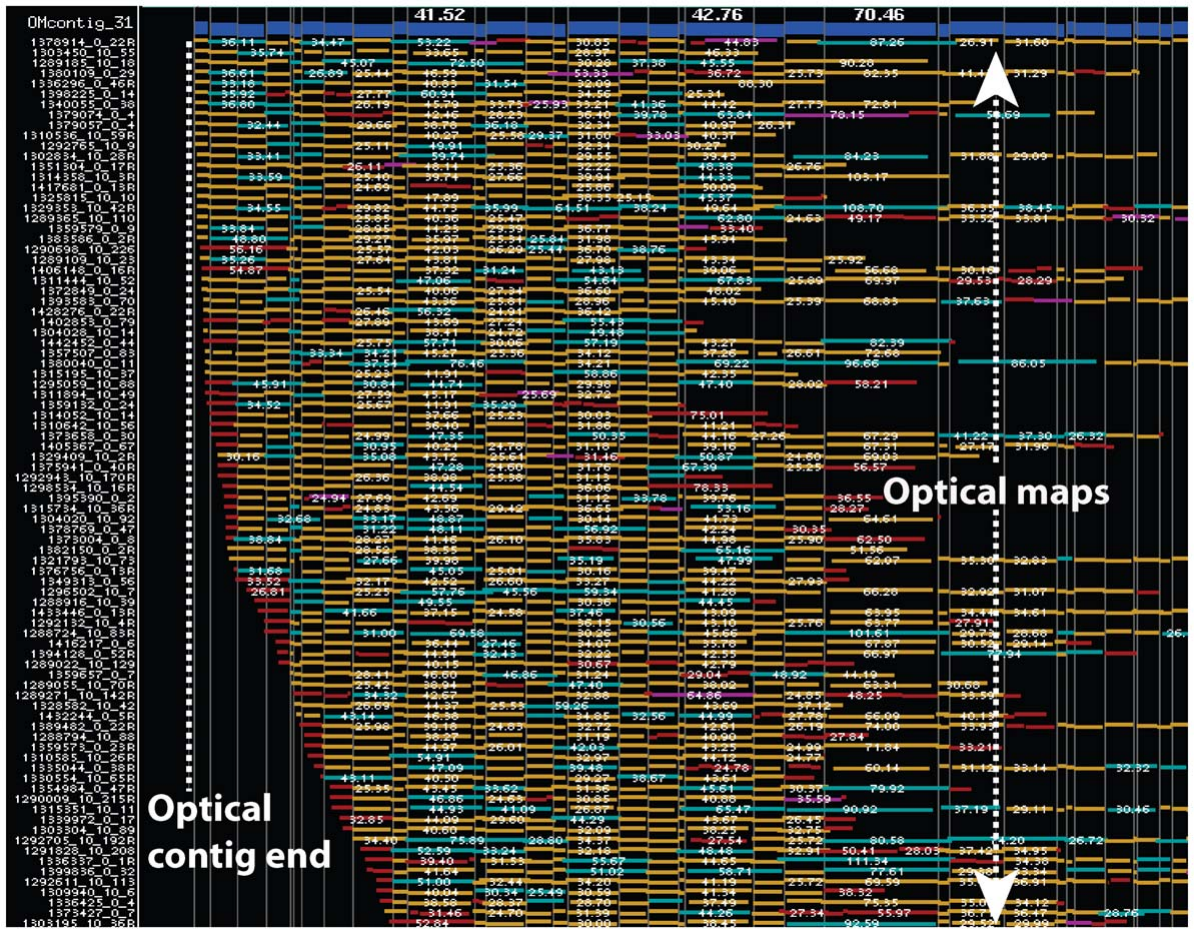
A screenshot of an optical contig (OMcontig_31) showing a possible telomeric-end. The Genspect viewer depicts each optical map, constructed from a genomic DNA molecule, as a horizontal track consisting of colored boxes. The length of each box represents the size (in kb) of a restriction fragment within a genomic molecule. The map information of the entire contig is combined into a single track (top; blue) called the consensus map. Restriction fragments are colored keyed for indicating their agreement with the consensus map; gold boxes show agreement, while red (false cut), blue (missing cut), and purple (false cut) indicate aforementioned restriction map differences. This deep contig shows a distinct edge, or end, populated by ∼40 optical maps indicating a telomeric region (Chr7).

Optical contigs terminate to form a gap when the SwaI restriction site density is low, or when a contig reaches the end of a chromosome. Sharply demarcated contig edges may represent telomere associated sequences near chromosome ends. Using these criteria we identified 15 contigs (OMcontigs_7, 8, 13, 16, 20, 21, 23, 28, 31, 35, 38, 39, 47, 51, and 61) that have reached the ends of chromosome as evidenced by contig “edges” comprising more than 5 maps that show no significant map “overhangs” (Figure 1). A collection of DNA molecules (maps) is said to overhang at a contig's end when their terminal restriction fragments are large and vary in length—such patterns describe gaps.

### Development of a new algorithm, BACop, for integration of optical map contigs with iMap

**Figure 2:**
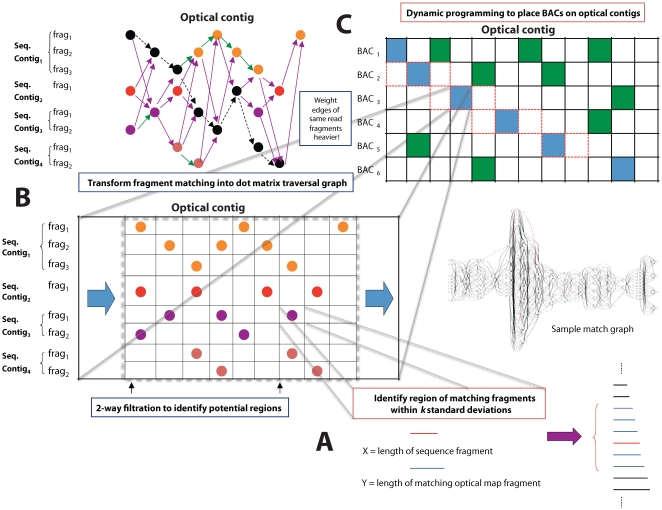
BACop, an algorithm that anchors optical contigs onto the iMap. BACop (Materials and Methods) employs four distinct steps for anchoring optical contigs; we illustrate the first three steps here: (A) Restriction fragments of an optical contig map are matched against BAC sequences comprising multiple sub-contigs. (B) Matching BAC sequence contigs are located along the optical contig map. (C) Dynamic programming places BACs onto optical map contigs. Seq. = sequence, frag = restriction fragment, and BAC = bacterial artificial chromosome.

The maize genome optical map contains 66 optical contigs and 91,453 *ordered* SwaI restriction fragments. However, placement of tiled (16,848 FPC clones), but unfinished, BAC sequences released by the maize genome sequencing project (release 3b.50; http://ftp.maizesequence.org/release-3b.50/All%20Releases/; March 19, 2009) on optical maps required development of a new algorithm that considers alignments of FPC clones comprising unordered and unoriented sequence contigs (averaging 11 sequence contigs per BAC). We had developed a new algorithm several years ago to integrate the optical and FPC maps through the alignment of unfinished BAC sequence data (Materials and Methods, Figure 2). Our motivation for its development was to anchor large optical contigs to the iMap, which in 2007 contained only ∼6,000 sequenced BACs. The algorithm—named “BACop” —considers “complete” SwaI restriction fragments (fragments having pairs of SwaI sites) present in the *in silico* digest of BAC sequence data; the BACs that are analyzed are restricted to those placed on the FPC map. When several consecutive restriction fragments are present, BACop places a set of contigs, belonging to a BAC, onto the optical contig using boundaries consistent with the upper size range (250 kb) of such clones. This alignment also considers the fragment sizing error model used for alignment of optical and sequence *in silico* maps [78]. The final placement of optical map contigs onto the maize iMap relies on global considerations of BAC locations on the optical *vs.* FPC maps (Figure 2). For example, when both maps have placed BACs showing similar ordering and spacing (with 20% error allowed), alignments are said to be “co-linear.” Overlaps or gaps are represented on the FPC framework when discordant optical and FPC distances range from ∼200 kb to 2 Mb. When an optical contig aligns to multiple locations on the iMap, the alignment having the greatest number of BACs is selected.

**Figure 3:**
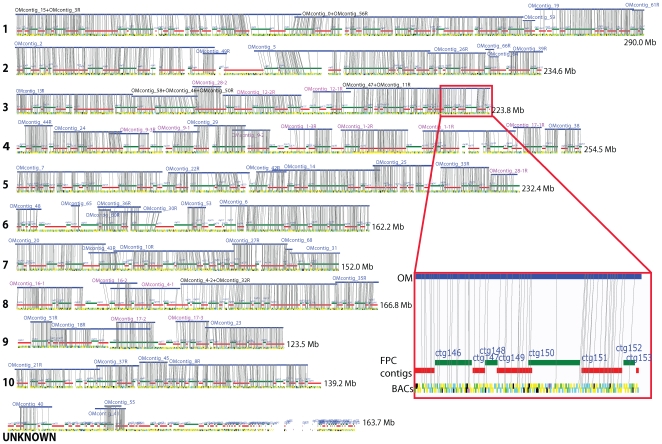
Optical contig placements on iMap using BACop. iMap chromosomes are numbered tracks showing the locations of FPC contigs and their subsidiary BACs. BACs (small boxes) are colored keyed according to their sequencing status: magenta [HTGS_FULLTOP - 2×384 paired-end attempts (6× coverage), completed shotgun phase, initial assembly]; lime [HTGS_PREFIN - completed automated improvement phase (AutoFinish)]; cyan [HTGS_ACTIVEFIN - active work being done by a finisher], yellow [HTGS_IMPROVED - finished sequence in gene regions; improved regions will be indicated, once order and orientation of improved segments are confirmed; a comment will be added to indicate this], and black [BACs with no usable or complete SwaI fragments]. The inset shows a zoomed view of a region (ctg146–153) on Chr 3. The blue tracks show optical map contigs anchored to the iMap by BACop. Optical contig identifiers are lettered in blue; pink lettering indicates that an OMcontig was split into two or more pieces for optimizing alignments. Black lettering and a “+,” indicate that two or more optical map contigs were joined. Vertical grey lines show placements of BACs onto optical map contigs. OM = optical map.

BACop placed 91% of the optical contigs (60/66) onto the 2006 FPC map [37], with 3 additional optical contigs placed onto FPC contigs that lack chromosome assignments (Table 1; Figure 3). The total breadth (2,032.42 Mb) of 60 optical contigs placed on this FPC map (1,981 Mb) is slightly larger than its total size. This extra mass accrues from optical contigs that bridge across FPC gaps, and pairs of optical contigs that partially span gaps. At these locations FPC gaps (reported, or optically revealed) are apparent because one of the overlapping optical contigs in such pairs has very few if any placed BACs, indicating the presence of a large gap between adjoining FPC contigs, or their incorrect placement. For example, optical contigs OMcontigs_28 and 50 were originally incorrectly placed onto Chr 3 FPC contigs ctg120 and ctg121. Although these optical contigs overlapped, only OMcontig_28 showed a dense pattern of BACs that aligned to FPC contig ctg121, but none to the adjoining FPC ctg120 within the overlap region. After realigning each half of OMcontig_28, the half that hadn't aligned was found to align to the end of the chromosome 5 FPC contig ctg255 (see Figure 3). This result suggests that either FPC contig ctg121 or ctg255 was incorrectly placed. In the same way, each half of the map contigs OMcontigs_1, 9, 12, 16, and 17 was also realigned, and this led to improved placements on the iMap. In all, these findings suggest that the current assigned locations of some FPC contigs (ctg166, ctg172, ctg180, ctg183, ctg197, ctg331, ctg332, ctg377 and ctg378) should be reevaluated.

We assessed the accuracy of BACop by analyzing the expected placement of 15 telomeric optical contigs onto the ends of chromosomes on the iMap. Figure 3 indeed shows their placement at chromosome ends: OMcontigs_23, 28, 31, 35, 38, 39, 47, and 61 are respectively anchored on the rightmost ends of Chrs 9, 5, 7, 8, 4, 2, 3, and 1. OMcontigs_7, 13, 16, 20, and 21 are respectively anchored on the leftmost ends of Chrs 5, 3, 8, 7, and 10. Also, OMcontig_51 is anchored on FPC contigs ctg368–370 without covering the leftmost end of FPC contig ctg367 on Chr 9, and OMcontig_8 is anchored on FPC contigs ctg405–420 without covering the rightmost end of FPC contig ctg421 on Chr 10. These results suggest that FPC contigs ctg367 and ctg421 should be placed elsewhere, since OMcontigs_51 and 8 have contig “edges” that may represent telomeric regions. The telomeric portion of OMcontig_28 is anchored on FPC contig ctg255 at the rightmost end of Chr 5, and the other portion of OMcontig_28 is anchored on FPC contig ctg121, which is placed on the iMap Chr 3 pericentromeric region. Our findings here indicate that FPC contig ctg121 probably should be joined with ctg255 on Chr 5.

### Optical versus sequence alignments identify discordances

**Figure 4:**
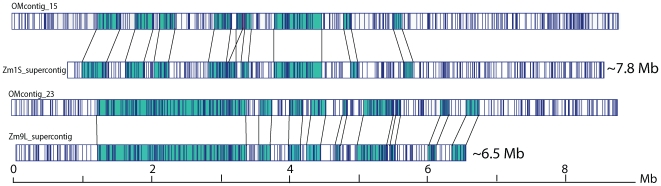
Comparative alignment of optical and pseudomolecule maps. *In silico* restriction maps of pseudomolecules (Zm1S_supercontig and Zm9L_supercontig) were found to align (Materials and Methods) to optical contigs (OMcontig_15 and 23). This allowed the identification of common and discordant regions. SwaI restriction sites are depicted by vertical lines. Regions of the optical contig and the pseudomolecule that align are teal colored, and the aligned regions are pointed to with black connecting lines.

We evaluated the quality of available and ongoing maize sequence assemblies by comparing optical contigs completely spanning large “supercontigs” (pseudomolecules) from Chrs 1, 3 and 9 [54] (Figure 4 and data not shown). Our alignments show 9 map segments in common, spanning 2.29 Mb (29.37%), between OMcontig_15 and the Zm1S_supercontig (Chr 1) *in silico* restriction map, and 9 in common between OMcontig_23 and Zm9L_supercontig (Chr 9) covering 3.62 Mb (54.85%). However, the Chr 3 finished supercontigs, corresponding to GenBank EF517601 and EF517600 [17], respectively, showed perfect alignment within OMcontigs_13 and 46, demonstrating the efficacy of our approach (data not shown). The lack of comprehensive alignment between the optical and the *in silico* maps for Chrs 1 and 9 pseudomolecules is not surprising because most of the sequenced BACs are in phase 1 assembly, awaiting the ordering and orienting of their associated sequence contigs. Accordingly, gaps of unknown size remain both within and between these nascent sequence assemblies. Based on these alignments of optical contigs *vs.* pseudomolecules, we characterized many of these gaps and identified issues with orientation. The assembly of the Zm9L_supercontig appears to be superior to that of the Zm1S_supercontig. This view is further buttressed by the higher proportion of phase 2 BAC sequences (28/56) in the Zm9L_supercontig than in the Zm1S_supercontig (14/60).

**Figure 5:**
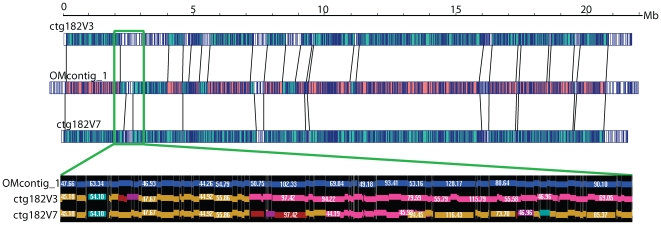
Optical maps guide ongoing construction of pseudomolecules. The quality of a large pseudomolecule (ctg182; ∼21.8 Mb) was successively improved by alignment of provisional builds against OMcontig_1 (optical contig). The increase in aligned regions of ctg182V7 to OMcontig_1 compared to the earlier version, ctg182V3, to the OMcontig demonstrates the improvement in the quality of the build. Red highlighting in OMcontig_1 shows optical contig regions aligning to both pseudomolecule versions. Maps of the ∼1.3 M region boxed in green are shown below, with concordances and discordances illustrated between the optical contig and ctg182V3 and V7 on a per restriction fragment basis. Gold colored fragments (boxes) signify concordance, while other colors signify discordance. Sizes are in kb. Ctg182V3 contains a run of pink fragments, indicating discordance with OMcontig_1, which is partially mediated in ctg182V7 as evidenced by greater alignment (less pink and more gold boxes).

The process of constructing a large sequence pseudomolecule is an iterative one, drawing support for provisional assembly from many sources that guide the serial generation of hypothetical builds and their subsequent validation. As such, we performed a series of optical *vs.* sequence contig alignments that tracked, guided, and validated the ongoing sequence finishing efforts of a ∼22 Mb sequence pseudomolecule (FPC ctg182) by the Arizona Genomics Institute (AGI). Figure 5 shows two versions of ctg182, V3 and V7, aligned to the optical contig—OMcontig_1. The earliest sequence contig build (V3) contained 12 segments that aligned (∼74.9%) with the optical contig, totaling 16.30 Mb. Rounds of directed sequence finishing effort led to the construction of the updated build, V7, which addressed discordances. V7 and the optical contig show an increased alignment of 89.6% with 8 larger segments aligning, totaling 19.51 Mb.

### Toward the AGP (B73 RefGen_v1): placement of 435 FPC contig pseudomolecules onto optical contig maps

The assembly of the accessioned golden path (AGP) involved the merging of 435 correctly ordered FPC pseudomolecules (90.2 kb–34.783 Mb; 2,061 Mb total) to span across the entire maize genome, and it is now known as the B73 RefGen_v1 [53],[79]. These FPC contig pseudomolecules are constructed from recent sequencing data (16,848 tiled BACs). We facilitated the construction of the AGP by ordering these 435 FPC pseudomolecules, using their alignment to our optical contigs to place them. We uniquely placed 338 of the 435 FPC pseudomolecules onto optical maps; 16 were placed on two optical map contigs, bridging two optical map contigs. Alignments also revealed two possible FPC chimeras (ctg84 and ctg299; Table S1). The remaining 82 FPC pseudomolecules (∼63 Mb) were not placed on optical contigs due to regions bearing few SwaI sites, or to problems in sequence assembly. Among the 338 uniquely placed FPC pseudomolecules, 65 (∼19%) are either newly placed (33; Table S1; blue rows) or reassigned to amended locations (32; Table S1; yellow rows). Overall, these results demonstrate the utility of a scalable optical map framework for guiding sequence assembly within a complex genomic environment.

### Comparing optical maps and B73 RefGen_v1 sequence

The accessioned golden path (AGP)(B73 RefGen_v1) from the Arizona Genomics Institute recently released by the maize genome sequencing consortium comprises 10 chromosome-wide “reference chromosomes” (pseudomolecules), and its assembly was guided by several physical maps, including the optical mapping findings presented here (http://www2.genome.arizona.edu/genomes/maize_contig_quality_table). The B73 RefGen_v1 reference chromosomes represent a unified genomic resource showing chromosome-wide placement of sequence and associated gaps. We provided an independent, optical reference map for this important resource via alignments that comprehensively revealed and sized sequence gaps in FPC pseudomolecules and the B73 RefGen_v1, which compose reference chromosomes. Local alignment reveals AGP assembly errors characterized as novel gaps, extra and/or missing cuts, and fragment sizing errors. In total, 1,102 optical contig segments (strings of contiguous restriction fragments) aligned to the B73 RefGen_v1 reference chromosomes (1,014.49 Mb, or ∼50% of AGP [2,046.35 Mb]; Table 2). The number of optical contig segments that align per chromosome ranges from 74 (Chr 10) to 159 (Chr 1), and the average map segment size is 937.67 kb (Table 2). The total aligned mass per chromosome varies from 64.65 Mb (Chr 8) to 166.15 Mb (Chr 1). The coverage by the aligned map segments for all the maize chromosomes ranges from 37.04% (Chr 8) to 59.15% (Chr 4) and averages 49.58% for all chromosomes (Table 2). Since the construction of the B73 RefGen is still ongoing, we expected that the optical map: B73 RefGen_v1 alignments would reveal a high level of discordance and an attenuated rate of optical contig alignment. A total of 4,465 discordances are identified (Table S2). These findings include 564 loci with extra sequence data, 829 revealing novel gaps or missing sequences, 2,348 misassemblies, 478 additional SwaI restriction sites, and 246 missing SwaI restriction sites.

**Table 2:** Statistics for optical map alignments against the *in silico* maps of the B73 RefGen_v1.

Chr. No.	Ref. Chr. Size (Mb)	No. of Aligned Map Segments	Ave. Map Segment Size (kb)	Total Aligned Map Segment Size (Mb)	% Coverage
1	300.24	159	1044.97	166.15	55.34
2	234.75	121	1258.93	125.33	53.39
3	230.56	132	873.33	115.28	50.00
4	247.10	109	1340.83	146.15	59.15
5	216.92	134	702.39	94.12	43.39
6	169.25	92	839.02	77.19	45.61
7	170.97	88	944.21	83.09	48.60
8	174.52	103	627.67	64.65	37.04
9	152.35	90	836.33	75.27	49.41
10	149.69	74	909.05	67.27	44.94
**Total/** ***Ave.***	**2046.35**	**1102**	***937.67***	**1014.49**	**49.58**

* Note: Chr. = chromosome, Ref. Chr. = reference chromosome, Ave. = average.

Gaps between adjacent FPC contigs were sized by alignments of optical contigs that span across them. Accordingly, gap size is determined by comparing optical map and B73 RefGen_v1 coordinates across a gap formed between neighboring FPC contigs in the B73 RefGen_v1 reference chromosomes. The FPC contigs do not continuously and seamlessly align to optical contigs since they are constructed from unfinished BACs (Figure 3 shows optical alignments to FPC contigs). Thus we estimated B73 RefGen_v1 gap sizes by considering the pair of coordinates on an aligned optical contig that most closely flank the spanned FPC gap (Figure S1). More precisely: Gap (kb) = [|(right optical coordinate)−(left optical coordinate)|−|(right sequence coordinate)−(left sequence coordinate)|]/1000. In this way, we characterized 263 gaps (Table S3) comprising 44 “negative gaps” (false B73 RefGen_v1 gaps, or novel sequence) and 219 “positive gaps” (confirmed FPC gaps, or unaccounted sequence). These 263 gaps were called taking the optical mapping sizing error per restriction fragment into consideration, which is typically +/−5% [80]. However, optical sizing errors can accrue in a complex way across long genomic regions that are spanned by summing consecutive restriction fragments [81]–[82]. As such, 169 of the 263 gap calls were conservatively made when the AGP and optical alignments differences were ≥10%, and the remainder was called below this threshold. Here differences <10% indicate the presence of gaps that were called with less confidence, but their tabulation provides considered targets for sequence bridging and filling. In all, 155 gaps were bridged by optical contigs (covering 36.59 Mb of gaps), and an extra 2.09 Mb of AGP pseudomolecule sequence was identified.

## Discussion

An optical map was created that spans across ∼91% of the maize (*Zea mays* L.) B73 inbred line (PI 550473) genome, which is a parent of the IBM mapping population. 66 optical contigs are included in this map representing 2,103.93 Mb of the maize genome decorated by 91,453 ordered SwaI restriction sites with accurate physical distances between these sites. On average, there is a SwaI site every 23 kb across the genome, and this restriction recognition sequence “marker” density is far greater than those on genetic (∼6,000 markers) and FPC (∼24,000 markers) maps [37] (http://maize-mapping.plantgenomics.iastate.edu/). Because the optical data format is a high-resolution ordered restriction map (SwaI), we were able to anchor and orient FPC-sequence contigs (http://www2.genome.arizona.edu/genomes/maize_contig_quality_table) onto this scaffold. While the immediate utility of the maize optical reference map is as an independent reference for sequence finishing and closing gaps, it will also drive comparative studies for unraveling complex patterns of structural variation as additional inbred lines and cultivars are mapped. Here the optical reference map would serve as a scaffold for future map assemblies enabling rapid discernment of genomic architecture. In this regard, optical mapping may be unique since large ∼500 kb molecules are directly mapped, and this advantage supports scalable genome analysis spanning from a restriction site to multi-megabase-sized regions.

The *de novo* approach that we used to construct the maize optical reference map ensures that it a unique, purely independent resource for sequence assembly and validation. (The ∼2.1 Gb map constructed *de novo* represents the largest created using single, genomic DNA molecules.) This physical map is free from common cloning and PCR artifacts, since individual genomic molecules are directly analyzed. These advantages are demonstrated by our comprehensive analysis of several pseudomolecules (Figure 4 and Figure 5), both published and ongoing, as well as B73 RefGen_v1 reference chromosomes (Table S3) spanning the entire maize genome.

**Figure 6:**
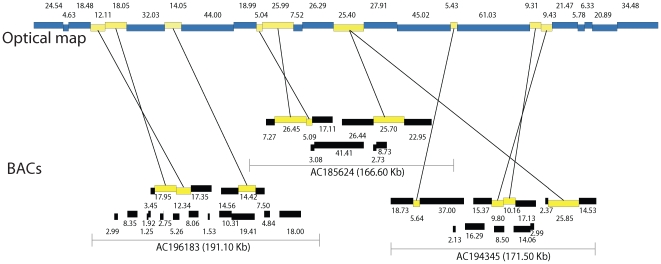
Alignment of unfinished BAC sequence contigs to optical contig maps. Matching restriction fragments in the BAC sub-contigs and the optical contig map are indicated by yellow boxes connected by lines; numbers show fragment size (kb). These alignments illustrate how BAC sequence contigs can be ordered and oriented using optical map alignments.

Our development of a new algorithm, named BACop (Materials and Methods; Figure 2, Figure 3, and Figure 6), greatly facilitated our ability to analyze and contribute to ongoing sequencing efforts. BACop specifically addressed issues of aligning nascent sequence builds of BAC clones, harboring multiple unordered and unoriented contigs (averaging 11 per BAC), against the optical reference map. Here, BACop was able to link optical contig maps to many unfinished BAC sequences already placed on the FPC map [37], and to presciently orient and order optical findings across all 10 maize chromosomes. Furthermore, BACop enabled the placement of 60 of the 66 optical contigs onto iMap and identified 12 FPC contigs whose current placement on the iMap requires reevaluation. The calls on 11 out of the 12 FPC contigs identified by BACop for replacements on the iMap (ctg121, ctg166, ctg172, ctg180, ctg183, ctg197, ctg332, ctg333, ctg367, ctg377 and ctg378) were also supported by comparative analysis of optical maps and *in silico* maps of the FPC contig sequence pseudomolecules (Table S1). The additional FPC contig identified for replacement by BACop (ctg421) was supported by other sequence markers indicating a new placement abutting ctg90 on chromosome 2 [79]. This BACop algorithm, in combination with optical map data, can also order and orient nascent sequence assemblies. As shown in Figure 6, many of the unordered and unoriented BAC subcontigs for several clones are nicely placed onto an optical map. Accordingly, BACop provides a useful tool for guiding the ongoing finishing of individual BACs.

Given the abundance of maize repetitive sequence, restriction maps directly constructed from ∼500 kb genomic molecules offer many advantages for spanning and structurally characterizing heterochromatic regions. This advantage is evidenced by the long contigs (3.64 Mb to 100.76 Mb) within the optical reference map, averaging 31.88 Mb in length. In comparison, the May 2006 maize FPC map comprises 721 FPC contigs averaging ∼2.98 Mb in length with a total mass of 2,150 Mb (300/721, or 163.7 Mb, are unassigned) [37]. Long optical contigs offer unique benefits especially when they span across genomic regions sparsely populated by markers, enabling structural insights to be drawn in these regions. In part, these advantages have characterized gaps and sequence misassemblies and reordered 19% of the FPC pseudomolecules (Table S1), which was persisted into the current B73 RefGen_v1 sequence for the maize genome.

Maize centromeres have been mapped to regions with flanking molecular markers using many different approaches [83]–[88]. However, for some maize chromosomes such as Chrs 1, 3, and 6, the proposed centromeric locations differ among different mapping methods; while for other chromosomes - Chrs 2, 4, 9, and 10 - there is a consensus running across different mapping techniques [84]. Recently, maize centromeres were located on the B73 RefGen_v1 sequence using centromeric markers [53] derived from numerous sources: transposon display, repeat junction, centromere repeat, and chromatin immunoprecipitation data [53]. Accordingly, we located centromeric loci assigned to Chrs 1, 2, 5, 6, 8, 9, and 10 around gaps in the optical contigs: [CEN1 (OMcontig_3 and 69); CEN2 (OMcontig_2 and 41); CEN5 (OMcontig_22 and 42); CEN6 (OMcontig_65 and 36); CEN8 (OMcontig_16 and 4); CEN 9 (OMcontig_55 and 17); CEN10 (OMcontig_49 and 17)] (data not shown). Unfortunately, the flanking contigs did not fully span any centromeric regions; however, these optical contigs did structurally characterize several pericentric regions.

Although we have demonstrated here that optical mapping offers numerous benefits for physical mapping and genome sequence assembly, its utility would be appreciably extended when combined with next generation sequencing. Genome analysis approaches are now rapidly evolving and tracking the increasingly cost-effective capabilities offered by next generation sequencing. Next generation sequencing approaches using single molecule libraries are now tackling the analysis of complex genomes [89]–[90], but they do not offer data sets competent for *de novo* assembly because of modest read lengths and errors. As such, new sequencing strategies must be developed for wheat and other complex crop genomes that effectively seize the new opportunities enabled by next generation sequencing. To this end, we propose the proactive use of optical mapping data for sequence assembly [91]. The combination of long-range (optical) and nucleotide-level (next generation) data sets, both generated directly from genomic molecules, may prove to be a cost-effective approach - especially when new algorithms are developed that intimately comingle both data sets during the assembly process.

## Materials and Methods

### Seed germination and DNA preparation

Maize kernels (inbred line B73, PI550473), obtained from the USDA-Agriculture Research Service North Central Regional Plant Introduction Station (Iowa State University, Ames, IA 50011-1170), were washed in 10% Clorox bleach for 10 min, rinsed in sterile water (3×, ∼3 min per wash), and germinated on moistened brown paper towels in a dark, moist chamber at 30°C for 12 days. Residual ungerminated seeds were removed from maize sprouts prior to nuclei isolation. The procedures for isolation of nuclei and storage have been described previously [75]. Prior to use, isolated nuclei were washed 2× with fresh Dulbecco's PBS (1.54 mM KH_2_PO_4_, 155.17 mM NaCl, 2.71 mM Na_2_HPO_4_, pH 7.2) to remove glycerol. Rapid DNA concentration assays were conducted by lysing small aliquots of nuclei in TE (10 mM Tris-Cl, pH 8.0, 1 mM EDTA) with 1 mg/ml proteinase K, and adenovirus DNA added at 25 pg/µl (internal sizing standard; Invitrogen, Carlsbad, CA), followed by mounting, restriction digestion, staining and imaging as previously described. Appropriate dilutions for mapping (optimized to minimize molecular crossovers) were made by adjusting the amount of isolated nuclei in the lysing solution (TE with 1 mg/ml proteinase K, 25 pg/µl adenovirus DNA in TE), by slowly pipetting up and down several times using a wide-bore pipette tip; samples were incubated at 65°C for 1 hr and at 37°C overnight. Samples were mounted onto optical mapping surfaces and imaged by fluorescence microscopy to assess DNA integrity and concentration of both genomic and reference standard DNA molecules.

### Surface preparation

Surface preparation was done as previously described [71]–[72]. Briefly, glass cover slips (22×22 mm, Fisher's Finest) were cleaned by boiling in Nano-Strip (Cyantek Corp., Freemont, CA), acidified by boiling in concentrated HCl, extensively rinsed with high purity water and ethanol under sonication, and derivatized using trimethyl and vinyl silanes to confer a positive charge and the means to crosslink the acrylamide overlay to the surface. Surfaces were evaluated by mounting lambda DASH II bacteriophage DNA (Stratagene, La Jolla, CA) and digesting them with 40 units of SwaI, diluted in 100 µL of digestion buffer containing 0.02% Triton X-100 at room temperature to determine the optimal digestion time (30 min to 2.5 hrs).

### DNA mapping, image acquisition, and processing

Genomic DNA molecules (∼400–500 kb) premixed with the adenovirus DNA sizing standards were deposited as stripes on derivatized glass surfaces using a silastic microchannel system [92]. A fully automated image acquisition and processing system collected data and compiled large files consisting of an ordered restriction map for each genomic DNA molecule. All microscope and camera functionalities and machine vision processes are fully automated and controlled by computer software. Detailed procedures were previously described [74]–[76],[92].

### Map assembly and cluster computing

With a raw map data set of >2 million maps, a divide and conquer strategy for optical map assembly was needed to deal with the severe computational load through parallel processing. We previously used this approach for the assembly of genome maps spanning the rice as well as the *Leishmania major* genomes [74]–[75]. Briefly, the map data set was divided into smaller sub-data sets (∼30,000 single molecule optical maps) allowing efficient parallel assembly [93]–[96], over 2–3 days, without taxing computer memory limits. The consensus maps from all contig assemblies were reassembled together for identifying redundant contigs and for merging overlapping optical consensus maps. After this reassembly process, a unique set of optical consensus maps was identified as “seed” maps for initiating iterative assembly. Iterative assembly consists of cycles of pairwise alignment [78] of the entire map data set against seed maps, followed by the contiging of these aligned single molecular optical maps for extending and refining seed maps in each subsequent cycle. Cycles of iterative assembly broaden and increase the coverage depth of nascent contigs.

Consensus maps are then stripped from the newly formed contigs as updated seed maps after further processing. The pairwise alignment phase extracted multiple high-scoring alignments based on the efficient linear scaling approach of Huang and Miller [97], and the confidence scores (p-values) were generated using an approach similar to that used by Waterman and Vingron [98]. Updated consensus maps were assembled to identify redundancy and to merge overlapping consensus maps. This process identified seed maps for the next iteration, and this iteration process was repeated typically more than ten times until the optical map contigs no longer grew. Large contigs (>10 Mb in breadth) also present computational challenges. For these contigs, iterative assembly considers and augments only the terminal 40 restriction fragments.

### Development of a new algorithm, BACop, for anchoring optical map contigs onto BAC sequences within iMap

About 85% of the maize genome comprises extensive families of repetitive sequences. Consequently, multiple contigs emerge from the sequence assembly of a single BAC, which are also unordered and unoriented. In order to integrate our optical map with the iMap, we developed a new algorithm —“**BACop**” — that utilizes unfinished BAC sequence data. The algorithm for anchoring the optical maps on the maize genetic-physical (FPC) map precedes in four distinct steps: *i*) matching restriction fragments between the optical map and the *in silico* restriction fragments from the sequence contigs of the BACs, *ii*) determining locations of all the BAC sequence contigs along the optical map, *iii*) anchoring the BACs on the optical maps, and *iv*) filtering and combining the alignments of BACs in the FPC map and optical map to find the most significant ones (Figure 2). The first step compares individual restriction fragment sizes from the optical map contigs with the *in silico* restriction fragments of the sequence contigs of the BACs. A fragment from the optical map assembly of size X and an *in silico* restriction fragment of size Y match if |X−Y|/σ√Y< = k, for parameters σ and k based on the statistical model developed by Valouev et al. 2006 [78]. Once the matching fragments have been determined, the BACs are located on the optical map assembly by examining each BAC's *in silico* restriction fragments. Approximate locations are determined by a filtration method that selects candidate regions on the optical map assembly that a BAC can align to, based on the matching fragment density. The approximate locations are further screened to produce a feasible alignment of a BAC's restriction fragments to the restriction fragments on the optical map assembly. Since each BAC is shotgun sequenced, multiple sequence contigs can result since the orientation and order of sequences are unknown. A feasible alignment must preserve the order of the *in silico* restriction fragments from within the same sequence contig but is allowed to match fragments from different sequence contigs in any order and orientation.

A match graph is constructed from a candidate region on the optical map assembly that the BAC can align to using the matching restriction fragments between the two as nodes within the graph. A traversal through the match graph induces an alignment of the BAC to the optical map assembly from the nodes representing matching fragments along the traversed path. The graph traversal resembles a branch and bound algorithm that exhaustively enumerates all feasible alignments and selects the best one. After all possible locations of the BACs are determined, the optical contig is then aligned to the FPC map. The FPC map gives the relative positions of the BACs within each FPC contig as well as the positions and order of the FPC contigs with respect to each other. Dynamic programming is used to align the optical map contigs to the FPC contigs by scoring for matching BACs along the optical contig that respect the order and location of BACs along the FPC map. A scoring scheme that weighs for higher quality BACs based on sequencing status and for BACs with greater fragment density is used. A fudge factor is applied when examining the locations of the BACs specified by the FPC map since they are approximate. Gaps between FPC contigs are specified using lower and upper bounds. The alignment is evaluated based on the number of BACs that are scored within the alignment region to remove spurious alignments according to a set threshold. The threshold is adjusted to allow for alignments in regions where there is sparse sequence data resulting in a lower number of usable BACs to align to. All of the alignments made with different thresholds are then collected, and the best alignments are selected according to coverage of the optical map assembly and number of aligned BACs.

## Supporting Information

Figure S1 Using optical contigs to estimate sizes of gaps between adjacent FPC contigs. (A) Restriction map view (box = restriction fragment) of FPC contigs aligned to the AGP Chr 6 pseudomolecule; the gap is highlighted. (B) Cartoon showing the alignments of FPC contigs ct280 and ctg281 to Chr6 pseudomolecule. A gap of unknown size is illustrated, with green lines showing alignment to the pseudomolecule. Dashed lines show alignments of ct280 and ctg281 against OMcontig_6 (gold track) and a large gap. (C) Restriction map view of OMcontig_6, locating the large gap within the grayed restriction fragments. FPC gap sizes are calculated as: Gap (kb) = [|(right optical coordinate)−(left optical coordinate)|−|(right sequence coordinate)−(left sequence coordinate)|]/1000.
(0.30 MB PDF)

Table S1 Ordering FPC contig sequence pseudomolecules based on the map alignments between optical maps and the *in silico* maps of the FPC contig sequence pseudomolecules.
(0.10 MB PDF)

Table S2 Discordances in the well-aligned map segments between optical maps and the *in silico* maps of the B73 RefGen_v1 reference chromosomes.
(0.25 MB PDF)

Table S3 FPC gap estimations based on map alignments between optical maps and the *in silico* maps of FPC contig sequence pseudomolecules and B73 RefGen_v1 reference chromosomes.
(0.12 MB PDF)
