# Supplementary material for: A Single Molecule Scaffold for the Maize Genome
Source: PLoS Genet. 2009 Nov 20;5(11):e1000711. doi: 10.1371/journal.pgen.1000711 (PMC2774507; doi:10.1371/journal.pgen.1000711)
Supplement: Figure S1 — Using optical contigs to estimate sizes of gaps between adjacent FPC contigs. (A) Restriction map view (box = restriction fragment) of FPC contigs aligned to the AGP Chr 6 pseudomolecule; the gap is highlighted. (B) Cartoon showing the alignments of FPC contigs ct280 and ctg281 to Chr6 pseudomolecule. A gap of unknown size is illustrated, with green lines showing alignment to the pseudomolecule. Dashed lines show alignments of ct280 and ctg281 against OMcontig_6 (gold track) and a large gap. (C) Restriction map view of OMcontig_6, locating the large gap within the grayed restriction fragments. FPC gap sizes are calculated as: Gap (kb) = [|(right optical coordinate)−(left optical coordinate)|−|(right sequence coordinate)−(left sequence coordinate)|]/1000. (0.30 MB PDF) [file pgen.1000711.s001.pdf]

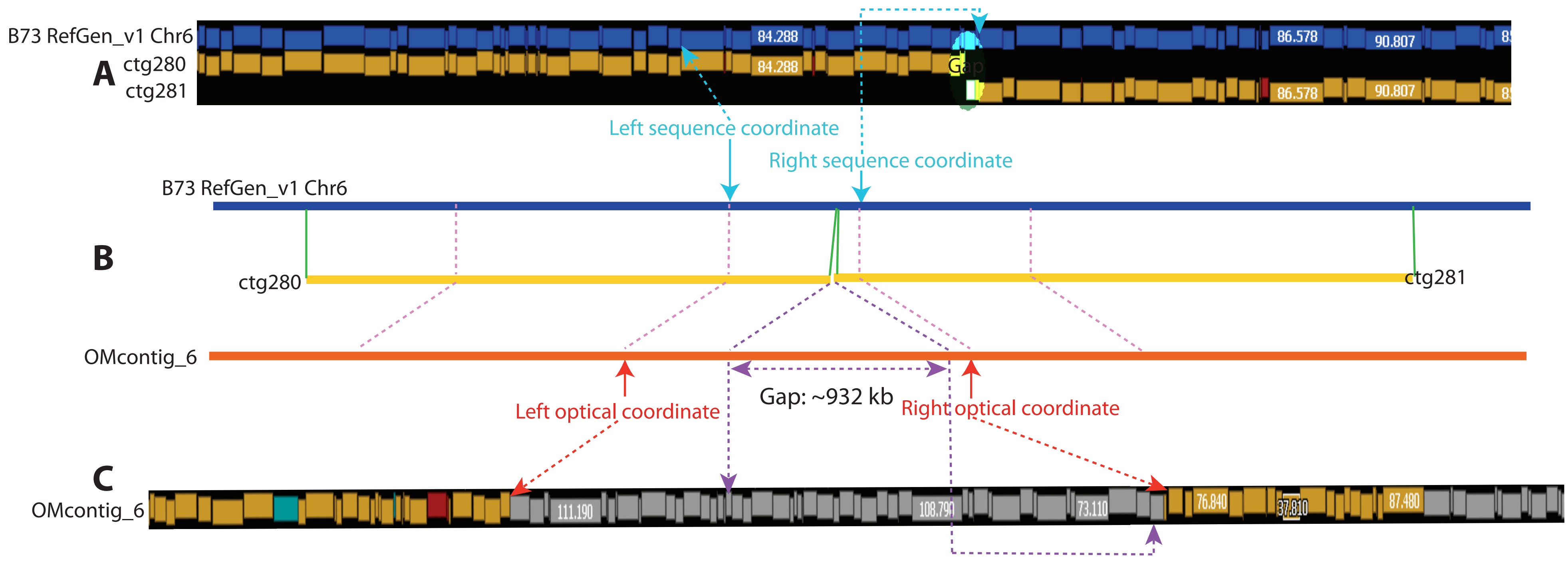

Figure S1. Using optical contigs to estimate sizes of gaps between adjacent FPC contigs. (A) Restriction map view (box = restriction fragment) of FPC contigs aligned to the AGP Chr 6 pseudomolecule; the gap is highlighted. (B) Cartoon showing the alignments of FPC contigs ct280 and ctg281 to Chr6 pseudomolecule. A gap of unknown size is illustrated, with green lines showing alignment to the pseudomolecule. Dashed lines show alignments of ct280 and ctg281 against OMcontig_6 (gold track) and a large gap. (C) Restriction map view of OMcontig_6, locating the large gap within the grayed restriction fragments. FPC gap sizes are calculated as:

Gap (kb) = [|(right optical coordinate) – (left optical coordinate)| - |(right sequence coordinate) – (left sequence coordinate)|]/1000
