# Supplementary material for: A Single Molecule Scaffold for the Maize Genome
Source: PLoS Genet. 2009 Nov 20;5(11):e1000711. doi: 10.1371/journal.pgen.1000711 (PMC2774507; doi:10.1371/journal.pgen.1000711)
Supplement: Table S1 — Ordering FPC contig sequence pseudomolecules based on the map alignments between optical maps and the in silico maps of the FPC contig sequence pseudomolecules. (0.10 MB PDF) [file pgen.1000711.s002.pdf]

## Ordering FPC contig sequence pseudomolecules based on the map alignments between optical maps and the *in silico* maps of the FPC contig sequence pseudomolecules

| FPC contig Seq Pseudo-molecule | Optical Map Contig | Coodinate On Optical Map (bp) | Coordinate On Optical Map (bp) | Aligned Map Segment Size (bp) | Comments | Anchored Chr No. BACop |
|---|---|---|---|---|---|---|
| ctg48 | OMcontig_0 | 796710 | 2536650 | 1739940 | | |
| ctg46 | OMcontig_0 | 3148900 | 14082050 | 10933150 | | |
| ctg492 | OMcontig_0 | 14130030 | 14535270 | 405240 | | |
| ctg45 | OMcontig_0 | 14716050 | 15871390 | 1155340 | | |
| ctg44 | OMcontig_0 | 16651910 | 22615680 | 5963770 | | |
| ctg43 | OMcontig_0 | 30247730 | 32959800 | 2712070 | | |
| ctg42 | OMcontig_0 | 34434210 | 34962240 | 528030 | | |
| ctg41 | OMcontig_0 | 41383940 | 43049850 | 1665910 | | |
| ctg40 | OMcontig_0 | 44199800 | 45018570 | 818770 | | |
| ctg39 | OMcontig_0 | 45824670 | 47406170 | 1581500 | | |
| ctg38 | OMcontig_0 | 48081620 | 53687010 | 5605390 | | |
| ctg15 | OMcontig_0 | 53747710 | 54649860 | 902150 | | |
| ctg37 | OMcontig_0 | 54661100 | 60064050 | 5402950 | | |
| ctg36 | OMcontig_0 | 60622690 | 69335470 | 8712780 | | |
| ctg33 | OMcontig_0 | 69536570 | 76828150 | 7291580 | | |
| ctg709 | OMcontig_0 | 76866440 | 77150340 | 283900 | | |
| ctg480 | OMcontig_0 | 77715110 | 78013010 | 297900 | | |
| ctg32 | OMcontig_0 | 78458840 | 83382460 | 4923620 | | |
| ctg31 | OMcontig_0 | 83552620 | 89847670 | 6295050 | | |
| ctg30 | OMcontig_0 | 94560210 | 95875190 | 1314980 | | |
| ctg196 | OMcontig_1 | 2022750 | 3329470 | 1306720 | | |
| ctg127 | OMcontig_1 | 6502800 | 10467970 | 3965170 | | |
| ctg195 | OMcontig_1 | 10953410 | 12588720 | 1635310 | | |
| ctg192 | OMcontig_1 | 12638860 | 13825000 | 1186140 | | |
| ctg469 | OMcontig_1 | 14500360 | 16813060 | 2312700 | | |
| ctg193 | OMcontig_1 | 17643560 | 23276290 | 5632730 | | |
| ctg194 | OMcontig_1 | 23472460 | 25840980 | 2368520 | | |
| ctg191 | OMcontig_1 | 26037820 | 27199480 | 1161660 | | |
| ctg188 | OMcontig_1 | 27719080 | 34764210 | 7045130 | | |
| ctg187 | OMcontig_1 | 35907890 | 37601190 | 1693300 | | |
| ctg184 | OMcontig_1 | 39396760 | 44342740 | 4945980 | | |
| ctg182 | OMcontig_1 | 54412500 | 57549610 | 3137110 | | |
| ctg181 | OMcontig_1 | 66805390 | 74057890 | 7252500 | | |
| ctg246 | OMcontig_1 | 74433760 | 76377300 | 1943540 | | |
| ctg179 | OMcontig_1 | 76684140 | 84337820 | 7653680 | | |
| ctg176 | OMcontig_1 | 84587610 | 91644620 | 7057010 | | |
| ctg173 | OMcontig_1 | 93781310 | 96624000 | 2842690 | | |
| ctg316 | OMcontig_10 | 945058 | 1553418 | 608360 | | |
| ctg315 | OMcontig_10 | 2616738 | 5180738 | 2564000 | | |
| ctg313 | OMcontig_10 | 5423598 | 7751148 | 2327550 | | |
| ctg312 | OMcontig_10 | 9394988 | 12790008 | 3395020 | | |
| ctg311 | OMcontig_10 | 14227398 | 15594248 | 1366850 | | |
| ctg309 | OMcontig_10 | 19559638 | 27227428 | 7667790 | | |
| ctg307 | OMcontig_10 | 29397558 | 32464938 | 3067380 | | |
| ctg306 | OMcontig_10 | 34195998 | 40900458 | 6704460 | | |
| ctg304 | OMcontig_10 | 41146318 | 47480458 | 6334140 | | |
| ctg470 | OMcontig_10 | 47627718 | 55218978 | 7591260 | | |
| ctg459 | OMcontig_10 | 55448078 | 57186398 | 1738320 | | |
| ctg149 | OMcontig_11 | 582930 | 3415270 | 2832340 | | |

| | | | | | | |
|---|---|---|---|---|---|---|
| ctg147 | OMcontig_11 | 3436890 | 5801860 | 2364970 | | |
| ctg145 | OMcontig_11 | 7353040 | 11333270 | 3980230 | | |
| ctg144 | OMcontig_11 | 15753910 | 16063490 | 309580 | | |
| ctg143 | OMcontig_11 | 17149360 | 17926000 | 776640 | | |
| ctg142 | OMcontig_11 | 17998830 | 20322670 | 2323840 | | |
| ctg141 | OMcontig_11 | 20391070 | 20837070 | 446000 | | |
| ctg141 | OMcontig_11 | 21836920 | 24153650 | 2316730 | | |
| ctg140 | OMcontig_11 | 24311110 | 25046590 | 735480 | | |
| ctg138 | OMcontig_11 | 28733280 | 32531810 | 3798530 | | |
| ctg136 | OMcontig_11 | 39222320 | 39713930 | 491610 | | |
| ctg135 | OMcontig_11 | 41557850 | 43600430 | 2042580 | | |
| ctg134 | OMcontig_11 | 43846040 | 45482580 | 1636540 | | |
| ctg131 | OMcontig_11 | 54249790 | 55049650 | 799860 | bridge between OMcontigs_11 and 12 | chr3 |
| ctg131 | OMcontig_12 | 3492077 | 10672467 | 7180390 | bridge between OMcontigs_11 and 12 | chr3 |
| ctg129 | OMcontig_12 | 11377387 | 14191577 | 2814190 | | |
| ctg128 | OMcontig_12 | 14223067 | 19409567 | 5186500 | | |
| ctg126 | OMcontig_12 | 22450927 | 25401967 | 2951040 | | |
| ctg124 | OMcontig_12 | 30993707 | 32380087 | 1386380 | | |
| ctg122 | OMcontig_12 | 50113707 | 51290347 | 1176640 | | |
| ctg118 | OMcontig_13 | 1495080 | 2950290 | 1455210 | bridge between OMcontigs_13 and 46 | chr3 |
| ctg63 | OMcontig_13 | 4310900 | 4881420 | 570520 | | |
| ctg117 | OMcontig_13 | 19733200 | 22933690 | 3200490 | | |
| ctg115 | OMcontig_13 | 27874350 | 36349210 | 8474860 | | |
| ctg113 | OMcontig_13 | 36619940 | 43164560 | 6544620 | | |
| ctg112 | OMcontig_13 | 43883480 | 44449260 | 565780 | | |
| ctg111 | OMcontig_13 | 49429160 | 54905300 | 5476140 | | |
| ctg231 | OMcontig_14 | 183930 | 2333050 | 2149120 | bridge between OMcontigs_14 and 42 | chr5 |
| ctg230 | OMcontig_14 | 2710040 | 3548730 | 838690 | | |
| ctg494 | OMcontig_14 | 4587740 | 7511050 | 2923310 | | |
| ctg233 | OMcontig_14 | 7856670 | 10878780 | 3022110 | | |
| ctg234 | OMcontig_14 | 23921710 | 26521410 | 2599700 | | |
| ctg399 | OMcontig_14 | 28694760 | 29254770 | 560010 | | |
| ctg237 | OMcontig_14 | 42812480 | 43968150 | 1155670 | | |
| ctg238 | OMcontig_14 | 48162520 | 49589030 | 1426510 | bridge between OMcontigs_14 and 25 | chr5 |
| ctg12 | OMcontig_15 | 0 | 2162490 | 2162490 | bridge between OMcontigs_3 and 15 | chr1 |
| ctg10 | OMcontig_15 | 16671420 | 17891530 | 1220110 | | |
| ctg9 | OMcontig_15 | 21014080 | 26309830 | 5295750 | | |
| ctg8 | OMcontig_15 | 26918510 | 32663960 | 5745450 | | |
| ctg6 | OMcontig_15 | 33911980 | 36290240 | 2378260 | | |
| ctg5 | OMcontig_15 | 37372880 | 39143830 | 1770950 | | |
| ctg4 | OMcontig_15 | 39933580 | 43980420 | 4046840 | | |
| ctg2 | OMcontig_15 | 46647900 | 47379100 | 731200 | | |
| ctg1 | OMcontig_15 | 48424680 | 49505730 | 1081050 | | |
| ctg326 | OMcontig_16 | 1753700 | 2844230 | 1090530 | | |
| ctg326 | OMcontig_16 | 11142190 | 11903850 | 761660 | | |
| ctg327 | OMcontig_16 | 16628040 | 17968400 | 1340360 | | |
| ctg328 | OMcontig_16 | 18067570 | 19027390 | 959820 | | |
| ctg329 | OMcontig_16 | 19512710 | 26529450 | 7016740 | | |
| ctg330 | OMcontig_16 | 27770220 | 32528650 | 4758430 | | |
| ctg331 | OMcontig_16 | 32779570 | 38277490 | 5497920 | | |
| ctg334 | OMcontig_16 | 39066410 | 45128560 | 6062150 | | |
| ctg457 | OMcontig_16 | 45758470 | 47206080 | 1447610 | | |
| ctg197 | OMcontig_17 | 360720 | 9112640 | 8751920 | | |
| ctg431 | OMcontig_17 | 9749340 | 10293140 | 543800 | | |
| ctg376 | OMcontig_17 | 11646990 | 14844440 | 3197450 | | |
| ctg380 | OMcontig_17 | 33569290 | 35925820 | 2356530 | | |

| | | | | | | |
|---|---|---|---|---|---|---|
| ctg473 | OMcontig_17 | 37062320 | 37437780 | 375460 | | |
| ctg707 | OMcontig_17 | 37583420 | 37900910 | 317490 | | |
| ctg382 | OMcontig_17 | 38918650 | 41399910 | 2481260 | | |
| ctg383 | OMcontig_17 | 41610250 | 47112880 | 5502630 | | |
| ctg214 | OMcontig_18 | 0 | 2038710 | 2038710 | | |
| ctg375 | OMcontig_18 | 5389450 | 8151130 | 2761680 | | |
| ctg374 | OMcontig_18 | 10338420 | 12008760 | 1670340 | | |
| ctg367 | OMcontig_18 | 12310110 | 16723470 | 4413360 | | |
| ctg378 | OMcontig_18 | 17885430 | 18865690 | 980260 | | |
| ctg377 | OMcontig_18 | 20748860 | 23842390 | 3093530 | | |
| ctg381 | OMcontig_18 | 25039730 | 26152260 | 1112530 | | |
| ctg490 | OMcontig_18 | 26265340 | 27294870 | 1029530 | | |
| ctg373 | OMcontig_18 | 40129480 | 40932400 | 802920 | | |
| ctg370 | OMcontig_18 | 44439920 | 46653860 | 2213940 | | |
| ctg52 | OMcontig_19 | 332840 | 3479400 | 3146560 | | |
| ctg51 | OMcontig_19 | 3858510 | 5218830 | 1360320 | | |
| ctg54 | OMcontig_19 | 5487970 | 9570280 | 4082310 | | |
| ctg56 | OMcontig_19 | 17887290 | 18615680 | 728390 | | |
| ctg60 | OMcontig_19 | 30368050 | 30835900 | 467850 | | |
| ctg61 | OMcontig_19 | 32521140 | 33948080 | 1426940 | | |
| ctg62 | OMcontig_19 | 34622340 | 37809280 | 3186940 | | |
| ctg63 | OMcontig_19 | 38549590 | 44855650 | 6306060 | | |
| ctg63 | OMcontig_19 | 46132000 | 46863800 | 731800 | bridge between OMcontigs_19 and 61 | chr1 |
| ctg68 | OMcontig_2 | 343300 | 3405540 | 3062240 | | |
| ctg69 | OMcontig_2 | 5050050 | 6082370 | 1032320 | | |
| ctg70 | OMcontig_2 | 8156490 | 10748150 | 2591660 | | |
| ctg71 | OMcontig_2 | 11738630 | 13979510 | 2240880 | | |
| ctg72 | OMcontig_2 | 14326050 | 16491190 | 2165140 | | |
| ctg74 | OMcontig_2 | 17503250 | 21302790 | 3799540 | | |
| ctg76 | OMcontig_2 | 30063410 | 34130320 | 4066910 | | |
| ctg77 | OMcontig_2 | 34909750 | 41063030 | 6153280 | | |
| ctg78 | OMcontig_2 | 47334460 | 48336460 | 1002000 | | |
| ctg79 | OMcontig_2 | 50890180 | 56650270 | 5760090 | | |
| ctg80 | OMcontig_2 | 62275360 | 64005800 | 1730440 | | |
| ctg81 | OMcontig_2 | 64316280 | 65620510 | 1304230 | | |
| ctg451 | OMcontig_2 | 66340140 | 68144840 | 1804700 | | |
| ctg82 | OMcontig_2 | 73535570 | 74425820 | 890250 | | |
| ctg302 | OMcontig_2 | 80058920 | 80801290 | 742370 | | |
| ctg83 | OMcontig_2 | 81781920 | 83324160 | 1542240 | | |
| ctg426 | OMcontig_2 | 83652300 | 84132010 | 479710 | | |
| ctg85 | OMcontig_2 | 84372510 | 85034480 | 661970 | | |
| ctg130 | OMcontig_2 | 85110710 | 87610790 | 2500080 | | |
| ctg87 | OMcontig_2 | 87701310 | 93541190 | 5839880 | | |
| ctg293 | OMcontig_20 | 1229000 | 2706580 | 1477580 | | |
| ctg294 | OMcontig_20 | 3900800 | 4732340 | 831540 | | |
| ctg487 | OMcontig_20 | 5802020 | 6457260 | 655240 | | |
| ctg296 | OMcontig_20 | 6535150 | 13975250 | 7440100 | | |
| ctg297 | OMcontig_20 | 15111380 | 19543150 | 4431770 | | |
| ctg298 | OMcontig_20 | 21404040 | 23368210 | 1964170 | | |
| ctg299 | OMcontig_20 | 25472330 | 31042100 | 5569770 | chimeric | chr7 |
| ctg300 | OMcontig_20 | 32270490 | 39559900 | 7289410 | | |
| ctg301 | OMcontig_20 | 39801350 | 42448450 | 2647100 | bridge between OMcontigs_20 and 43 | chr7 |
| ctg84 | OMcontig_21 | 0 | 4473900 | 4473900 | chimeric | chr10 |
| ctg398 | OMcontig_21 | 4591900 | 5305250 | 713350 | | |
| ctg397 | OMcontig_21 | 13850790 | 16326570 | 2475780 | | |
| ctg395 | OMcontig_21 | 16693110 | 17350320 | 657210 | | |

| | | | | | | |
|---|---|---|---|---|---|---|
| ctg394 | OMcontig_21 | 26095680 | 29224400 | 3128720 | | |
| ctg393 | OMcontig_21 | 29290290 | 32605290 | 3315000 | | |
| ctg392 | OMcontig_21 | 35534990 | 40175690 | 4640700 | | |
| ctg180 | OMcontig_22 | 592130 | 6048360 | 5456230 | | |
| ctg228 | OMcontig_22 | 6048360 | 10212290 | 4163930 | | |
| ctg227 | OMcontig_22 | 10935320 | 16154210 | 5218890 | | |
| ctg225 | OMcontig_22 | 20015620 | 21377270 | 1361650 | | |
| ctg223 | OMcontig_22 | 35431030 | 35968220 | 537190 | bridge between OMcontigs_7 and 22 | chr5 |
| ctg384 | OMcontig_23 | 1706440 | 3114100 | 1407660 | | |
| ctg385 | OMcontig_23 | 3557540 | 10145070 | 6587530 | | |
| ctg386 | OMcontig_23 | 12869520 | 14225110 | 1355590 | | |
| ctg387 | OMcontig_23 | 14391070 | 19498640 | 5107570 | | |
| ctg388 | OMcontig_23 | 19987280 | 20981370 | 994090 | | |
| ctg389 | OMcontig_23 | 21044560 | 26640320 | 5595760 | | |
| ctg390 | OMcontig_23 | 27573990 | 28895760 | 1321770 | | |
| ctg391 | OMcontig_23 | 29466260 | 36971370 | 7505110 | | |
| ctg160 | OMcontig_24 | 191030 | 6728400 | 6537370 | | |
| ctg531 | OMcontig_24 | 7104460 | 7848020 | 743560 | | |
| ctg162 | OMcontig_24 | 9586050 | 10352710 | 766660 | | |
| ctg163 | OMcontig_24 | 11542650 | 14344590 | 2801940 | | |
| ctg163 | OMcontig_24 | 15119760 | 15534980 | 415220 | | |
| ctg164 | OMcontig_24 | 22799200 | 23573830 | 774630 | | |
| ctg238 | OMcontig_25 | 1195600 | 11338910 | 10143310 | bridge between OMcontigs_14 and 25 | chr5 |
| ctg242 | OMcontig_25 | 12678950 | 16586530 | 3907580 | | |
| ctg244 | OMcontig_25 | 19154770 | 19724700 | 569930 | | |
| ctg245 | OMcontig_25 | 20109950 | 23896350 | 3786400 | | |
| ctg247 | OMcontig_25 | 24152690 | 25678800 | 1526110 | | |
| ctg104 | OMcontig_26 | 5214940 | 6805110 | 1590170 | | |
| ctg103 | OMcontig_26 | 13196570 | 18169600 | 4973030 | | |
| ctg103 | OMcontig_26 | 19054960 | 19531060 | 476100 | | |
| ctg102 | OMcontig_26 | 19677430 | 20587330 | 909900 | | |
| ctg101 | OMcontig_26 | 21595240 | 22431250 | 836010 | | |
| ctg99 | OMcontig_26 | 24984680 | 27021540 | 2036860 | | |
| ctg320 | OMcontig_27 | 5357340 | 6501850 | 1144510 | | |
| ctg318 | OMcontig_27 | 12974130 | 15564280 | 2590150 | | |
| ctg318 | OMcontig_27 | 22017340 | 22521230 | 503890 | | |
| ctg317 | OMcontig_27 | 24465800 | 25296250 | 830450 | | |
| ctg255 | OMcontig_28 | 10513170 | 11301480 | 788310 | | |
| ctg121 | OMcontig_28 | 13984010 | 15905090 | 1921080 | | |
| ctg170 | OMcontig_29 | 1968310 | 3301760 | 1333450 | | |
| ctg171 | OMcontig_29 | 10275500 | 13293820 | 3018320 | | |
| ctg437 | OMcontig_3 | 472010 | 1571530 | 1099520 | | |
| ctg433 | OMcontig_3 | 3235290 | 7695750 | 4460460 | | |
| ctg471 | OMcontig_3 | 7908150 | 10018740 | 2110590 | | |
| ctg29 | OMcontig_3 | 10528810 | 17314200 | 6785390 | | |
| ctg28 | OMcontig_3 | 24097640 | 26283490 | 2185850 | | |
| ctg27 | OMcontig_3 | 31662120 | 32572420 | 910300 | | |
| ctg26 | OMcontig_3 | 33259950 | 38881040 | 5621090 | | |
| ctg24 | OMcontig_3 | 39630030 | 42612170 | 2982140 | | |
| ctg23 | OMcontig_3 | 43687770 | 47310750 | 3622980 | | |
| ctg22 | OMcontig_3 | 48272490 | 49355580 | 1083090 | | |
| ctg20 | OMcontig_3 | 49916720 | 55788960 | 5872240 | | |
| ctg19 | OMcontig_3 | 57416480 | 58013490 | 597010 | | |
| ctg18 | OMcontig_3 | 61112160 | 63303440 | 2191280 | | |
| ctg17 | OMcontig_3 | 65169560 | 65882670 | 713110 | | |
| ctg16 | OMcontig_3 | 66135490 | 68003030 | 1867540 | | |

| | | | | | | |
|---|---|---|---|---|---|---|
| ctg474 | OMcontig_3 | 68064950 | 68621150 | 556200 | | |
| ctg445 | OMcontig_3 | 69447560 | 69791630 | 344070 | | |
| ctg14 | OMcontig_3 | 80644330 | 82627190 | 1982860 | | |
| ctg12 | OMcontig_3 | 83649280 | 85359450 | 1710170 | bridge between OMcontigs_3 and 15 | chr1 |
| ctg261 | OMcontig_30 | 2174370 | 3006860 | 832490 | | |
| ctg262 | OMcontig_30 | 4120240 | 4833130 | 712890 | | |
| ctg262 | OMcontig_30 | 8603190 | 14897860 | 6294670 | | |
| ctg268 | OMcontig_30 | 16250860 | 18901660 | 2650800 | | |
| ctg267 | OMcontig_30 | 19777720 | 24163570 | 4385850 | bridge between OMcontigs_30 and 60 | chr6 |
| ctg322 | OMcontig_31 | 0 | 1365530 | 1365530 | bridge between OMcontigs_31 and 68 | chr7 |
| ctg322 | OMcontig_31 | 3827560 | 9188580 | 5361020 | | |
| ctg325 | OMcontig_31 | 21179070 | 22250600 | 1071530 | | |
| ctg501 | OMcontig_31 | 23313050 | 23598030 | 284980 | | |
| ctg354 | OMcontig_32 | 219530 | 4909730 | 4690200 | bridge between OMcontigs_4 and 32 | |
| ctg355 | OMcontig_32 | 5094330 | 5763660 | 669330 | | |
| ctg356 | OMcontig_32 | 6833690 | 9364750 | 2531060 | | |
| ctg358 | OMcontig_32 | 16346700 | 17228360 | 881660 | | |
| ctg254 | OMcontig_33 | 599730 | 4705530 | 4105800 | | |
| ctg253 | OMcontig_33 | 4754790 | 9503880 | 4749090 | | |
| ctg251 | OMcontig_33 | 10698180 | 12199050 | 1500870 | | |
| ctg250 | OMcontig_33 | 14878610 | 20793700 | 5915090 | | |
| ctg249 | OMcontig_33 | 21198620 | 22492960 | 1294340 | | |
| ctg271 | OMcontig_34 | 910700 | 1935920 | 1025220 | | |
| ctg270 | OMcontig_34 | 7935080 | 10682610 | 2747530 | | |
| ctg269 | OMcontig_34 | 15282180 | 20735580 | 5453400 | bridge between OMcontigs_34 and 36 | chr? |
| ctg366 | OMcontig_35 | 772330 | 3688600 | 2916270 | | |
| ctg365 | OMcontig_35 | 3812410 | 4129860 | 317450 | | |
| ctg364 | OMcontig_35 | 4165030 | 5807280 | 1642250 | | |
| ctg363 | OMcontig_35 | 7115000 | 11062630 | 3947630 | | |
| ctg362 | OMcontig_35 | 11625950 | 15347760 | 3721810 | | |
| ctg360 | OMcontig_35 | 15491120 | 19129260 | 3638140 | | |
| ctg269 | OMcontig_36 | 66710 | 5021400 | 4954690 | bridge between OMcontigs_34 and 36 | chr6 |
| ctg442 | OMcontig_36 | 5112110 | 6427230 | 1315120 | | |
| ctg265 | OMcontig_36 | 7974930 | 10370510 | 2395580 | | |
| ctg401 | OMcontig_37 | 339550 | 827110 | 487560 | bridge between OMcontigs_37 and 45 | chr10 |
| ctg400 | OMcontig_37 | 8507210 | 10152920 | 1645710 | | |
| ctg399 | OMcontig_37 | 10949170 | 18143530 | 7194360 | | |
| ctg399 | OMcontig_37 | 19280850 | 19962360 | 681510 | | |
| ctg199 | OMcontig_38 | 431650 | 6131320 | 5699670 | | |
| ctg198 | OMcontig_38 | 6285160 | 8084860 | 1799700 | | |
| ctg200 | OMcontig_38 | 8698490 | 10375130 | 1676640 | | |
| ctg201 | OMcontig_38 | 11623920 | 13775720 | 2151800 | | |
| ctg202 | OMcontig_38 | 13852420 | 15432800 | 1580380 | | |
| ctg109 | OMcontig_39 | 2627290 | 6167340 | 3540050 | | |
| ctg108 | OMcontig_39 | 6222470 | 8097380 | 1874910 | | |
| ctg354 | OMcontig_4 | 454170 | 8478390 | 8024220 | bridge between OMcontigs_4 and 32 | |
| ctg353 | OMcontig_4 | 8862800 | 11117230 | 2254430 | | |
| ctg352 | OMcontig_4 | 12072640 | 13232960 | 1160320 | | |
| ctg350 | OMcontig_4 | 13459180 | 17588730 | 4129550 | | |
| ctg349 | OMcontig_4 | 18301350 | 23411410 | 5110060 | | |
| ctg348 | OMcontig_4 | 26074450 | 26940420 | 865970 | | |
| ctg348 | OMcontig_4 | 27439320 | 27995280 | 555960 | | |
| ctg347 | OMcontig_4 | 29733890 | 30485220 | 751330 | | |
| ctg346 | OMcontig_4 | 31778350 | 32546820 | 768470 | | |
| ctg345 | OMcontig_4 | 33470990 | 37772330 | 4301340 | | |
| ctg340 | OMcontig_4 | 43954560 | 44929360 | 974800 | | |

| | | | | | | |
|---|---|---|---|---|---|---|
| ctg337 | OMcontig_4 | 47902860 | 48656400 | 753540 | | |
| ctg338 | OMcontig_4 | 48826370 | 53595020 | 4768650 | | |
| ctg344 | OMcontig_4 | 53806960 | 60326290 | 6519330 | | |
| ctg339 | OMcontig_4 | 60531400 | 62443800 | 1912400 | | |
| ctg336 | OMcontig_4 | 62578740 | 65264320 | 2685580 | | |
| ctg333 | OMcontig_4 | 72419390 | 75804770 | 3385380 | | |
| ctg343 | OMcontig_4 | 75916180 | 79375630 | 3459450 | | |
| ctg332 | OMcontig_4 | 80133560 | 84259980 | 4126420 | | |
| ctg167 | OMcontig_40 | 7663720 | 12831630 | 5167910 | | |
| ctg167 | OMcontig_40 | 13495430 | 14714000 | 1218570 | | |
| ctg86 | OMcontig_41 | 6703900 | 7755290 | 1051390 | bridge between OMcontigs_5 and 41 | chr? |
| ctg449 | OMcontig_41 | 10020230 | 13145080 | 3124850 | | |
| ctg231 | OMcontig_42 | 12421300 | 14314230 | 1892930 | bridge between OMcontigs_14 and 42 | chr5 |
| ctg301 | OMcontig_43 | 264420 | 8436320 | 8171900 | bridge between OMcontigs_20 and 43 | chr7 |
| ctg303 | OMcontig_43 | 10320740 | 10989840 | 669100 | | |
| ctg456 | OMcontig_43 | 11429640 | 12436830 | 1007190 | | |
| ctg159 | OMcontig_44 | 490310 | 1846670 | 1356360 | | |
| ctg158 | OMcontig_44 | 2791010 | 5481830 | 2690820 | | |
| ctg156 | OMcontig_44 | 5880880 | 12714950 | 6834070 | | |
| ctg401 | OMcontig_45 | 322050 | 4535370 | 4213320 | bridge between OMcontigs_37 and 45 | chr10 |
| ctg402 | OMcontig_45 | 4725850 | 6420790 | 1694940 | | |
| ctg403 | OMcontig_45 | 7799700 | 10007140 | 2207440 | | |
| ctg404 | OMcontig_45 | 11159460 | 13043600 | 1884140 | | |
| ctg118 | OMcontig_46 | 153120 | 1395710 | 1242590 | bridge between OMcontigs_13 and 46 | chr3 |
| ctg119 | OMcontig_46 | 1956150 | 8977020 | 7020870 | | |
| ctg120 | OMcontig_46 | 11480350 | 11854570 | 374220 | | |
| ctg149 | OMcontig_47 | 0 | 546350 | 546350 | | |
| ctg150 | OMcontig_47 | 1005530 | 5993210 | 4987680 | | |
| ctg151 | OMcontig_47 | 6539160 | 10649220 | 4110060 | | |
| ctg152 | OMcontig_47 | 11142250 | 12263750 | 1121500 | | |
| ctg256 | OMcontig_48 | 592170 | 2262870 | 1670700 | | |
| ctg257 | OMcontig_48 | 3022880 | 5186920 | 2164040 | | |
| ctg259 | OMcontig_48 | 5923520 | 6423320 | 499800 | | |
| ctg260 | OMcontig_48 | 8209040 | 10523870 | 2314830 | | |
| ctg84 | OMcontig_49 | 1733670 | 2160720 | 427050 | | |
| ctg84 | OMcontig_49 | 2881870 | 10983030 | 8101160 | chimeric | chr2 |
| ctg86 | OMcontig_5 | 701600 | 7352670 | 6651070 | bridge between OMcontigs_5 and 41 | chr2 |
| ctg467 | OMcontig_5 | 8354890 | 9570700 | 1215810 | | |
| ctg89 | OMcontig_5 | 21629270 | 23876260 | 2246990 | | |
| ctg90 | OMcontig_5 | 53107040 | 58417460 | 5310420 | | |
| ctg92 | OMcontig_5 | 63218280 | 66783310 | 3565030 | | |
| ctg95 | OMcontig_5 | 67868650 | 69234940 | 1366290 | | |
| ctg96 | OMcontig_5 | 69557110 | 74897060 | 5339950 | | |
| ctg97 | OMcontig_5 | 75715990 | 76287040 | 571050 | | |
| ctg98 | OMcontig_5 | 76695330 | 83008220 | 6312890 | | |
| ctg120 | OMcontig_50 | 546900 | 2703370 | 2156470 | bridge between OMcontigs_50 and 57 | chr3 |
| ctg369 | OMcontig_51 | 1619700 | 1953290 | 333590 | | |
| ctg368 | OMcontig_51 | 2681010 | 6581240 | 3900230 | | |
| ctg441 | OMcontig_51 | 9547040 | 9943430 | 396390 | | |
| ctg272 | OMcontig_53 | 2623480 | 4341790 | 1718310 | | |
| ctg273 | OMcontig_53 | 5870880 | 6899970 | 1029090 | | |
| ctg448 | OMcontig_55 | 365090 | 3250540 | 2885450 | | |
| ctg432 | OMcontig_55 | 5086490 | 7978350 | 2891860 | bridge between OMcontigs_55 and 58 | unknown |
| ctg49 | OMcontig_56 | 806320 | 7508590 | 6702270 | | |
| ctg299 | OMcontig_57 | 362910 | 2580300 | 2217390 | chimeric | chr? |
| ctg120 | OMcontig_57 | 2706020 | 7819050 | 5113030 | bridge between OMcontigs_50 and 57 | chr? |

| | | | | | | |
|---|---|---|---|---|---|---|
| ctg432 | OMcontig_58 | 357983 | 1730798 | 1372815 | bridge between OMcontigs_55 and 58 | chr2 |
| ctg106 | OMcontig_58 | 3242282 | 6165585 | 2923303 | | |
| ctg50 | OMcontig_59 | 932210 | 5515160 | 4582950 | | |
| ctg274 | OMcontig_6 | 732580 | 3778830 | 3046250 | | |
| ctg276 | OMcontig_6 | 4845090 | 7018840 | 2173750 | | |
| ctg280 | OMcontig_6 | 8871580 | 11981100 | 3109520 | | |
| ctg477 | OMcontig_6 | 12072580 | 12673340 | 600760 | | |
| ctg281 | OMcontig_6 | 19003800 | 20788970 | 1785170 | | |
| ctg282 | OMcontig_6 | 30090450 | 32460570 | 2370120 | | |
| ctg283 | OMcontig_6 | 33288980 | 40961260 | 7672280 | | |
| ctg284 | OMcontig_6 | 41818020 | 42918330 | 1100310 | | |
| ctg285 | OMcontig_6 | 43675540 | 50779370 | 7103830 | | |
| ctg286 | OMcontig_6 | 50809350 | 51478020 | 668670 | | |
| ctg287 | OMcontig_6 | 62649620 | 64605930 | 1956310 | | |
| ctg289 | OMcontig_6 | 65363110 | 68853800 | 3490690 | | |
| ctg267 | OMcontig_60 | 59840 | 4440020 | 4380180 | bridge between OMcontigs_30 and 60 | chr6 |
| ctg266 | OMcontig_60 | 4958340 | 5526010 | 567670 | | |
| ctg67 | OMcontig_61 | 626320 | 1468470 | 842150 | | |
| ctg63 | OMcontig_61 | 2457500 | 5691380 | 3233880 | bridge between OMcontigs_19 and 61 | chr1 |
| ctg106 | OMcontig_64 | 986060 | 4429643 | 3443583 | | |
| ctg264 | OMcontig_65 | 903488 | 3682295 | 2778807 | | |
| ctg107 | OMcontig_66 | 428251 | 1336793 | 908542 | | |
| ctg105 | OMcontig_66 | 1680043 | 2605512 | 925469 | | |
| ctg263 | OMcontig_67 | 1088547 | 3467200 | 2378653 | | |
| ctg322 | OMcontig_68 | 656472 | 3258468 | 2601996 | bridge between OMcontigs_31 and 68 | chr7 |
| ctg30 | OMcontig_69 | 2267939 | 2536863 | 268924 | | |
| ctg204 | OMcontig_7 | 449900 | 5863740 | 5413840 | | |
| ctg206 | OMcontig_7 | 7487750 | 8450690 | 962940 | | |
| ctg207 | OMcontig_7 | 11269850 | 11697110 | 427260 | | |
| ctg209 | OMcontig_7 | 12309300 | 14628620 | 2319320 | | |
| ctg210 | OMcontig_7 | 15823880 | 17780830 | 1956950 | | |
| ctg211 | OMcontig_7 | 18805400 | 21232980 | 2427580 | | |
| ctg212 | OMcontig_7 | 22410610 | 24362880 | 1952270 | | |
| ctg212 | OMcontig_7 | 26283330 | 29437820 | 3154490 | | |
| ctg215 | OMcontig_7 | 31544260 | 32123470 | 579210 | | |
| ctg216 | OMcontig_7 | 32322040 | 32981430 | 659390 | | |
| ctg217 | OMcontig_7 | 33346600 | 39444800 | 6098200 | | |
| ctg218 | OMcontig_7 | 39720820 | 46109610 | 6388790 | | |
| ctg219 | OMcontig_7 | 47668220 | 48466300 | 798080 | | |
| ctg219 | OMcontig_7 | 49500280 | 56508330 | 7008050 | | |
| ctg220 | OMcontig_7 | 56552780 | 63849070 | 7296290 | | |
| ctg223 | OMcontig_7 | 66194760 | 68544060 | 2349300 | bridge between OMcontigs_7 and 22 | chr5 |
| ctg420 | OMcontig_8 | 371700 | 1471030 | 1099330 | | |
| ctg419 | OMcontig_8 | 2211110 | 4551290 | 2340180 | | |
| ctg417 | OMcontig_8 | 6397120 | 8823130 | 2426010 | | |
| ctg414 | OMcontig_8 | 10333980 | 16502860 | 6168880 | | |
| ctg413 | OMcontig_8 | 17877540 | 18970360 | 1092820 | | |
| ctg413 | OMcontig_8 | 21664980 | 28191520 | 6526540 | | |
| ctg412 | OMcontig_8 | 28832650 | 35769130 | 6936480 | | |
| ctg411 | OMcontig_8 | 46682770 | 48491170 | 1808400 | | |
| ctg410 | OMcontig_8 | 50018780 | 51596530 | 1577750 | | |
| ctg409 | OMcontig_8 | 51707550 | 52183780 | 476230 | | |
| ctg408 | OMcontig_8 | 58642300 | 59160420 | 518120 | | |
| ctg406 | OMcontig_8 | 61778520 | 63884590 | 2106070 | | |
| ctg405 | OMcontig_8 | 64484120 | 65635470 | 1151350 | | |
| ctg427 | OMcontig_9 | 2268846 | 2646426 | 377580 | | |

| | | | | | | |
|---|---|---|---|---|---|---|
| ctg183 | OMcontig_9 | 4140126 | 4531716 | 391590 | | |
| ctg169 | OMcontig_9 | 16426206 | 19107396 | 2681190 | | |
| ctg444 | OMcontig_9 | 22224696 | 23453236 | 1228540 | | |
| ctg168 | OMcontig_9 | 23766706 | 26508836 | 2742130 | | |
| ctg174 | OMcontig_9 | 26625226 | 29671506 | 3046280 | | |
| ctg172 | OMcontig_9 | 30016816 | 39159646 | 9142830 | | |
| ctg166 | OMcontig_9 | 40206476 | 45539016 | 5332540 | | |
| ctg165 | OMcontig_9 | 45704006 | 52288476 | 6584470 | | |
| ctg190 | OMcontig_9 | 53563766 | 54244996 | 681230 | | |
| ctg435 | OMcontig_9 | 57519056 | 59081076 | 1562020 | | |

**Note:** Red letters indicate the FPC contig sequence pseudomolecule was aligned to more than one optical map contig;
Blue rows indicate the FPC contig sequence pseudomolecules were not originally placed on the maize iMap;
Yellow rows indicate that the placement of the FPC contig sequence pseudomolecule is inconsistent with the iMap.
