# Supplementary material for: A Single Molecule Scaffold for the Maize Genome
Source: PLoS Genet. 2009 Nov 20;5(11):e1000711. doi: 10.1371/journal.pgen.1000711 (PMC2774507; doi:10.1371/journal.pgen.1000711)
Supplement: Table S2 — Discordances in the well-aligned map segments between optical maps and the in silico maps of the B73 RefGen_v1 reference chromosomes. (0.25 MB PDF) [file pgen.1000711.s003.pdf]

Discordances in the well-aligned map segments between optical maps and the *in silico* maps of the B73 RefGen_v1 reference chromosomes

Discordance ID	Chr. No.	Start Coordinate (bp)	End Coordinate (bp)	Cut Classification	Size Classification	Start Location	End Location	Cut Location	Length Change	Length P Value	Cut P Value	Optical Map Span	Optical Map Align	Variation type	p value	comments
1-381	1	124387615	124415360	EC	Extra sequence included	124387615	124415360		-5621	7.12E-23		59	40	DEL	7.12E-23	
1-599	1	204871100	204922921	EC	Extra sequence included	204871100	204922921		-5998	1.50E-18		68	34	DEL	1.50E-18	
2-213	2	81943946	82028026	EC	Extra sequence included	81943946	82028026		-27273	7.27E-23		45	39	DEL	7.27E-23	
2-250	2	96078634	96136949	EC	Extra sequence included	96078634	96136949		-21440	7.27E-23		69	52	DEL	7.27E-23	
3-66	3			EC	Extra sequence included	37063663	37138218		-17544	7.24E-23		40	35	DEL	7.24E-23	
3-204	3	83247546	83300973	EC	Extra sequence included	83247546	83300973		-10434	7.24E-23		50	32	DEL	7.24E-23	
3-380	3	163225783	163291740	EC	Extra sequence included	163225783	163291740		-12749	1.08E-22		58	42	DEL	1.08E-22	
3-500	3	211484020	211614840	EC	Extra sequence included	211484020	211614840		-39971	7.24E-23		47	42	DEL	7.24E-23	
4-252	4	96201978	96390772	EC	Extra sequence included	96201978	96390772		-20135	7.53E-13		24	24	DEL	7.53E-13	
4-365	4	145712333	145787153	EC	Extra sequence included	145712333	145787153		-8322	7.57E-23		63	52	DEL	7.57E-23	
8-165	8	56026565	56111289	EC	Extra sequence included	56026565	56111289		-52245	8.64E-23		73	57	DEL	8.64E-23	
1-3	1	2232916	2232916	EC	NONE			2232916	0		5.44E-23	61	52	EC	5.44E-23	
1-4	1	2329308	2329308	EC	NONE			2329308	0		1.62E-31	58	53	EC	1.62E-31	
1-9	1	5242930	5242930	EC	NONE			5242930	0		3.17E-43	57	47	EC	3.17E-43	
1-24	1	9573678	9573678	EC	NONE			9573678	0		1.34E-36	77	67	EC	1.34E-36	
1-32	1	11190068	11190068	EC	NONE			11190068	0		8.61E-08	30	27	EC	8.61E-08	
1-36	1	12008871	12008871	EC	NONE			12008871	0		5.90E-37	79	68	EC	5.90E-37	
1-37	1	12202222	12202222	EC	NONE			12202222	0		2.84E-35	69	59	EC	2.84E-35	
1-38	1	12490201	12490201	EC	NONE			12490201	0		7.94E-41	74	59	EC	7.94E-41	
1-41	1	12743586	12743586	EC	NONE			12743586	0		1.09E-23	78	66	EC	1.09E-23	
1-56	1	14823530	14823530	EC	NONE			14823530	0		2.12E-42	82	71	EC	2.12E-42	
1-60	1	15893107	15893107	EC	NONE			15893107	0		6.20E-10	42	41	EC	6.20E-10	
1-67	1	17404177	17404177	EC	NONE			17404177	0		1.75E-38	70	64	EC	1.75E-38	
1-90	1	33315694	33315694	EC	NONE			33315694	0		1.04E-35	43	34	EC	1.04E-35	
1-111	1	42578913	42578913	EC	NONE			42578913	0		2.73E-14	41	36	EC	2.73E-14	
1-112	1	44503513	44503513	EC	NONE			44503513	0		3.40E-10	66	54	EC	3.40E-10	
1-114	1	44868494	44868494	EC	NONE			44868494	0		1.41E-49	56	55	EC	1.41E-49	
1-128	1	53785978	53785978	EC	NONE			53785978	0		1.57E-36	69	62	EC	1.57E-36	
1-131	1	54273646	54273646	EC	NONE			54273646	0		1.62E-22	55	50	EC	1.62E-22	
1-141	1	57283701	57283701	EC	NONE			57283701	0		2.76E-38	72	64	EC	2.76E-38	
1-142	1	57332625	57332625	EC	NONE			57332625	0		8.60E-40	73	58	EC	8.60E-40	
1-148	1	57996572	57996572	EC	NONE			57996572	0		7.53E-42	81	69	EC	7.53E-42	
1-156	1	59512334	59512334	EC	NONE			59512334	0		3.07E-38	83	67	EC	3.07E-38	
1-167	1	61577140	61577140	EC	NONE			61577140	0		5.86E-73	86	74	EC	5.86E-73	
1-168	1	61610153	61610153	EC	NONE			61610153	0		2.79E-66	85	69	EC	2.79E-66	
1-169	1	61865860	61865860	EC	NONE			61865860	0		3.30E-67	90	78	EC	3.30E-67	
1-170	1	62250738	62250738	EC	NONE			62250738	0		8.18E-43	55	49	EC	8.18E-43	
1-175	1	63663651	63663651	EC	NONE			63663651	0		2.32E-16	61	55	EC	2.32E-16	
1-176	1	63672161	63672161	EC	NONE			63672161	0		4.48E-23	69	66	EC	4.48E-23	
1-211	1	75922535	75922535	EC	NONE			75922535	0		4.77E-25	56	43	EC	4.77E-25	
1-213	1	77394280	77394280	EC	NONE			77394280	0		1.56E-20	44	39	EC	1.56E-20	
1-215	1	79345845	79345845	EC	NONE			79345845	0		1.92E-35	46	38	EC	1.92E-35	
1-225	1	80910757	80910757	EC	NONE			80910757	0		1.79E-23	79	67	EC	1.79E-23	
1-230	1	82205240	82205240	EC	NONE			82205240	0		2.81E-21	69	56	EC	2.81E-21	
1-243	1	87892101	87892101	EC	NONE			87892101	0		1.45E-18	53	47	EC	1.45E-18	
1-245	1	88024773	88024773	EC	NONE			88024773	0		9.16E-42	57	53	EC	9.16E-42	
1-252	1	89173857	89173857	EC	NONE			89173857	0		9.32E-49	63	54	EC	9.32E-49	
1-257	1	90633492	90633492	EC	NONE			90633492	0		4.44E-59	69	64	EC	4.44E-59	
1-267	1	93185024	93185024	EC	NONE			93185024	0		2.13E-22	74	61	EC	2.13E-22	
1-269	1	94015267	94015267	EC	NONE			94015267	0		1.52E-47	78	63	EC	1.52E-47	
1-288	1	101638956	101638956	EC	NONE			101638956	0		2.44E-14	39	30	EC	2.44E-14	
1-292	1	102350586	102350586	EC	NONE			102350586	0		1.10E-29	60	54	EC	1.10E-29	
1-297	1	103336446	103336446	EC	NONE			103336446	0		1.55E-25	47	36	EC	1.55E-25	
1-324	1	109747656	109747656	EC	NONE			109747656	0		9.80E-17	41	37	EC	9.80E-17	
1-339	1	111926815	111926815	EC	NONE			111926815	0		4.86E-58	72	65	EC	4.86E-58	
1-342	1	112544412	112544412	EC	NONE			112544412	0		8.81E-19	51	43	EC	8.81E-19	
1-351	1	114167533	114167533	EC	NONE			114167533	0		2.28E-30	90	73	EC	2.28E-30	
1-353	1	114967276	114967276	EC	NONE			114967276	0		1.56E-29	53	41	EC	1.56E-29	
1-367	1	119932623	119932623	EC	NONE			119932623	0		2.04E-14	42	36	EC	2.04E-14	
1-397	1	129750238	129750238	EC	NONE			129750238	0		1.33E-46	76	66	EC	1.33E-46	
1-405	1	134145776	134145776	EC	NONE			134145776	0		3.34E-27	38	29	EC	3.34E-27	
1-410	1	135476495	135476495	EC	NONE			135476495	0		1.27E-27	61	52	EC	1.27E-27	
1-420	1	140911184	140911184	EC	NONE			140911184	0		1.95E-28	60	46	EC	1.95E-28	
1-425	1	142117411	142117411	EC	NONE			142117411	0		2.09E-19	57	47	EC	2.09E-19	
1-429	1	143465721	143465721	EC	NONE			143465721	0		1.14E-41	70	62	EC	1.14E-41	
1-434	1	144758126	144758126	EC	NONE			144758126	0		2.45E-23	65	59	EC	2.45E-23	
1-447	1	153925092	153925092	EC	NONE			153925092	0		4.41E-32	68	66	EC	4.41E-32	
1-451	1	154435216	154435216	EC	NONE			154435216	0		3.89E-78	90	74	EC	3.89E-78	
1-464	1	157859989	157859989	EC	NONE			157859989	0		1.97E-39	69	59	EC	1.97E-39	
1-470	1	158984712	158984712	EC	NONE			158984712	0		2.71E-25	51	43	EC	2.71E-25	
1-480	1	161210340	161210340	EC	NONE			161210340	0		1.99E-06	24	24	EC	1.99E-06	
1-489	1	163579668	163579668	EC	NONE			163579668	0		2.23E-39	67	64	EC	2.23E-39	
1-511	1	169111119	169111119	EC	NONE			169111119	0		2.43E-31	49	44	EC	2.43E-31	
1-513	1	169750552	169750552	EC	NONE			169750552	0		2.52E-23	49	48	EC	2.52E-23	
1-521	1	172321994	172321994	EC	NONE			172321994	0		4.44E-22	67	51	EC	4.44E-22	
1-528	1	174098838	174098838	EC	NONE			174098838	0		5.28E-26	82	65	EC	5.28E-26	
1-531	1	174720586	174720586	EC	NONE			174720586	0		4.01E-31	66	60	EC	4.01E-31	
1-535	1	175287528	175287528	EC	NONE			175287528	0		6.85E-56	92	82	EC	6.85E-56	
1-543	1	180914571	180914571	EC	NONE			180914571	0		6.32E-35	64	60	EC	6.32E-35	
1-564	1	192201709	192201709	EC	NONE			192201709	0		3.27E-16	68	52	EC	3.27E-16	
1-569	1	193919420	193919420	EC	NONE			193919420	0		2.08E-56	86	76	EC	2.08E-56	
1-575	1	194933398	194933398	EC	NONE			194933398	0		8.20E-32	59	57	EC	8.20E-32	
1-577	1	195298102	195298102	EC	NONE			195298102	0		4.11E-41	65	63	EC	4.11E-41	
1-590	1	197742123	197742123	EC	NONE			197742123	0		1.55E-32	59	53	EC	1.55E-32	
1-593	1	198623617	198623617	EC	NONE			198623617	0		1.11E-04	32	26	EC	1.11E-04	
1-598	1	201296101	201296101	EC	NONE			201296101	0		1.06E-21	24	22	EC	1.06E-21	
1-608	1	206693038	206693038	EC	NONE			206693038	0		1.03E-53	57	51	EC	1.03E-53	

1-611	1	207271598	207271598	EC	NONE			207271598	0		1.07E-62	92	76	EC	1.07E-62	
1-635	1	213063952	213063952	EC	NONE			213063952	0		3.32E-40	62	55	EC	3.32E-40	
1-645	1	217307150	217307150	EC	NONE			217307150	0		3.08E-39	70	59	EC	3.08E-39	
1-656	1	220458702	220458702	EC	NONE			220458702	0		8.42E-18	52	49	EC	8.42E-18	
1-675	1	227518556	227518556	EC	NONE			227518556	0		1.24E-37	75	63	EC	1.24E-37	
1-680	1	229186324	229186324	EC	NONE			229186324	0		4.54E-18	69	52	EC	4.54E-18	
1-692	1	231892404	231892404	EC	NONE			231892404	0		6.22E-27	44	37	EC	6.22E-27	
1-709	1	238829574	238829574	EC	NONE			238829574	0		4.70E-32	57	51	EC	4.70E-32	
1-728	1	251281671	251281671	EC	NONE			251281671	0		8.96E-27	68	60	EC	8.96E-27	
1-732	1	252669982	252669982	EC	NONE			252669982	0		3.84E-56	69	56	EC	3.84E-56	
1-746	1	260623031	260623031	EC	NONE			260623031	0		5.45E-33	78	73	EC	5.45E-33	
1-747	1	260673988	260673988	EC	NONE			260673988	0		4.22E-29	73	67	EC	4.22E-29	
1-814	1	298930722	298930722	EC	NONE			298930722	0		1.15E-75	91	81	EC	1.15E-75	
2-11	2	5942883	5942883	EC	NONE			5942883	0		6.05E-29	70	64	EC	6.05E-29	
2-15	2	9732728	9732728	EC	NONE			9732728	0		3.25E-40	99	88	EC	3.25E-40	
2-20	2	12866338	12866338	EC	NONE			12866338	0		1.05E-32	71	61	EC	1.05E-32	
2-22	2	13459122	13459122	EC	NONE			13459122	0		0.00E+00	69	69	EC	0.00E+00	complex event
2-30	2	16629273	16629273	EC	NONE			16629273	0		0.00E+00	50	50	EC	0.00E+00	complex event
2-38	2	19057039	19057039	EC	NONE			19057039	0		1.45E-28	47	41	EC	1.45E-28	
2-48	2	21815183	21815183	EC	NONE			21815183	0		1.14E-52	62	55	EC	1.14E-52	
2-53	2	23558503	23558503	EC	NONE			23558503	0		2.35E-28	46	46	EC	2.35E-28	
2-55	2	23778862	23778862	EC	NONE			23778862	0		2.32E-31	57	45	EC	2.32E-31	
2-68	2	26882271	26882271	EC	NONE			26882271	0		5.26E-54	92	73	EC	5.26E-54	
2-84	2	32671704	32671704	EC	NONE			32671704	0		9.26E-36	92	89	EC	9.26E-36	
2-100	2	39317994	39317994	EC	NONE			39317994	0		1.56E-34	70	66	EC	1.56E-34	
2-114	2	45653440	45653440	EC	NONE			45653440	0		8.93E-18	43	35	EC	8.93E-18	
2-115	2	45913345	45913345	EC	NONE			45913345	0		9.52E-25	53	47	EC	9.52E-25	
2-117	2	46236332	46236332	EC	NONE			46236332	0		2.60E-11	43	36	EC	2.60E-11	
2-139	2	53452258	53452258	EC	NONE			53452258	0		5.03E-07	47	41	EC	5.03E-07	
2-151	2	61754899	61754899	EC	NONE			61754899	0		6.93E-18	33	32	EC	6.93E-18	
2-156	2	65500805	65500805	EC	NONE			65500805	0		3.05E-18	39	33	EC	3.05E-18	
2-161	2	66811599	66811599	EC	NONE			66811599	0		1.84E-46	69	64	EC	1.84E-46	
2-172	2	68784591	68784591	EC	NONE			68784591	0		2.77E-48	85	72	EC	2.77E-48	
2-212	2	81782628	81782628	EC	NONE			81782628	0		4.28E-27	39	36	EC	4.28E-27	
2-223	2	85426903	85426903	EC	NONE			85426903	0		1.74E-06	50	45	EC	1.74E-06	
2-243	2	94067671	94067671	EC	NONE			94067671	0		3.33E-09	41	37	EC	3.33E-09	
2-244	2	94078172	94078172	EC	NONE			94078172	0		3.76E-07	43	36	EC	3.76E-07	
2-245	2	94089152	94089152	EC	NONE			94089152	0		5.48E-06	44	35	EC	5.48E-06	
2-246	2	94101064	94101064	EC	NONE			94101064	0		2.16E-09	45	40	EC	2.16E-09	
2-254	2	97402689	97402689	EC	NONE			97402689	0		4.81E-28	52	45	EC	4.81E-28	
2-256	2	97647674	97647674	EC	NONE			97647674	0		6.49E-26	51	47	EC	6.49E-26	
2-278	2	103949251	103949251	EC	NONE			103949251	0		3.20E-16	57	55	EC	3.20E-16	
2-279	2	112432539	112432539	EC	NONE			112432539	0		9.36E-03	10	8	EC	9.36E-03	
2-280	2	112616928	112616928	EC	NONE			112616928	0		2.21E-19	42	38	EC	2.21E-19	
2-287	2	197430950	197430950	EC	NONE			197430950	0		9.57E-24	45	43	EC	9.57E-24	
2-292	2	199565541	199565541	EC	NONE			199565541	0		1.02E-54	96	83	EC	1.02E-54	
2-311	2	211185194	211185194	EC	NONE			211185194	0		1.03E-49	89	81	EC	1.03E-49	
2-315	2	211901670	211901670	EC	NONE			211901670	0		3.04E-36	42	36	EC	3.04E-36	
2-338	2	223028093	223028093	EC	NONE			223028093	0		1.79E-31	65	58	EC	1.79E-31	
2-342	2	226627485	226627485	EC	NONE			226627485	0		4.95E-13	43	40	EC	4.95E-13	
2-351	2	230000033	230000033	EC	NONE			230000033	0		2.19E-27	66	57	EC	2.19E-27	
2-354	2	230645953	230645953	EC	NONE			230645953	0		1.83E-16	44	38	EC	1.83E-16	
2-357	2	231611414	231611414	EC	NONE			231611414	0		6.61E-18	57	54	EC	6.61E-18	
3-6	3	3642480	3642480	EC	NONE			3642480	0		6.75E-71	77	71	EC	6.75E-71	
3-18	3	18230717	18230717	EC	NONE			18230717	0		6.17E-19	56	52	EC	6.17E-19	
3-28	3	26675965	26675965	EC	NONE			26675965	0		2.56E-25	48	42	EC	2.56E-25	
3-41	3	30809421	30809421	EC	NONE			30809421	0		1.68E-16	49	43	EC	1.68E-16	
3-49	3	32568623	32568623	EC	NONE			32568623	0		4.27E-14	38	35	EC	4.27E-14	
3-52	3	33270674	33270674	EC	NONE			33270674	0		2.84E-54	78	66	EC	2.84E-54	
3-56	3	34735332	34735332	EC	NONE			34735332	0		3.43E-27	49	48	EC	3.43E-27	
3-77	3	39605886	39605886	EC	NONE			39605886	0		1.10E-07	32	31	EC	1.10E-07	
3-97	3	44439877	44439877	EC	NONE			44439877	0		4.39E-11	56	49	EC	4.39E-11	
3-99	3	44623822	44623822	EC	NONE			44623822	0		1.81E-39	52	46	EC	1.81E-39	
3-111	3	47667586	47667586	EC	NONE			47667586	0		2.58E-32	52	44	EC	2.58E-32	
3-122	3	50928401	50928401	EC	NONE			50928401	0		1.07E-33	66	62	EC	1.07E-33	
3-141	3	56695558	56695558	EC	NONE			56695558	0		3.76E-15	52	44	EC	3.76E-15	
3-176	3	73722464	73722464	EC	NONE			73722464	0		4.30E-12	22	22	EC	4.30E-12	
3-181	3	76097403	76097403	EC	NONE			76097403	0		1.03E-09	48	43	EC	1.03E-09	
3-196	3	79638000	79638000	EC	NONE			79638000	0		6.61E-33	40	33	EC	6.61E-33	
3-208	3	84719991	84719991	EC	NONE			84719991	0		7.97E-31	38	29	EC	7.97E-31	
3-216	3	87732292	87732292	EC	NONE			87732292	0		1.08E-18	47	43	EC	1.08E-18	
3-221	3	90274472	90274472	EC	NONE			90274472	0		1.84E-43	61	55	EC	1.84E-43	
3-227	3	96453118	96453118	EC	NONE			96453118	0		6.42E-47	73	62	EC	6.42E-47	
3-238	3	99520475	99520475	EC	NONE			99520475	0		2.26E-44	49	43	EC	2.26E-44	
3-243	3	101617527	101617527	EC	NONE			101617527	0		7.83E-12	38	36	EC	7.83E-12	
3-253	3	103889768	103889768	EC	NONE			103889768	0		1.83E-46	73	61	EC	1.83E-46	
3-254	3	104037207	104037207	EC	NONE			104037207	0		8.01E-40	74	66	EC	8.01E-40	
3-278	3	114776296	114776296	EC	NONE			114776296	0		5.22E-12	31	29	EC	5.22E-12	
3-282	3	116847048	116847048	EC	NONE			116847048	0		1.04E-29	54	50	EC	1.04E-29	
3-285	3	121143270	121143270	EC	NONE			121143270	0		9.46E-10	45	41	EC	9.46E-10	
3-294	3	125603671	125603671	EC	NONE			125603671	0		2.73E-30	57	51	EC	2.73E-30	
3-330	3	139655969	139655969	EC	NONE			139655969	0		1.15E-57	88	75	EC	1.15E-57	
3-338	3	141580110	141580110	EC	NONE			141580110	0		8.31E-45	63	53	EC	8.31E-45	
3-342	3	143220259	143220259	EC	NONE			143220259	0		1.30E-33	83	72	EC	1.30E-33	
3-347	3	145249189	145249189	EC	NONE			145249189	0		1.75E-10	28	28	EC	1.75E-10	
3-359	3	149997003	149997003	EC	NONE			149997003	0		4.00E-50	93	88	EC	4.00E-50	
3-361	3	150662011	150662011	EC	NONE			150662011	0		1.03E-25	73	68	EC	1.03E-25	
3-364	3	152353159	152353159	EC	NONE			152353159	0		2.61E-11	44	40	EC	2.61E-11	
3-365	3	152361500	152361500	EC	NONE			152361500	0		1.13E-12	41	39	EC	1.13E-12	
3-374	3	160997732	160997732	EC	NONE			160997732	0		4.79E-40	59	55	EC	4.79E-40	
3-384	3	163942360	163942360	EC	NONE			163942360	0		1.47E-16	51	47	EC	1.47E-16	

3-387	3	164592034	164592034	EC	NONE			164592034	0		5.55E-23	66	61	EC	5.55E-23	
3-393	3	169025081	169025081	EC	NONE			169025081	0		4.67E-48	62	61	EC	4.67E-48	
3-394	3	172101446	172101446	EC	NONE			172101446	0		1.07E-28	66	61	EC	1.07E-28	
3-397	3	173737760	173737760	EC	NONE			173737760	0		1.26E-36	87	79	EC	1.26E-36	
3-400	3	174381740	174381740	EC	NONE			174381740	0		4.41E-07	48	41	EC	4.41E-07	
3-422	3	184744385	184744385	EC	NONE			184744385	0		3.55E-22	70	64	EC	3.55E-22	
3-453	3	194891809	194891809	EC	NONE			194891809	0		4.73E-41	63	58	EC	4.73E-41	
3-481	3	205737099	205737099	EC	NONE			205737099	0		1.03E-44	53	45	EC	1.03E-44	
3-483	3	206905612	206905612	EC	NONE			206905612	0		6.35E-31	67	56	EC	6.35E-31	
3-487	3	208369108	208369108	EC	NONE			208369108	0		2.82E-36	76	62	EC	2.82E-36	
3-488	3	208381461	208381461	EC	NONE			208381461	0		1.07E-33	76	60	EC	1.07E-33	
3-494	3	210094504	210094504	EC	NONE			210094504	0		1.07E-67	98	81	EC	1.07E-67	
3-499	3	211062825	211062825	EC	NONE			211062825	0		1.44E-31	83	74	EC	1.44E-31	
3-515	3	215617434	215617434	EC	NONE			215617434	0		2.50E-52	88	71	EC	2.50E-52	
3-536	3	222157294	222157294	EC	NONE			222157294	0		3.33E-21	63	54	EC	3.33E-21	
3-543	3	226558072	226558072	EC	NONE			226558072	0		2.33E-24	62	48	EC	2.33E-24	
3-551	3	228593419	228593419	EC	NONE			228593419	0		8.79E-20	70	63	EC	8.79E-20	
3-552	3	228616984	228616984	EC	NONE			228616984	0		5.53E-18	71	62	EC	5.53E-18	
3-554	3	229146757	229146757	EC	NONE			229146757	0		2.87E-32	82	70	EC	2.87E-32	
4-7	4	6166247	6166247	EC	NONE			6166247	0		3.56E-40	83	68	EC	3.56E-40	
4-28	4	10787880	10787880	EC	NONE			10787880	0		1.59E-50	68	58	EC	1.59E-50	
4-52	4	20000842	20000842	EC	NONE			20000842	0		8.57E-28	73	64	EC	8.57E-28	
4-53	4	20026487	20026487	EC	NONE			20026487	0		1.61E-27	70	62	EC	1.61E-27	
4-55	4	23031885	23031885	EC	NONE			23031885	0		1.02E-29	44	42	EC	1.02E-29	
4-59	4	25804353	25804353	EC	NONE			25804353	0		2.53E-46	68	57	EC	2.53E-46	
4-79	4	37535703	37535703	EC	NONE			37535703	0		3.32E-21	45	40	EC	3.32E-21	
4-91	4	41976569	41976569	EC	NONE			41976569	0		2.76E-33	64	57	EC	2.76E-33	
4-92	4	42017137	42017137	EC	NONE			42017137	0		6.30E-30	62	56	EC	6.30E-30	
4-93	4	42120138	42120138	EC	NONE			42120138	0		1.21E-46	69	66	EC	1.21E-46	
4-108	4	48753177	48753177	EC	NONE			48753177	0		2.14E-17	58	50	EC	2.14E-17	
4-110	4	49001633	49001633	EC	NONE			49001633	0		3.25E-13	51	46	EC	3.25E-13	
4-114	4	50826397	50826397	EC	NONE			50826397	0		1.99E-09	20	18	EC	1.99E-09	
4-115	4	50835438	50835438	EC	NONE			50835438	0		4.47E-07	20	16	EC	4.47E-07	
4-127	4	54357516	54357516	EC	NONE			54357516	0		9.20E-20	51	45	EC	9.20E-20	
4-131	4	56711979	56711979	EC	NONE			56711979	0		5.91E-32	74	67	EC	5.91E-32	
4-136	4	60770233	60770233	EC	NONE			60770233	0		4.89E-21	45	30	EC	4.89E-21	
4-145	4	63966218	63966218	EC	NONE			63966218	0		1.17E-40	45	43	EC	1.17E-40	
4-158	4	69270213	69270213	EC	NONE			69270213	0		1.96E-25	84	73	EC	1.96E-25	
4-215	4	86564957	86564957	EC	NONE			86564957	0		9.26E-24	69	59	EC	9.26E-24	
4-216	4	86580235	86580235	EC	NONE			86580235	0		1.76E-18	69	54	EC	1.76E-18	
4-249	4	95882994	95882994	EC	NONE			95882994	0		6.45E-08	22	21	EC	6.45E-08	
4-266	4	101108654	101108654	EC	NONE			101108654	0		2.74E-26	67	47	EC	2.74E-26	
4-274	4	103313621	103313621	EC	NONE			103313621	0		3.65E-32	67	64	EC	3.65E-32	
4-280	4	107780528	107780528	EC	NONE			107780528	0		1.42E-31	68	56	EC	1.42E-31	
4-321	4	123310090	123310090	EC	NONE			123310090	0		2.46E-45	84	71	EC	2.46E-45	
4-342	4	137494123	137494123	EC	NONE			137494123	0		2.29E-28	36	33	EC	2.29E-28	
4-373	4	150214399	150214399	EC	NONE			150214399	0		5.56E-49	73	66	EC	5.56E-49	
4-378	4	153375500	153375500	EC	NONE			153375500	0		3.35E-10	47	41	EC	3.35E-10	
4-380	4	154890408	154890408	EC	NONE			154890408	0		1.27E-46	83	71	EC	1.27E-46	
4-381	4	155271093	155271093	EC	NONE			155271093	0		5.08E-10	66	58	EC	5.08E-10	
4-394	4	158222782	158222782	EC	NONE			158222782	0		2.80E-49	88	73	EC	2.80E-49	
4-399	4	159163091	159163091	EC	NONE			159163091	0		1.81E-19	40	31	EC	1.81E-19	
4-402	4	159671943	159671943	EC	NONE			159671943	0		1.95E-28	48	42	EC	1.95E-28	
4-405	4	160167966	160167966	EC	NONE			160167966	0		3.45E-57	76	59	EC	3.45E-57	
4-410	4	161202367	161202367	EC	NONE			161202367	0		4.08E-22	33	26	EC	4.08E-22	
4-427	4	164979937	164979937	EC	NONE			164979937	0		3.23E-07	36	33	EC	3.23E-07	
4-440	4	168332802	168332802	EC	NONE			168332802	0		1.22E-27	70	62	EC	1.22E-27	
4-441	4	168617100	168617100	EC	NONE			168617100	0		1.06E-50	90	79	EC	1.06E-50	
4-445	4	169527087	169527087	EC	NONE			169527087	0		5.13E-49	65	58	EC	5.13E-49	
4-451	4	172709671	172709671	EC	NONE			172709671	0		7.53E-41	75	72	EC	7.53E-41	
4-456	4	173915123	173915123	EC	NONE			173915123	0		4.08E-31	80	74	EC	4.08E-31	
4-464	4	175137770	175137770	EC	NONE			175137770	0		3.78E-54	72	66	EC	3.78E-54	
4-466	4	175764828	175764828	EC	NONE			175764828	0		6.79E-48	73	63	EC	6.79E-48	
4-478	4	183786352	183786352	EC	NONE			183786352	0		2.30E-54	79	64	EC	2.30E-54	
4-482	4	186617719	186617719	EC	NONE			186617719	0		1.68E-17	55	45	EC	1.68E-17	
4-502	4	197908499	197908499	EC	NONE			197908499	0		7.96E-44	86	73	EC	7.96E-44	
4-505	4	198344156	198344156	EC	NONE			198344156	0		1.02E-29	82	72	EC	1.02E-29	
4-520	4	202772420	202772420	EC	NONE			202772420	0		2.08E-06	40	39	EC	2.08E-06	
4-532	4	205290951	205290951	EC	NONE			205290951	0		1.14E-31	77	68	EC	1.14E-31	
4-575	4	216798370	216798370	EC	NONE			216798370	0		1.61E-48	65	58	EC	1.61E-48	
4-584	4	220671337	220671337	EC	NONE			220671337	0		3.40E-53	70	59	EC	3.40E-53	
4-598	4	223580101	223580101	EC	NONE			223580101	0		3.66E-21	63	51	EC	3.66E-21	
4-599	4	223597902	223597902	EC	NONE			223597902	0		1.24E-30	64	59	EC	1.24E-30	
4-601	4	223745425	223745425	EC	NONE			223745425	0		4.30E-24	55	49	EC	4.30E-24	
4-606	4	224916816	224916816	EC	NONE			224916816	0		4.97E-41	56	49	EC	4.97E-41	
4-607	4	225089678	225089678	EC	NONE			225089678	0		8.40E-21	63	52	EC	8.40E-21	
4-627	4	231003124	231003124	EC	NONE			231003124	0		1.36E-20	44	37	EC	1.36E-20	
4-642	4	233989209	233989209	EC	NONE			233989209	0		3.36E-54	85	73	EC	3.36E-54	
4-656	4	238116082	238116082	EC	NONE			238116082	0		1.95E-28	71	64	EC	1.95E-28	
5-4	5	1563261	1563261	EC	NONE			1563261	0		6.92E-51	77	66	EC	6.92E-51	
5-6	5	1810344	1810344	EC	NONE			1810344	0		1.54E-22	73	65	EC	1.54E-22	
5-16	5	4187055	4187055	EC	NONE			4187055	0		5.46E-48	56	50	EC	5.46E-48	
5-40	5	10178393	10178393	EC	NONE			10178393	0		7.80E-19	78	66	EC	7.80E-19	
5-63	5	30720733	30720733	EC	NONE			30720733	0		2.03E-50	57	49	EC	2.03E-50	
5-82	5	35894491	35894491	EC	NONE			35894491	0		3.65E-42	81	66	EC	3.65E-42	
5-88	5	38085996	38085996	EC	NONE			38085996	0		6.43E-21	59	52	EC	6.43E-21	
5-94	5	40040383	40040383	EC	NONE			40040383	0		3.90E-07	42	33	EC	3.90E-07	
5-103	5	43682422	43682422	EC	NONE			43682422	0		4.60E-50	87	66	EC	4.60E-50	
5-110	5	47895520	47895520	EC	NONE			47895520	0		5.49E-62	79	72	EC	5.49E-62	
5-133	5	55066762	55066762	EC	NONE			55066762	0		2.01E-36	55	46	EC	2.01E-36	
5-145	5	57128086	57128086	EC	NONE			57128086	0		8.59E-47	83	74	EC	8.59E-47	

5-153	5	60185254	60185254	EC	NONE			60185254	0		2.68E-50	87	77	EC	2.68E-50	
5-154	5	61041693	61041693	EC	NONE			61041693	0		1.53E-39	68	62	EC	1.53E-39	
5-155	5	61153740	61153740	EC	NONE			61153740	0		4.48E-26	64	51	EC	4.48E-26	
5-159	5	65393104	65393104	EC	NONE			65393104	0		4.40E-35	57	47	EC	4.40E-35	
5-161	5	65582532	65582532	EC	NONE			65582532	0		1.84E-41	69	50	EC	1.84E-41	
5-162	5	66857562	66857562	EC	NONE			66857562	0		3.54E-09	39	37	EC	3.54E-09	
5-163	5	66864092	66864092	EC	NONE			66864092	0		4.69E-11	46	44	EC	4.69E-11	
5-185	5	74045514	74045514	EC	NONE			74045514	0		1.37E-36	52	51	EC	1.37E-36	
5-195	5	76012875	76012875	EC	NONE			76012875	0		2.34E-24	58	50	EC	2.34E-24	
5-202	5	77944928	77944928	EC	NONE			77944928	0		1.30E-15	39	36	EC	1.30E-15	
5-209	5	83427349	83427349	EC	NONE			83427349	0		2.16E-46	92	83	EC	2.16E-46	
5-228	5	88316197	88316197	EC	NONE			88316197	0		1.08E-25	56	51	EC	1.08E-25	
5-237	5	91175330	91175330	EC	NONE			91175330	0		3.79E-24	86	77	EC	3.79E-24	
5-239	5	91780997	91780997	EC	NONE			91780997	0		3.43E-15	53	48	EC	3.43E-15	
5-247	5	94706665	94706665	EC	NONE			94706665	0		1.57E-27	50	46	EC	1.57E-27	
5-305	5	118785730	118785730	EC	NONE			118785730	0		1.10E-39	94	81	EC	1.10E-39	
5-308	5	120270837	120270837	EC	NONE			120270837	0		1.48E-26	57	55	EC	1.48E-26	
5-329	5	125245337	125245337	EC	NONE			125245337	0		2.90E-40	42	38	EC	2.90E-40	
5-353	5	134309845	134309845	EC	NONE			134309845	0		1.13E-32	47	36	EC	1.13E-32	
5-362	5	136279686	136279686	EC	NONE			136279686	0		8.37E-15	69	53	EC	8.37E-15	
5-391	5	145140163	145140163	EC	NONE			145140163	0		2.42E-36	71	61	EC	2.42E-36	
5-437	5	168621020	168621020	EC	NONE			168621020	0		2.08E-12	44	39	EC	2.08E-12	
5-444	5	171041132	171041132	EC	NONE			171041132	0		1.34E-24	53	49	EC	1.34E-24	
5-456	5	181642617	181642617	EC	NONE			181642617	0		3.11E-15	44	40	EC	3.11E-15	
5-460	5	184903853	184903853	EC	NONE			184903853	0		7.69E-24	66	57	EC	7.69E-24	
5-461	5	185474747	185474747	EC	NONE			185474747	0		7.03E-48	51	42	EC	7.03E-48	
5-479	5	195826960	195826960	EC	NONE			195826960	0		1.75E-23	62	53	EC	1.75E-23	
5-488	5	198752829	198752829	EC	NONE			198752829	0		1.64E-19	54	44	EC	1.64E-19	
5-490	5	200127999	200127999	EC	NONE			200127999	0		2.34E-12	45	41	EC	2.34E-12	
5-495	5	212311286	212311286	EC	NONE			212311286	0		1.19E-56	58	48	EC	1.19E-56	
5-506	5	215746063	215746063	EC	NONE			215746063	0		8.76E-19	47	42	EC	8.76E-19	
6-1	6	612219	612219	EC	NONE			612219	0		3.14E-47	51	49	EC	3.14E-47	
6-9	6	11855977	11855977	EC	NONE			11855977	0		1.07E-30	63	56	EC	1.07E-30	
6-34	6	33228488	33228488	EC	NONE			33228488	0		4.82E-18	59	54	EC	4.82E-18	
6-37	6	33769773	33769773	EC	NONE			33769773	0		5.36E-22	79	69	EC	5.36E-22	
6-48	6	37006631	37006631	EC	NONE			37006631	0		1.77E-14	41	33	EC	1.77E-14	
6-72	6	47484369	47484369	EC	NONE			47484369	0		2.48E-22	48	46	EC	2.48E-22	
6-78	6	52108872	52108872	EC	NONE			52108872	0		1.86E-24	50	44	EC	1.86E-24	
6-82	6	52992674	52992674	EC	NONE			52992674	0		4.91E-25	66	53	EC	4.91E-25	
6-90	6	54885352	54885352	EC	NONE			54885352	0		1.25E-57	71	68	EC	1.25E-57	
6-96	6	56403379	56403379	EC	NONE			56403379	0		2.46E-13	44	40	EC	2.46E-13	
6-97	6	56419040	56419040	EC	NONE			56419040	0		1.05E-16	46	44	EC	1.05E-16	
6-119	6	64775797	64775797	EC	NONE			64775797	0		6.15E-71	97	82	EC	6.15E-71	
6-123	6	65406695	65406695	EC	NONE			65406695	0		3.72E-36	64	58	EC	3.72E-36	
6-134	6	67884306	67884306	EC	NONE			67884306	0		2.19E-15	24	24	EC	2.19E-15	
6-170	6	92667234	92667234	EC	NONE			92667234	0		4.09E-18	59	51	EC	4.09E-18	
7-10	7	7236176	7236176	EC	NONE			7236176	0		2.71E-42	68	62	EC	2.71E-42	
7-17	7	8690206	8690206	EC	NONE			8690206	0		3.75E-71	78	72	EC	3.75E-71	
7-22	7	9967546	9967546	EC	NONE			9967546	0		1.61E-56	90	81	EC	1.61E-56	
7-25	7	11491548	11491548	EC	NONE			11491548	0		6.46E-37	75	64	EC	6.46E-37	
7-38	7	21521735	21521735	EC	NONE			21521735	0		9.75E-33	62	53	EC	9.75E-33	
7-41	7	23420007	23420007	EC	NONE			23420007	0		4.53E-64	75	64	EC	4.53E-64	
7-47	7	29521426	29521426	EC	NONE			29521426	0		9.44E-22	47	46	EC	9.44E-22	
7-48	7	29861751	29861751	EC	NONE			29861751	0		7.68E-45	55	49	EC	7.68E-45	
7-51	7	34356562	34356562	EC	NONE			34356562	0		3.78E-28	39	36	EC	3.78E-28	
7-54	7	35666044	35666044	EC	NONE			35666044	0		1.80E-21	28	21	EC	1.80E-21	
7-55	7	35803580	35803580	EC	NONE			35803580	0		2.03E-05	25	21	EC	2.03E-05	
7-71	7	40609165	40609165	EC	NONE			40609165	0		4.34E-43	68	63	EC	4.34E-43	
7-92	7	48060764	48060764	EC	NONE			48060764	0		4.00E-40	73	65	EC	4.00E-40	
7-106	7	52297974	52297974	EC	NONE			52297974	0		1.07E-50	54	49	EC	1.07E-50	
7-118	7	59960204	59960204	EC	NONE			59960204	0		9.57E-41	47	39	EC	9.57E-41	
7-119	7	60252161	60252161	EC	NONE			60252161	0		5.58E-06	26	24	EC	5.58E-06	
7-156	7	72265926	72265926	EC	NONE			72265926	0		8.25E-23	48	46	EC	8.25E-23	
7-168	7	76613307	76613307	EC	NONE			76613307	0		3.13E-27	62	55	EC	3.13E-27	
7-182	7	81347288	81347288	EC	NONE			81347288	0		1.79E-24	53	48	EC	1.79E-24	
7-190	7	86026528	86026528	EC	NONE			86026528	0		1.19E-31	56	55	EC	1.19E-31	
7-196	7	90241798	90241798	EC	NONE			90241798	0		1.23E-16	32	29	EC	1.23E-16	
7-217	7	98378089	98378089	EC	NONE			98378089	0		1.93E-33	84	78	EC	1.93E-33	
7-238	7	107335003	107335003	EC	NONE			107335003	0		4.35E-02	28	25	EC	4.35E-02	
7-245	7	113452257	113452257	EC	NONE			113452257	0		1.91E-16	62	59	EC	1.91E-16	
7-247	7	113651530	113651530	EC	NONE			113651530	0		3.36E-34	69	62	EC	3.36E-34	
7-255	7	117234827	117234827	EC	NONE			117234827	0		3.43E-50	89	81	EC	3.43E-50	
7-258	7	119050953	119050953	EC	NONE			119050953	0		1.38E-06	25	23	EC	1.38E-06	
7-261	7	120321929	120321929	EC	NONE			120321929	0		3.39E-20	60	46	EC	3.39E-20	
7-319	7	170886511	170886511	EC	NONE			170886511	0		2.99E-53	87	83	EC	2.99E-53	
8-6	8	5099157	5099157	EC	NONE			5099157	0		1.23E-38	71	63	EC	1.23E-38	
8-13	8	8113366	8113366	EC	NONE			8113366	0		9.74E-63	97	81	EC	9.74E-63	
8-14	8	8293620	8293620	EC	NONE			8293620	0		4.43E-41	86	58	EC	4.43E-41	
8-18	8	8960553	8960553	EC	NONE			8960553	0		6.94E-44	83	75	EC	6.94E-44	
8-29	8	11611086	11611086	EC	NONE			11611086	0		1.69E-64	97	88	EC	1.69E-64	
8-34	8	12222881	12222881	EC	NONE			12222881	0		1.36E-35	90	68	EC	1.36E-35	
8-35	8	12232550	12232550	EC	NONE			12232550	0		3.69E-50	90	79	EC	3.69E-50	
8-41	8	13953276	13953276	EC	NONE			13953276	0		7.17E-30	42	35	EC	7.17E-30	
8-54	8	18406417	18406417	EC	NONE			18406417	0		7.36E-21	60	52	EC	7.36E-21	
8-60	8	20400359	20400359	EC	NONE			20400359	0		4.52E-47	59	47	EC	4.52E-47	
8-63	8	20931846	20931846	EC	NONE			20931846	0		1.30E-12	74	48	EC	1.30E-12	
8-68	8	23675042	23675042	EC	NONE			23675042	0		1.74E-04	31	25	EC	1.74E-04	
8-69	8	23737639	23737639	EC	NONE			23737639	0		1.05E-07	34	31	EC	1.05E-07	
8-70	8	23964105	23964105	EC	NONE			23964105	0		3.19E-21	44	41	EC	3.19E-21	
8-71	8	24019203	24019203	EC	NONE			24019203	0		8.36E-27	49	36	EC	8.36E-27	
8-75	8	24889271	24889271	EC	NONE			24889271	0		5.72E-07	37	35	EC	5.72E-07	

8-81	8	26470835	26470835	EC	NONE			26470835	0		3.78E-11	12	10	EC	3.78E-11	
8-85	8	27052669	27052669	EC	NONE			27052669	0		2.48E-37	70	63	EC	2.48E-37	
8-86	8	27074372	27074372	EC	NONE			27074372	0		1.71E-28	69	56	EC	1.71E-28	
8-95	8	29755327	29755327	EC	NONE			29755327	0		5.39E-09	28	26	EC	5.39E-09	
8-100	8	33154397	33154397	EC	NONE			33154397	0		6.69E-25	62	56	EC	6.69E-25	
8-108	8	35412638	35412638	EC	NONE			35412638	0		2.12E-10	48	42	EC	2.12E-10	
8-112	8	37404916	37404916	EC	NONE			37404916	0		1.81E-16	64	51	EC	1.81E-16	
8-118	8	38656121	38656121	EC	NONE			38656121	0		1.71E-18	33	33	EC	1.71E-18	
8-152	8	53326887	53326887	EC	NONE			53326887	0		2.54E-31	53	48	EC	2.54E-31	
8-163	8	55786942	55786942	EC	NONE			55786942	0		5.77E-22	63	49	EC	5.77E-22	
8-170	8	60685862	60685862	EC	NONE			60685862	0		1.71E-42	64	58	EC	1.71E-42	
8-171	8	60696889	60696889	EC	NONE			60696889	0		2.04E-35	63	53	EC	2.04E-35	
8-178	8	63921952	63921952	EC	NONE			63921952	0		1.85E-41	68	51	EC	1.85E-41	
8-185	8	64878125	64878125	EC	NONE			64878125	0		1.76E-50	71	60	EC	1.76E-50	
8-191	8	68499391	68499391	EC	NONE			68499391	0		7.25E-48	73	65	EC	7.25E-48	
8-195	8	69596916	69596916	EC	NONE			69596916	0		6.13E-12	51	40	EC	6.13E-12	
8-201	8	71366941	71366941	EC	NONE			71366941	0		2.62E-31	76	65	EC	2.62E-31	
8-216	8	76222078	76222078	EC	NONE			76222078	0		4.30E-23	72	58	EC	4.30E-23	
8-229	8	80135049	80135049	EC	NONE			80135049	0		2.03E-34	47	40	EC	2.03E-34	
8-239	8	85893249	85893249	EC	NONE			85893249	0		2.78E-29	53	52	EC	2.78E-29	
8-248	8	92757916	92757916	EC	NONE			92757916	0		8.10E-16	61	58	EC	8.10E-16	
8-256	8	100184065	100184065	EC	NONE			100184065	0		4.26E-18	72	69	EC	4.26E-18	
8-259	8	107029258	107029258	EC	NONE			107029258	0		8.76E-20	73	63	EC	8.76E-20	
8-268	8	113108262	113108262	EC	NONE			113108262	0		1.54E-40	89	81	EC	1.54E-40	
8-269	8	113268429	113268429	EC	NONE			113268429	0		9.07E-22	64	57	EC	9.07E-22	
8-276	8	118470548	118470548	EC	NONE			118470548	0		2.88E-43	84	69	EC	2.88E-43	
8-285	8	124514260	124514260	EC	NONE			124514260	0		2.72E-13	43	40	EC	2.72E-13	
8-288	8	127705692	127705692	EC	NONE			127705692	0		1.91E-23	63	48	EC	1.91E-23	
8-299	8	129727118	129727118	EC	NONE			129727118	0		9.18E-41	62	54	EC	9.18E-41	
8-309	8	133016023	133016023	EC	NONE			133016023	0		9.52E-21	67	58	EC	9.52E-21	
8-314	8	135198945	135198945	EC	NONE			135198945	0		2.38E-23	70	60	EC	2.38E-23	
8-319	8	136240163	136240163	EC	NONE			136240163	0		2.15E-29	64	49	EC	2.15E-29	
8-320	8	136376649	136376649	EC	NONE			136376649	0		4.00E-14	61	55	EC	4.00E-14	
8-332	8	145530078	145530078	EC	NONE			145530078	0		3.67E-09	59	43	EC	3.67E-09	
8-333	8	145578189	145578189	EC	NONE			145578189	0		8.20E-18	58	52	EC	8.20E-18	
8-336	8	148517301	148517301	EC	NONE			148517301	0		2.24E-57	94	73	EC	2.24E-57	
8-357	8	159940405	159940405	EC	NONE			159940405	0		6.19E-24	94	83	EC	6.19E-24	
8-362	8	161125465	161125465	EC	NONE			161125465	0		7.86E-24	70	61	EC	7.86E-24	
8-363	8	161387714	161387714	EC	NONE			161387714	0		1.50E-23	58	51	EC	1.50E-23	
8-370	8	165604112	165604112	EC	NONE			165604112	0		1.24E-21	71	64	EC	1.24E-21	
8-371	8	170307629	170307629	EC	NONE			170307629	0		3.30E-44	84	71	EC	3.30E-44	
8-373	8	170660448	170660448	EC	NONE			170660448	0		1.57E-86	102	85	EC	1.57E-86	
8-376	8	171175275	171175275	EC	NONE			171175275	0		3.68E-22	62	55	EC	3.68E-22	
9-2	9	1663103	1663103	EC	NONE			1663103	0		2.54E-20	72	61	EC	2.54E-20	
9-4	9	4546804	4546804	EC	NONE			4546804	0		5.87E-10	41	35	EC	5.87E-10	
9-11	9	6317305	6317305	EC	NONE			6317305	0		5.36E-11	32	30	EC	5.36E-11	
9-12	9	8630361	8630361	EC	NONE			8630361	0		4.13E-21	54	49	EC	4.13E-21	
9-20	9	18876562	18876562	EC	NONE			18876562	0		5.06E-50	69	58	EC	5.06E-50	
9-21	9	18962286	18962286	EC	NONE			18962286	0		4.73E-21	68	60	EC	4.73E-21	
9-22	9	19006698	19006698	EC	NONE			19006698	0		9.14E-21	70	61	EC	9.14E-21	
9-25	9	21316708	21316708	EC	NONE			21316708	0		2.84E-56	88	67	EC	2.84E-56	
9-31	9	22090754	22090754	EC	NONE			22090754	0		1.61E-35	73	63	EC	1.61E-35	
9-66	9	40798134	40798134	EC	NONE			40798134	0		4.48E-28	56	44	EC	4.48E-28	
9-86	9	49156220	49156220	EC	NONE			49156220	0		5.41E-50	84	77	EC	5.41E-50	
9-87	9	49600781	49600781	EC	NONE			49600781	0		1.64E-18	27	21	EC	1.64E-18	
9-102	9	55928556	55928556	EC	NONE			55928556	0		1.36E-04	50	43	EC	1.36E-04	
9-106	9	57389087	57389087	EC	NONE			57389087	0		1.20E-02	17	15	EC	1.20E-02	
9-107	9	58397155	58397155	EC	NONE			58397155	0		5.40E-06	42	33	EC	5.40E-06	
9-113	9	60193974	60193974	EC	NONE			60193974	0		1.48E-22	46	45	EC	1.48E-22	
9-126	9	67016585	67016585	EC	NONE			67016585	0		1.26E-57	108	100	EC	1.26E-57	
9-132	9	68143553	68143553	EC	NONE			68143553	0		2.08E-13	37	33	EC	2.08E-13	
9-134	9	69605117	69605117	EC	NONE			69605117	0		2.49E-27	43	38	EC	2.49E-27	
9-147	9	72667866	72667866	EC	NONE			72667866	0		2.57E-51	58	52	EC	2.57E-51	
9-149	9	73001882	73001882	EC	NONE			73001882	0		8.19E-18	72	58	EC	8.19E-18	
9-157	9	76201746	76201746	EC	NONE			76201746	0		5.27E-22	52	49	EC	5.27E-22	
9-166	9	80108185	80108185	EC	NONE			80108185	0		1.45E-53	96	88	EC	1.45E-53	
9-178	9	84320248	84320248	EC	NONE			84320248	0		5.24E-16	68	61	EC	5.24E-16	
9-227	9	96498976	96498976	EC	NONE			96498976	0		1.18E-41	79	65	EC	1.18E-41	
9-231	9	97389555	97389555	EC	NONE			97389555	0		4.29E-34	55	41	EC	4.29E-34	
9-234	9	97789226	97789226	EC	NONE			97789226	0		1.09E-18	42	39	EC	1.09E-18	
9-240	9	98652096	98652096	EC	NONE			98652096	0		9.10E-25	76	57	EC	9.10E-25	
9-250	9	106397647	106397647	EC	NONE			106397647	0		1.52E-09	52	44	EC	1.52E-09	
9-254	9	112441796	112441796	EC	NONE			112441796	0		4.54E-15	52	44	EC	4.54E-15	
9-256	9	113131053	113131053	EC	NONE			113131053	0		1.06E-33	83	76	EC	1.06E-33	
9-261	9	113960676	113960676	EC	NONE			113960676	0		1.80E-21	71	67	EC	1.80E-21	
9-264	9	114590379	114590379	EC	NONE			114590379	0		2.87E-30	52	46	EC	2.87E-30	
9-267	9	116106577	116106577	EC	NONE			116106577	0		1.91E-49	69	59	EC	1.91E-49	
9-268	9	116247191	116247191	EC	NONE			116247191	0		2.43E-20	54	49	EC	2.43E-20	
9-277	9	123724973	123724973	EC	NONE			123724973	0		2.59E-24	58	49	EC	2.59E-24	
9-281	9	129780194	129780194	EC	NONE			129780194	0		3.15E-21	64	61	EC	3.15E-21	
9-310	9	138796123	138796123	EC	NONE			138796123	0		5.46E-46	55	51	EC	5.46E-46	
9-317	9	141007998	141007998	EC	NONE			141007998	0		3.11E-15	50	48	EC	3.11E-15	
9-324	9	143438030	143438030	EC	NONE			143438030	0		7.09E-36	85	74	EC	7.09E-36	
9-330	9	144464570	144464570	EC	NONE			144464570	0		1.16E-17	71	62	EC	1.16E-17	
9-347	9	150239302	150239302	EC	NONE			150239302	0		1.33E-29	77	56	EC	1.33E-29	
10-16	10	8166660	8166660	EC	NONE			8166660	0		3.94E-53	69	56	EC	3.94E-53	
10-24	10	9709262	9709262	EC	NONE			9709262	0		7.56E-20	69	62	EC	7.56E-20	
10-27	10	10335480	10335480	EC	NONE			10335480	0		5.67E-27	85	74	EC	5.67E-27	
10-30	10	13536953	13536953	EC	NONE			13536953	0		8.20E-27	47	42	EC	8.20E-27	
10-58	10	26426711	26426711	EC	NONE			26426711	0		5.19E-62	64	60	EC	5.19E-62	
10-116	10	56549665	56549665	EC	NONE			56549665	0		3.27E-32	63	59	EC	3.27E-32	

10-117	10	56685713	56685713	EC	NONE			56685713	0		4.87E-13	64	55	EC	4.87E-13	
10-118	10	56708562	56708562	EC	NONE			56708562	0		4.75E-21	68	65	EC	4.75E-21	
10-123	10	58827008	58827008	EC	NONE			58827008	0		1.55E-32	66	57	EC	1.55E-32	
10-125	10	60862199	60862199	EC	NONE			60862199	0		3.93E-19	67	57	EC	3.93E-19	
10-127	10	61076689	61076689	EC	NONE			61076689	0		7.21E-37	77	71	EC	7.21E-37	
10-128	10	61092777	61092777	EC	NONE			61092777	0		1.17E-26	75	62	EC	1.17E-26	
10-175	10	79378371	79378371	EC	NONE			79378371	0		1.61E-28	82	67	EC	1.61E-28	
10-179	10	82955169	82955169	EC	NONE			82955169	0		4.79E-29	62	48	EC	4.79E-29	
10-188	10	86152956	86152956	EC	NONE			86152956	0		3.14E-19	62	54	EC	3.14E-19	
10-189	10	86246693	86246693	EC	NONE			86246693	0		1.62E-38	66	58	EC	1.62E-38	
10-201	10	92520853	92520853	EC	NONE			92520853	0		1.63E-12	39	39	EC	1.63E-12	
10-204	10	93269374	93269374	EC	NONE			93269374	0		7.76E-30	59	48	EC	7.76E-30	
10-205	10	97516679	97516679	EC	NONE			97516679	0		1.19E-29	48	43	EC	1.19E-29	
10-209	10	98238151	98238151	EC	NONE			98238151	0		2.90E-31	50	41	EC	2.90E-31	
10-221	10	104074602	104074602	EC	NONE			104074602	0		2.23E-65	94	82	EC	2.23E-65	
10-224	10	104678850	104678850	EC	NONE			104678850	0		9.13E-36	82	62	EC	9.13E-36	
10-234	10	106947178	106947178	EC	NONE			106947178	0		1.15E-21	66	61	EC	1.15E-21	
10-249	10	109648049	109648049	EC	NONE			109648049	0		1.38E-26	67	62	EC	1.38E-26	
10-257	10	110978619	110978619	EC	NONE			110978619	0		9.00E-27	54	52	EC	9.00E-27	
10-264	10	112768345	112768345	EC	NONE			112768345	0		9.55E-44	74	63	EC	9.55E-44	
10-276	10	114810659	114810659	EC	NONE			114810659	0		3.18E-25	67	62	EC	3.18E-25	
10-277	10	114841704	114841704	EC	NONE			114841704	0		4.44E-27	68	64	EC	4.44E-27	
10-282	10	116139077	116139077	EC	NONE			116139077	0		9.77E-36	61	53	EC	9.77E-36	
10-303	10	124824915	124824915	EC	NONE			124824915	0		1.32E-36	66	63	EC	1.32E-36	
10-306	10	127178656	127178656	EC	NONE			127178656	0		1.33E-32	64	49	EC	1.33E-32	
10-315	10	136073180	136073180	EC	NONE			136073180	0		1.88E-38	74	65	EC	1.88E-38	
10-317	10	137146550	137146550	EC	NONE			137146550	0		7.87E-08	18	14	EC	7.87E-08	
1-331	1	110629368	110662204	MC	gap called	110629368	110662204		7382	7.12E-23		48	42	INS	7.12E-23	
2-273	2	102295413	102405897	MC	gap called	102295413	102405897		12587	1.97E-10		20	17	INS	1.97E-10	
3-205	3	83327834	83402352	MC	gap called	83327834	83402352		9107	5.65E-21		39	35	INS	5.65E-21	
8-43	8	14486174	14615014	MC	gap called	14486174	14615014		11880	5.54E-11		42	28	INS	5.54E-11	
1-17	1	8469067	8469067	MC	NONE			8469067	0		1.44E-30	63	1	MC	1.44E-30	
1-18	1	8474356	8474356	MC	NONE			8474356	0		1.44E-30	63	1	MC	1.44E-30	
1-27	1	9715587	9715587	MC	NONE			9715587	0		1.53E-19	76	6	MC	1.53E-19	
1-40	1	12704032	12704032	MC	NONE			12704032	0		1.48E-23	75	2	MC	1.48E-23	
1-63	1	16631014	16631014	MC	NONE			16631014	0		2.20E-11	41	5	MC	2.20E-11	
1-89	1	31448785	31448785	MC	NONE			31448785	0		1.40E-20	48	2	MC	1.40E-20	
1-99	1	36117949	36117949	MC	NONE			36117949	0		3.05E-02	53	22	MC	3.05E-02	
1-102	1	36526242	36526242	MC	NONE			36526242	0		8.05E-18	33	0	MC	8.05E-18	
1-121	1	47211052	47211052	MC	NONE			47211052	0		7.82E-10	57	10	MC	7.82E-10	
1-143	1	57389824	57389824	MC	NONE			57389824	0		4.39E-28	63	1	MC	4.39E-28	
1-185	1	67993488	67993488	MC	NONE			67993488	0		8.12E-33	85	3	MC	8.12E-33	
1-210	1	75824601	75824601	MC	NONE			75824601	0		2.20E-07	42	2	MC	2.20E-07	
1-239	1	84975673	84975673	MC	NONE			84975673	0		1.28E-36	69	2	MC	1.28E-36	
1-242	1	87816176	87816176	MC	NONE			87816176	0		1.13E-19	59	3	MC	1.13E-19	
1-251	1	89142269	89142269	MC	NONE			89142269	0		1.98E-23	58	1	MC	1.98E-23	
1-265	1	92850716	92850716	MC	NONE			92850716	0		1.92E-12	47	3	MC	1.92E-12	
1-272	1	95620897	95620897	MC	NONE			95620897	0		4.56E-13	61	6	MC	4.56E-13	
1-284	1	100721701	100721701	MC	NONE			100721701	0		3.88E-26	82	6	MC	3.88E-26	
1-285	1	100729916	100729916	MC	NONE			100729916	0		7.45E-29	82	4	MC	7.45E-29	
1-290	1	102141905	102141905	MC	NONE			102141905	0		4.08E-11	45	3	MC	4.08E-11	
1-310	1	105373990	105373990	MC	NONE			105373990	0		5.85E-14	59	2	MC	5.85E-14	
1-311	1	105426843	105426843	MC	NONE			105426843	0		1.03E-09	59	6	MC	1.03E-09	
1-338	1	111752631	111752631	MC	NONE			111752631	0		6.31E-18	62	6	MC	6.31E-18	
1-350	1	114098648	114098648	MC	NONE			114098648	0		1.15E-36	81	2	MC	1.15E-36	
1-358	1	117774690	117774690	MC	NONE			117774690	0		5.36E-31	68	0	MC	5.36E-31	
1-359	1	118151239	118151239	MC	NONE			118151239	0		3.24E-13	48	1	MC	3.24E-13	
1-366	1	119716422	119716422	MC	NONE			119716422	0		8.77E-17	47	1	MC	8.77E-17	
1-399	1	130226945	130226945	MC	NONE			130226945	0		4.23E-23	57	0	MC	4.23E-23	
1-409	1	135362390	135362390	MC	NONE			135362390	0		8.75E-29	60	2	MC	8.75E-29	
1-426	1	142399920	142399920	MC	NONE			142399920	0		6.40E-03	17	3	MC	6.40E-03	
1-435	1	144861356	144861356	MC	NONE			144861356	0		2.35E-18	55	2	MC	2.35E-18	
1-441	1	146743480	146743480	MC	NONE			146743480	0		8.16E-11	56	6	MC	8.16E-11	
1-443	1	149821062	149821062	MC	NONE			149821062	0		4.99E-09	28	2	MC	4.99E-09	
1-452	1	154464779	154464779	MC	NONE			154464779	0		1.03E-47	89	2	MC	1.03E-47	
1-459	1	156698434	156698434	MC	NONE			156698434	0		1.73E-29	62	1	MC	1.73E-29	
1-466	1	158268305	158268305	MC	NONE			158268305	0		4.16E-21	49	1	MC	4.16E-21	
1-479	1	161090720	161090720	MC	NONE			161090720	0		2.08E-02	14	2	MC	2.08E-02	
1-500	1	166128124	166128124	MC	NONE			166128124	0		8.90E-35	68	3	MC	8.90E-35	
1-522	1	172423934	172423934	MC	NONE			172423934	0		4.13E-32	68	1	MC	4.13E-32	
1-532	1	174800188	174800188	MC	NONE			174800188	0		8.60E-19	64	4	MC	8.60E-19	
1-533	1	174895205	174895205	MC	NONE			174895205	0		6.29E-30	76	3	MC	6.29E-30	
1-556	1	186089953	186089953	MC	NONE			186089953	0		6.90E-12	26	0	MC	6.90E-12	
1-566	1	192481898	192481898	MC	NONE			192481898	0		3.01E-22	77	7	MC	3.01E-22	
1-588	1	197373647	197373647	MC	NONE			197373647	0		4.32E-22	69	4	MC	4.32E-22	
1-597	1	199727777	199727777	MC	NONE			199727777	0		1.58E-16	62	4	MC	1.58E-16	
1-618	1	209396907	209396907	MC	NONE			209396907	0		2.12E-06	30	0	MC	2.12E-06	
1-644	1	217233105	217233105	MC	NONE			217233105	0		1.93E-18	66	4	MC	1.93E-18	
1-651	1	219565045	219565045	MC	NONE			219565045	0		2.49E-19	57	4	MC	2.49E-19	
1-655	1	220276222	220276222	MC	NONE			220276222	0		5.46E-24	54	2	MC	5.46E-24	
1-657	1	220631106	220631106	MC	NONE			220631106	0		3.95E-11	37	2	MC	3.95E-11	
1-685	1	230651586	230651586	MC	NONE			230651586	0		5.06E-36	73	2	MC	5.06E-36	
1-687	1	230965215	230965215	MC	NONE			230965215	0		2.65E-40	89	4	MC	2.65E-40	
1-716	1	245707727	245707727	MC	NONE			245707727	0		3.16E-38	80	4	MC	3.16E-38	
1-743	1	255455688	255455688	MC	NONE			255455688	0		7.41E-14	27	1	MC	7.41E-14	
1-744	1	257826613	257826613	MC	NONE			257826613	0		2.40E-27	64	2	MC	2.40E-27	
1-795	1	286601807	286601807	MC	NONE			286601807	0		6.05E-04	23	4	MC	6.05E-04	
2-4	2	4768557	4768557	MC	NONE			4768557	0		3.09E-20	60	2	MC	3.09E-20	
2-80	2	31830182	31830182	MC	NONE			31830182	0		1.32E-11	50	4	MC	1.32E-11	
2-88	2	33246665	33246665	MC	NONE			33246665	0		1.42E-28	48	0	MC	1.42E-28	
2-95	2	36999296	36999296	MC	NONE			36999296	0		3.45E-21	60	2	MC	3.45E-21	

2-147	2	59895892	59895892	MC	NONE			59895892	0		6.37E-12	35	1	MC	6.37E-12	
2-155	2	62735951	62735951	MC	NONE			62735951	0		5.48E-43	77	1	MC	5.48E-43	
2-160	2	66610859	66610859	MC	NONE			66610859	0		3.98E-23	55	3	MC	3.98E-23	
2-184	2	71816278	71816278	MC	NONE			71816278	0		3.75E-08	25	0	MC	3.75E-08	
2-199	2	75968566	75968566	MC	NONE			75968566	0		3.84E-11	39	2	MC	3.84E-11	
2-202	2	76618903	76618903	MC	NONE			76618903	0		6.27E-09	39	3	MC	6.27E-09	
2-211	2	81612721	81612721	MC	NONE			81612721	0		9.23E-05	26	2	MC	9.23E-05	
2-303	2	208123393	208123393	MC	NONE			208123393	0		6.51E-17	66	5	MC	6.51E-17	
2-309	2	209477480	209477480	MC	NONE			209477480	0		5.03E-30	72	5	MC	5.03E-30	
2-337	2	221477525	221477525	MC	NONE			221477525	0		2.49E-35	59	0	MC	2.49E-35	
2-346	2	228360330	228360330	MC	NONE			228360330	0		2.92E-19	49	5	MC	2.92E-19	
3-2	3	2156033	2156033	MC	NONE			2156033	0		4.31E-23	77	7	MC	4.31E-23	
3-13	3	12857738	12857738	MC	NONE			12857738	0		1.45E-12	58	5	MC	1.45E-12	
3-20	3	18418199	18418199	MC	NONE			18418199	0		2.33E-12	46	4	MC	2.33E-12	
3-42	3	30912094	30912094	MC	NONE			30912094	0		9.83E-16	34	0	MC	9.83E-16	
3-54	3	33900579	33900579	MC	NONE			33900579	0		8.49E-06	25	1	MC	8.49E-06	
3-62	3	36358375	36358375	MC	NONE			36358375	0		3.25E-38	79	4	MC	3.25E-38	
3-68	3	37818708	37818708	MC	NONE			37818708	0		2.84E-18	66	3	MC	2.84E-18	
3-103	3	46258737	46258737	MC	NONE			46258737	0		3.69E-25	72	8	MC	3.69E-25	
3-118	3	50290863	50290863	MC	NONE			50290863	0		1.63E-07	37	4	MC	1.63E-07	
3-179	3	74994349	74994349	MC	NONE			74994349	0		1.59E-03	22	4	MC	1.59E-03	
3-184	3	77130533	77130533	MC	NONE			77130533	0		6.53E-09	21	1	MC	6.53E-09	
3-223	3	90714635	90714635	MC	NONE			90714635	0		8.34E-04	25	2	MC	8.34E-04	
3-225	3	91708339	91708339	MC	NONE			91708339	0		9.96E-04	35	5	MC	9.96E-04	
3-236	3	99325467	99325467	MC	NONE			99325467	0		4.23E-04	20	1	MC	4.23E-04	
3-276	3	113391835	113391835	MC	NONE			113391835	0		7.25E-08	24	1	MC	7.25E-08	
3-281	3	116806154	116806154	MC	NONE			116806154	0		1.92E-10	41	1	MC	1.92E-10	
3-292	3	125276894	125276894	MC	NONE			125276894	0		2.58E-15	50	2	MC	2.58E-15	
3-296	3	126401266	126401266	MC	NONE			126401266	0		2.15E-17	64	8	MC	2.15E-17	
3-306	3	131956850	131956850	MC	NONE			131956850	0		5.01E-16	70	6	MC	5.01E-16	
3-333	3	140426950	140426950	MC	NONE			140426950	0		8.59E-21	39	0	MC	8.59E-21	
3-336	3	141292978	141292978	MC	NONE			141292978	0		2.20E-23	56	3	MC	2.20E-23	
3-392	3	168853954	168853954	MC	NONE			168853954	0		1.27E-30	58	1	MC	1.27E-30	
3-398	3	173796887	173796887	MC	NONE			173796887	0		7.04E-22	75	5	MC	7.04E-22	
3-411	3	181842433	181842433	MC	NONE			181842433	0		1.91E-18	63	5	MC	1.91E-18	
3-425	3	185045756	185045756	MC	NONE			185045756	0		1.93E-22	68	2	MC	1.93E-22	
3-437	3	187416941	187416941	MC	NONE			187416941	0		4.95E-25	79	5	MC	4.95E-25	
3-450	3	194195302	194195302	MC	NONE			194195302	0		1.54E-09	37	3	MC	1.54E-09	
3-455	3	196144505	196144505	MC	NONE			196144505	0		3.52E-09	38	3	MC	3.52E-09	
3-458	3	199360808	199360808	MC	NONE			199360808	0		1.61E-30	63	2	MC	1.61E-30	
3-461	3	200962455	200962455	MC	NONE			200962455	0		3.31E-28	68	5	MC	3.31E-28	
3-477	3	204718882	204718882	MC	NONE			204718882	0		4.69E-26	58	2	MC	4.69E-26	
3-506	3	213969732	213969732	MC	NONE			213969732	0		2.64E-36	82	4	MC	2.64E-36	
3-509	3	214707938	214707938	MC	NONE			214707938	0		1.49E-26	72	5	MC	1.49E-26	
3-524	3	218481674	218481674	MC	NONE			218481674	0		3.76E-02	12	1	MC	3.76E-02	
4-5	4	5708728	5708728	MC	NONE			5708728	0		2.76E-26	60	0	MC	2.76E-26	
4-11	4	6634841	6634841	MC	NONE			6634841	0		9.16E-02	82	47	MC	9.16E-02	
4-41	4	16816048	16816048	MC	NONE			16816048	0		2.44E-03	31	5	MC	2.44E-03	
4-43	4	18220340	18220340	MC	NONE			18220340	0		3.23E-31	73	1	MC	3.23E-31	
4-48	4	18717367	18717367	MC	NONE			18717367	0		4.70E-16	51	4	MC	4.70E-16	
4-60	4	25977701	25977701	MC	NONE			25977701	0		8.48E-19	66	5	MC	8.48E-19	
4-70	4	32183069	32183069	MC	NONE			32183069	0		1.21E-16	55	6	MC	1.21E-16	
4-87	4	39875818	39875818	MC	NONE			39875818	0		1.87E-27	50	1	MC	1.87E-27	
4-123	4	53223372	53223372	MC	NONE			53223372	0		1.99E-10	42	5	MC	1.99E-10	
4-130	4	55425110	55425110	MC	NONE			55425110	0		5.68E-20	42	2	MC	5.68E-20	
4-144	4	63732250	63732250	MC	NONE			63732250	0		4.30E-18	37	0	MC	4.30E-18	
4-149	4	66142051	66142051	MC	NONE			66142051	0		1.37E-03	22	1	MC	1.37E-03	
4-159	4	69334626	69334626	MC	NONE			69334626	0		2.09E-42	93	3	MC	2.09E-42	
4-187	4	79940335	79940335	MC	NONE			79940335	0		6.06E-11	53	6	MC	6.06E-11	
4-196	4	81838376	81838376	MC	NONE			81838376	0		1.08E-38	74	2	MC	1.08E-38	
4-211	4	85712635	85712635	MC	NONE			85712635	0		2.30E-10	47	2	MC	2.30E-10	
4-241	4	93322530	93322530	MC	NONE			93322530	0		6.37E-08	42	3	MC	6.37E-08	
4-265	4	101079127	101079127	MC	NONE			101079127	0		5.03E-32	67	3	MC	5.03E-32	
4-309	4	120212381	120212381	MC	NONE			120212381	0		1.08E-29	61	1	MC	1.08E-29	
4-322	4	123625090	123625090	MC	NONE			123625090	0		2.75E-09	33	0	MC	2.75E-09	
4-326	4	129451144	129451144	MC	NONE			129451144	0		4.95E-07	35	4	MC	4.95E-07	
4-341	4	137349588	137349588	MC	NONE			137349588	0		2.26E-22	48	0	MC	2.26E-22	
4-367	4	146014515	146014515	MC	NONE			146014515	0		2.32E-39	95	4	MC	2.32E-39	
4-376	4	152953164	152953164	MC	NONE			152953164	0		1.37E-20	58	1	MC	1.37E-20	
4-379	4	153467855	153467855	MC	NONE			153467855	0		5.74E-19	47	3	MC	5.74E-19	
4-391	4	157639832	157639832	MC	NONE			157639832	0		6.84E-25	62	2	MC	6.84E-25	
4-401	4	159460693	159460693	MC	NONE			159460693	0		3.02E-02	17	4	MC	3.02E-02	
4-432	4	166505562	166505562	MC	NONE			166505562	0		2.24E-15	40	1	MC	2.24E-15	
4-433	4	166624122	166624122	MC	NONE			166624122	0		0.00E+00	24	24	MC	0.00E+00	complex event
4-468	4	177343455	177343455	MC	NONE			177343455	0		8.20E-11	45	5	MC	8.20E-11	
4-469	4	181473920	181473920	MC	NONE			181473920	0		1.03E-06	36	3	MC	1.03E-06	
4-475	4	183302960	183302960	MC	NONE			183302960	0		2.35E-07	30	1	MC	2.35E-07	
4-481	4	186097900	186097900	MC	NONE			186097900	0		1.79E-49	108	2	MC	1.79E-49	
4-518	4	202025909	202025909	MC	NONE			202025909	0		1.36E-18	66	4	MC	1.36E-18	
4-558	4	212338556	212338556	MC	NONE			212338556	0		3.21E-06	25	1	MC	3.21E-06	
4-560	4	212950323	212950323	MC	NONE			212950323	0		2.00E-17	63	7	MC	2.00E-17	
4-600	4	223730332	223730332	MC	NONE			223730332	0		4.75E-26	52	1	MC	4.75E-26	
4-663	4	242834226	242834226	MC	NONE			242834226	0		3.68E-12	36	1	MC	3.68E-12	
5-22	5	5098781	5098781	MC	NONE			5098781	0		1.26E-16	69	5	MC	1.26E-16	
5-76	5	34328789	34328789	MC	NONE			34328789	0		4.66E-23	65	2	MC	4.66E-23	
5-105	5	44116379	44116379	MC	NONE			44116379	0		5.36E-08	21	2	MC	5.36E-08	
5-126	5	52377267	52377267	MC	NONE			52377267	0		1.27E-36	82	3	MC	1.27E-36	
5-136	5	55732001	55732001	MC	NONE			55732001	0		1.33E-23	57	2	MC	1.33E-23	
5-144	5	57050933	57050933	MC	NONE			57050933	0		4.35E-28	81	7	MC	4.35E-28	
5-160	5	65506917	65506917	MC	NONE			65506917	0		8.11E-08	53	6	MC	8.11E-08	
5-164	5	66942514	66942514	MC	NONE			66942514	0		1.69E-26	56	1	MC	1.69E-26	

5-178	5	72421917	72421917	MC	NONE			72421917	0		1.15E-16	53	3	MC	1.15E-16
5-238	5	91588086	91588086	MC	NONE			91588086	0		1.38E-12	51	6	MC	1.38E-12
5-253	5	96997412	96997412	MC	NONE			96997412	0		4.03E-37	65	0	MC	4.03E-37
5-288	5	110367490	110367490	MC	NONE			110367490	0		2.83E-24	80	9	MC	2.83E-24
5-315	5	121817277	121817277	MC	NONE			121817277	0		6.06E-21	47	1	MC	6.06E-21
5-354	5	134421650	134421650	MC	NONE			134421650	0		5.28E-20	47	2	MC	5.28E-20
5-357	5	135045110	135045110	MC	NONE			135045110	0		9.50E-23	69	5	MC	9.50E-23
5-422	5	159716803	159716803	MC	NONE			159716803	0		3.87E-13	38	1	MC	3.87E-13
5-428	5	162644504	162644504	MC	NONE			162644504	0		2.42E-23	69	3	MC	2.42E-23
6-2	6	2745119	2745119	MC	NONE			2745119	0		2.31E-13	53	1	MC	2.31E-13
6-11	6	12058770	12058770	MC	NONE			12058770	0		9.71E-16	61	7	MC	9.71E-16
6-13	6	18488728	18488728	MC	NONE			18488728	0		5.98E-16	58	7	MC	5.98E-16
6-23	6	20808967	20808967	MC	NONE			20808967	0		7.11E-18	62	6	MC	7.11E-18
6-25	6	29535970	29535970	MC	NONE			29535970	0		1.41E-06	19	0	MC	1.41E-06
6-91	6	54941425	54941425	MC	NONE			54941425	0		6.00E-17	56	1	MC	6.00E-17
6-118	6	64676223	64676223	MC	NONE			64676223	0		4.66E-26	89	7	MC	4.66E-26
6-138	6	70373257	70373257	MC	NONE			70373257	0		2.58E-09	27	3	MC	2.58E-09
6-141	6	73894689	73894689	MC	NONE			73894689	0		1.77E-17	44	2	MC	1.77E-17
7-26	7	14095976	14095976	MC	NONE			14095976	0		1.14E-12	43	3	MC	1.14E-12
7-37	7	21314619	21314619	MC	NONE			21314619	0		1.99E-07	30	0	MC	1.99E-07
7-90	7	47790796	47790796	MC	NONE			47790796	0		2.89E-11	57	3	MC	2.89E-11
7-104	7	50751060	50751060	MC	NONE			50751060	0		8.45E-16	47	0	MC	8.45E-16
7-112	7	56617916	56617916	MC	NONE			56617916	0		2.79E-06	34	2	MC	2.79E-06
7-129	7	63134934	63134934	MC	NONE			63134934	0		2.27E-08	36	2	MC	2.27E-08
7-191	7	89183690	89183690	MC	NONE			89183690	0		3.49E-09	36	2	MC	3.49E-09
7-205	7	92818873	92818873	MC	NONE			92818873	0		8.59E-17	54	3	MC	8.59E-17
7-213	7	95238858	95238858	MC	NONE			95238858	0		1.12E-19	52	4	MC	1.12E-19
7-220	7	99086268	99086268	MC	NONE			99086268	0		1.64E-31	79	6	MC	1.64E-31
7-296	7	144805768	144805768	MC	NONE			144805768	0		9.65E-09	35	1	MC	9.65E-09
8-56	8	18748209	18748209	MC	NONE			18748209	0		1.03E-29	63	2	MC	1.03E-29
8-59	8	19922516	19922516	MC	NONE			19922516	0		2.20E-02	28	5	MC	2.20E-02
8-84	8	27020634	27020634	MC	NONE			27020634	0		5.33E-26	66	4	MC	5.33E-26
8-127	8	40658230	40658230	MC	NONE			40658230	0		1.21E-21	51	4	MC	1.21E-21
8-144	8	48329382	48329382	MC	NONE			48329382	0		7.15E-18	40	2	MC	7.15E-18
8-158	8	54851439	54851439	MC	NONE			54851439	0		1.20E-18	49	3	MC	1.20E-18
8-169	8	60645842	60645842	MC	NONE			60645842	0		4.26E-28	59	2	MC	4.26E-28
8-172	8	60748013	60748013	MC	NONE			60748013	0		2.00E-24	55	3	MC	2.00E-24
8-179	8	63935323	63935323	MC	NONE			63935323	0		0.00E+00	63	63	MC	0.00E+00
8-277	8	118599353	118599353	MC	NONE			118599353	0		2.56E-16	64	8	MC	2.56E-16
8-280	8	119329042	119329042	MC	NONE			119329042	0		1.76E-07	29	2	MC	1.76E-07
8-293	8	128905035	128905035	MC	NONE			128905035	0		1.15E-25	74	5	MC	1.15E-25
8-296	8	129198075	129198075	MC	NONE			129198075	0		4.14E-25	70	5	MC	4.14E-25
8-306	8	131129623	131129623	MC	NONE			131129623	0		6.79E-13	64	10	MC	6.79E-13
8-311	8	134586758	134586758	MC	NONE			134586758	0		2.83E-04	29	6	MC	2.83E-04
8-322	8	136508630	136508630	MC	NONE			136508630	0		8.43E-36	67	2	MC	8.43E-36
8-334	8	147640004	147640004	MC	NONE			147640004	0		1.22E-17	55	5	MC	1.22E-17
8-346	8	150109853	150109853	MC	NONE			150109853	0		2.49E-41	70	0	MC	2.49E-41
8-369	8	164150783	164150783	MC	NONE			164150783	0		3.71E-04	33	6	MC	3.71E-04
9-41	9	29802297	29802297	MC	NONE			29802297	0		2.92E-04	22	4	MC	2.92E-04
9-63	9	40164254	40164254	MC	NONE			40164254	0		1.94E-21	62	3	MC	1.94E-21
9-75	9	42885272	42885272	MC	NONE			42885272	0		7.62E-20	54	4	MC	7.62E-20
9-98	9	54953413	54953413	MC	NONE			54953413	0		8.22E-08	39	3	MC	8.22E-08
9-104	9	56673331	56673331	MC	NONE			56673331	0		3.27E-13	47	2	MC	3.27E-13
9-110	9	59063271	59063271	MC	NONE			59063271	0		1.17E-08	36	1	MC	1.17E-08
9-118	9	64162741	64162741	MC	NONE			64162741	0		2.89E-34	59	1	MC	2.89E-34
9-158	9	76368314	76368314	MC	NONE			76368314	0		1.68E-31	63	1	MC	1.68E-31
9-161	9	76759941	76759941	MC	NONE			76759941	0		2.01E-55	94	0	MC	2.01E-55
9-185	9	86422605	86422605	MC	NONE			86422605	0		6.07E-36	79	3	MC	6.07E-36
9-200	9	90180655	90180655	MC	NONE			90180655	0		3.04E-35	74	2	MC	3.04E-35
9-203	9	91003389	91003389	MC	NONE			91003389	0		1.35E-05	35	2	MC	1.35E-05
9-233	9	97770681	97770681	MC	NONE			97770681	0		7.66E-12	37	2	MC	7.66E-12
9-239	9	98609227	98609227	MC	NONE			98609227	0		1.15E-37	70	0	MC	1.15E-37
9-274	9	120392089	120392089	MC	NONE			120392089	0		3.19E-21	52	2	MC	3.19E-21
9-294	9	135192122	135192122	MC	NONE			135192122	0		3.70E-25	71	5	MC	3.70E-25
9-303	9	137165465	137165465	MC	NONE			137165465	0		1.31E-28	66	3	MC	1.31E-28
9-328	9	144156956	144156956	MC	NONE			144156956	0		2.47E-21	66	2	MC	2.47E-21
10-2	10	436495	436495	MC	NONE			436495	0		2.52E-52	128	6	MC	2.52E-52
10-77	10	36323277	36323277	MC	NONE			36323277	0		4.25E-06	20	1	MC	4.25E-06
10-106	10	53248122	53248122	MC	NONE			53248122	0		8.84E-10	38	1	MC	8.84E-10
10-107	10	53441045	53441045	MC	NONE			53441045	0		5.26E-04	27	5	MC	5.26E-04
10-109	10	53942336	53942336	MC	NONE			53942336	0		4.24E-16	58	2	MC	4.24E-16
10-141	10	68747720	68747720	MC	NONE			68747720	0		5.58E-34	70	2	MC	5.58E-34
10-164	10	75503268	75503268	MC	NONE			75503268	0		2.17E-32	66	2	MC	2.17E-32
10-171	10	77116117	77116117	MC	NONE			77116117	0		2.69E-08	33	2	MC	2.69E-08
10-177	10	82267770	82267770	MC	NONE			82267770	0		1.84E-21	46	1	MC	1.84E-21
10-180	10	82986141	82986141	MC	NONE			82986141	0		2.72E-38	67	0	MC	2.72E-38
10-184	10	83613667	83613667	MC	NONE			83613667	0		2.49E-26	66	3	MC	2.49E-26
10-187	10	86085655	86085655	MC	NONE			86085655	0		1.73E-23	50	2	MC	1.73E-23
10-196	10	90822941	90822941	MC	NONE			90822941	0		2.23E-19	48	0	MC	2.23E-19
10-212	10	99999350	99999350	MC	NONE			99999350	0		8.34E-13	40	1	MC	8.34E-13
10-228	10	105417348	105417348	MC	NONE			105417348	0		3.77E-17	78	9	MC	3.77E-17
10-236	10	107285944	107285944	MC	NONE			107285944	0		1.21E-39	67	0	MC	1.21E-39
10-254	10	110616713	110616713	MC	NONE			110616713	0		7.25E-20	55	3	MC	7.25E-20
10-271	10	113788172	113788172	MC	NONE			113788172	0		2.27E-32	60	2	MC	2.27E-32
10-286	10	116820853	116820853	MC	NONE			116820853	0		3.02E-13	49	3	MC	3.02E-13
10-287	10	117048370	117048370	MC	NONE			117048370	0		4.91E-22	51	1	MC	4.91E-22
10-294	10	118027267	118027267	MC	NONE			118027267	0		1.15E-05	41	5	MC	1.15E-05
10-298	10	119336303	119336303	MC	NONE			119336303	0		3.07E-18	47	2	MC	3.07E-18
10-307	10	127302197	127302197	MC	NONE			127302197	0		2.42E-27	64	4	MC	2.42E-27
10-316	10	137084107	137084107	MC	NONE			137084107	0		3.84E-10	17	0	MC	3.84E-10
1-7	1	2870915	2900564	NONE	Extra sequence included	2870915	2900564		-5304	7.12E-23		90	63	Extra sequ	7.12E-23

1-8	1	5093981	5223564	NONE	Extra sequence included	5093981	5223564	-35231	7.12E-23	39	33	DEL	7.12E-23
1-11	1	5649835	5697275	NONE	Extra sequence included	5649835	5697275	-9626	7.12E-23	72	55	DEL	7.12E-23
1-15	1	7962165	7994331	NONE	Extra sequence included	7962165	7994331	-6937	7.12E-23	78	62	DEL	7.12E-23
1-20	1	8670170	8764224	NONE	Extra sequence included	8670170	8764224	-14234	7.12E-23	50	41	DEL	7.12E-23
1-22	1	9036636	9173244	NONE	Extra sequence included	9036636	9173244	-87932	1.01E-22	52	44	DEL	1.01E-22
1-25	1	9591313	9611714	NONE	Extra sequence included	9591313	9611714	-20401	0.00E+00	80	0	DEL	0.00E+00
1-26	1	9642757	9660754	NONE	Extra sequence included	9642757	9660754	-17997	0.00E+00	77	0	DEL	0.00E+00
1-35	1	11907037	11927693	NONE	Extra sequence included	11907037	11927693	-6075	7.12E-23	80	54	DEL	7.12E-23
1-46	1	13398838	13468970	NONE	Extra sequence included	13398838	13468970	-9808	7.12E-23	67	52	DEL	7.12E-23
1-49	1	13717079	13808138	NONE	Extra sequence included	13717079	13808138	-17755	7.12E-23	58	44	DEL	7.12E-23
1-50	1	14034506	14095925	NONE	Extra sequence included	14034506	14095925	-22880	1.01E-22	54	42	DEL	1.01E-22
1-51	1	14102372	14164884	NONE	Extra sequence included	14102372	14164884	-8505	7.12E-23	55	48	DEL	7.12E-23
1-52	1	14208709	14262945	NONE	Extra sequence included	14208709	14262945	-10505	7.12E-23	56	52	DEL	7.12E-23
1-57	1	14910821	14987214	NONE	Extra sequence included	14910821	14987214	-15449	7.12E-23	78	63	DEL	7.12E-23
1-59	1	15707773	15742972	NONE	Extra sequence included	15707773	15742972	-5578	7.12E-23	56	42	DEL	7.12E-23
1-64	1	16648258	16720228	NONE	Extra sequence included	16648258	16720228	-12289	7.12E-23	31	24	DEL	7.12E-23
1-68	1	17469084	17532636	NONE	Extra sequence included	17469084	17532636	-11908	1.01E-22	61	51	DEL	1.01E-22
1-70	1	17919754	17964088	NONE	Extra sequence included	17919754	17964088	-8552	7.12E-23	70	47	DEL	7.12E-23
1-83	1	28950659	28970799	NONE	Extra sequence included	28950659	28970799	-20140	0.00E+00	74	0	DEL	0.00E+00
1-87	1	31122848	31201889	NONE	Extra sequence included	31122848	31201889	-12773	7.12E-23	43	32	DEL	7.12E-23
1-91	1	33564991	33627387	NONE	Extra sequence included	33564991	33627387	-9204	7.12E-23	72	62	DEL	7.12E-23
1-100	1	36193013	36226149	NONE	Extra sequence included	36193013	36226149	-33136	0.00E+00	56	0	DEL	0.00E+00
1-106	1	37237219	37267764	NONE	Extra sequence included	37237219	37267764	-7154	7.12E-23	70	53	DEL	7.12E-23
1-113	1	44803842	44855666	NONE	Extra sequence included	44803842	44855666	-5243	3.28E-15	53	36	DEL	3.28E-15
1-115	1	44883390	44949913	NONE	Extra sequence included	44883390	44949913	-11532	7.12E-23	39	33	DEL	7.12E-23
1-118	1	46407270	46535110	NONE	Extra sequence included	46407270	46535110	-18513	7.12E-23	24	24	DEL	7.12E-23
1-120	1	47077302	47095125	NONE	Extra sequence included	47077302	47095125	-17823	0.00E+00	59	0	DEL	0.00E+00
1-123	1	50965166	51047441	NONE	Extra sequence included	50965166	51047441	-8461	2.75E-16	48	35	DEL	2.75E-16
1-129	1	53886800	53912990	NONE	Extra sequence included	53886800	53912990	-5665	7.12E-23	81	57	DEL	7.12E-23
1-130	1	54003687	54053237	NONE	Extra sequence included	54003687	54053237	-7429	7.12E-23	75	60	DEL	7.12E-23
1-135	1	55566550	55648111	NONE	Extra sequence included	55566550	55648111	-10553	7.12E-23	47	38	DEL	7.12E-23
1-145	1	57558951	57635411	NONE	Extra sequence included	57558951	57635411	-34460	1.01E-22	45	30	DEL	1.01E-22
1-149	1	58054322	58106541	NONE	Extra sequence included	58054322	58106541	-5970	7.12E-23	81	54	DEL	7.12E-23
1-150	1	58289840	58320669	NONE	Extra sequence included	58289840	58320669	-30829	0.00E+00	68	0	DEL	0.00E+00
1-152	1	58414699	58444879	NONE	Extra sequence included	58414699	58444879	-8928	7.12E-23	64	58	DEL	7.12E-23
1-159	1	59744180	59771453	NONE	Extra sequence included	59744180	59771453	-5090	7.12E-23	73	55	DEL	7.12E-23
1-166	1	61447972	61496726	NONE	Extra sequence included	61447972	61496726	-13814	7.12E-23	64	47	DEL	7.12E-23
1-171	1	62406119	62454313	NONE	Extra sequence included	62406119	62454313	-13454	7.12E-23	54	47	DEL	7.12E-23
1-177	1	63769045	63867831	NONE	Extra sequence included	63769045	63867831	-36352	7.12E-23	61	53	DEL	7.12E-23
1-179	1	63999948	64051089	NONE	Extra sequence included	63999948	64051089	-51141	0.00E+00	67	0	DEL	0.00E+00
1-181	1	64859590	64892897	NONE	Extra sequence included	64859590	64892897	-5897	7.12E-23	66	62	DEL	7.12E-23
1-186	1	68091250	68129713	NONE	Extra sequence included	68091250	68129713	-13831	7.12E-23	87	61	DEL	7.12E-23
1-192	1	71641767	71710605	NONE	Extra sequence included	71641767	71710605	-8136	8.84E-18	40	30	DEL	8.84E-18
1-201	1	74325423	74347342	NONE	Extra sequence included	74325423	74347342	-5531	7.12E-23	98	84	DEL	7.12E-23
1-214	1	77548234	77597704	NONE	Extra sequence included	77548234	77597704	-16933	7.12E-23	52	33	DEL	7.12E-23
1-218	1	79617814	79633967	NONE	Extra sequence included	79617814	79633967	-16153	0.00E+00	89	0	DEL	0.00E+00
1-247	1	88255131	88276336	NONE	Extra sequence included	88255131	88276336	-21205	0.00E+00	47	0	DEL	0.00E+00
1-268	1	93822092	93942011	NONE	Extra sequence included	93822092	93942011	-119919	0.00E+00	74	0	DEL	0.00E+00
1-274	1	95912909	96002277	NONE	Extra sequence included	95912909	96002277	-20270	1.01E-22	47	29	DEL	1.01E-22
1-275	1	96103781	96155426	NONE	Extra sequence included	96103781	96155426	-5222	1.45E-15	42	37	DEL	1.45E-15
1-295	1	102848919	102893366	NONE	Extra sequence included	102848919	102893366	-9084	7.12E-23	15	15	DEL	7.12E-23
1-298	1	103394813	103455669	NONE	Extra sequence included	103394813	103455669	-45976	1.01E-22	57	48	DEL	1.01E-22
1-302	1	104210212	104260119	NONE	Extra sequence included	104210212	104260119	-5475	7.12E-23	72	54	DEL	7.12E-23
1-303	1	104369918	104415377	NONE	Extra sequence included	104369918	104415377	-18984	7.12E-23	72	53	DEL	7.12E-23
1-305	1	104590035	104677798	NONE	Extra sequence included	104590035	104677798	-19726	9.90E-23	63	50	DEL	9.90E-23
1-308	1	105077409	105104761	NONE	Extra sequence included	105077409	105104761	-27352	0.00E+00	63	0	DEL	0.00E+00
1-313	1	105751199	105777451	NONE	Extra sequence included	105751199	105777451	-7125	7.12E-23	63	52	DEL	7.12E-23
1-318	1	108027670	108059223	NONE	Extra sequence included	108027670	108059223	-6007	7.12E-23	46	37	DEL	7.12E-23
1-326	1	110033319	110066208	NONE	Extra sequence included	110033319	110066208	-6775	7.12E-23	47	39	DEL	7.12E-23
1-328	1	110279081	110335183	NONE	Extra sequence included	110279081	110335183	-9064	7.12E-23	67	49	DEL	7.12E-23
1-333	1	110754459	110810371	NONE	Extra sequence included	110754459	110810371	-14630	7.12E-23	38	34	DEL	7.12E-23
1-334	1	111173896	111226968	NONE	Extra sequence included	111173896	111226968	-21516	1.00E-22	56	49	DEL	1.00E-22
1-344	1	112684678	112695631	NONE	Extra sequence included	112684678	112695631	-10953	0.00E+00	50	0	DEL	0.00E+00
1-345	1	112721822	112792318	NONE	Extra sequence included	112721822	112792318	-25414	7.12E-23	39	31	DEL	7.12E-23
1-363	1	118933868	118959767	NONE	Extra sequence included	118933868	118959767	-25899	0.00E+00	44	0	DEL	0.00E+00
1-371	1	121184318	121239338	NONE	Extra sequence included	121184318	121239338	-6475	5.01E-17	41	30	DEL	5.01E-17
1-372	1	121480323	121622120	NONE	Extra sequence included	121480323	121622120	-24562	9.92E-23	42	29	DEL	9.92E-23
1-388	1	126674915	126709526	NONE	Extra sequence included	126674915	126709526	-5335	7.12E-23	82	69	DEL	7.12E-23
1-389	1	126828766	126835029	NONE	Extra sequence included	126828766	126835029	-6263	0.00E+00	70	0	DEL	0.00E+00
1-391	1	126958203	127017834	NONE	Extra sequence included	126958203	127017834	-59631	0.00E+00	48	0	DEL	0.00E+00
1-400	1	130545432	130595558	NONE	Extra sequence included	130545432	130595558	-20841	7.12E-23	64	44	DEL	7.12E-23
1-404	1	131297955	131356285	NONE	Extra sequence included	131297955	131356285	-9942	1.46E-20	33	17	DEL	1.46E-20
1-413	1	137701190	137717159	NONE	Extra sequence included	137701190	137717159	-15969	0.00E+00	56	0	DEL	0.00E+00
1-415	1	139266483	139301792	NONE	Extra sequence included	139266483	139301792	-13135	1.01E-22	60	46	DEL	1.01E-22
1-418	1	140373047	140386612	NONE	Extra sequence included	140373047	140386612	-13565	0.00E+00	66	0	DEL	0.00E+00
1-419	1	140820110	140837730	NONE	Extra sequence included	140820110	140837730	-17620	0.00E+00	68	0	DEL	0.00E+00
1-424	1	141647883	141667511	NONE	Extra sequence included	141647883	141667511	-19628	0.00E+00	29	0	DEL	0.00E+00
1-428	1	142620365	142730454	NONE	Extra sequence included	142620365	142730454	-31102	7.12E-23	27	21	DEL	7.12E-23
1-437	1	145509220	145612798	NONE	Extra sequence included	145509220	145612798	-7353	2.97E-07	39	29	DEL	2.97E-07
1-449	1	154128997	154163930	NONE	Extra sequence included	154128997	154163930	-5132	7.12E-23	77	55	DEL	7.12E-23
1-456	1	155835038	155855420	NONE	Extra sequence included	155835038	155855420	-20382	0.00E+00	65	0	DEL	0.00E+00
1-472	1	159803708	159810436	NONE	Extra sequence included	159803708	159810436	-6728	0.00E+00	59	0	DEL	0.00E+00
1-473	1	159937956	160001042	NONE	Extra sequence included	159937956	160001042	-6869	7.72E-22	50	44	DEL	7.72E-22
1-492	1	164134625	164229972	NONE	Extra sequence included	164134625	164229972	-14268	7.12E-23	42	41	DEL	7.12E-23
1-499	1	165889823	165906350	NONE	Extra sequence included	165889823	165906350	-16527	0.00E+00	91	0	DEL	0.00E+00
1-503	1	166580255	166702431	NONE	Extra sequence included	166580255	166702431	-19060	7.12E-23	51	45	DEL	7.12E-23
1-505	1	167072780	167142571	NONE	Extra sequence included	167072780	167142571	-13664	7.12E-23	38	28	DEL	7.12E-23
1-517	1	171555861	171566922	NONE	Extra sequence included	171555861	171566922	-11061	0.00E+00	57	0	DEL	0.00E+00
1-538	1	178207931	178252351	NONE	Extra sequence included	178207931	178252351	-5085	8.78E-09	19	16	DEL	8.78E-09
1-540	1	180096193	180141258	NONE	Extra sequence included	180096193	180141258	-7921	7.12E-23	68	47	DEL	7.12E-23
1-544	1	181069521	181097072	NONE	Extra sequence included	181069521	181097072	-5319	7.12E-23	60	50	DEL	7.12E-23

1-560	1	186933843	187020026	NONE	Extra sequence included	186933843	187020026	-32147	9.76E-23	22	15	DEL	9.76E-23
1-571	1	194251740	194287472	NONE	Extra sequence included	194251740	194287472	-5906	7.12E-23	43	34	DEL	7.12E-23
1-580	1	195839082	195907820	NONE	Extra sequence included	195839082	195907820	-9184	7.12E-23	59	38	DEL	7.12E-23
1-586	1	197133521	197185660	NONE	Extra sequence included	197133521	197185660	-6981	7.12E-23	72	57	DEL	7.12E-23
1-592	1	198410064	198528623	NONE	Extra sequence included	198410064	198528623	-22037	7.12E-23	26	21	DEL	7.12E-23
1-604	1	205677080	205727515	NONE	Extra sequence included	205677080	205727515	-7753	7.12E-23	64	39	DEL	7.12E-23
1-605	1	206008404	206084449	NONE	Extra sequence included	206008404	206084449	-9059	7.72E-22	64	36	DEL	7.72E-22
1-625	1	211330050	211351585	NONE	Extra sequence included	211330050	211351585	-21535	0.00E+00	95	0	DEL	0.00E+00
1-626	1	211388124	211405016	NONE	Extra sequence included	211388124	211405016	-16892	0.00E+00	94	0	DEL	0.00E+00
1-652	1	219762217	219800257	NONE	Extra sequence included	219762217	219800257	-9541	7.12E-23	48	35	DEL	7.12E-23
1-662	1	224044564	224092873	NONE	Extra sequence included	224044564	224092873	-5811	7.12E-23	60	47	DEL	7.12E-23
1-665	1	224748719	224798467	NONE	Extra sequence included	224748719	224798467	-6664	7.12E-23	60	55	DEL	7.12E-23
1-676	1	227578641	227592439	NONE	Extra sequence included	227578641	227592439	-13798	0.00E+00	70	0	DEL	0.00E+00
1-688	1	231171314	231212067	NONE	Extra sequence included	231171314	231212067	-14023	9.96E-23	84	63	DEL	9.96E-23
1-689	1	231396141	231433420	NONE	Extra sequence included	231396141	231433420	-13023	7.12E-23	83	62	DEL	7.12E-23
1-690	1	231470291	231490339	NONE	Extra sequence included	231470291	231490339	-5230	7.12E-23	71	49	DEL	7.12E-23
1-723	1	246860019	246950602	NONE	Extra sequence included	246860019	246950602	-20193	7.12E-23	74	51	DEL	7.12E-23
1-725	1	247318034	247372742	NONE	Extra sequence included	247318034	247372742	-54708	0.00E+00	86	0	DEL	0.00E+00
1-748	1	260794833	260861409	NONE	Extra sequence included	260794833	260861409	-20743	7.12E-23	35	29	DEL	7.12E-23
1-755	1	265462378	265542107	NONE	Extra sequence included	265462378	265542107	-79729	0.00E+00	70	0	DEL	0.00E+00
1-760	1	267430985	267477916	NONE	Extra sequence included	267430985	267477916	-6255	7.12E-23	69	51	DEL	7.12E-23
1-761	1	267512883	267547122	NONE	Extra sequence included	267512883	267547122	-6427	7.12E-23	74	56	DEL	7.12E-23
1-763	1	267698701	267775310	NONE	Extra sequence included	267698701	267775310	-9735	7.12E-23	77	61	DEL	7.12E-23
1-764	1	267962675	267982885	NONE	Extra sequence included	267962675	267982885	-8158	7.12E-23	89	59	DEL	7.12E-23
1-767	1	270713496	270777003	NONE	Extra sequence included	270713496	270777003	-7153	7.12E-23	68	53	DEL	7.12E-23
1-777	1	273433614	273445300	NONE	Extra sequence included	273433614	273445300	-11686	0.00E+00	58	0	DEL	0.00E+00
1-780	1	273956824	273985322	NONE	Extra sequence included	273956824	273985322	-5068	7.12E-23	62	51	DEL	7.12E-23
1-782	1	275636343	275721134	NONE	Extra sequence included	275636343	275721134	-9682	1.63E-14	31	25	DEL	1.63E-14
1-783	1	278083330	278115542	NONE	Extra sequence included	278083330	278115542	-6295	7.12E-23	64	49	DEL	7.12E-23
1-787	1	283277674	283307829	NONE	Extra sequence included	283277674	283307829	-10719	7.12E-23	83	67	DEL	7.12E-23
1-800	1	289208920	289247331	NONE	Extra sequence included	289208920	289247331	-12452	7.12E-23	80	62	DEL	7.12E-23
1-802	1	289723541	289739179	NONE	Extra sequence included	289723541	289739179	-15638	0.00E+00	82	0	DEL	0.00E+00
1-808	1	291118290	291178231	NONE	Extra sequence included	291118290	291178231	-59941	0.00E+00	89	0	DEL	0.00E+00
2-7	2	5277877	5357398	NONE	Extra sequence included	5277877	5357398	-36288	9.89E-23	63	49	DEL	9.89E-23
2-28	2	16187154	16197907	NONE	Extra sequence included	16187154	16197907	-10753	0.00E+00	65	0	DEL	0.00E+00
2-29	2	16428574	16509732	NONE	Extra sequence included	16428574	16509732	-81158	0.00E+00	59	0	DEL	0.00E+00
2-43	2	20229274	20267925	NONE	Extra sequence included	20229274	20267925	-6291	7.27E-23	45	41	DEL	7.27E-23
2-56	2	23874181	23914291	NONE	Extra sequence included	23874181	23914291	-8593	7.27E-23	57	47	DEL	7.27E-23
2-58	2	24242644	24283718	NONE	Extra sequence included	24242644	24283718	-41074	0.00E+00	63	0	DEL	0.00E+00
2-66	2	26579653	26616937	NONE	Extra sequence included	26579653	26616937	-9167	7.27E-23	59	49	DEL	7.27E-23
2-70	2	27458674	27563602	NONE	Extra sequence included	27458674	27563602	-17660	7.27E-23	50	39	DEL	7.27E-23
2-92	2	36572633	36597224	NONE	Extra sequence included	36572633	36597224	-7969	7.27E-23	48	38	DEL	7.27E-23
2-109	2	44897038	44982266	NONE	Extra sequence included	44897038	44982266	-25343	9.78E-23	53	36	DEL	9.78E-23
2-127	2	48415158	48511406	NONE	Extra sequence included	48415158	48511406	-10691	2.19E-17	36	31	DEL	2.19E-17
2-141	2	54209380	54266604	NONE	Extra sequence included	54209380	54266604	-7295	7.27E-23	52	41	DEL	7.27E-23
2-143	2	57808859	57834550	NONE	Extra sequence included	57808859	57834550	-7690	7.27E-23	76	51	DEL	7.27E-23
2-165	2	67401526	67442866	NONE	Extra sequence included	67401526	67442866	-12401	7.27E-23	93	70	DEL	7.27E-23
2-167	2	67693641	67729820	NONE	Extra sequence included	67693641	67729820	-5966	7.27E-23	90	63	DEL	7.27E-23
2-178	2	70084652	70098110	NONE	Extra sequence included	70084652	70098110	-13458	0.00E+00	70	0	DEL	0.00E+00
2-180	2	70426411	70499468	NONE	Extra sequence included	70426411	70499468	-13131	7.27E-23	74	60	DEL	7.27E-23
2-187	2	72422442	72545382	NONE	Extra sequence included	72422442	72545382	-72111	9.48E-23	70	62	DEL	9.48E-23
2-205	2	79143731	79237392	NONE	Extra sequence included	79143731	79237392	-24875	7.27E-23	39	34	DEL	7.27E-23
2-208	2	80501077	80553095	NONE	Extra sequence included	80501077	80553095	-13670	7.27E-23	50	45	DEL	7.27E-23
2-215	2	82335773	82411641	NONE	Extra sequence included	82335773	82411641	-11190	7.27E-23	43	35	DEL	7.27E-23
2-216	2	82916205	82946965	NONE	Extra sequence included	82916205	82946965	-5721	7.27E-23	50	42	DEL	7.27E-23
2-217	2	83393551	83447455	NONE	Extra sequence included	83393551	83447455	-53904	0.00E+00	57	0	DEL	0.00E+00
2-221	2	84804980	84888309	NONE	Extra sequence included	84804980	84888309	-11523	7.27E-23	41	29	DEL	7.27E-23
2-225	2	86249102	86285060	NONE	Extra sequence included	86249102	86285060	-6525	7.27E-23	39	31	DEL	7.27E-23
2-242	2	93521443	93564971	NONE	Extra sequence included	93521443	93564971	-6010	7.27E-23	55	44	DEL	7.27E-23
2-251	2	96537932	96598599	NONE	Extra sequence included	96537932	96598599	-13644	9.89E-23	61	49	DEL	9.89E-23
2-252	2	96608228	96651000	NONE	Extra sequence included	96608228	96651000	-9658	7.27E-23	59	44	DEL	7.27E-23
2-265	2	99804189	99882008	NONE	Extra sequence included	99804189	99882008	-25267	7.27E-23	69	51	DEL	7.27E-23
2-283	2	189731384	189794063	NONE	Extra sequence included	189731384	189794063	-7272	7.27E-23	60	48	DEL	7.27E-23
2-285	2	196502089	196517494	NONE	Extra sequence included	196502089	196517494	-15405	0.00E+00	59	0	DEL	0.00E+00
2-294	2	203038680	203098513	NONE	Extra sequence included	203038680	203098513	-12383	7.27E-23	34	23	DEL	7.27E-23
2-297	2	203674301	203683246	NONE	Extra sequence included	203674301	203683246	-8945	0.00E+00	85	0	DEL	0.00E+00
2-302	2	207725143	207760636	NONE	Extra sequence included	207725143	207760636	-8654	7.27E-23	51	39	DEL	7.27E-23
2-306	2	208733648	208824817	NONE	Extra sequence included	208733648	208824817	-25525	7.27E-23	39	30	DEL	7.27E-23
2-310	2	209622991	209698457	NONE	Extra sequence included	209622991	209698457	-14683	7.27E-23	70	48	DEL	7.27E-23
2-318	2	212471399	212500175	NONE	Extra sequence included	212471399	212500175	-7517	7.27E-23	68	50	DEL	7.27E-23
2-321	2	216668720	216708106	NONE	Extra sequence included	216668720	216708106	-39386	0.00E+00	61	0	DEL	0.00E+00
2-323	2	216908989	216953458	NONE	Extra sequence included	216908989	216953458	-8367	7.27E-23	76	61	DEL	7.27E-23
2-334	2	220977117	220992107	NONE	Extra sequence included	220977117	220992107	-14990	0.00E+00	51	0	DEL	0.00E+00
2-335	2	221124130	221144168	NONE	Extra sequence included	221124130	221144168	-6510	7.27E-23	58	43	DEL	7.27E-23
2-339	2	223146493	223232865	NONE	Extra sequence included	223146493	223232865	-9236	2.85E-16	43	32	DEL	2.85E-16
2-343	2	226913070	226946297	NONE	Extra sequence included	226913070	226946297	-9029	7.27E-23	82	67	DEL	7.27E-23
2-348	2	229031914	229053679	NONE	Extra sequence included	229031914	229053679	-5684	7.27E-23	57	37	DEL	7.27E-23
2-353	2	230474746	230536281	NONE	Extra sequence included	230474746	230536281	-14513	7.27E-23	37	35	DEL	7.27E-23
2-355	2	230757787	230786594	NONE	Extra sequence included	230757787	230786594	-8131	7.27E-23	39	30	DEL	7.27E-23
3-12	3	12305816	12346981	NONE	Extra sequence included	12305816	12346981	-5999	7.24E-23	72	54	DEL	7.24E-23
3-23	3	25311610	25442105	NONE	Extra sequence included	25311610	25442105	-36463	7.24E-23	38	28	DEL	7.24E-23
3-33	3	28358570	28413919	NONE	Extra sequence included	28358570	28413919	-55349	0.00E+00	77	0	DEL	0.00E+00
3-38	3	29466356	29498000	NONE	Extra sequence included	29466356	29498000	-15792	1.08E-22	69	54	DEL	1.08E-22
3-43	3	31062604	31109943	NONE	Extra sequence included	31062604	31109943	-5100	4.07E-08	20	17	DEL	4.07E-08
3-46	3	31621250	31688683	NONE	Extra sequence included	31621250	31688683	-15464	7.24E-23	27	23	DEL	7.24E-23
3-57	3	34961201	35019280	NONE	Extra sequence included	34961201	35019280	-9254	7.24E-23	53	41	DEL	7.24E-23
3-61	3	36007654	36057961	NONE	Extra sequence included	36007654	36057961	-9637	7.24E-23	88	66	DEL	7.24E-23
3-71	3	38081702	38138367	NONE	Extra sequence included	38081702	38138367	-8181	7.24E-23	67	58	DEL	7.24E-23
3-82	3	40964397	40987779	NONE	Extra sequence included	40964397	40987779	-5033	7.24E-23	75	58	DEL	7.24E-23
3-83	3	41016316	41054474	NONE	Extra sequence included	41016316	41054474	-8286	7.24E-23	73	55	DEL	7.24E-23
3-85	3	41526118	41620152	NONE	Extra sequence included	41526118	41620152	-94034	0.00E+00	77	0	DEL	0.00E+00

3-90	3	42556109	42584982	NONE	Extra sequence included	42556109	42584982	-7818	7.24E-23	38	31	DEL	7.24E-23
3-110	3	47400209	47434679	NONE	Extra sequence included	47400209	47434679	-9534	7.24E-23	56	50	DEL	7.24E-23
3-114	3	48710686	48756137	NONE	Extra sequence included	48710686	48756137	-12525	7.24E-23	41	31	DEL	7.24E-23
3-127	3	51895275	51962850	NONE	Extra sequence included	51895275	51962850	-14059	7.24E-23	59	52	DEL	7.24E-23
3-131	3	53130095	53155595	NONE	Extra sequence included	53130095	53155595	-7243	7.24E-23	55	38	DEL	7.24E-23
3-135	3	54253523	54283330	NONE	Extra sequence included	54253523	54283330	-29807	0.00E+00	66	0	DEL	0.00E+00
3-142	3	56795594	56822454	NONE	Extra sequence included	56795594	56822454	-26860	0.00E+00	46	0	DEL	0.00E+00
3-145	3	57504186	57595682	NONE	Extra sequence included	57504186	57595682	-18541	1.07E-22	47	37	DEL	1.07E-22
3-158	3	64234299	64300989	NONE	Extra sequence included	64234299	64300989	-66690	0.00E+00	28	0	DEL	0.00E+00
3-159	3	64551172	64602633	NONE	Extra sequence included	64551172	64602633	-10710	7.24E-23	31	28	DEL	7.24E-23
3-165	3	65955051	66029490	NONE	Extra sequence included	65955051	66029490	-8866	7.24E-23	68	55	DEL	7.24E-23
3-180	3	75912409	75987143	NONE	Extra sequence included	75912409	75987143	-11543	7.24E-23	49	36	DEL	7.24E-23
3-183	3	76865175	76900469	NONE	Extra sequence included	76865175	76900469	-10806	7.24E-23	55	37	DEL	7.24E-23
3-185	3	77287286	77384848	NONE	Extra sequence included	77287286	77384848	-22621	7.24E-23	36	32	DEL	7.24E-23
3-191	3	78628510	78737132	NONE	Extra sequence included	78628510	78737132	-13830	7.24E-23	49	36	DEL	7.24E-23
3-192	3	78977725	79055752	NONE	Extra sequence included	78977725	79055752	-11219	7.24E-23	57	44	DEL	7.24E-23
3-198	3	80196996	80266465	NONE	Extra sequence included	80196996	80266465	-9902	2.68E-16	30	20	DEL	2.68E-16
3-199	3	80489161	80558342	NONE	Extra sequence included	80489161	80558342	-12600	7.24E-23	27	19	DEL	7.24E-23
3-206	3	83434750	83506439	NONE	Extra sequence included	83434750	83506439	-18825	7.24E-23	40	27	DEL	7.24E-23
3-212	3	85643383	85670032	NONE	Extra sequence included	85643383	85670032	-5120	7.24E-23	61	43	DEL	7.24E-23
3-217	3	87813737	87945650	NONE	Extra sequence included	87813737	87945650	-22340	7.24E-23	27	24	DEL	7.24E-23
3-218	3	88072974	88139166	NONE	Extra sequence included	88072974	88139166	-16242	7.24E-23	28	22	DEL	7.24E-23
3-226	3	91821298	91840294	NONE	Extra sequence included	91821298	91840294	-8214	7.24E-23	55	48	DEL	7.24E-23
3-242	3	101451070	101606418	NONE	Extra sequence included	101451070	101606418	-40022	7.24E-23	28	17	DEL	7.24E-23
3-251	3	103391810	103472919	NONE	Extra sequence included	103391810	103472919	-18707	1.06E-22	41	30	DEL	1.06E-22
3-258	3	105672639	105705585	NONE	Extra sequence included	105672639	105705585	-12358	7.24E-23	55	33	DEL	7.24E-23
3-271	3	112398583	112445540	NONE	Extra sequence included	112398583	112445540	-5153	6.17E-16	41	35	DEL	6.17E-16
3-273	3	112671699	112712289	NONE	Extra sequence included	112671699	112712289	-5144	7.24E-23	60	45	DEL	7.24E-23
3-274	3	112983563	113051758	NONE	Extra sequence included	112983563	113051758	-14155	7.24E-23	72	63	DEL	7.24E-23
3-279	3	114912673	114953515	NONE	Extra sequence included	114912673	114953515	-40842	0.00E+00	20	0	DEL	0.00E+00
3-298	3	126626949	126676058	NONE	Extra sequence included	126626949	126676058	-49109	0.00E+00	77	0	DEL	0.00E+00
3-300	3	127419524	127486865	NONE	Extra sequence included	127419524	127486865	-8741	6.98E-15	33	22	DEL	6.98E-15
3-301	3	130847390	130863131	NONE	Extra sequence included	130847390	130863131	-15741	0.00E+00	55	0	DEL	0.00E+00
3-303	3	131086347	131150062	NONE	Extra sequence included	131086347	131150062	-9957	7.24E-23	54	36	DEL	7.24E-23
3-307	3	132091336	132130662	NONE	Extra sequence included	132091336	132130662	-5145	7.24E-23	65	52	DEL	7.24E-23
3-311	3	135804426	135891454	NONE	Extra sequence included	135804426	135891454	-26375	7.24E-23	66	59	DEL	7.24E-23
3-321	3	137980524	138021025	NONE	Extra sequence included	137980524	138021025	-11038	7.24E-23	50	43	DEL	7.24E-23
3-326	3	139273036	139289239	NONE	Extra sequence included	139273036	139289239	-6572	7.24E-23	64	55	DEL	7.24E-23
3-348	3	145341261	145397770	NONE	Extra sequence included	145341261	145397770	-5802	9.06E-11	27	25	DEL	9.06E-11
3-349	3	147046539	147056683	NONE	Extra sequence included	147046539	147056683	-5473	1.06E-22	48	31	DEL	1.06E-22
3-351	3	147239596	147284958	NONE	Extra sequence included	147239596	147284958	-5795	7.24E-23	53	42	DEL	7.24E-23
3-353	3	147494740	147544785	NONE	Extra sequence included	147494740	147544785	-8729	2.42E-19	24	17	DEL	2.42E-19
3-354	3	148214381	148238057	NONE	Extra sequence included	148214381	148238057	-23676	0.00E+00	32	0	DEL	0.00E+00
3-357	3	149182402	149219271	NONE	Extra sequence included	149182402	149219271	-6922	7.24E-23	68	60	DEL	7.24E-23
3-362	3	150873961	150916675	NONE	Extra sequence included	150873961	150916675	-42714	0.00E+00	76	0	DEL	0.00E+00
3-366	3	152443850	152498895	NONE	Extra sequence included	152443850	152498895	-22890	7.24E-23	46	36	DEL	7.24E-23
3-367	3	152560263	152619580	NONE	Extra sequence included	152560263	152619580	-19452	7.24E-23	54	42	DEL	7.24E-23
3-373	3	160872388	160901900	NONE	Extra sequence included	160872388	160901900	-7661	7.24E-23	54	39	DEL	7.24E-23
3-376	3	161293801	161386709	NONE	Extra sequence included	161293801	161386709	-20763	7.24E-23	32	26	DEL	7.24E-23
3-378	3	161772709	161827626	NONE	Extra sequence included	161772709	161827626	-6626	7.24E-23	46	41	DEL	7.24E-23
3-379	3	162955356	163120463	NONE	Extra sequence included	162955356	163120463	-26072	7.24E-23	25	23	DEL	7.24E-23
3-385	3	164023907	164076963	NONE	Extra sequence included	164023907	164076963	-19560	7.24E-23	45	36	DEL	7.24E-23
3-388	3	164819473	164869035	NONE	Extra sequence included	164819473	164869035	-5218	1.48E-18	52	44	DEL	1.48E-18
3-395	3	172243168	172387727	NONE	Extra sequence included	172243168	172387727	-25874	7.24E-23	40	31	DEL	7.24E-23
3-408	3	176351543	176429235	NONE	Extra sequence included	176351543	176429235	-9273	2.68E-16	34	28	DEL	2.68E-16
3-413	3	182043319	182070680	NONE	Extra sequence included	182043319	182070680	-5724	7.24E-23	69	50	DEL	7.24E-23
3-414	3	182157317	182303869	NONE	Extra sequence included	182157317	182303869	-146552	0.00E+00	48	0	DEL	0.00E+00
3-417	3	183406367	183463426	NONE	Extra sequence included	183406367	183463426	-13276	1.06E-22	63	48	DEL	1.06E-22
3-424	3	185009595	185021604	NONE	Extra sequence included	185009595	185021604	-12009	0.00E+00	69	0	DEL	0.00E+00
3-431	3	186015930	186073917	NONE	Extra sequence included	186015930	186073917	-8132	7.24E-23	58	52	DEL	7.24E-23
3-444	3	189083282	189114346	NONE	Extra sequence included	189083282	189114346	-11202	7.24E-23	82	69	DEL	7.24E-23
3-457	3	198495222	198506489	NONE	Extra sequence included	198495222	198506489	-11267	0.00E+00	35	0	DEL	0.00E+00
3-462	3	201057691	201092283	NONE	Extra sequence included	201057691	201092283	-10416	7.24E-23	60	41	DEL	7.24E-23
3-465	3	201914016	201934936	NONE	Extra sequence included	201914016	201934936	-8729	1.02E-22	42	32	DEL	1.02E-22
3-467	3	202298616	202310249	NONE	Extra sequence included	202298616	202310249	-11633	0.00E+00	77	0	DEL	0.00E+00
3-478	3	204943691	205058606	NONE	Extra sequence included	204943691	205058606	-16836	7.24E-23	39	29	DEL	7.24E-23
3-486	3	208074059	208131578	NONE	Extra sequence included	208074059	208131578	-7842	7.24E-23	67	52	DEL	7.24E-23
3-516	3	215714139	215739637	NONE	Extra sequence included	215714139	215739637	-6574	7.24E-23	78	55	DEL	7.24E-23
3-530	3	219942292	219952248	NONE	Extra sequence included	219942292	219952248	-9956	0.00E+00	98	0	DEL	0.00E+00
3-532	3	220678583	220738872	NONE	Extra sequence included	220678583	220738872	-13509	7.24E-23	36	26	DEL	7.24E-23
3-533	3	221618617	221690468	NONE	Extra sequence included	221618617	221690468	-28852	1.09E-22	52	33	DEL	1.09E-22
3-535	3	222044235	222054204	NONE	Extra sequence included	222044235	222054204	-9969	0.00E+00	54	0	DEL	0.00E+00
3-547	3	227527692	227589254	NONE	Extra sequence included	227527692	227589254	-13720	7.24E-23	73	51	DEL	7.24E-23
3-550	3	228497488	228515149	NONE	Extra sequence included	228497488	228515149	-17661	0.00E+00	63	0	DEL	0.00E+00
3-556	3	229579957	229588562	NONE	Extra sequence included	229579957	229588562	-8605	0.00E+00	96	0	DEL	0.00E+00
4-1	4	5001638	5047791	NONE	Extra sequence included	5001638	5047791	-11395	7.57E-23	57	40	DEL	7.57E-23
4-19	4	7958383	8014404	NONE	Extra sequence included	7958383	8014404	-7530	3.99E-20	31	28	DEL	3.99E-20
4-27	4	9823145	9906061	NONE	Extra sequence included	9823145	9906061	-51392	7.57E-23	72	54	DEL	7.57E-23
4-36	4	12539598	12588482	NONE	Extra sequence included	12539598	12588482	-9193	7.57E-23	47	29	DEL	7.57E-23
4-44	4	18269470	18288562	NONE	Extra sequence included	18269470	18288562	-19092	0.00E+00	75	0	DEL	0.00E+00
4-57	4	25549999	25595657	NONE	Extra sequence included	25549999	25595657	-7935	7.57E-23	62	48	DEL	7.57E-23
4-61	4	29008926	29036595	NONE	Extra sequence included	29008926	29036595	-5599	7.57E-23	69	52	DEL	7.57E-23
4-62	4	29334707	29370246	NONE	Extra sequence included	29334707	29370246	-5931	7.57E-23	36	29	DEL	7.57E-23
4-64	4	29908665	29923925	NONE	Extra sequence included	29908665	29923925	-15260	0.00E+00	58	0	DEL	0.00E+00
4-76	4	36884594	36987866	NONE	Extra sequence included	36884594	36987866	-23225	7.57E-23	41	34	DEL	7.57E-23
4-83	4	38533673	38569161	NONE	Extra sequence included	38533673	38569161	-12539	1.11E-22	63	47	DEL	1.11E-22
4-89	4	41725817	41768748	NONE	Extra sequence included	41725817	41768748	-16853	7.57E-23	44	32	DEL	7.57E-23
4-100	4	43318349	43334262	NONE	Extra sequence included	43318349	43334262	-15913	0.00E+00	61	0	DEL	0.00E+00
4-109	4	48876436	48943497	NONE	Extra sequence included	48876436	48943497	-17802	7.57E-23	59	51	DEL	7.57E-23
4-111	4	49074113	49118066	NONE	Extra sequence included	49074113	49118066	-6483	7.57E-23	38	31	DEL	7.57E-23
4-121	4	52925542	53023007	NONE	Extra sequence included	52925542	53023007	-19616	7.57E-23	30	28	DEL	7.57E-23

4-125	4	53987560	54046663	NONE	Extra sequence included	53987560	54046663		-7108	5.36E-17		39	29	DEL	5.36E-17
4-132	4	56997879	57081955	NONE	Extra sequence included	56997879	57081955		-9677	7.57E-23		53	47	DEL	7.57E-23
4-146	4	63999799	64011570	NONE	Extra sequence included	63999799	64011570		-11771	0.00E+00		44	0	DEL	0.00E+00
4-182	4	77175912	77262304	NONE	Extra sequence included	77175912	77262304		-10296	2.58E-07		12	11	DEL	2.58E-07
4-200	4	82472497	82535647	NONE	Extra sequence included	82472497	82535647		-12823	7.57E-23		48	35	DEL	7.57E-23
4-208	4	84775961	84820975	NONE	Extra sequence included	84775961	84820975		-9355	7.57E-23		40	31	DEL	7.57E-23
4-219	4	87205670	87263411	NONE	Extra sequence included	87205670	87263411		-13883	7.57E-23		70	54	DEL	7.57E-23
4-228	4	89074281	89181128	NONE	Extra sequence included	89074281	89181128		-26320	1.10E-22		77	61	DEL	1.10E-22
4-242	4	93416349	93879654	NONE	Extra sequence included	93416349	93879654		-463305	0.00E+00		25	0	DEL	0.00E+00
4-243	4	94088221	94176233	NONE	Extra sequence included	94088221	94176233		-10095	1.58E-18		38	33	DEL	1.58E-18
4-248	4	95554499	95668337	NONE	Extra sequence included	95554499	95668337		-12152	1.22E-06		13	12	DEL	1.22E-06
4-262	4	100704928	100721494	NONE	Extra sequence included	100704928	100721494		-16566	0.00E+00		63	0	DEL	0.00E+00
4-264	4	100925040	100968525	NONE	Extra sequence included	100925040	100968525		-5252	7.57E-23		63	53	DEL	7.57E-23
4-284	4	110959084	110984586	NONE	Extra sequence included	110959084	110984586		-6179	7.57E-23		40	35	DEL	7.57E-23
4-285	4	111257933	111375509	NONE	Extra sequence included	111257933	111375509		-29301	7.57E-23		30	21	DEL	7.57E-23
4-290	4	113095976	113141103	NONE	Extra sequence included	113095976	113141103		-6669	7.57E-23		39	31	DEL	7.57E-23
4-301	4	116870721	116979321	NONE	Extra sequence included	116870721	116979321		-45624	7.57E-23		42	37	DEL	7.57E-23
4-313	4	121079235	121132893	NONE	Extra sequence included	121079235	121132893		-8154	7.57E-23		41	35	DEL	7.57E-23
4-315	4	121570111	121622763	NONE	Extra sequence included	121570111	121622763		-16802	7.57E-23		39	27	DEL	7.57E-23
4-330	4	134172398	134246393	NONE	Extra sequence included	134172398	134246393		-73995	0.00E+00		45	0	DEL	0.00E+00
4-333	4	135554247	135580163	NONE	Extra sequence included	135554247	135580163		-25916	0.00E+00		61	0	DEL	0.00E+00
4-360	4	143636482	143774802	NONE	Extra sequence included	143636482	143774802		-15541	1.57E-08		18	14	DEL	1.57E-08
4-370	4	148981316	149024581	NONE	Extra sequence included	148981316	149024581		-11874	7.57E-23		34	31	DEL	7.57E-23
4-371	4	149332977	149409075	NONE	Extra sequence included	149332977	149409075		-9314	7.57E-23		47	36	DEL	7.57E-23
4-393	4	157873600	157947119	NONE	Extra sequence included	157873600	157947119		-73519	0.00E+00		72	0	DEL	0.00E+00
4-413	4	161861607	161936954	NONE	Extra sequence included	161861607	161936954		-14078	7.57E-23		43	40	DEL	7.57E-23
4-417	4	162369850	162435095	NONE	Extra sequence included	162369850	162435095		-10538	1.54E-20		33	19	DEL	1.54E-20
4-423	4	163996726	164047014	NONE	Extra sequence included	163996726	164047014		-50288	0.00E+00		72	0	DEL	0.00E+00
4-438	4	168139399	168218824	NONE	Extra sequence included	168139399	168218824		-14423	7.57E-23		50	33	DEL	7.57E-23
4-458	4	174327680	174362515	NONE	Extra sequence included	174327680	174362515		-10961	7.57E-23		67	55	DEL	7.57E-23
4-462	4	174929778	174973178	NONE	Extra sequence included	174929778	174973178		-6532	7.57E-23		85	65	DEL	7.57E-23
4-471	4	182369694	182462243	NONE	Extra sequence included	182369694	182462243		-16013	7.57E-23		69	55	DEL	7.57E-23
4-486	4	189147113	189161261	NONE	Extra sequence included	189147113	189161261		-14148	0.00E+00		36	0	DEL	0.00E+00
4-490	4	190081848	190097559	NONE	Extra sequence included	190081848	190097559		-15711	0.00E+00		68	0	DEL	0.00E+00
4-500	4	194482241	194527176	NONE	Extra sequence included	194482241	194527176		-11830	7.57E-23		72	55	DEL	7.57E-23
4-511	4	200054302	200064483	NONE	Extra sequence included	200054302	200064483		-10181	0.00E+00		51	0	DEL	0.00E+00
4-512	4	200273074	200299531	NONE	Extra sequence included	200273074	200299531		-9137	7.57E-23		65	43	DEL	7.57E-23
4-522	4	203095185	203124130	NONE	Extra sequence included	203095185	203124130		-28945	0.00E+00		60	0	DEL	0.00E+00
4-523	4	203466115	203513310	NONE	Extra sequence included	203466115	203513310		-19704	7.57E-23		53	45	DEL	7.57E-23
4-526	4	203851929	203879669	NONE	Extra sequence included	203851929	203879669		-6365	7.57E-23		30	27	DEL	7.57E-23
4-539	4	206112343	206171586	NONE	Extra sequence included	206112343	206171586		-12693	7.57E-23		51	42	DEL	7.57E-23
4-540	4	206720073	206749777	NONE	Extra sequence included	206720073	206749777		-13172	1.08E-22		30	25	DEL	1.08E-22
4-543	4	207598749	207671176	NONE	Extra sequence included	207598749	207671176		-61135	1.10E-22		72	55	DEL	1.10E-22
4-578	4	218049167	218144127	NONE	Extra sequence included	218049167	218144127		-9830	8.17E-22		59	47	DEL	8.17E-22
4-581	4	219384332	219393303	NONE	Extra sequence included	219384332	219393303		-8971	0.00E+00		71	0	DEL	0.00E+00
4-582	4	219918333	219958908	NONE	Extra sequence included	219918333	219958908		-5280	7.57E-23		64	52	DEL	7.57E-23
4-587	4	221447661	221495139	NONE	Extra sequence included	221447661	221495139		-47478	0.00E+00		66	0	DEL	0.00E+00
4-592	4	222570676	222621398	NONE	Extra sequence included	222570676	222621398		-14783	7.57E-23		52	40	DEL	7.57E-23
4-608	4	225144541	225153910	NONE	Extra sequence included	225144541	225153910		-9369	0.00E+00		61	0	DEL	0.00E+00
4-610	4	225274398	225299984	NONE	Extra sequence included	225274398	225299984		-5887	7.57E-23		60	51	DEL	7.57E-23
4-614	4	225862692	225909038	NONE	Extra sequence included	225862692	225909038		-5784	7.57E-23		50	45	DEL	7.57E-23
4-615	4	225977488	226003150	NONE	Extra sequence included	225977488	226003150		-6809	7.57E-23		60	48	DEL	7.57E-23
4-618	4	226495975	226521040	NONE	Extra sequence included	226495975	226521040		-25065	0.00E+00		76	0	DEL	0.00E+00
4-620	4	226726443	226734186	NONE	Extra sequence included	226726443	226734186		-7743	0.00E+00		83	0	DEL	0.00E+00
4-622	4	226894951	226923907	NONE	Extra sequence included	226894951	226923907		-5471	7.57E-23		68	55	DEL	7.57E-23
4-637	4	232942447	233040659	NONE	Extra sequence included	232942447	233040659		-98212	0.00E+00		65	0	DEL	0.00E+00
4-643	4	234129213	234143499	NONE	Extra sequence included	234129213	234143499		-14286	0.00E+00		67	0	DEL	0.00E+00
4-646	4	234799208	234834794	NONE	Extra sequence included	234799208	234834794		-8290	7.57E-23		62	48	DEL	7.57E-23
5-25	5	5672707	5735821	NONE	Extra sequence included	5672707	5735821		-6734	7.45E-13		33	26	DEL	7.45E-13
5-30	5	8122386	8138303	NONE	Extra sequence included	8122386	8138303		-15917	0.00E+00		91	0	DEL	0.00E+00
5-36	5	9132223	9219221	NONE	Extra sequence included	9132223	9219221		-9716	7.30E-23		73	47	DEL	7.30E-23
5-38	5	9690238	9725049	NONE	Extra sequence included	9690238	9725049		-9398	7.30E-23		54	42	DEL	7.30E-23
5-43	5	10893751	11003756	NONE	Extra sequence included	10893751	11003756		-13220	6.50E-16		32	24	DEL	6.50E-16
5-52	5	18280030	18338721	NONE	Extra sequence included	18280030	18338721		-13276	7.30E-23		75	66	DEL	7.30E-23
5-92	5	39560020	39604608	NONE	Extra sequence included	39560020	39604608		-13627	7.30E-23		64	53	DEL	7.30E-23
5-95	5	40160267	40267767	NONE	Extra sequence included	40160267	40267767		-35172	7.30E-23		55	46	DEL	7.30E-23
5-107	5	44908423	44966680	NONE	Extra sequence included	44908423	44966680		-9247	7.30E-23		57	41	DEL	7.30E-23
5-112	5	48210163	48352109	NONE	Extra sequence included	48210163	48352109		-17317	1.47E-15		27	22	DEL	1.47E-15
5-124	5	52002961	52029740	NONE	Extra sequence included	52002961	52029740		-10977	7.30E-23		69	44	DEL	7.30E-23
5-146	5	57256916	57332953	NONE	Extra sequence included	57256916	57332953		-8578	7.30E-23		80	64	DEL	7.30E-23
5-156	5	64264720	64312996	NONE	Extra sequence included	64264720	64312996		-5965	7.30E-23		78	64	DEL	7.30E-23
5-158	5	64793637	64815825	NONE	Extra sequence included	64793637	64815825		-3345	1.03E-22		84	66	DEL	1.03E-22
5-206	5	79866283	79924121	NONE	Extra sequence included	79866283	79924121		-8624	7.30E-23		60	40	DEL	7.30E-23
5-230	5	88808524	88875742	NONE	Extra sequence included	88808524	88875742		-67218	0.00E+00		69	0	DEL	0.00E+00
5-240	5	91875062	91920020	NONE	Extra sequence included	91875062	91920020		-14791	7.30E-23		58	42	DEL	7.30E-23
5-244	5	92952622	92984479	NONE	Extra sequence included	92952622	92984479		-9201	7.30E-23		59	50	DEL	7.30E-23
5-256	5	97856408	97864623	NONE	Extra sequence included	97856408	97864623		-8215	0.00E+00		67	0	DEL	0.00E+00
5-260	5	98534604	98572929	NONE	Extra sequence included	98534604	98572929		-7134	7.30E-23		49	41	DEL	7.30E-23
5-268	5	101221874	101339277	NONE	Extra sequence included	101221874	101339277		-15883	1.53E-18		22	22	DEL	1.53E-18
5-289	5	110470059	110476679	NONE	Extra sequence included	110470059	110476679		-6620	0.00E+00		75	0	DEL	0.00E+00
5-296	5	113877909	113913157	NONE	Extra sequence included	113877909	113913157		-5081	2.67E-22		41	29	DEL	2.67E-22
5-298	5	114374703	114424299	NONE	Extra sequence included	114374703	114424299		-5854	7.30E-23		60	49	DEL	7.30E-23
5-299	5	115987450	116075541	NONE	Extra sequence included	115987450	116075541		-11346	3.59E-14		22	19	DEL	3.59E-14
5-311	5	120925352	120971970	NONE	Extra sequence included	120925352	120971970		-6991	7.30E-23		69	61	DEL	7.30E-23
5-312	5	121136142	121186331	NONE	Extra sequence included	121136142	121186331		-50189	0.00E+00		53	0	DEL	0.00E+00
5-316	5	122452896	122480109	NONE	Extra sequence included	122452896	122480109		-10396	7.30E-23		51	43	DEL	7.30E-23
5-323	5	124027334	124095335	NONE	Extra sequence included	124027334	124095335		-9503	7.30E-23		55	46	DEL	7.30E-23
5-328	5	124966463	125110055	NONE	Extra sequence included	124966463	125110055		-23884	1.47E-15		16	12	DEL	1.47E-15
5-334	5	126515455	126522048	NONE	Extra sequence included	126515455	126522048		-6593	0.00E+00		80	0	DEL	0.00E+00
5-340	5	131017671	131069181	NONE	Extra sequence included	131017671	131069181		-6725	7.30E-23		55	46	DEL	7.30E-23
5-347	5	132570980	132606150	NONE	Extra sequence included	132570980	132606150		-15016	7.30E-23		62	44	DEL	7.30E-23

5-367	5	137550378	137585694	NONE	Extra sequence included	137550378	137585694	-11453	7.30E-23	84	77	DEL	7.30E-23
5-371	5	138402187	138451703	NONE	Extra sequence included	138402187	138451703	-9511	7.30E-23	68	38	DEL	7.30E-23
5-376	5	139686946	139765156	NONE	Extra sequence included	139686946	139765156	-78210	0.00E+00	60	0	DEL	0.00E+00
5-379	5	140773897	140895631	NONE	Extra sequence included	140773897	140895631	-34548	7.30E-23	39	31	DEL	7.30E-23
5-383	5	142256355	142327988	NONE	Extra sequence included	142256355	142327988	-8051	7.30E-23	55	45	DEL	7.30E-23
5-394	5	146084542	146123854	NONE	Extra sequence included	146084542	146123854	-7204	7.30E-23	74	59	DEL	7.30E-23
5-397	5	146455707	146544613	NONE	Extra sequence included	146455707	146544613	-8912	1.31E-11	28	25	DEL	1.31E-11
5-417	5	157984027	158034865	NONE	Extra sequence included	157984027	158034865	-6401	5.69E-21	42	33	DEL	5.69E-21
5-423	5	159826753	159862202	NONE	Extra sequence included	159826753	159862202	-9421	7.30E-23	43	39	DEL	7.30E-23
5-425	5	162182330	162315372	NONE	Extra sequence included	162182330	162315372	-133042	0.00E+00	70	0	DEL	0.00E+00
5-435	5	168165372	168188709	NONE	Extra sequence included	168165372	168188709	-6570	7.30E-23	54	41	DEL	7.30E-23
5-465	5	186483536	186536776	NONE	Extra sequence included	186483536	186536776	-53240	0.00E+00	70	0	DEL	0.00E+00
5-474	5	194714559	194748438	NONE	Extra sequence included	194714559	194748438	-33879	0.00E+00	41	0	DEL	0.00E+00
5-477	5	195406850	195468676	NONE	Extra sequence included	195406850	195468676	-7242	7.30E-23	60	46	DEL	7.30E-23
5-482	5	197467086	197545396	NONE	Extra sequence included	197467086	197545396	-3698	3.53E-04	43	34	DEL	3.53E-04
5-493	5	208359826	208442938	NONE	Extra sequence included	208359826	208442938	-10357	7.30E-23	71	54	DEL	7.30E-23
6-3	6	3169017	3204589	NONE	Extra sequence included	3169017	3204589	-7479	8.15E-23	41	35	DEL	8.15E-23
6-17	6	19374816	19391907	NONE	Extra sequence included	19374816	19391907	-8314	8.15E-23	47	26	DEL	8.15E-23
6-50	6	37550355	37601775	NONE	Extra sequence included	37550355	37601775	-51420	0.00E+00	71	0	DEL	0.00E+00
6-51	6	37650109	37662409	NONE	Extra sequence included	37650109	37662409	-12300	0.00E+00	54	0	DEL	0.00E+00
6-62	6	40369965	40408306	NONE	Extra sequence included	40369965	40408306	-27167	8.15E-23	47	34	DEL	8.15E-23
6-68	6	42071313	42139141	NONE	Extra sequence included	42071313	42139141	-9651	8.15E-23	55	51	DEL	8.15E-23
6-76	6	51482804	51491009	NONE	Extra sequence included	51482804	51491009	-8205	0.00E+00	60	0	DEL	0.00E+00
6-84	6	53385421	53438141	NONE	Extra sequence included	53385421	53438141	-11509	8.15E-23	89	65	DEL	8.15E-23
6-94	6	56010278	56052782	NONE	Extra sequence included	56010278	56052782	-6624	8.15E-23	50	46	DEL	8.15E-23
6-98	6	56601175	56681612	NONE	Extra sequence included	56601175	56681612	-11519	8.15E-23	45	39	DEL	8.15E-23
6-106	6	61207315	61299462	NONE	Extra sequence included	61207315	61299462	-9453	8.15E-23	72	60	DEL	8.15E-23
6-130	6	66940833	66981092	NONE	Extra sequence included	66940833	66981092	-6631	8.15E-23	52	45	DEL	8.15E-23
6-131	6	67031453	67149994	NONE	Extra sequence included	67031453	67149994	-13613	8.15E-23	48	42	DEL	8.15E-23
6-135	6	69332099	69398826	NONE	Extra sequence included	69332099	69398826	-66727	0.00E+00	28	0	DEL	0.00E+00
6-137	6	69677739	69726630	NONE	Extra sequence included	69677739	69726630	-8331	8.15E-23	45	37	DEL	8.15E-23
6-142	6	73974378	74051735	NONE	Extra sequence included	73974378	74051735	-21463	8.15E-23	47	32	DEL	8.15E-23
6-150	6	77169509	77244851	NONE	Extra sequence included	77169509	77244851	-75342	0.00E+00	57	0	DEL	0.00E+00
6-154	6	78232988	78276376	NONE	Extra sequence included	78232988	78276376	-11656	8.15E-23	60	45	DEL	8.15E-23
6-159	6	79473612	79516683	NONE	Extra sequence included	79473612	79516683	-6470	8.15E-23	57	43	DEL	8.15E-23
6-160	6	79745532	79754768	NONE	Extra sequence included	79745532	79754768	-9236	0.00E+00	63	0	DEL	0.00E+00
6-168	6	88782801	88874984	NONE	Extra sequence included	88782801	88874984	-10142	1.09E-19	47	38	DEL	1.09E-19
6-169	6	90790135	90842446	NONE	Extra sequence included	90790135	90842446	-7923	6.79E-19	25	21	DEL	6.79E-19
7-2	7	5066063	5107900	NONE	Extra sequence included	5066063	5107900	-5175	7.74E-23	51	40	DEL	7.74E-23
7-20	7	9252534	9415511	NONE	Extra sequence included	9252534	9415511	-20963	2.85E-22	45	29	DEL	2.85E-22
7-30	7	15863715	15874945	NONE	Extra sequence included	15863715	15874945	-11230	0.00E+00	46	0	DEL	0.00E+00
7-39	7	22968753	23135760	NONE	Extra sequence included	22968753	23135760	-15027	9.89E-08	24	19	DEL	9.89E-08
7-44	7	28035665	28072795	NONE	Extra sequence included	28035665	28072795	-37130	0.00E+00	48	0	DEL	0.00E+00
7-61	7	38052874	38077063	NONE	Extra sequence included	38052874	38077063	-8987	7.74E-23	59	41	DEL	7.74E-23
7-70	7	40503980	40585010	NONE	Extra sequence included	40503980	40585010	-8404	7.74E-23	59	49	DEL	7.74E-23
7-79	7	44607034	44673508	NONE	Extra sequence included	44607034	44673508	-12533	7.74E-23	71	59	DEL	7.74E-23
7-80	7	44803826	44833203	NONE	Extra sequence included	44803826	44833203	-10656	7.74E-23	76	51	DEL	7.74E-23
7-81	7	44902911	44949462	NONE	Extra sequence included	44902911	44949462	-5087	2.28E-21	68	46	DEL	2.28E-21
7-96	7	48549010	48599673	NONE	Extra sequence included	48549010	48599673	-10771	7.74E-23	42	35	DEL	7.74E-23
7-110	7	56312200	56358983	NONE	Extra sequence included	56312200	56358983	-9789	7.74E-23	37	28	DEL	7.74E-23
7-111	7	56444478	56483790	NONE	Extra sequence included	56444478	56483790	-5667	7.74E-23	38	31	DEL	7.74E-23
7-113	7	56836477	56927616	NONE	Extra sequence included	56836477	56927616	-9534	8.72E-09	17	17	DEL	8.72E-09
7-114	7	59392845	59423487	NONE	Extra sequence included	59392845	59423487	-7790	7.74E-23	60	48	DEL	7.74E-23
7-125	7	61977968	62024692	NONE	Extra sequence included	61977968	62024692	-6905	7.74E-23	65	40	DEL	7.74E-23
7-140	7	66587023	66611950	NONE	Extra sequence included	66587023	66611950	-7455	7.74E-23	77	55	DEL	7.74E-23
7-146	7	68515388	68550956	NONE	Extra sequence included	68515388	68550956	-6138	7.74E-23	44	35	DEL	7.74E-23
7-147	7	68550956	68644265	NONE	Extra sequence included	68550956	68644265	-10149	7.90E-15	33	28	DEL	7.90E-15
7-177	7	78868863	78923039	NONE	Extra sequence included	78868863	78923039	-54176	0.00E+00	54	0	DEL	0.00E+00
7-214	7	95372385	95426848	NONE	Extra sequence included	95372385	95426848	-21198	7.74E-23	48	43	DEL	7.74E-23
7-221	7	99245004	99303961	NONE	Extra sequence included	99245004	99303961	-18884	7.74E-23	74	62	DEL	7.74E-23
7-241	7	110172859	110214414	NONE	Extra sequence included	110172859	110214414	-6575	7.74E-23	46	39	DEL	7.74E-23
7-243	7	110889373	110950677	NONE	Extra sequence included	110889373	110950677	-15627	7.74E-23	79	53	DEL	7.74E-23
7-246	7	113527789	113545596	NONE	Extra sequence included	113527789	113545596	-17807	0.00E+00	61	0	DEL	0.00E+00
7-253	7	115180722	115223694	NONE	Extra sequence included	115180722	115223694	-11615	7.74E-23	52	38	DEL	7.74E-23
7-259	7	119996010	120068957	NONE	Extra sequence included	119996010	120068957	-11018	7.74E-23	70	56	DEL	7.74E-23
7-264	7	125143683	125208603	NONE	Extra sequence included	125143683	125208603	-7101	7.74E-23	65	57	DEL	7.74E-23
7-283	7	138358118	138391461	NONE	Extra sequence included	138358118	138391461	-5078	7.74E-23	72	59	DEL	7.74E-23
7-285	7	138600439	138623278	NONE	Extra sequence included	138600439	138623278	-8051	7.74E-23	79	55	DEL	7.74E-23
7-292	7	142510052	142565622	NONE	Extra sequence included	142510052	142565622	-6023	2.57E-07	22	14	DEL	2.57E-07
7-302	7	153114866	153148042	NONE	Extra sequence included	153114866	153148042	-33176	0.00E+00	59	0	DEL	0.00E+00
7-303	7	153464778	153486248	NONE	Extra sequence included	153464778	153486248	-5855	1.08E-22	77	54	DEL	1.08E-22
7-305	7	155440514	155475982	NONE	Extra sequence included	155440514	155475982	-9321	7.74E-23	64	52	DEL	7.74E-23
8-3	8	2214680	2258445	NONE	Extra sequence included	2214680	2258445	-7214	6.97E-23	75	62	DEL	6.97E-23
8-4	8	4925229	4977004	NONE	Extra sequence included	4925229	4977004	-13471	6.97E-23	78	60	DEL	6.97E-23
8-11	8	5732525	5754809	NONE	Extra sequence included	5732525	5754809	-6993	6.97E-23	45	38	DEL	6.97E-23
8-16	8	8594390	8658073	NONE	Extra sequence included	8594390	8658073	-21412	6.97E-23	71	51	DEL	6.97E-23
8-30	8	11686853	11726167	NONE	Extra sequence included	11686853	11726167	-9070	6.97E-23	88	67	DEL	6.97E-23
8-45	8	15752896	15801697	NONE	Extra sequence included	15752896	15801697	-14060	6.97E-23	67	53	DEL	6.97E-23
8-46	8	16020368	16118402	NONE	Extra sequence included	16020368	16118402	-22143	6.97E-23	51	42	DEL	6.97E-23
8-57	8	19097115	19154218	NONE	Extra sequence included	19097115	19154218	-7367	6.97E-23	75	54	DEL	6.97E-23
8-58	8	19189483	19228443	NONE	Extra sequence included	19189483	19228443	-9379	6.97E-23	76	60	DEL	6.97E-23
8-64	8	21218002	21252113	NONE	Extra sequence included	21218002	21252113	-5383	6.97E-23	72	54	DEL	6.97E-23
8-73	8	24092623	24175642	NONE	Extra sequence included	24092623	24175642	-83019	0.00E+00	50	0	DEL	0.00E+00
8-78	8	25393023	25404141	NONE	Extra sequence included	25393023	25404141	-11118	0.00E+00	67	0	DEL	0.00E+00
8-103	8	33398982	33440261	NONE	Extra sequence included	33398982	33440261	-5748	6.97E-23	43	35	DEL	6.97E-23
8-104	8	34439982	34453987	NONE	Extra sequence included	34439982	34453987	-14005	0.00E+00	32	0	DEL	0.00E+00
8-109	8	35447185	35486353	NONE	Extra sequence included	35447185	35486353	-7544	6.97E-23	55	47	DEL	6.97E-23
8-119	8	38698189	38733597	NONE	Extra sequence included	38698189	38733597	-10225	6.97E-23	38	30	DEL	6.97E-23
8-124	8	39684206	39759279	NONE	Extra sequence included	39684206	39759279	-32408	8.64E-23	41	38	DEL	8.64E-23
8-125	8	39942559	40022705	NONE	Extra sequence included	39942559	40022705	-10428	6.97E-23	50	42	DEL	6.97E-23
8-134	8	42869480	42990085	NONE	Extra sequence included	42869480	42990085	-30292	6.97E-23	40	34	DEL	6.97E-23

8-135	8	43127642	43172315	NONE	Extra sequence included	43127642	43172315		-9475	6.97E-23		49	40	DEL	6.97E-23	
8-142	8	44720714	44785444	NONE	Extra sequence included	44720714	44785444		-14347	6.97E-23		77	66	DEL	6.97E-23	
8-151	8	51910445	51961870	NONE	Extra sequence included	51910445	51961870		-7627	6.97E-23		46	33	DEL	6.97E-23	
8-157	8	54694379	54784919	NONE	Extra sequence included	54694379	54784919		-21948	6.97E-23		49	37	DEL	6.97E-23	
8-159	8	55329739	55342916	NONE	Extra sequence included	55329739	55342916		-13177	0.00E+00		82	0	DEL	0.00E+00	
8-161	8	55589607	55659443	NONE	Extra sequence included	55589607	55659443		-8720	6.97E-23		66	57	DEL	6.97E-23	
8-180	8	63996531	64053585	NONE	Extra sequence included	63996531	64053585		-12751	6.97E-23		67	51	DEL	6.97E-23	
8-181	8	64117297	64144781	NONE	Extra sequence included	64117297	64144781		-6469	6.97E-23		74	53	DEL	6.97E-23	
8-198	8	70132032	70182527	NONE	Extra sequence included	70132032	70182527		-14103	6.97E-23		55	36	DEL	6.97E-23	
8-210	8	74501377	74558184	NONE	Extra sequence included	74501377	74558184		-12643	6.97E-23		63	47	DEL	6.97E-23	
8-221	8	77016090	77109355	NONE	Extra sequence included	77016090	77109355		-18990	6.97E-23		66	52	DEL	6.97E-23	
8-233	8	82394738	82467628	NONE	Extra sequence included	82394738	82467628		-9760	6.97E-23		44	38	DEL	6.97E-23	
8-242	8	89700369	89736422	NONE	Extra sequence included	89700369	89736422		-8020	6.97E-23		55	42	DEL	6.97E-23	
8-262	8	110387173	110442793	NONE	Extra sequence included	110387173	110442793		-6542	6.19E-16		43	29	DEL	6.19E-16	
8-270	8	113569516	113658009	NONE	Extra sequence included	113569516	113658009		-3849	4.49E-05		63	54	DEL	4.49E-05	
8-298	8	129537776	129624221	NONE	Extra sequence included	129537776	129624221		-11215	6.97E-23		62	53	DEL	6.97E-23	
8-303	8	130663852	130727558	NONE	Extra sequence included	130663852	130727558		-12629	6.97E-23		54	38	DEL	6.97E-23	
8-308	8	132920645	132968777	NONE	Extra sequence included	132920645	132968777		-6154	6.97E-23		55	44	DEL	6.97E-23	
8-324	8	136815989	136842012	NONE	Extra sequence included	136815989	136842012		-5122	6.97E-23		55	44	DEL	6.97E-23	
8-328	8	144497787	144535234	NONE	Extra sequence included	144497787	144535234		-37447	0.00E+00		74	0	DEL	0.00E+00	
8-337	8	148720708	148756448	NONE	Extra sequence included	148720708	148756448		-10636	6.97E-23		79	60	DEL	6.97E-23	
8-345	8	150034643	150088214	NONE	Extra sequence included	150034643	150088214		-8742	6.97E-23		58	32	DEL	6.97E-23	
8-377	8	171334485	171396553	NONE	Extra sequence included	171334485	171396553		-7124	6.97E-23		67	52	DEL	6.97E-23	
8-378	8	171462838	171509498	NONE	Extra sequence included	171462838	171509498		-16861	8.28E-23		71	53	DEL	8.28E-23	
9-15	9	15846768	15946657	NONE	Extra sequence included	15846768	15946657		-17140	7.33E-23		43	36	DEL	7.33E-23	
9-17	9	16497026	16555392	NONE	Extra sequence included	16497026	16555392		-8719	7.33E-23		74	64	DEL	7.33E-23	
9-29	9	21769530	21785847	NONE	Extra sequence included	21769530	21785847		-16317	0.00E+00		83	0	DEL	0.00E+00	
9-33	9	25033568	25067364	NONE	Extra sequence included	25033568	25067364		-6081	7.33E-23		55	46	DEL	7.33E-23	
9-42	9	30656447	30748767	NONE	Extra sequence included	30656447	30748767		-17727	7.33E-23		35	27	DEL	7.33E-23	
9-47	9	33176031	33216289	NONE	Extra sequence included	33176031	33216289		-7823	7.33E-23		76	52	DEL	7.33E-23	
9-60	9	39384704	39427644	NONE	Extra sequence included	39384704	39427644		-7482	7.33E-23		62	51	DEL	7.33E-23	
9-84	9	48543349	48627917	NONE	Extra sequence included	48543349	48627917		-10079	1.22E-16		34	27	DEL	1.22E-16	
9-88	9	49671866	49735525	NONE	Extra sequence included	49671866	49735525		-8119	1.54E-12		22	18	DEL	1.54E-12	
9-96	9	53321018	53339567	NONE	Extra sequence included	53321018	53339567		-5183	7.33E-23		38	28	DEL	7.33E-23	
9-120	9	64948265	65016516	NONE	Extra sequence included	64948265	65016516		-16093	7.33E-23		48	42	DEL	7.33E-23	
9-129	9	67376585	67426091	NONE	Extra sequence included	67376585	67426091		-49506	0.00E+00		83	0	DEL	0.00E+00	
9-135	9	69974322	70025720	NONE	Extra sequence included	69974322	70025720		-16067	7.33E-23		60	45	DEL	7.33E-23	
9-140	9	70904868	70955423	NONE	Extra sequence included	70904868	70955423		-15244	7.33E-23		61	54	DEL	7.33E-23	
9-145	9	72036960	72081538	NONE	Extra sequence included	72036960	72081538		-5685	9.10E-18		30	28	DEL	9.10E-18	
9-150	9	73650882	73714333	NONE	Extra sequence included	73650882	73714333		-6649	2.69E-22		68	49	DEL	2.69E-22	
9-167	9	80132348	80172031	NONE	Extra sequence included	80132348	80172031		-5804	7.33E-23		85	56	DEL	7.33E-23	
9-210	9	92556675	92620389	NONE	Extra sequence included	92556675	92620389		-63714	0.00E+00		34	0	DEL	0.00E+00	
9-216	9	94489696	94506556	NONE	Extra sequence included	94489696	94506556		-5699	7.33E-23		84	55	DEL	7.33E-23	
9-220	9	95263117	95324452	NONE	Extra sequence included	95263117	95324452		-22943	7.33E-23		38	35	DEL	7.33E-23	
9-229	9	96948739	97015776	NONE	Extra sequence included	96948739	97015776		-27367	7.33E-23		42	26	DEL	7.33E-23	
9-244	9	102065952	102103790	NONE	Extra sequence included	102065952	102103790		-9874	7.33E-23		74	54	DEL	7.33E-23	
9-251	9	106543520	106605881	NONE	Extra sequence included	106543520	106605881		-8900	5.77E-21		34	25	DEL	5.77E-21	
9-260	9	113863209	113896338	NONE	Extra sequence included	113863209	113896338		-6704	7.33E-23		79	67	DEL	7.33E-23	
9-262	9	114174667	114201577	NONE	Extra sequence included	114174667	114201577		-26910	0.00E+00		57	0	DEL	0.00E+00	
9-265	9	114652618	114686788	NONE	Extra sequence included	114652618	114686788		-5008	7.33E-23		50	46	DEL	7.33E-23	
9-266	9	114760005	114882737	NONE	Extra sequence included	114760005	114882737		-30826	7.33E-23		58	39	DEL	7.33E-23	
9-280	9	125296218	125359010	NONE	Extra sequence included	125296218	125359010		-9230	1.51E-20		25	23	DEL	1.51E-20	
9-283	9	132641295	132650262	NONE	Extra sequence included	132641295	132650262		-8967	0.00E+00		45	0	DEL	0.00E+00	
9-284	9	132692502	132755774	NONE	Extra sequence included	132692502	132755774		-6616	1.53E-18		58	41	DEL	1.53E-18	
9-289	9	134075976	134152192	NONE	Extra sequence included	134075976	134152192		-8170	2.85E-16		42	33	DEL	2.85E-16	
9-291	9	134543773	134565752	NONE	Extra sequence included	134543773	134565752		-6117	7.33E-23		81	56	DEL	7.33E-23	
9-331	9	144707940	144750603	NONE	Extra sequence included	144707940	144750603		-7838	7.33E-23		40	31	DEL	7.33E-23	
9-332	9	144955904	145063090	NONE	Extra sequence included	144955904	145063090		-54305	1.01E-22		33	25	DEL	1.01E-22	
9-339	9	146642504	146664093	NONE	Extra sequence included	146642504	146664093		-8416	7.33E-23		100	73	DEL	7.33E-23	
9-341	9	147234558	147281402	NONE	Extra sequence included	147234558	147281402		-46844	0.00E+00		72	0	DEL	0.00E+00	
10-4	10	658950	753273	NONE	Extra sequence included	658950	753273		-10060	7.28E-23		76	64	DEL	7.28E-23	
10-5	10	866207	927726	NONE	Extra sequence included	866207	927726		-4116	1.02E-10		77	55	DEL	1.02E-10	
10-17	10	8197577	8270229	NONE	Extra sequence included	8197577	8270229		-6942	8.93E-18		57	45	DEL	8.93E-18	
10-32	10	15573477	15598922	NONE	Extra sequence included	15573477	15598922		-3360	7.28E-23		64	56	DEL	7.28E-23	
10-34	10	16362848	16455985	NONE	Extra sequence included	16362848	16455985		-10047	7.45E-15		36	28	DEL	7.45E-15	
10-50	10	23284697	23342840	NONE	Extra sequence included	23284697	23342840		-11167	7.28E-23		81	60	DEL	7.28E-23	
10-52	10	24100848	24166963	NONE	Extra sequence included	24100848	24166963		-9851	7.28E-23		43	37	DEL	7.28E-23	
10-63	10	30095825	30132260	NONE	Extra sequence included	30095825	30132260		-12652	7.28E-23		64	44	DEL	7.28E-23	
10-64	10	30170129	30229807	NONE	Extra sequence included	30170129	30229807		-6203	2.13E-21		71	48	DEL	2.13E-21	
10-84	10	42271351	42316234	NONE	Extra sequence included	42271351	42316234		-9800	7.28E-23		58	48	DEL	7.28E-23	
10-93	10	46663287	46731557	NONE	Extra sequence included	46663287	46731557		-17649	7.28E-23		54	42	DEL	7.28E-23	
10-97	10	47490072	47530738	NONE	Extra sequence included	47490072	47530738		-12577	7.28E-23		46	43	DEL	7.28E-23	
10-104	10	52987310	53091834	NONE	Extra sequence included	52987310	53091834		-10638	5.08E-17		49	36	DEL	5.08E-17	
10-126	10	60943134	60972987	NONE	Extra sequence included	60943134	60972987		-5577	7.28E-23		72	62	DEL	7.28E-23	
10-136	10	62461281	62501300	NONE	Extra sequence included	62461281	62501300		-6436	7.28E-23		43	39	DEL	7.28E-23	
10-138	10	67632714	67658555	NONE	Extra sequence included	67632714	67658555		-5021	7.28E-23		67	50	DEL	7.28E-23	
10-142	10	69086786	69143924	NONE	Extra sequence included	69086786	69143924		-57138	0.00E+00		47	0	DEL	0.00E+00	
10-143	10	69211729	69252028	NONE	Extra sequence included	69211729	69252028		-40299	0.00E+00		43	0	DEL	0.00E+00	
10-145	10	69561317	69618634	NONE	Extra sequence included	69561317	69618634		-9769	7.28E-23		63	51	DEL	7.28E-23	
10-147	10	69864526	69955713	NONE	Extra sequence included	69864526	69955713		-91187	0.00E+00		73	0	DEL	0.00E+00	
10-148	10	71236328	71259018	NONE	Extra sequence included	71236328	71259018		-22690	0.00E+00		36	0	DEL	0.00E+00	
10-158	10	74008389	74049554	NONE	Extra sequence included	74008389	74049554		-13482	7.28E-23		39	31	DEL	7.28E-23	
10-161	10	74785473	74892003	NONE	Extra sequence included	74785473	74892003		-14702	7.28E-23		39	30	DEL	7.28E-23	
10-174	10	77379070	77395484	NONE	Extra sequence included	77379070	77395484		-16414	0.00E+00		65	0	DEL	0.00E+00	
10-178	10	82274168	82296602	NONE	Extra sequence included	82274168	82296602		-22434	0.00E+00		45	0	DEL	0.00E+00	
10-186	10	84302891	84392793	NONE	Extra sequence included	84302891	84392793		-18661	5.66E-21		13	11	DEL	5.66E-21	
10-191	10	86638150	86648215	NONE	Extra sequence included	86638150	86648215		-10065	0.00E+00		71	0	DEL	0.00E+00	
10-194	10	87011945	87055320	NONE	Extra sequence included	87011945	87055320		-7758	7.28E-23		50	40	DEL	7.28E-23	
10-202	10	92785500	92831155	NONE	Extra sequence included	92785500	92831155		-14977	7.28E-23		50	43	DEL	7.28E-23	
10-207	10	97775874	97844270	NONE	Extra sequence included	97775874	97844270		-15901	7.28E-23		55	41	DEL	7.28E-23	
10-217	10	103274962	103368552	NONE	Extra sequence included	103274962	103368552		-93590	0.00E+00		51	0	DEL	0.00E+00	

10-226	10	105223593	105361720	NONE	Extra sequence included	105223593	105361720		-138127	0.00E+00		64	0	DEL	0.00E+00	
10-231	10	106201067	106269597	NONE	Extra sequence included	106201067	106269597		-10315	7.28E-23		59	53	DEL	7.28E-23	
10-235	10	107197035	107248439	NONE	Extra sequence included	107197035	107248439		-7618	7.28E-23		58	47	DEL	7.28E-23	
10-241	10	108279134	108325960	NONE	Extra sequence included	108279134	108325960		-5784	7.28E-23		76	62	DEL	7.28E-23	
10-255	10	110626614	110700923	NONE	Extra sequence included	110626614	110700923		-74309	0.00E+00		53	0	DEL	0.00E+00	
10-258	10	111188659	111227540	NONE	Extra sequence included	111188659	111227540		-5177	7.28E-23		67	45	DEL	7.28E-23	
10-260	10	112158697	112166983	NONE	Extra sequence included	112158697	112166983		-8286	0.00E+00		71	0	DEL	0.00E+00	
10-265	10	112825848	112865206	NONE	Extra sequence included	112825848	112865206		-39358	0.00E+00		52	0	DEL	0.00E+00	
10-270	10	113464570	113553730	NONE	Extra sequence included	113464570	113553730		-19280	7.28E-23		42	37	DEL	7.28E-23	
10-279	10	115265370	115308547	NONE	Extra sequence included	115265370	115308547		-6109	7.28E-23		42	36	DEL	7.28E-23	
10-283	10	116494093	116568118	NONE	Extra sequence included	116494093	116568118		-19631	7.28E-23		64	48	DEL	7.28E-23	
10-289	10	117344108	117366880	NONE	Extra sequence included	117344108	117366880		-6005	7.28E-23		74	61	DEL	7.28E-23	
10-312	10	134335382	134371480	NONE	Extra sequence included	134335382	134371480		-8707	7.28E-23		75	60	DEL	7.28E-23	
10-313	10	134371480	134449091	NONE	Extra sequence included	134371480	134449091		-12682	7.28E-23		67	46	DEL	7.28E-23	
10-321	10	142125137	142238436	NONE	Extra sequence included	142125137	142238436		-27678	7.28E-23		50	41	DEL	7.28E-23	
10-324	10	142628463	142692663	NONE	Extra sequence included	142628463	142692663		-7833	7.28E-23		60	43	DEL	7.28E-23	
10-325	10	142692663	142750612	NONE	Extra sequence included	142692663	142750612		-9677	7.28E-23		59	41	DEL	7.28E-23	
1-2	1	2127550	2150858	NONE	gap called	2127550	2150858		7711	7.12E-23		59	49	INS	7.12E-23	
1-5	1	2368965	2428240	NONE	gap called	2368965	2428240		12242	7.12E-23		49	39	INS	7.12E-23	
1-14	1	7852044	7863623	NONE	gap called	7852044	7863623		5779	7.12E-23		77	63	INS	7.12E-23	
1-19	1	8559920	8592822	NONE	gap called	8559920	8592822		10358	1.01E-22		51	46	INS	1.01E-22	
1-28	1	9773176	9773176	NONE	gap called	9773176	9773176		63258	0.00E+00		80	0	INS	0.00E+00	
1-29	1	10081846	10159094	NONE	gap called	10081846	10159094		40641	1.01E-22		24	21	INS	1.01E-22	
1-39	1	12627003	12665882	NONE	gap called	12627003	12665882		12374	7.12E-23		78	58	INS	7.12E-23	
1-42	1	12813348	12813348	NONE	gap called	12813348	12813348		7569	0.00E+00		80	0	INS	0.00E+00	
1-45	1	13365763	13365763	NONE	gap called	13365763	13365763		9461	0.00E+00		82	0	INS	0.00E+00	
1-53	1	14299303	14335213	NONE	gap called	14299303	14335213		16153	7.12E-23		51	38	INS	7.12E-23	
1-55	1	14795505	14795505	NONE	gap called	14795505	14795505		25116	0.00E+00		75	0	INS	0.00E+00	
1-61	1	16161846	16161846	NONE	gap called	16161846	16161846		101201	0.00E+00		60	0	INS	0.00E+00	
1-69	1	17799103	17832772	NONE	gap called	17799103	17832772		11666	7.12E-23		50	41	INS	7.12E-23	
1-73	1	18314417	18412200	NONE	gap called	18314417	18412200		9928	5.01E-17		57	37	INS	5.01E-17	
1-76	1	22622291	22673519	NONE	gap called	22622291	22673519		9392	7.12E-23		54	38	INS	7.12E-23	
1-77	1	22961218	22961218	NONE	gap called	22961218	22961218		10088	0.00E+00		82	0	INS	0.00E+00	
1-80	1	24418086	24439618	NONE	gap called	24418086	24439618		5253	7.12E-23		35	30	INS	7.12E-23	
1-84	1	29144446	29287716	NONE	gap called	29144446	29287716		38552	7.12E-23		45	37	INS	7.12E-23	
1-85	1	29372421	29410874	NONE	gap called	29372421	29410874		7736	7.12E-23		61	48	INS	7.12E-23	
1-104	1	36821566	36926760	NONE	gap called	36821566	36926760		10260	1.34E-11		34	26	INS	1.34E-11	
1-105	1	37177784	37220461	NONE	gap called	37177784	37220461		8525	7.12E-23		61	47	INS	7.12E-23	
1-116	1	45976929	46016506	NONE	gap called	45976929	46016506		8814	7.12E-23		68	53	INS	7.12E-23	
1-122	1	50718245	50718245	NONE	gap called	50718245	50718245		30507	0.00E+00		11	0	INS	0.00E+00	
1-124	1	51075847	51095669	NONE	gap called	51075847	51095669		7098	7.12E-23		66	45	INS	7.12E-23	
1-125	1	53182084	53236306	NONE	gap called	53182084	53236306		8581	1.03E-10		13	10	INS	1.03E-10	
1-126	1	53520560	53570337	NONE	gap called	53520560	53570337		7991	7.12E-23		36	28	INS	7.12E-23	
1-127	1	53664451	53690669	NONE	gap called	53664451	53690669		11183	7.12E-23		50	43	INS	7.12E-23	
1-140	1	57127114	57167809	NONE	gap called	57127114	57167809		6889	7.12E-23		56	45	INS	7.12E-23	
1-144	1	57457916	57533025	NONE	gap called	57457916	57533025		35162	7.12E-23		45	35	INS	7.12E-23	
1-146	1	57674694	57674694	NONE	gap called	57674694	57674694		17173	0.00E+00		55	0	INS	0.00E+00	
1-153	1	58493533	58521345	NONE	gap called	58493533	58521345		8473	7.12E-23		65	37	INS	7.12E-23	
1-157	1	59597233	59597233	NONE	gap called	59597233	59597233		27185	0.00E+00		78	0	INS	0.00E+00	
1-158	1	59668818	59727268	NONE	gap called	59668818	59727268		11732	7.12E-23		70	52	INS	7.12E-23	
1-165	1	61359117	61402287	NONE	gap called	61359117	61402287		7087	7.12E-23		71	52	INS	7.12E-23	
1-174	1	63523347	63580091	NONE	gap called	63523347	63580091		9618	7.12E-23		44	34	INS	7.12E-23	
1-178	1	63956066	63956066	NONE	gap called	63956066	63956066		55212	0.00E+00		66	0	INS	0.00E+00	
1-183	1	66118230	66249066	NONE	gap called	66118230	66249066		16397	2.11E-21		37	30	INS	2.11E-21	
1-194	1	72294370	72352466	NONE	gap called	72294370	72352466		5932	1.45E-15		44	36	INS	1.45E-15	
1-204	1	74702576	74749358	NONE	gap called	74702576	74749358		12150	7.12E-23		68	57	INS	7.12E-23	
1-205	1	74926103	74926103	NONE	gap called	74926103	74926103		69391	0.00E+00		69	0	INS	0.00E+00	
1-207	1	75116993	75116993	NONE	gap called	75116993	75116993		5389	0.00E+00		73	0	INS	0.00E+00	
1-216	1	79356615	79412644	NONE	gap called	79356615	79412644		12179	7.12E-23		52	45	INS	7.12E-23	
1-217	1	79555468	79592996	NONE	gap called	79555468	79592996		6890	7.12E-23		83	58	INS	7.12E-23	
1-222	1	80429223	80505694	NONE	gap called	80429223	80505694		8039	7.12E-23		57	54	INS	7.12E-23	
1-224	1	80716675	80716675	NONE	gap called	80716675	80716675		9565	0.00E+00		76	0	INS	0.00E+00	
1-233	1	83105240	83143571	NONE	gap called	83105240	83143571		9562	7.12E-23		61	40	INS	7.12E-23	
1-234	1	83837818	83932902	NONE	gap called	83837818	83932902		31933	7.12E-23		17	13	INS	7.12E-23	
1-238	1	84873096	84873096	NONE	gap called	84873096	84873096		9118	0.00E+00		64	0	INS	0.00E+00	
1-244	1	87960847	87977323	NONE	gap called	87960847	87977323		3073	1.01E-22		52	37	INS	1.01E-22	
1-246	1	88087008	88099483	NONE	gap called	88087008	88099483		5499	1.00E-22		49	28	INS	1.00E-22	
1-248	1	88373351	88435297	NONE	gap called	88373351	88435297		33205	1.01E-22		43	37	INS	1.01E-22	
1-254	1	89469855	89514490	NONE	gap called	89469855	89514490		6542	7.12E-23		52	42	INS	7.12E-23	
1-255	1	90480202	90529468	NONE	gap called	90480202	90529468		9462	7.12E-23		60	48	INS	7.12E-23	
1-256	1	90549973	90585948	NONE	gap called	90549973	90585948		9162	7.12E-23		63	51	INS	7.12E-23	
1-273	1	95814206	95875712	NONE	gap called	95814206	95875712		16273	7.12E-23		60	53	INS	7.12E-23	
1-276	1	96315476	96345144	NONE	gap called	96315476	96345144		5706	7.12E-23		52	42	INS	7.12E-23	
1-283	1	100690178	100690178	NONE	gap called	100690178	100690178		257032	0.00E+00		27	0	INS	0.00E+00	
1-291	1	102233913	102256712	NONE	gap called	102233913	102256712		3995	1.01E-22		48	30	INS	1.01E-22	
1-294	1	102824104	102824104	NONE	gap called	102824104	102824104		42020	0.00E+00		22	0	INS	0.00E+00	
1-296	1	103151823	103201669	NONE	gap called	103151823	103201669		6498	6.33E-16		30	23	INS	6.33E-16	
1-299	1	103940872	103956789	NONE	gap called	103940872	103956789		5320	7.12E-23		69	55	INS	7.12E-23	
1-304	1	104444554	104472495	NONE	gap called	104444554	104472495		10404	7.12E-23		74	58	INS	7.12E-23	
1-306	1	104763540	104763540	NONE	gap called	104763540	104763540		32732	0.00E+00		66	0	INS	0.00E+00	
1-307	1	104980774	104980774	NONE	gap called	104980774	104980774		24008	0.00E+00		68	0	INS	0.00E+00	
1-309	1	105291835	105341020	NONE	gap called	105291835	105341020		11632	7.12E-23		65	51	INS	7.12E-23	
1-316	1	107622378	107662195	NONE	gap called	107622378	107662195		19924	9.91E-23		43	30	INS	9.91E-23	
1-317	1	107922732	107974181	NONE	gap called	107922732	107974181		9542	7.12E-23		44	32	INS	7.12E-23	
1-320	1	108341497	108414119	NONE	gap called	108341497	108414119		12200	7.12E-23		42	28	INS	7.12E-23	
1-327	1	110202699	110235308	NONE	gap called	110202699	110235308		12441	7.12E-23		67	57	INS	7.12E-23	
1-329	1	110367913	110383253	NONE	gap called	110367913	110383253		5031	7.12E-23		73	61	INS	7.12E-23	
1-332	1	110697017	110731035	NONE	gap called	110697017	110731035		6299	7.12E-23		46	29	INS	7.12E-23	
1-336	1	111589465	111624850	NONE	gap called	111589465	111624850		5167	7.12E-23		61	52	INS	7.12E-23	
1-337	1	111695148	111730670	NONE	gap called	111695148	111730670		5880	7.12E-23		60	51	INS	7.12E-23	
1-340	1	112003813	112023034	NONE	gap called	112003813	112023034		5818	7.12E-23		73	47	INS	7.12E-23	

1-343	1	112616798	112616798	NONE	gap called	112616798	112616798	11674	0.00E+00	50	0	INS	0.00E+00
1-347	1	113358313	113358313	NONE	gap called	113358313	113358313	18349	0.00E+00	72	0	INS	0.00E+00
1-355	1	115196186	115196186	NONE	gap called	115196186	115196186	13969	0.00E+00	52	0	INS	0.00E+00
1-360	1	118198772	118238494	NONE	gap called	118198772	118238494	5664	7.12E-23	48	38	INS	7.12E-23
1-365	1	119391642	119409093	NONE	gap called	119391642	119409093	11284	7.12E-23	58	46	INS	7.12E-23
1-379	1	123834043	123834043	NONE	gap called	123834043	123834043	168300	0.00E+00	16	0	INS	0.00E+00
1-390	1	126853583	126879224	NONE	gap called	126853583	126879224	9483	7.12E-23	68	54	INS	7.12E-23
1-392	1	127383203	127407845	NONE	gap called	127383203	127407845	6440	7.12E-23	65	57	INS	7.12E-23
1-403	1	131182847	131209783	NONE	gap called	131182847	131209783	12915	9.96E-23	42	34	INS	9.96E-23
1-412	1	137438739	137478136	NONE	gap called	137438739	137478136	8160	7.12E-23	87	72	INS	7.12E-23
1-421	1	140963258	141017200	NONE	gap called	140963258	141017200	23981	7.12E-23	33	19	INS	7.12E-23
1-423	1	141507622	141626496	NONE	gap called	141507622	141626496	12071	1.58E-08	21	17	INS	1.58E-08
1-427	1	142585500	142585500	NONE	gap called	142585500	142585500	31378	0.00E+00	24	0	INS	0.00E+00
1-431	1	143762999	143792310	NONE	gap called	143762999	143792310	8002	7.12E-23	69	50	INS	7.12E-23
1-442	1	149527244	149568138	NONE	gap called	149527244	149568138	9514	7.12E-23	39	30	INS	7.12E-23
1-448	1	154029068	154029068	NONE	gap called	154029068	154029068	11274	0.00E+00	80	0	INS	0.00E+00
1-458	1	156464922	156515724	NONE	gap called	156464922	156515724	5223	3.77E-20	62	47	INS	3.77E-20
1-462	1	157504232	157590088	NONE	gap called	157504232	157590088	9796	8.84E-18	39	31	INS	8.84E-18
1-469	1	158846041	158891182	NONE	gap called	158846041	158891182	11898	7.12E-23	45	31	INS	7.12E-23
1-474	1	160113336	160148371	NONE	gap called	160113336	160148371	11583	7.12E-23	28	21	INS	7.12E-23
1-482	1	162064054	162064054	NONE	gap called	162064054	162064054	74623	0.00E+00	58	0	INS	0.00E+00
1-483	1	162576488	162601319	NONE	gap called	162576488	162601319	5051	7.12E-23	62	52	INS	7.12E-23
1-484	1	162733125	162754943	NONE	gap called	162733125	162754943	10881	7.12E-23	63	56	INS	7.12E-23
1-486	1	163093992	163093992	NONE	gap called	163093992	163093992	15336	0.00E+00	59	0	INS	0.00E+00
1-487	1	163148025	163176365	NONE	gap called	163148025	163176365	6879	7.12E-23	56	48	INS	7.12E-23
1-490	1	163825038	163838892	NONE	gap called	163825038	163838892	5657	7.12E-23	58	42	INS	7.12E-23
1-494	1	164899508	164922267	NONE	gap called	164899508	164922267	5293	7.12E-23	52	47	INS	7.12E-23
1-498	1	165823492	165866407	NONE	gap called	165823492	165866407	5844	7.12E-23	92	79	INS	7.12E-23
1-509	1	168248220	168248220	NONE	gap called	168248220	168248220	16307	0.00E+00	53	0	INS	0.00E+00
1-516	1	171039901	171088492	NONE	gap called	171039901	171088492	14487	7.12E-23	32	23	INS	7.12E-23
1-524	1	172735345	172805770	NONE	gap called	172735345	172805770	15606	7.12E-23	27	17	INS	7.12E-23
1-547	1	183325428	183337507	NONE	gap called	183325428	183337507	5981	7.12E-23	65	48	INS	7.12E-23
1-548	1	183618165	183663946	NONE	gap called	183618165	183663946	19664	9.98E-23	48	38	INS	9.98E-23
1-549	1	183903350	183903350	NONE	gap called	183903350	183903350	31060	0.00E+00	55	0	INS	0.00E+00
1-550	1	184036065	184083536	NONE	gap called	184036065	184083536	18715	9.76E-23	37	29	INS	9.76E-23
1-551	1	184203771	184368798	NONE	gap called	184203771	184368798	21319	1.18E-16	31	21	INS	1.18E-16
1-565	1	192428206	192428206	NONE	gap called	192428206	192428206	68970	0.00E+00	69	0	INS	0.00E+00
1-583	1	196564089	196608353	NONE	gap called	196564089	196608353	7937	7.12E-23	54	39	INS	7.12E-23
1-587	1	197229894	197261222	NONE	gap called	197229894	197261222	7209	7.12E-23	66	45	INS	7.12E-23
1-601	1	205261494	205261494	NONE	gap called	205261494	205261494	31979	0.00E+00	80	0	INS	0.00E+00
1-603	1	205629018	205661818	NONE	gap called	205629018	205661818	9226	7.12E-23	61	49	INS	7.12E-23
1-606	1	206124781	206143687	NONE	gap called	206124781	206143687	10773	1.01E-22	61	48	INS	1.01E-22
1-609	1	206910626	206964929	NONE	gap called	206910626	206964929	29365	9.90E-23	46	39	INS	9.90E-23
1-612	1	207346383	207346383	NONE	gap called	207346383	207346383	103659	0.00E+00	58	0	INS	0.00E+00
1-613	1	207641822	207697133	NONE	gap called	207641822	207697133	6955	7.12E-23	61	51	INS	7.12E-23
1-624	1	211292852	211292852	NONE	gap called	211292852	211292852	45119	0.00E+00	89	0	INS	0.00E+00
1-634	1	212964989	213013505	NONE	gap called	212964989	213013505	9300	7.12E-23	58	40	INS	7.12E-23
1-654	1	220224113	220253746	NONE	gap called	220224113	220253746	16487	7.12E-23	50	41	INS	7.12E-23
1-658	1	221408757	221504661	NONE	gap called	221408757	221504661	12169	7.12E-23	36	32	INS	7.12E-23
1-660	1	223183878	223204785	NONE	gap called	223183878	223204785	5152	7.12E-23	38	36	INS	7.12E-23
1-661	1	223915949	223915949	NONE	gap called	223915949	223915949	14451	0.00E+00	78	0	INS	0.00E+00
1-670	1	226244745	226264939	NONE	gap called	226244745	226264939	5440	7.12E-23	71	63	INS	7.12E-23
1-673	1	226904235	226904235	NONE	gap called	226904235	226904235	6060	0.00E+00	45	0	INS	0.00E+00
1-674	1	227212020	227250340	NONE	gap called	227212020	227250340	9442	7.12E-23	61	54	INS	7.12E-23
1-678	1	228802476	228858477	NONE	gap called	228802476	228858477	5612	3.28E-15	39	36	INS	3.28E-15
1-679	1	228940813	228999275	NONE	gap called	228940813	228999275	8714	7.12E-23	39	28	INS	7.12E-23
1-682	1	229922943	230042613	NONE	gap called	229922943	230042613	20957	7.12E-23	31	24	INS	7.12E-23
1-684	1	230444924	230531937	NONE	gap called	230444924	230531937	9896	7.12E-23	67	58	INS	7.12E-23
1-700	1	234760579	234790137	NONE	gap called	234760579	234790137	11015	7.12E-23	60	47	INS	7.12E-23
1-702	1	235264240	235287657	NONE	gap called	235264240	235287657	7201	7.12E-23	34	23	INS	7.12E-23
1-706	1	237874216	237912790	NONE	gap called	237874216	237912790	6313	7.12E-23	52	37	INS	7.12E-23
1-707	1	238073739	238100574	NONE	gap called	238073739	238100574	5100	7.12E-23	65	54	INS	7.12E-23
1-713	1	242633254	242668396	NONE	gap called	242633254	242668396	6807	7.12E-23	57	50	INS	7.12E-23
1-718	1	246097490	246097490	NONE	gap called	246097490	246097490	356073	0.00E+00	13	0	INS	0.00E+00
1-719	1	246552665	246583133	NONE	gap called	246552665	246583133	18564	7.12E-23	71	58	INS	7.12E-23
1-720	1	246591806	246610619	NONE	gap called	246591806	246610619	5246	7.12E-23	82	67	INS	7.12E-23
1-724	1	247092254	247121596	NONE	gap called	247092254	247121596	5800	7.12E-23	91	72	INS	7.12E-23
1-741	1	255067057	255094248	NONE	gap called	255067057	255094248	6635	7.12E-23	66	53	INS	7.12E-23
1-751	1	261692893	261750585	NONE	gap called	261692893	261750585	6187	7.12E-23	72	55	INS	7.12E-23
1-754	1	265386285	265386285	NONE	gap called	265386285	265386285	41146	0.00E+00	89	0	INS	0.00E+00
1-756	1	266456953	266484267	NONE	gap called	266456953	266484267	5333	7.12E-23	79	69	INS	7.12E-23
1-757	1	266577895	266626589	NONE	gap called	266577895	266626589	5382	7.12E-23	60	48	INS	7.12E-23
1-758	1	266654982	266674518	NONE	gap called	266654982	266674518	9506	7.12E-23	57	48	INS	7.12E-23
1-762	1	267607145	267622323	NONE	gap called	267607145	267622323	8622	7.12E-23	81	59	INS	7.12E-23
1-770	1	272307819	272351493	NONE	gap called	272307819	272351493	6989	7.12E-23	50	43	INS	7.12E-23
1-785	1	278375203	278390617	NONE	gap called	278375203	278390617	106980	1.01E-22	49	34	INS	1.01E-22
1-788	1	283332606	283371461	NONE	gap called	283332606	283371461	12252	7.12E-23	82	63	INS	7.12E-23
1-793	1	284880674	284928616	NONE	gap called	284880674	284928616	8124	7.12E-23	64	44	INS	7.12E-23
1-797	1	288412030	288412030	NONE	gap called	288412030	288412030	18232	0.00E+00	52	0	INS	0.00E+00
1-803	1	289793793	289793793	NONE	gap called	289793793	289793793	26475	0.00E+00	78	0	INS	0.00E+00
1-807	1	291053746	291053746	NONE	gap called	291053746	291053746	69476	0.00E+00	70	0	INS	0.00E+00
1-815	1	299147475	299179000	NONE	gap called	299147475	299179000	6312	7.12E-23	66	51	INS	7.12E-23
1-816	1	299470329	299500226	NONE	gap called	299470329	299500226	6043	7.12E-23	57	45	INS	7.12E-23
2-3	2	1986716	2019891	NONE	gap called	1986716	2019891	5007	7.27E-23	55	40	INS	7.27E-23
2-6	2	5194600	5247793	NONE	gap called	5194600	5247793	7145	7.27E-23	55	51	INS	7.27E-23
2-8	2	5417832	5449698	NONE	gap called	5417832	5449698	6166	7.27E-23	65	46	INS	7.27E-23
2-10	2	5826512	5862400	NONE	gap called	5826512	5862400	4483	7.27E-23	60	50	INS	7.27E-23
2-12	2	5997560	6004741	NONE	gap called	5997560	6004741	4977	7.27E-23	68	49	INS	7.27E-23
2-14	2	9509533	9522518	NONE	gap called	9509533	9522518	6538	7.27E-23	75	52	INS	7.27E-23
2-17	2	12295731	12305657	NONE	gap called	12295731	12305657	5695	7.27E-23	51	49	INS	7.27E-23
2-19	2	12765742	12765742	NONE	gap called	12765742	12765742	26232	0.00E+00	52	0	INS	0.00E+00

2-37	2	19017656	19017656	NONE	gap called	19017656	19017656	16026	0.00E+00	47	0	INS	0.00E+00
2-40	2	19797316	19833057	NONE	gap called	19797316	19833057	7644	7.27E-23	57	47	INS	7.27E-23
2-45	2	20972824	21055828	NONE	gap called	20972824	21055828	9631	3.20E-12	27	20	INS	3.20E-12
2-46	2	21093517	21157399	NONE	gap called	21093517	21157399	7148	3.57E-13	34	24	INS	3.57E-13
2-54	2	23683923	23722586	NONE	gap called	23683923	23722586	8190	7.27E-23	50	41	INS	7.27E-23
2-71	2	27923113	27970333	NONE	gap called	27923113	27970333	13687	7.27E-23	72	58	INS	7.27E-23
2-76	2	31223620	31247845	NONE	gap called	31223620	31247845	12151	7.27E-23	47	35	INS	7.27E-23
2-77	2	31388493	31422484	NONE	gap called	31388493	31422484	7435	7.27E-23	58	46	INS	7.27E-23
2-93	2	36699375	36741592	NONE	gap called	36699375	36741592	7104	7.27E-23	46	41	INS	7.27E-23
2-98	2	38991586	38991586	NONE	gap called	38991586	38991586	7342	0.00E+00	99	0	INS	0.00E+00
2-101	2	39516675	39616263	NONE	gap called	39516675	39616263	16433	7.27E-23	27	22	INS	7.27E-23
2-102	2	39736510	39742500	NONE	gap called	39736510	39742500	6978	9.90E-23	22	15	INS	9.90E-23
2-104	2	40226371	40260045	NONE	gap called	40226371	40260045	8330	7.27E-23	55	45	INS	7.27E-23
2-110	2	45010700	45038995	NONE	gap called	45010700	45038995	21354	7.27E-23	46	36	INS	7.27E-23
2-116	2	46021200	46036398	NONE	gap called	46021200	46036398	7076	7.27E-23	46	42	INS	7.27E-23
2-118	2	46631152	46665604	NONE	gap called	46631152	46665604	7999	7.27E-23	44	37	INS	7.27E-23
2-119	2	46689532	46743973	NONE	gap called	46689532	46743973	24961	7.27E-23	49	30	INS	7.27E-23
2-120	2	46796336	46796336	NONE	gap called	46796336	46796336	14691	0.00E+00	65	0	INS	0.00E+00
2-124	2	47725058	47752583	NONE	gap called	47725058	47752583	5172	7.27E-23	46	41	INS	7.27E-23
2-128	2	48531354	48606892	NONE	gap called	48531354	48606892	12220	7.27E-23	34	24	INS	7.27E-23
2-131	2	49163633	49174432	NONE	gap called	49163633	49174432	6479	9.85E-23	61	53	INS	9.85E-23
2-138	2	53391185	53417300	NONE	gap called	53391185	53417300	6083	7.27E-23	38	29	INS	7.27E-23
2-140	2	53728897	53770159	NONE	gap called	53728897	53770159	10618	7.27E-23	64	49	INS	7.27E-23
2-144	2	57862475	57908834	NONE	gap called	57862475	57908834	5574	7.27E-23	67	49	INS	7.27E-23
2-154	2	62653788	62677883	NONE	gap called	62653788	62677883	6270	7.27E-23	76	54	INS	7.27E-23
2-158	2	65977854	66007803	NONE	gap called	65977854	66007803	5344	7.27E-23	51	43	INS	7.27E-23
2-163	2	67211712	67252241	NONE	gap called	67211712	67252241	16317	7.27E-23	59	46	INS	7.27E-23
2-166	2	67485494	67506427	NONE	gap called	67485494	67506427	11348	7.27E-23	90	77	INS	7.27E-23
2-168	2	67823101	67823101	NONE	gap called	67823101	67823101	11010	0.00E+00	82	0	INS	0.00E+00
2-186	2	72329332	72336054	NONE	gap called	72329332	72336054	5274	7.27E-23	61	55	INS	7.27E-23
2-188	2	72590355	72590355	NONE	gap called	72590355	72590355	77704	0.00E+00	43	0	INS	0.00E+00
2-189	2	73016739	73058375	NONE	gap called	73016739	73058375	5131	7.90E-14	30	23	INS	7.90E-14
2-195	2	75040381	75102115	NONE	gap called	75040381	75102115	6669	1.46E-07	23	14	INS	1.46E-07
2-200	2	76082574	76105125	NONE	gap called	76082574	76105125	5067	7.27E-23	44	40	INS	7.27E-23
2-207	2	80431921	80466579	NONE	gap called	80431921	80466579	7830	7.27E-23	44	32	INS	7.27E-23
2-226	2	86415893	86415893	NONE	gap called	86415893	86415893	70463	0.00E+00	39	0	INS	0.00E+00
2-228	2	87140277	87200827	NONE	gap called	87140277	87200827	10809	7.27E-23	45	42	INS	7.27E-23
2-229	2	87268305	87268305	NONE	gap called	87268305	87268305	18743	0.00E+00	48	0	INS	0.00E+00
2-232	2	88137342	88184534	NONE	gap called	88137342	88184534	7723	7.27E-23	41	31	INS	7.27E-23
2-236	2	92168704	92215107	NONE	gap called	92168704	92215107	6853	7.27E-23	49	32	INS	7.27E-23
2-238	2	92895683	92895683	NONE	gap called	92895683	92895683	72448	0.00E+00	59	0	INS	0.00E+00
2-241	2	93423533	93440302	NONE	gap called	93423533	93440302	6768	7.27E-23	54	43	INS	7.27E-23
2-253	2	96706609	96706609	NONE	gap called	96706609	96706609	30303	0.00E+00	55	0	INS	0.00E+00
2-255	2	97422352	97456628	NONE	gap called	97422352	97456628	7118	7.27E-23	48	39	INS	7.27E-23
2-259	2	98214698	98214698	NONE	gap called	98214698	98214698	12436	0.00E+00	66	0	INS	0.00E+00
2-260	2	98409281	98497357	NONE	gap called	98409281	98497357	14777	7.27E-23	48	32	INS	7.27E-23
2-261	2	98804442	98804442	NONE	gap called	98804442	98804442	70684	0.00E+00	47	0	INS	0.00E+00
2-264	2	99659497	99659497	NONE	gap called	99659497	99659497	19051	0.00E+00	41	0	INS	0.00E+00
2-267	2	100491316	100554600	NONE	gap called	100491316	100554600	108973	9.67E-23	24	21	INS	9.67E-23
2-281	2	112773646	112791462	NONE	gap called	112773646	112791462	5316	7.27E-23	75	50	INS	7.27E-23
2-289	2	198936057	198962352	NONE	gap called	198936057	198962352	7156	7.27E-23	58	50	INS	7.27E-23
2-291	2	199310833	199329496	NONE	gap called	199310833	199329496	7349	7.27E-23	83	57	INS	7.27E-23
2-301	2	204837163	204841507	NONE	gap called	204837163	204841507	6943	9.93E-23	31	19	INS	9.93E-23
2-307	2	209035977	209083161	NONE	gap called	209035977	209083161	7952	7.27E-23	42	37	INS	7.27E-23
2-313	2	211582454	211615348	NONE	gap called	211582454	211615348	6597	7.27E-23	47	35	INS	7.27E-23
2-316	2	211947418	211995420	NONE	gap called	211947418	211995420	7019	7.27E-23	44	36	INS	7.27E-23
2-320	2	216561076	216561076	NONE	gap called	216561076	216561076	60404	0.00E+00	78	0	INS	0.00E+00
2-322	2	216755478	216860391	NONE	gap called	216755478	216860391	16975	7.27E-23	60	49	INS	7.27E-23
2-326	2	219061370	219075700	NONE	gap called	219061370	219075700	5364	7.27E-23	81	49	INS	7.27E-23
2-327	2	219275807	219275807	NONE	gap called	219275807	219275807	11865	0.00E+00	78	0	INS	0.00E+00
2-328	2	219356088	219356088	NONE	gap called	219356088	219356088	19546	0.00E+00	76	0	INS	0.00E+00
2-329	2	219576306	219631723	NONE	gap called	219576306	219631723	23187	7.27E-23	67	43	INS	7.27E-23
2-333	2	220726518	220774116	NONE	gap called	220726518	220774116	6353	7.27E-23	44	36	INS	7.27E-23
2-336	2	221335873	221374326	NONE	gap called	221335873	221374326	18261	9.85E-23	57	45	INS	9.85E-23
2-344	2	227631232	227657482	NONE	gap called	227631232	227657482	7826	7.27E-23	40	28	INS	7.27E-23
2-349	2	229090587	229137021	NONE	gap called	229090587	229137021	8319	7.27E-23	61	29	INS	7.27E-23
3-8	3	4797664	4837035	NONE	gap called	4797664	4837035	10524	7.24E-23	25	20	INS	7.24E-23
3-11	3	12244314	12265893	NONE	gap called	12244314	12265893	11951	7.24E-23	76	52	INS	7.24E-23
3-15	3	17605347	17654036	NONE	gap called	17605347	17654036	5148	7.24E-23	77	65	INS	7.24E-23
3-16	3	17707817	17736841	NONE	gap called	17707817	17736841	7426	7.24E-23	75	65	INS	7.24E-23
3-21	3	18725384	18765274	NONE	gap called	18725384	18765274	10836	7.24E-23	47	35	INS	7.24E-23
3-22	3	23499184	23530893	NONE	gap called	23499184	23530893	5505	7.24E-23	60	44	INS	7.24E-23
3-24	3	25769612	25769612	NONE	gap called	25769612	25769612	6309	0.00E+00	65	0	INS	0.00E+00
3-25	3	26051967	26093336	NONE	gap called	26051967	26093336	9556	7.24E-23	62	49	INS	7.24E-23
3-26	3	26411796	26482067	NONE	gap called	26411796	26482067	8643	2.66E-22	51	37	INS	2.66E-22
3-32	3	28324860	28324860	NONE	gap called	28324860	28324860	28394	0.00E+00	79	0	INS	0.00E+00
3-34	3	28465088	28495715	NONE	gap called	28465088	28495715	40097	1.08E-22	79	64	INS	1.08E-22
3-35	3	28812714	28859100	NONE	gap called	28812714	28859100	7485	7.24E-23	55	35	INS	7.24E-23
3-39	3	29746354	29771290	NONE	gap called	29746354	29771290	6586	7.24E-23	63	49	INS	7.24E-23
3-45	3	31498723	31577560	NONE	gap called	31498723	31577560	12718	3.61E-18	22	17	INS	3.61E-18
3-50	3	32696448	32784205	NONE	gap called	32696448	32784205	9354	4.02E-09	26	18	INS	4.02E-09
3-51	3	33106332	33164742	NONE	gap called	33106332	33164742	6367	3.61E-18	52	39	INS	3.61E-18
3-55	3	34026868	34099650	NONE	gap called	34026868	34099650	19979	7.24E-23	32	28	INS	7.24E-23
3-64	3	36782280	36853292	NONE	gap called	36782280	36853292	12198	7.24E-23	42	36	INS	7.24E-23
3-65	3	36873224	36897050	NONE	gap called	36873224	36897050	35656	7.24E-23	44	35	INS	7.24E-23
3-69	3	37988532	38016487	NONE	gap called	37988532	38016487	6543	7.24E-23	79	54	INS	7.24E-23
3-70	3	38042365	38058428	NONE	gap called	38042365	38058428	6302	7.24E-23	74	59	INS	7.24E-23
3-72	3	38219199	38275168	NONE	gap called	38219199	38275168	7757	7.24E-23	53	46	INS	7.24E-23
3-87	3	42036913	42077494	NONE	gap called	42036913	42077494	9934	7.24E-23	48	43	INS	7.24E-23
3-89	3	42437892	42437892	NONE	gap called	42437892	42437892	11562	0.00E+00	62	0	INS	0.00E+00
3-94	3	43877431	43942582	NONE	gap called	43877431	43942582	27247	1.08E-22	48	38	INS	1.08E-22

3-98	3	44508024	44595250	NONE	gap called	44508024	44595250	20980	7.24E-23	40	31	INS	7.24E-23
3-101	3	45882550	45882550	NONE	gap called	45882550	45882550	28243	0.00E+00	85	0	INS	0.00E+00
3-105	3	46417324	46417324	NONE	gap called	46417324	46417324	39819	0.00E+00	67	0	INS	0.00E+00
3-106	3	46477616	46495468	NONE	gap called	46477616	46495468	36758	7.24E-23	53	38	INS	7.24E-23
3-117	3	50098959	50154469	NONE	gap called	50098959	50154469	8512	7.24E-23	42	33	INS	7.24E-23
3-121	3	50764433	50787166	NONE	gap called	50764433	50787166	3637	1.07E-22	69	50	INS	1.07E-22
3-123	3	50968781	50989019	NONE	gap called	50968781	50989019	6516	7.24E-23	61	49	INS	7.24E-23
3-132	3	53370708	53411251	NONE	gap called	53370708	53411251	9383	7.24E-23	40	31	INS	7.24E-23
3-136	3	54333990	54333990	NONE	gap called	54333990	54333990	34802	0.00E+00	65	0	INS	0.00E+00
3-139	3	55680675	55680675	NONE	gap called	55680675	55680675	11312	0.00E+00	77	0	INS	0.00E+00
3-143	3	56844500	56868319	NONE	gap called	56844500	56868319	11276	7.24E-23	43	36	INS	7.24E-23
3-148	3	59991169	60045401	NONE	gap called	59991169	60045401	6420	2.92E-12	34	22	INS	2.92E-12
3-155	3	63738438	63750240	NONE	gap called	63738438	63750240	28701	1.08E-22	68	54	INS	1.08E-22
3-157	3	64145508	64199464	NONE	gap called	64145508	64199464	63429	1.07E-22	36	26	INS	1.07E-22
3-163	3	65438109	65499588	NONE	gap called	65438109	65499588	11373	7.24E-23	27	18	INS	7.24E-23
3-166	3	66208171	66208171	NONE	gap called	66208171	66208171	6473	0.00E+00	64	0	INS	0.00E+00
3-186	3	77407901	77421712	NONE	gap called	77407901	77421712	8579	7.24E-23	50	40	INS	7.24E-23
3-189	3	78465139	78520496	NONE	gap called	78465139	78520496	5648	6.02E-19	65	47	INS	6.02E-19
3-190	3	78561070	78561070	NONE	gap called	78561070	78561070	29248	0.00E+00	62	0	INS	0.00E+00
3-193	3	79196632	79196632	NONE	gap called	79196632	79196632	20823	0.00E+00	70	0	INS	0.00E+00
3-203	3	81717016	81735918	NONE	gap called	81717016	81735918	5832	7.24E-23	74	42	INS	7.24E-23
3-211	3	85486095	85533002	NONE	gap called	85486095	85533002	6929	7.24E-23	64	48	INS	7.24E-23
3-215	3	87624008	87666324	NONE	gap called	87624008	87666324	6866	7.24E-23	52	44	INS	7.24E-23
3-229	3	96696946	96765654	NONE	gap called	96696946	96765654	10750	7.24E-23	33	27	INS	7.24E-23
3-234	3	98980750	99045061	NONE	gap called	98980750	99045061	16113	7.24E-23	28	25	INS	7.24E-23
3-239	3	99719187	99809005	NONE	gap called	99719187	99809005	12323	7.24E-23	53	40	INS	7.24E-23
3-241	3	101276520	101317707	NONE	gap called	101276520	101317707	5253	7.24E-23	52	39	INS	7.24E-23
3-244	3	101844059	101894953	NONE	gap called	101844059	101894953	6818	7.24E-23	48	42	INS	7.24E-23
3-246	3	102237718	102284885	NONE	gap called	102237718	102284885	5338	2.13E-21	54	45	INS	2.13E-21
3-250	3	103158815	103189218	NONE	gap called	103158815	103189218	17734	7.24E-23	49	35	INS	7.24E-23
3-260	3	106382492	106396907	NONE	gap called	106382492	106396907	13751	1.07E-22	55	39	INS	1.07E-22
3-266	3	111464146	111521869	NONE	gap called	111464146	111521869	4699	6.28E-10	61	36	INS	6.28E-10
3-269	3	112065637	112122091	NONE	gap called	112065637	112122091	6132	6.02E-19	52	41	INS	6.02E-19
3-272	3	112632391	112643648	NONE	gap called	112632391	112643648	10288	7.24E-23	60	48	INS	7.24E-23
3-275	3	113135292	113151478	NONE	gap called	113135292	113151478	8231	7.24E-23	63	52	INS	7.24E-23
3-287	3	121344439	121361862	NONE	gap called	121344439	121361862	6878	7.24E-23	78	61	INS	7.24E-23
3-291	3	124089262	124136174	NONE	gap called	124089262	124136174	8000	7.24E-23	57	51	INS	7.24E-23
3-297	3	126594314	126613759	NONE	gap called	126594314	126613759	14286	7.24E-23	78	45	INS	7.24E-23
3-299	3	126780351	126780351	NONE	gap called	126780351	126780351	69228	0.00E+00	54	0	INS	0.00E+00
3-302	3	131023468	131036639	NONE	gap called	131023468	131036639	8832	7.24E-23	57	45	INS	7.24E-23
3-304	3	131232446	131253383	NONE	gap called	131232446	131253383	5413	7.24E-23	66	49	INS	7.24E-23
3-305	3	131602719	131645650	NONE	gap called	131602719	131645650	5545	7.24E-23	65	46	INS	7.24E-23
3-308	3	132355106	132400410	NONE	gap called	132355106	132400410	12050	7.24E-23	58	46	INS	7.24E-23
3-312	3	135930694	135947161	NONE	gap called	135930694	135947161	7835	7.24E-23	66	50	INS	7.24E-23
3-314	3	136494649	136524388	NONE	gap called	136494649	136524388	6538	7.24E-23	75	50	INS	7.24E-23
3-315	3	136699017	136699017	NONE	gap called	136699017	136699017	45036	0.00E+00	68	0	INS	0.00E+00
3-320	3	137917652	137940180	NONE	gap called	137917652	137940180	8978	7.24E-23	50	37	INS	7.24E-23
3-322	3	138349325	138367451	NONE	gap called	138349325	138367451	6744	1.08E-22	37	33	INS	1.08E-22
3-323	3	138367451	138416501	NONE	gap called	138367451	138416501	5844	1.40E-15	35	29	INS	1.40E-15
3-343	3	143504876	143559525	NONE	gap called	143504876	143559525	9604	7.24E-23	75	60	INS	7.24E-23
3-352	3	147295041	147335031	NONE	gap called	147295041	147335031	7253	7.24E-23	49	42	INS	7.24E-23
3-355	3	148574704	148635871	NONE	gap called	148574704	148635871	12397	7.24E-23	34	33	INS	7.24E-23
3-356	3	148635871	148659576	NONE	gap called	148635871	148659576	6507	7.24E-23	37	29	INS	7.24E-23
3-360	3	150284649	150377866	NONE	gap called	150284649	150377866	28615	7.24E-23	52	40	INS	7.24E-23
3-363	3	152246604	152266655	NONE	gap called	152246604	152266655	7451	7.24E-23	42	30	INS	7.24E-23
3-368	3	157949796	157981274	NONE	gap called	157949796	157981274	9080	7.24E-23	39	35	INS	7.24E-23
3-372	3	160839862	160857870	NONE	gap called	160839862	160857870	8115	1.06E-22	46	37	INS	1.06E-22
3-377	3	161391486	161439091	NONE	gap called	161391486	161439091	6169	3.14E-15	29	24	INS	3.14E-15
3-391	3	168525522	168539242	NONE	gap called	168525522	168539242	8927	7.24E-23	63	45	INS	7.24E-23
3-396	3	172616049	172660261	NONE	gap called	172616049	172660261	8645	7.24E-23	49	36	INS	7.24E-23
3-401	3	174440057	174512496	NONE	gap called	174440057	174512496	8765	8.69E-18	40	30	INS	8.69E-18
3-403	3	175045593	175045593	NONE	gap called	175045593	175045593	153236	0.00E+00	21	0	INS	0.00E+00
3-404	3	175486972	175543920	NONE	gap called	175486972	175543920	5070	2.52E-09	32	29	INS	2.52E-09
3-405	3	175573930	175660118	NONE	gap called	175573930	175660118	10288	8.69E-18	39	30	INS	8.69E-18
3-416	3	183037008	183037008	NONE	gap called	183037008	183037008	7457	0.00E+00	62	0	INS	0.00E+00
3-418	3	183482549	183497881	NONE	gap called	183482549	183497881	5090	7.24E-23	65	47	INS	7.24E-23
3-436	3	187328752	187362615	NONE	gap called	187328752	187362615	12081	7.24E-23	72	58	INS	7.24E-23
3-438	3	187578665	187597144	NONE	gap called	187578665	187597144	6563	7.24E-23	78	71	INS	7.24E-23
3-439	3	187667229	187723469	NONE	gap called	187667229	187723469	7925	7.24E-23	67	62	INS	7.24E-23
3-443	3	188987334	189063714	NONE	gap called	188987334	189063714	7654	2.66E-22	65	55	INS	2.66E-22
3-445	3	189221524	189221524	NONE	gap called	189221524	189221524	11586	0.00E+00	84	0	INS	0.00E+00
3-448	3	189587118	189642904	NONE	gap called	189587118	189642904	9899	7.24E-23	43	36	INS	7.24E-23
3-449	3	189774710	189781123	NONE	gap called	189774710	189781123	5799	7.24E-23	35	25	INS	7.24E-23
3-454	3	195839207	195839207	NONE	gap called	195839207	195839207	9932	0.00E+00	80	0	INS	0.00E+00
3-456	3	196194562	196257923	NONE	gap called	196194562	196257923	11608	7.24E-23	23	19	INS	7.24E-23
3-460	3	200703948	200783132	NONE	gap called	200703948	200783132	52410	1.07E-22	55	43	INS	1.07E-22
3-466	3	202119954	202193306	NONE	gap called	202119954	202193306	12547	7.24E-23	66	53	INS	7.24E-23
3-472	3	203366200	203366200	NONE	gap called	203366200	203366200	8733	0.00E+00	72	0	INS	0.00E+00
3-473	3	203419790	203419790	NONE	gap called	203419790	203419790	113715	0.00E+00	53	0	INS	0.00E+00
3-495	3	210176557	210196815	NONE	gap called	210176557	210196815	6489	7.24E-23	81	61	INS	7.24E-23
3-503	3	212826287	212836847	NONE	gap called	212826287	212836847	6609	7.24E-23	82	65	INS	7.24E-23
3-510	3	214745618	214767213	NONE	gap called	214745618	214767213	7594	7.24E-23	74	59	INS	7.24E-23
3-511	3	214894101	214894101	NONE	gap called	214894101	214894101	7402	0.00E+00	74	0	INS	0.00E+00
3-514	3	215513986	215564616	NONE	gap called	215513986	215564616	30726	1.08E-22	69	58	INS	1.08E-22
3-518	3	216133772	216192635	NONE	gap called	216133772	216192635	7384	7.24E-23	56	44	INS	7.24E-23
3-521	3	216943572	216968874	NONE	gap called	216943572	216968874	12333	7.24E-23	81	73	INS	7.24E-23
3-526	3	219159318	219188834	NONE	gap called	219159318	219188834	31850	1.02E-22	58	49	INS	1.02E-22
3-528	3	219676564	219676564	NONE	gap called	219676564	219676564	17610	0.00E+00	74	0	INS	0.00E+00
3-529	3	219871474	219871474	NONE	gap called	219871474	219871474	14325	0.00E+00	96	0	INS	0.00E+00
3-534	3	221921979	222001165	NONE	gap called	221921979	222001165	14618	7.24E-23	39	29	INS	7.24E-23
3-542	3	226446406	226454510	NONE	gap called	226446406	226454510	8273	7.24E-23	68	45	INS	7.24E-23

3-545	3	226799293	226829154	NONE	gap called	226799293	226829154	5944	7.24E-23	56	45	INS	7.24E-23
3-555	3	229521776	229548428	NONE	gap called	229521776	229548428	9265	7.24E-23	87	62	INS	7.24E-23
4-3	4	5469760	5469760	NONE	gap called	5469760	5469760	8821	0.00E+00	59	0	INS	0.00E+00
4-6	4	5742463	5742463	NONE	gap called	5742463	5742463	6097	0.00E+00	66	0	INS	0.00E+00
4-9	4	6451480	6494330	NONE	gap called	6451480	6494330	6615	7.57E-23	76	65	INS	7.57E-23
4-20	4	8100248	8132979	NONE	gap called	8100248	8132979	6096	7.57E-23	32	30	INS	7.57E-23
4-24	4	9287549	9314708	NONE	gap called	9287549	9314708	9122	7.57E-23	46	37	INS	7.57E-23
4-26	4	9774999	9774999	NONE	gap called	9774999	9774999	56274	0.00E+00	63	0	INS	0.00E+00
4-30	4	11195836	11296556	NONE	gap called	11195836	11296556	43808	1.11E-22	50	35	INS	1.11E-22
4-37	4	13742517	13782924	NONE	gap called	13742517	13782924	6340	7.57E-23	63	54	INS	7.57E-23
4-46	4	18414669	18414669	NONE	gap called	18414669	18414669	106226	0.00E+00	53	0	INS	0.00E+00
4-49	4	19017719	19056523	NONE	gap called	19017719	19056523	5144	7.57E-23	92	67	INS	7.57E-23
4-63	4	29708855	29770137	NONE	gap called	29708855	29770137	8009	3.88E-18	36	26	INS	3.88E-18
4-75	4	36791932	36843005	NONE	gap called	36791932	36843005	12770	7.57E-23	40	33	INS	7.57E-23
4-78	4	37390895	37483663	NONE	gap called	37390895	37483663	13722	7.57E-23	49	38	INS	7.57E-23
4-82	4	38186735	38343015	NONE	gap called	38186735	38343015	22563	1.02E-19	21	21	INS	1.02E-19
4-84	4	38669300	38703039	NONE	gap called	38669300	38703039	6488	7.57E-23	63	42	INS	7.57E-23
4-96	4	42474200	42518418	NONE	gap called	42474200	42518418	8342	7.57E-23	82	56	INS	7.57E-23
4-101	4	43450974	43450974	NONE	gap called	43450974	43450974	20098	0.00E+00	59	0	INS	0.00E+00
4-104	4	43931487	43963435	NONE	gap called	43931487	43963435	5030	7.57E-23	57	47	INS	7.57E-23
4-107	4	47921720	47948478	NONE	gap called	47921720	47948478	8019	7.57E-23	27	25	INS	7.57E-23
4-112	4	49180471	49236544	NONE	gap called	49180471	49236544	15563	7.57E-23	31	26	INS	7.57E-23
4-116	4	51222666	51222666	NONE	gap called	51222666	51222666	10880	0.00E+00	58	0	INS	0.00E+00
4-119	4	52328607	52328607	NONE	gap called	52328607	52328607	28757	0.00E+00	48	0	INS	0.00E+00
4-122	4	53110459	53154171	NONE	gap called	53110459	53154171	12477	7.57E-23	36	27	INS	7.57E-23
4-134	4	60511935	60535501	NONE	gap called	60511935	60535501	7877	7.57E-23	49	40	INS	7.57E-23
4-140	4	62163502	62163502	NONE	gap called	62163502	62163502	47033	0.00E+00	48	0	INS	0.00E+00
4-142	4	62747165	62846508	NONE	gap called	62747165	62846508	23793	1.11E-22	40	36	INS	1.11E-22
4-147	4	64207786	64272729	NONE	gap called	64207786	64272729	8776	7.57E-23	46	41	INS	7.57E-23
4-150	4	66364486	66383772	NONE	gap called	66364486	66383772	5666	7.57E-23	55	43	INS	7.57E-23
4-151	4	66433555	66451641	NONE	gap called	66433555	66451641	5244	7.57E-23	66	47	INS	7.57E-23
4-154	4	68282165	68346842	NONE	gap called	68282165	68346842	6748	3.65E-14	39	31	INS	3.65E-14
4-157	4	69162616	69162616	NONE	gap called	69162616	69162616	12663	0.00E+00	74	0	INS	0.00E+00
4-169	4	72104583	72143652	NONE	gap called	72104583	72143652	8563	7.57E-23	66	47	INS	7.57E-23
4-174	4	73432083	73482043	NONE	gap called	73432083	73482043	5704	7.57E-23	58	48	INS	7.57E-23
4-176	4	74162686	74205268	NONE	gap called	74162686	74205268	14009	1.10E-22	80	56	INS	1.10E-22
4-177	4	74962898	75022501	NONE	gap called	74962898	75022501	59528	1.10E-22	51	39	INS	1.10E-22
4-179	4	76278032	76324839	NONE	gap called	76278032	76324839	7599	7.57E-23	37	36	INS	7.57E-23
4-190	4	80486434	80603015	NONE	gap called	80486434	80603015	12583	1.05E-10	27	20	INS	1.05E-10
4-192	4	80798297	80830597	NONE	gap called	80798297	80830597	11979	7.57E-23	54	46	INS	7.57E-23
4-193	4	81131878	81131878	NONE	gap called	81131878	81131878	28131	0.00E+00	64	0	INS	0.00E+00
4-197	4	82085772	82098954	NONE	gap called	82085772	82098954	11507	1.11E-22	57	45	INS	1.11E-22
4-201	4	82875810	82888887	NONE	gap called	82875810	82888887	6256	7.57E-23	48	34	INS	7.57E-23
4-202	4	82975191	83135532	NONE	gap called	82975191	83135532	53048	1.09E-22	28	23	INS	1.09E-22
4-206	4	84440919	84454699	NONE	gap called	84440919	84454699	6976	7.57E-23	61	53	INS	7.57E-23
4-207	4	84517360	84555473	NONE	gap called	84517360	84555473	11028	7.57E-23	42	37	INS	7.57E-23
4-213	4	86095163	86121473	NONE	gap called	86095163	86121473	6328	7.57E-23	60	51	INS	7.57E-23
4-222	4	88023184	88050539	NONE	gap called	88023184	88050539	5421	7.57E-23	62	50	INS	7.57E-23
4-223	4	88095636	88109457	NONE	gap called	88095636	88109457	5262	7.57E-23	66	29	INS	7.57E-23
4-224	4	88269671	88269671	NONE	gap called	88269671	88269671	118943	0.00E+00	52	0	INS	0.00E+00
4-234	4	91542121	91542670	NONE	gap called	91542121	91542670	7953	7.57E-23	67	55	INS	7.57E-23
4-236	4	91966296	91992971	NONE	gap called	91966296	91992971	12078	7.57E-23	41	34	INS	7.57E-23
4-237	4	92219452	92267418	NONE	gap called	92219452	92267418	5196	1.69E-13	35	29	INS	1.69E-13
4-244	4	94396008	94468306	NONE	gap called	94396008	94468306	9681	8.17E-22	47	29	INS	8.17E-22
4-250	4	95985976	96015907	NONE	gap called	95985976	96015907	8368	7.57E-23	30	25	INS	7.57E-23
4-251	4	96083087	96105433	NONE	gap called	96083087	96105433	9772	7.57E-23	35	29	INS	7.57E-23
4-255	4	97642117	97653351	NONE	gap called	97642117	97653351	7765	7.57E-23	59	50	INS	7.57E-23
4-259	4	99254381	99285858	NONE	gap called	99254381	99285858	11279	7.57E-23	62	50	INS	7.57E-23
4-260	4	99861607	99888616	NONE	gap called	99861607	99888616	9193	7.57E-23	50	45	INS	7.57E-23
4-267	4	101210711	101210711	NONE	gap called	101210711	101210711	7771	0.00E+00	53	0	INS	0.00E+00
4-272	4	102829491	102879014	NONE	gap called	102829491	102879014	8191	7.57E-23	64	57	INS	7.57E-23
4-275	4	105851607	105904655	NONE	gap called	105851607	105904655	17009	7.57E-23	57	50	INS	7.57E-23
4-276	4	106850677	106850677	NONE	gap called	106850677	106850677	11277	0.00E+00	69	0	INS	0.00E+00
4-277	4	106885138	106903673	NONE	gap called	106885138	106903673	7642	7.57E-23	81	66	INS	7.57E-23
4-282	4	109743562	109743562	NONE	gap called	109743562	109743562	11361	0.00E+00	57	0	INS	0.00E+00
4-293	4	113903009	113964215	NONE	gap called	113903009	113964215	7666	5.93E-21	39	33	INS	5.93E-21
4-296	4	114431330	114486258	NONE	gap called	114431330	114486258	6713	7.57E-23	68	53	INS	7.57E-23
4-298	4	115532373	115558493	NONE	gap called	115532373	115558493	7954	7.57E-23	59	41	INS	7.57E-23
4-299	4	115934477	116003440	NONE	gap called	115934477	116003440	8880	7.57E-23	43	37	INS	7.57E-23
4-314	4	121178139	121234597	NONE	gap called	121178139	121234597	6288	6.40E-19	42	38	INS	6.40E-19
4-319	4	122862395	122893984	NONE	gap called	122862395	122893984	6591	7.57E-23	51	36	INS	7.57E-23
4-323	4	123960619	123960619	NONE	gap called	123960619	123960619	8057	0.00E+00	70	0	INS	0.00E+00
4-325	4	125363518	125405759	NONE	gap called	125363518	125405759	7873	7.57E-23	64	43	INS	7.57E-23
4-327	4	132050041	132084033	NONE	gap called	132050041	132084033	5233	7.57E-23	67	59	INS	7.57E-23
4-337	4	136343118	136380305	NONE	gap called	136343118	136380305	8359	7.57E-23	94	73	INS	7.57E-23
4-340	4	137185533	137185533	NONE	gap called	137185533	137185533	10330	0.00E+00	56	0	INS	0.00E+00
4-343	4	138069213	138079614	NONE	gap called	138069213	138079614	6775	7.57E-23	42	35	INS	7.57E-23
4-344	4	138287003	138303110	NONE	gap called	138287003	138303110	5095	7.57E-23	46	34	INS	7.57E-23
4-348	4	140089997	140153703	NONE	gap called	140089997	140153703	6673	2.25E-17	49	38	INS	2.25E-17
4-349	4	140304920	140343345	NONE	gap called	140304920	140343345	14419	7.57E-23	54	45	INS	7.57E-23
4-354	4	142016079	142051469	NONE	gap called	142016079	142051469	5021	7.57E-23	67	49	INS	7.57E-23
4-356	4	142175938	142199924	NONE	gap called	142175938	142199924	5100	7.57E-23	71	58	INS	7.57E-23
4-372	4	149455399	149512122	NONE	gap called	149455399	149512122	15370	7.57E-23	65	43	INS	7.57E-23
4-375	4	152827081	152849116	NONE	gap called	152827081	152849116	8596	7.57E-23	79	51	INS	7.57E-23
4-386	4	156716299	156716299	NONE	gap called	156716299	156716299	43838	0.00E+00	81	0	INS	0.00E+00
4-388	4	156891632	156949787	NONE	gap called	156891632	156949787	22808	1.07E-22	62	47	INS	1.07E-22
4-396	4	158772110	158783754	NONE	gap called	158772110	158783754	21871	7.57E-23	63	51	INS	7.57E-23
4-406	4	160218644	160218644	NONE	gap called	160218644	160218644	16980	0.00E+00	71	0	INS	0.00E+00
4-415	4	162229234	162264665	NONE	gap called	162229234	162264665	8170	7.57E-23	24	18	INS	7.57E-23
4-419	4	163095716	163100009	NONE	gap called	163095716	163100009	8009	1.10E-22	66	49	INS	1.10E-22
4-425	4	164395367	164413958	NONE	gap called	164395367	164413958	5834	7.57E-23	72	50	INS	7.57E-23

4-439	4	168258778	168258778	NONE	gap called	168258778	168258778	27728	0.00E+00	57	0	INS	0.00E+00
4-449	4	170591993	170605250	NONE	gap called	170591993	170605250	7098	7.57E-23	98	75	INS	7.57E-23
4-450	4	170731563	170745661	NONE	gap called	170731563	170745661	6848	7.57E-23	88	67	INS	7.57E-23
4-460	4	174603982	174682372	NONE	gap called	174603982	174682372	9499	7.57E-23	87	69	INS	7.57E-23
4-461	4	174886236	174898588	NONE	gap called	174886236	174898588	6495	7.57E-23	90	68	INS	7.57E-23
4-484	4	187009946	187037564	NONE	gap called	187009946	187037564	8461	7.57E-23	58	45	INS	7.57E-23
4-489	4	189845891	189867684	NONE	gap called	189845891	189867684	11049	7.57E-23	93	76	INS	7.57E-23
4-495	4	191724410	191877509	NONE	gap called	191724410	191877509	34189	7.57E-23	23	14	INS	7.57E-23
4-506	4	198415611	198427903	NONE	gap called	198415611	198427903	9707	7.57E-23	86	64	INS	7.57E-23
4-516	4	201695741	201708808	NONE	gap called	201695741	201708808	11695	7.57E-23	73	62	INS	7.57E-23
4-534	4	205577848	205577848	NONE	gap called	205577848	205577848	26367	0.00E+00	65	0	INS	0.00E+00
4-537	4	205926049	205926049	NONE	gap called	205926049	205926049	68740	0.00E+00	48	0	INS	0.00E+00
4-538	4	206073965	206073965	NONE	gap called	206073965	206073965	8081	0.00E+00	56	0	INS	0.00E+00
4-544	4	207801091	207817100	NONE	gap called	207801091	207817100	8923	7.57E-23	76	54	INS	7.57E-23
4-545	4	208070196	208087960	NONE	gap called	208070196	208087960	11278	7.57E-23	61	39	INS	7.57E-23
4-548	4	208954072	208990691	NONE	gap called	208954072	208990691	7655	7.57E-23	81	53	INS	7.57E-23
4-562	4	213274392	213335299	NONE	gap called	213274392	213335299	34796	7.57E-23	36	32	INS	7.57E-23
4-568	4	215099875	215156611	NONE	gap called	215099875	215156611	9690	7.57E-23	49	37	INS	7.57E-23
4-570	4	215552729	215582615	NONE	gap called	215552729	215582615	6606	7.57E-23	58	44	INS	7.57E-23
4-574	4	216624752	216648468	NONE	gap called	216624752	216648468	8405	7.57E-23	54	34	INS	7.57E-23
4-579	4	218434701	218434701	NONE	gap called	218434701	218434701	51445	0.00E+00	41	0	INS	0.00E+00
4-585	4	220743653	220768845	NONE	gap called	220743653	220768845	6055	7.57E-23	58	47	INS	7.57E-23
4-586	4	221380225	221380225	NONE	gap called	221380225	221380225	36811	0.00E+00	58	0	INS	0.00E+00
4-589	4	221941977	221965917	NONE	gap called	221941977	221965917	6540	7.57E-23	55	42	INS	7.57E-23
4-590	4	222182503	222192952	NONE	gap called	222182503	222192952	5464	7.57E-23	49	42	INS	7.57E-23
4-593	4	222779004	222792546	NONE	gap called	222779004	222792546	7155	7.57E-23	75	69	INS	7.57E-23
4-594	4	222792546	222811534	NONE	gap called	222792546	222811534	5545	7.57E-23	73	60	INS	7.57E-23
4-596	4	223312881	223361719	NONE	gap called	223312881	223361719	13964	7.57E-23	62	53	INS	7.57E-23
4-602	4	223853580	223888585	NONE	gap called	223853580	223888585	7516	7.57E-23	45	36	INS	7.57E-23
4-605	4	224668354	224722088	NONE	gap called	224668354	224722088	5460	7.61E-15	41	36	INS	7.61E-15
4-609	4	225188115	225188115	NONE	gap called	225188115	225188115	7318	0.00E+00	62	0	INS	0.00E+00
4-612	4	225503248	225527553	NONE	gap called	225503248	225527553	12668	7.57E-23	42	35	INS	7.57E-23
4-617	4	226414862	226414862	NONE	gap called	226414862	226414862	12277	0.00E+00	81	0	INS	0.00E+00
4-619	4	226580250	226626901	NONE	gap called	226580250	226626901	6424	7.57E-23	71	50	INS	7.57E-23
4-621	4	226778383	226778383	NONE	gap called	226778383	226778383	12564	0.00E+00	84	0	INS	0.00E+00
4-625	4	227774399	227799763	NONE	gap called	227774399	227799763	8326	7.57E-23	49	34	INS	7.57E-23
4-628	4	231061322	231097161	NONE	gap called	231061322	231097161	15123	7.57E-23	42	33	INS	7.57E-23
4-635	4	232831093	232861335	NONE	gap called	232831093	232861335	6580	7.57E-23	61	40	INS	7.57E-23
4-636	4	232889788	232889788	NONE	gap called	232889788	232889788	86205	0.00E+00	59	0	INS	0.00E+00
4-641	4	233955355	233955355	NONE	gap called	233955355	233955355	7644	0.00E+00	86	0	INS	0.00E+00
4-649	4	235578687	235605616	NONE	gap called	235578687	235605616	6906	7.57E-23	76	52	INS	7.57E-23
4-652	4	236512282	236594847	NONE	gap called	236512282	236594847	45509	1.10E-22	30	25	INS	1.10E-22
4-661	4	242193142	242265071	NONE	gap called	242193142	242265071	7230	2.25E-17	46	41	INS	2.25E-17
4-667	4	243857084	243873630	NONE	gap called	243857084	243873630	7806	7.57E-23	91	69	INS	7.57E-23
4-668	4	244075845	244075845	NONE	gap called	244075845	244075845	78410	0.00E+00	68	0	INS	0.00E+00
4-673	4	245081160	245081160	NONE	gap called	245081160	245081160	7810	0.00E+00	69	0	INS	0.00E+00
4-674	4	245422542	245458568	NONE	gap called	245422542	245458568	10447	7.57E-23	40	35	INS	7.57E-23
4-675	4	245531149	245542859	NONE	gap called	245531149	245542859	5160	7.57E-23	51	43	INS	7.57E-23
5-8	5	2148852	2177048	NONE	gap called	2148852	2177048	6756	7.30E-23	45	37	INS	7.30E-23
5-20	5	4740102	4769040	NONE	gap called	4740102	4769040	7359	7.30E-23	79	61	INS	7.30E-23
5-21	5	4927210	4989527	NONE	gap called	4927210	4989527	12643	7.30E-23	59	43	INS	7.30E-23
5-24	5	5462102	5462102	NONE	gap called	5462102	5462102	53341	0.00E+00	66	0	INS	0.00E+00
5-27	5	6204127	6266682	NONE	gap called	6204127	6266682	9149	7.30E-23	53	43	INS	7.30E-23
5-49	5	16675564	16734605	NONE	gap called	16675564	16734605	11346	7.30E-23	27	25	INS	7.30E-23
5-56	5	23290213	23299484	NONE	gap called	23290213	23299484	7300	7.30E-23	79	55	INS	7.30E-23
5-69	5	32476299	32509126	NONE	gap called	32476299	32509126	6052	7.30E-23	89	69	INS	7.30E-23
5-72	5	33664975	33685768	NONE	gap called	33664975	33685768	5355	7.30E-23	45	36	INS	7.30E-23
5-75	5	34223288	34223288	NONE	gap called	34223288	34223288	19932	0.00E+00	73	0	INS	0.00E+00
5-89	5	38489898	38531067	NONE	gap called	38489898	38531067	5987	7.30E-23	79	57	INS	7.30E-23
5-100	5	42123864	42140810	NONE	gap called	42123864	42140810	8440	7.30E-23	60	49	INS	7.30E-23
5-104	5	43839405	43890647	NONE	gap called	43839405	43890647	5997	7.30E-23	68	43	INS	7.30E-23
5-108	5	45694265	45717429	NONE	gap called	45694265	45717429	15872	1.02E-22	49	34	INS	1.02E-22
5-121	5	51286521	51381565	NONE	gap called	51286521	51381565	17466	7.30E-23	29	25	INS	7.30E-23
5-138	5	56237489	56271687	NONE	gap called	56237489	56271687	6454	7.30E-23	62	48	INS	7.30E-23
5-141	5	56698555	56698555	NONE	gap called	56698555	56698555	5609	0.00E+00	54	0	INS	0.00E+00
5-147	5	57422669	57486290	NONE	gap called	57422669	57486290	6518	7.30E-23	70	59	INS	7.30E-23
5-167	5	69366747	69403119	NONE	gap called	69366747	69403119	6804	7.30E-23	62	48	INS	7.30E-23
5-179	5	72471605	72471605	NONE	gap called	72471605	72471605	9584	0.00E+00	47	0	INS	0.00E+00
5-180	5	72696482	72720923	NONE	gap called	72696482	72720923	6954	7.30E-23	38	38	INS	7.30E-23
5-186	5	74174133	74195387	NONE	gap called	74174133	74195387	5999	7.30E-23	79	55	INS	7.30E-23
5-187	5	74274297	74316834	NONE	gap called	74274297	74316834	9776	7.30E-23	75	51	INS	7.30E-23
5-188	5	74517778	74530528	NONE	gap called	74517778	74530528	8041	7.30E-23	72	41	INS	7.30E-23
5-193	5	75760377	75813677	NONE	gap called	75760377	75813677	7149	7.30E-23	53	42	INS	7.30E-23
5-212	5	84211915	84224591	NONE	gap called	84211915	84224591	7698	7.30E-23	97	73	INS	7.30E-23
5-215	5	84790869	84819623	NONE	gap called	84790869	84819623	10295	7.30E-23	50	43	INS	7.30E-23
5-241	5	91986776	91986776	NONE	gap called	91986776	91986776	5356	0.00E+00	61	0	INS	0.00E+00
5-257	5	97943126	97981873	NONE	gap called	97943126	97981873	7771	7.30E-23	69	53	INS	7.30E-23
5-265	5	99860366	99885885	NONE	gap called	99860366	99885885	6490	7.30E-23	51	40	INS	7.30E-23
5-270	5	104409261	104456411	NONE	gap called	104409261	104456411	5189	1.21E-16	39	34	INS	1.21E-16
5-271	5	104600538	104650064	NONE	gap called	104600538	104650064	9830	7.30E-23	29	28	INS	7.30E-23
5-272	5	105715701	105736247	NONE	gap called	105715701	105736247	5425	7.30E-23	15	11	INS	7.30E-23
5-283	5	108880072	108920816	NONE	gap called	108880072	108920816	3890	2.04E-14	57	41	INS	2.04E-14
5-290	5	110572337	110572337	NONE	gap called	110572337	110572337	11183	0.00E+00	70	0	INS	0.00E+00
5-295	5	113359248	113426974	NONE	gap called	113359248	113426974	4781	2.81E-07	35	32	INS	2.81E-07
5-303	5	118306554	118362316	NONE	gap called	118306554	118362316	5978	2.15E-21	54	46	INS	2.15E-21
5-313	5	121329069	121329069	NONE	gap called	121329069	121329069	26207	0.00E+00	49	0	INS	0.00E+00
5-320	5	123495332	123551107	NONE	gap called	123495332	123551107	12388	7.30E-23	55	50	INS	7.30E-23
5-325	5	124384226	124442113	NONE	gap called	124384226	124442113	8503	7.30E-23	45	39	INS	7.30E-23
5-327	5	124691831	124718391	NONE	gap called	124691831	124718391	7854	7.30E-23	29	28	INS	7.30E-23
5-338	5	127474493	127499484	NONE	gap called	127474493	127499484	6872	7.30E-23	13	9	INS	7.30E-23
5-348	5	132715805	132762900	NONE	gap called	132715805	132762900	3592	6.49E-12	70	50	INS	6.49E-12

5-359	5	135697964	135697964	NONE	gap called	135697964	135697964	9696	0.00E+00	79	0	INS	0.00E+00	
5-360	5	135792596	135879043	NONE	gap called	135792596	135879043	10182	2.16E-17	46	28	INS	2.16E-17	
5-363	5	136525020	136546161	NONE	gap called	136525020	136546161	8809	7.30E-23	50	42	INS	7.30E-23	
5-366	5	137290994	137290994	NONE	gap called	137290994	137290994	16159	0.00E+00	64	0	INS	0.00E+00	
5-370	5	138187377	138206325	NONE	gap called	138187377	138206325	3380	1.03E-22	71	53	INS	1.03E-22	
5-377	5	139851699	139851699	NONE	gap called	139851699	139851699	73650	0.00E+00	45	0	INS	0.00E+00	
5-389	5	144429636	144429636	NONE	gap called	144429636	144429636	7183	0.00E+00	65	0	INS	0.00E+00	
5-393	5	146005598	146025649	NONE	gap called	146005598	146025649	11855	7.30E-23	76	59	INS	7.30E-23	
5-398	5	147418946	147458829	NONE	gap called	147418946	147458829	7350	6.49E-12	13	9	INS	6.49E-12	
5-415	5	157069996	157115743	NONE	gap called	157069996	157115743	10748	7.30E-23	56	36	INS	7.30E-23	
5-426	5	162389476	162389476	NONE	gap called	162389476	162389476	8042	0.00E+00	79	0	INS	0.00E+00	
5-430	5	163317808	163317808	NONE	gap called	163317808	163317808	21004	0.00E+00	77	0	INS	0.00E+00	
5-434	5	167876492	167911699	NONE	gap called	167876492	167911699	15614	7.30E-23	66	53	INS	7.30E-23	
5-442	5	170396924	170420810	NONE	gap called	170396924	170420810	5246	7.30E-23	48	40	INS	7.30E-23	
5-449	5	175044512	175114381	NONE	gap called	175044512	175114381	11182	7.30E-23	48	36	INS	7.30E-23	
5-450	5	177178721	177178721	NONE	gap called	177178721	177178721	21126	0.00E+00	62	0	INS	0.00E+00	
5-455	5	181449965	181505889	NONE	gap called	181449965	181505889	8696	7.30E-23	41	33	INS	7.30E-23	
5-458	5	184688834	184704580	NONE	gap called	184688834	184704580	6295	7.30E-23	72	54	INS	7.30E-23	
5-466	5	186557578	186593604	NONE	gap called	186557578	186593604	13298	7.30E-23	59	38	INS	7.30E-23	
5-468	5	187202094	187234296	NONE	gap called	187202094	187234296	5425	7.30E-23	58	40	INS	7.30E-23	
5-475	5	194874381	194924104	NONE	gap called	194874381	194924104	7646	7.30E-23	38	27	INS	7.30E-23	
5-494	5	212045849	212075111	NONE	gap called	212045849	212075111	5532	2.82E-16	17	13	INS	2.82E-16	
5-501	5	213959861	213984393	NONE	gap called	213959861	213984393	7919	7.30E-23	74	68	INS	7.30E-23	
6-4	6	3262933	3268343	NONE	gap called	3262933	3268343	5369	8.15E-23	52	32	INS	8.15E-23	
6-7	6	8428024	8472999	NONE	gap called	8428024	8472999	5591	1.63E-12	27	21	INS	1.63E-12	
6-16	6	19338426	19357860	NONE	gap called	19338426	19357860	7987	8.15E-23	57	44	INS	8.15E-23	
6-19	6	20128757	20137013	NONE	gap called	20128757	20137013	7311	8.15E-23	67	50	INS	8.15E-23	
6-22	6	20760353	20760353	NONE	gap called	20760353	20760353	7478	0.00E+00	70	0	INS	0.00E+00	
6-42	6	34610954	34628036	NONE	gap called	34610954	34628036	5689	8.15E-23	51	35	INS	8.15E-23	
6-47	6	36556155	36601085	NONE	gap called	36556155	36601085	6260	8.15E-23	35	31	INS	8.15E-23	
6-61	6	40228386	40228386	NONE	gap called	40228386	40228386	29670	0.00E+00	43	0	INS	0.00E+00	
6-64	6	40839113	40857191	NONE	gap called	40839113	40857191	11398	8.15E-23	98	81	INS	8.15E-23	
6-69	6	42373670	42401293	NONE	gap called	42373670	42401293	5608	8.15E-23	91	68	INS	8.15E-23	
6-80	6	52630261	52702080	NONE	gap called	52630261	52702080	6814	1.43E-19	68	53	INS	1.43E-19	
6-95	6	56228815	56266974	NONE	gap called	56228815	56266974	6049	8.15E-23	43	37	INS	8.15E-23	
6-99	6	56815593	56865259	NONE	gap called	56815593	56865259	6234	8.15E-23	63	53	INS	8.15E-23	
6-103	6	59946638	59993031	NONE	gap called	59946638	59993031	7153	8.15E-23	52	44	INS	8.15E-23	
6-109	6	61653333	61674137	NONE	gap called	61653333	61674137	7952	8.15E-23	65	47	INS	8.15E-23	
6-112	6	62488449	62488449	NONE	gap called	62488449	62488449	52345	0.00E+00	55	0	INS	0.00E+00	
6-127	6	66363402	66397013	NONE	gap called	66363402	66397013	5478	8.15E-23	50	30	INS	8.15E-23	
6-133	6	67748831	67802200	NONE	gap called	67748831	67802200	5455	3.86E-14	37	34	INS	3.86E-14	
6-136	6	69592843	69635739	NONE	gap called	69592843	69635739	9705	8.15E-23	37	32	INS	8.15E-23	
6-140	6	70564497	70578781	NONE	gap called	70564497	70578781	5167	8.15E-23	78	54	INS	8.15E-23	
6-157	6	78829249	78867675	NONE	gap called	78829249	78867675	9137	8.15E-23	69	54	INS	8.15E-23	
6-166	6	84796562	84807832	NONE	gap called	84796562	84807832	5569	8.15E-23	77	64	INS	8.15E-23	
6-173	6	138394985	138394985	NONE	gap called	138394985	138394985	17713	0.00E+00	89	0	INS	0.00E+00	
6-175	6	152925163	152994013	NONE	gap called	152925163	152994013	12538	8.15E-23	27	26	INS	8.15E-23	
7-3	7	5786768	5786768	NONE	gap called	5786768	5786768	40714	0.00E+00	14	0	INS	0.00E+00	
7-4	7	6146406	6186478	NONE	gap called	6146406	6186478	8516	7.74E-23	75	61	INS	7.74E-23	
7-6	7	6504895	6504895	NONE	gap called	6504895	6504895	34224	0.00E+00	80	0	INS	0.00E+00	
7-7	7	6675754	6806462	NONE	gap called	6675754	6806462	13184	2.98E-16	47	35	INS	2.98E-16	
7-14	7	7853323	7853323	NONE	gap called	7853323	7853323	18398	0.00E+00	70	0	INS	0.00E+00	
7-18	7	8851454	8871232	NONE	gap called	8851454	8871232	6473	7.74E-23	90	75	INS	7.74E-23	
7-24	7	11462634	11462634	NONE	gap called	11462634	11462634	27994	0.00E+00	65	0	INS	0.00E+00	
7-27	7	15189044	15230183	NONE	gap called	15189044	15230183	8512	7.74E-23	53	39	INS	7.74E-23	
7-28	7	15348114	15374258	NONE	gap called	15348114	15374258	5493	7.74E-23	64	49	INS	7.74E-23	
7-36	7	18805005	18860691	NONE	gap called	18805005	18860691	5876	9.45E-18	48	39	INS	9.45E-18	
7-45	7	28179020	28202032	NONE	gap called	28179020	28202032	7379	7.74E-23	49	37	INS	7.74E-23	
7-49	7	32209803	32234480	NONE	gap called	32209803	32234480	5786	7.74E-23	58	44	INS	7.74E-23	
7-53	7	35143769	35414750	NONE	gap called	35143769	35414750	10056	3.49E-02	20	18	INS	3.49E-02	complex event
7-62	7	38482352	38511663	NONE	gap called	38482352	38511663	13145	7.74E-23	32	28	INS	7.74E-23	
7-64	7	38856530	38857081	NONE	gap called	38856530	38857081	19602	7.74E-23	59	40	INS	7.74E-23	
7-69	7	40350518	40382006	NONE	gap called	40350518	40382006	10941	7.74E-23	64	50	INS	7.74E-23	
7-75	7	41661451	41706006	NONE	gap called	41661451	41706006	7686	7.74E-23	55	42	INS	7.74E-23	
7-82	7	45049001	45054056	NONE	gap called	45049001	45054056	6011	7.74E-23	61	48	INS	7.74E-23	
7-94	7	48254332	48254332	NONE	gap called	48254332	48254332	31450	0.00E+00	61	0	INS	0.00E+00	
7-97	7	48626698	48626698	NONE	gap called	48626698	48626698	18314	0.00E+00	39	0	INS	0.00E+00	
7-102	7	50122600	50158800	NONE	gap called	50122600	50158800	7700	7.74E-23	61	50	INS	7.74E-23	
7-115	7	59462328	59523872	NONE	gap called	59462328	59523872	10142	7.74E-23	49	33	INS	7.74E-23	
7-116	7	59581654	59669061	NONE	gap called	59581654	59669061	17101	7.74E-23	34	25	INS	7.74E-23	
7-124	7	61940851	61940851	NONE	gap called	61940851	61940851	6914	0.00E+00	71	0	INS	0.00E+00	
7-126	7	62499937	62527815	NONE	gap called	62499937	62527815	8450	7.74E-23	54	50	INS	7.74E-23	
7-133	7	64169507	64181894	NONE	gap called	64169507	64181894	5151	7.74E-23	65	44	INS	7.74E-23	
7-139	7	66101374	66108522	NONE	gap called	66101374	66108522	8789	7.74E-23	84	54	INS	7.74E-23	
7-141	7	66644573	66653322	NONE	gap called	66644573	66653322	9834	7.74E-23	78	50	INS	7.74E-23	
7-154	7	71840295	71840295	NONE	gap called	71840295	71840295	35707	0.00E+00	58	0	INS	0.00E+00	
7-170	7	77047754	77174923	NONE	gap called	77047754	77174923	18571	7.74E-23	34	31	INS	7.74E-23	
7-174	7	78316089	78351880	NONE	gap called	78316089	78351880	10343	7.74E-23	46	39	INS	7.74E-23	
7-179	7	79906887	79980244	NONE	gap called	79906887	79980244	8935	7.74E-23	59	45	INS	7.74E-23	
7-184	7	83550408	83567624	NONE	gap called	83550408	83567624	5370	7.74E-23	45	43	INS	7.74E-23	
7-185	7	83637244	83694746	NONE	gap called	83637244	83694746	13564	7.74E-23	34	29	INS	7.74E-23	
7-198	7	90562569	90594419	NONE	gap called	90562569	90594419	6065	7.74E-23	70	50	INS	7.74E-23	
7-207	7	93928563	93971645	NONE	gap called	93928563	93971645	5403	7.74E-23	93	61	INS	7.74E-23	
7-208	7	94106997	94163316	NONE	gap called	94106997	94163316	9521	7.74E-23	86	75	INS	7.74E-23	
7-210	7	94601916	94601916	NONE	gap called	94601916	94601916	8745	0.00E+00	74	0	INS	0.00E+00	
7-215	7	97956237	97983714	NONE	gap called	97956237	97983714	5517	7.74E-23	68	47	INS	7.74E-23	
7-228	7	101000872	101012499	NONE	gap called	101000872	101012499	7613	7.74E-23	60	46	INS	7.74E-23	
7-230	7	101643139	101679831	NONE	gap called	101643139	101679831	9133	7.74E-23	52	43	INS	7.74E-23	
7-233	7	106424350	106446809	NONE	gap called	106424350	106446809	6868	7.74E-23	54	45	INS	7.74E-23	
7-239	7	108302615	108345322	NONE	gap called	108302615	108345322	8649	7.74E-23	60	42	INS	7.74E-23	
7-260	7	120116663	120146785	NONE	gap called	120116663	120146785	7754	7.74E-23	64	51	INS	7.74E-23	

7-265	7	125281611	125330472	NONE	gap called	125281611	125330472	10342	7.74E-23	83	63	INS	7.74E-23
7-272	7	135207999	135268791	NONE	gap called	135207999	135268791	8324	7.74E-23	50	38	INS	7.74E-23
7-288	7	139674346	139738538	NONE	gap called	139674346	139738538	7156	9.45E-18	43	34	INS	9.45E-18
7-291	7	140292538	140336430	NONE	gap called	140292538	140336430	15290	1.09E-22	35	28	INS	1.09E-22
7-293	7	144021649	144158857	NONE	gap called	144021649	144158857	21828	1.04E-19	25	17	INS	1.04E-19
7-295	7	144340952	144384211	NONE	gap called	144340952	144384211	5355	3.82E-13	28	22	INS	3.82E-13
7-308	7	159500960	159515972	NONE	gap called	159500960	159515972	5329	7.74E-23	88	54	INS	7.74E-23
7-317	7	169427941	169427941	NONE	gap called	169427941	169427941	7878	0.00E+00	68	0	INS	0.00E+00
8-5	8	4996971	5039358	NONE	gap called	4996971	5039358	3301	2.47E-11	70	49	INS	2.47E-11
8-7	8	5121681	5183148	NONE	gap called	5121681	5183148	11921	6.97E-23	62	48	INS	6.97E-23
8-8	8	5298236	5311267	NONE	gap called	5298236	5311267	5883	6.97E-23	54	48	INS	6.97E-23
8-9	8	5542885	5545592	NONE	gap called	5542885	5545592	6734	8.70E-23	42	29	INS	8.70E-23
8-19	8	9048505	9100427	NONE	gap called	9048505	9100427	17568	6.97E-23	70	52	INS	6.97E-23
8-28	8	11383246	11398070	NONE	gap called	11383246	11398070	5028	6.97E-23	112	75	INS	6.97E-23
8-38	8	12729659	12826152	NONE	gap called	12729659	12826152	13056	8.68E-23	65	52	INS	8.68E-23
8-42	8	14009604	14022130	NONE	gap called	14009604	14022130	8094	6.97E-23	31	25	INS	6.97E-23
8-48	8	16256578	16256578	NONE	gap called	16256578	16256578	95319	0.00E+00	59	0	INS	0.00E+00
8-49	8	16256578	16267813	NONE	gap called	16256578	16267813	5623	6.97E-23	63	38	INS	6.97E-23
8-51	8	16956754	17034945	NONE	gap called	16956754	17034945	5693	1.58E-13	78	60	INS	1.58E-13
8-52	8	17048896	17096415	NONE	gap called	17048896	17096415	6515	6.97E-23	89	67	INS	6.97E-23
8-65	8	21300180	21311133	NONE	gap called	21300180	21311133	6788	6.97E-23	75	63	INS	6.97E-23
8-66	8	21504305	21548858	NONE	gap called	21504305	21548858	8065	6.97E-23	49	37	INS	6.97E-23
8-72	8	24035610	24035610	NONE	gap called	24035610	24035610	96240	0.00E+00	39	0	INS	0.00E+00
8-83	8	26726363	26741130	NONE	gap called	26726363	26741130	7372	6.97E-23	68	45	INS	6.97E-23
8-93	8	29189259	29217362	NONE	gap called	29189259	29217362	8192	6.97E-23	49	42	INS	6.97E-23
8-98	8	32797589	32855555	NONE	gap called	32797589	32855555	8182	6.97E-23	54	44	INS	6.97E-23
8-101	8	33303401	33320967	NONE	gap called	33303401	33320967	5337	6.97E-23	57	38	INS	6.97E-23
8-102	8	33334024	33373482	NONE	gap called	33334024	33373482	5841	6.97E-23	51	42	INS	6.97E-23
8-105	8	34696496	34788680	NONE	gap called	34696496	34788680	15801	2.54E-22	21	18	INS	2.54E-22
8-114	8	37815654	37815654	NONE	gap called	37815654	37815654	55299	0.00E+00	43	0	INS	0.00E+00
8-116	8	38213985	38213985	NONE	gap called	38213985	38213985	25261	0.00E+00	29	0	INS	0.00E+00
8-122	8	39449255	39472046	NONE	gap called	39449255	39472046	5039	6.97E-23	19	15	INS	6.97E-23
8-123	8	39603407	39652892	NONE	gap called	39603407	39652892	44994	8.70E-23	35	25	INS	8.70E-23
8-137	8	43606354	43673656	NONE	gap called	43606354	43673656	8036	6.97E-23	58	46	INS	6.97E-23
8-140	8	44429929	44429929	NONE	gap called	44429929	44429929	8831	0.00E+00	77	0	INS	0.00E+00
8-146	8	50511459	50547274	NONE	gap called	50511459	50547274	6367	8.59E-23	51	39	INS	8.59E-23
8-147	8	50587829	50654805	NONE	gap called	50587829	50654805	2500	9.61E-04	59	50	INS	9.61E-04
8-153	8	53410647	53438335	NONE	gap called	53410647	53438335	9067	6.97E-23	52	37	INS	6.97E-23
8-154	8	53801715	53862705	NONE	gap called	53801715	53862705	9749	2.54E-22	31	22	INS	2.54E-22
8-164	8	55969345	55994769	NONE	gap called	55969345	55994769	5311	6.97E-23	69	53	INS	6.97E-23
8-175	8	63320333	63345296	NONE	gap called	63320333	63345296	6526	6.97E-23	65	40	INS	6.97E-23
8-188	8	65860765	65860765	NONE	gap called	65860765	65860765	20772	0.00E+00	56	0	INS	0.00E+00
8-196	8	69817048	69844423	NONE	gap called	69817048	69844423	8604	6.97E-23	78	65	INS	6.97E-23
8-199	8	70361032	70406905	NONE	gap called	70361032	70406905	5023	5.42E-21	49	47	INS	5.42E-21
8-200	8	70671598	70690819	NONE	gap called	70671598	70690819	5521	6.97E-23	40	35	INS	6.97E-23
8-202	8	71417804	71434165	NONE	gap called	71417804	71434165	5778	6.97E-23	78	65	INS	6.97E-23
8-209	8	74013974	74085167	NONE	gap called	74013974	74085167	7728	7.36E-14	48	28	INS	7.36E-14
8-215	8	75979180	76033096	NONE	gap called	75979180	76033096	7827	6.97E-23	57	45	INS	6.97E-23
8-219	8	76821111	76856028	NONE	gap called	76821111	76856028	8204	6.97E-23	70	62	INS	6.97E-23
8-234	8	82568872	82616088	NONE	gap called	82568872	82616088	5901	6.97E-23	64	49	INS	6.97E-23
8-253	8	97518874	97570766	NONE	gap called	97518874	97570766	5864	2.07E-17	49	35	INS	2.07E-17
8-254	8	97658142	97705217	NONE	gap called	97658142	97705217	7642	6.97E-23	57	41	INS	6.97E-23
8-263	8	110573709	110630926	NONE	gap called	110573709	110630926	5922	3.53E-10	31	23	INS	3.53E-10
8-265	8	112449571	112492261	NONE	gap called	112449571	112492261	6528	6.97E-23	54	43	INS	6.97E-23
8-272	8	116171929	116208571	NONE	gap called	116171929	116208571	6323	6.97E-23	49	41	INS	6.97E-23
8-273	8	116277513	116321299	NONE	gap called	116277513	116321299	7179	6.97E-23	46	37	INS	6.97E-23
8-274	8	116562218	116626150	NONE	gap called	116562218	116626150	11046	2.35E-19	22	16	INS	2.35E-19
8-278	8	118775804	118791901	NONE	gap called	118775804	118791901	5868	6.97E-23	73	51	INS	6.97E-23
8-281	8	119543767	119564805	NONE	gap called	119543767	119564805	6588	6.97E-23	49	45	INS	6.97E-23
8-286	8	127143846	127143846	NONE	gap called	127143846	127143846	20642	0.00E+00	50	0	INS	0.00E+00
8-289	8	127761645	127772510	NONE	gap called	127761645	127772510	5804	6.97E-23	58	45	INS	6.97E-23
8-295	8	129112893	129150163	NONE	gap called	129112893	129150163	7470	6.97E-23	70	56	INS	6.97E-23
8-302	8	130493746	130566720	NONE	gap called	130493746	130566720	9658	6.97E-23	46	39	INS	6.97E-23
8-304	8	130758950	130766786	NONE	gap called	130758950	130766786	6599	6.97E-23	52	40	INS	6.97E-23
8-325	8	137340404	137440908	NONE	gap called	137340404	137440908	30953	6.97E-23	48	36	INS	6.97E-23
8-326	8	137440908	137440908	NONE	gap called	137440908	137440908	10934	0.00E+00	56	0	INS	0.00E+00
8-327	8	137661952	137673757	NONE	gap called	137661952	137673757	8508	6.97E-23	62	52	INS	6.97E-23
8-330	8	144858718	144928905	NONE	gap called	144858718	144928905	11807	6.97E-23	47	40	INS	6.97E-23
8-338	8	148787427	148830719	NONE	gap called	148787427	148830719	9017	6.97E-23	69	45	INS	6.97E-23
8-343	8	149791671	149836366	NONE	gap called	149791671	149836366	6099	6.97E-23	54	43	INS	6.97E-23
8-344	8	149957971	150009523	NONE	gap called	149957971	150009523	8010	6.97E-23	59	51	INS	6.97E-23
8-350	8	151071454	151100724	NONE	gap called	151071454	151100724	5590	6.97E-23	54	39	INS	6.97E-23
8-351	8	152262090	152308625	NONE	gap called	152262090	152308625	8259	6.97E-23	63	54	INS	6.97E-23
8-366	8	162830765	162891690	NONE	gap called	162830765	162891690	10351	6.97E-23	54	45	INS	6.97E-23
8-367	8	163027109	163040741	NONE	gap called	163027109	163040741	5575	6.97E-23	61	47	INS	6.97E-23
8-368	8	163671794	163688068	NONE	gap called	163671794	163688068	6898	6.97E-23	41	36	INS	6.97E-23
8-372	8	170464570	170464570	NONE	gap called	170464570	170464570	35023	0.00E+00	81	0	INS	0.00E+00
8-375	8	170962330	171025359	NONE	gap called	170962330	171025359	8924	6.97E-23	68	45	INS	6.97E-23
9-1	9	465299	492154	NONE	gap called	465299	492154	9222	7.33E-23	54	46	INS	7.33E-23
9-9	9	5679757	5755616	NONE	gap called	5679757	5755616	13769	7.33E-23	48	39	INS	7.33E-23
9-14	9	10382940	10425098	NONE	gap called	10382940	10425098	6642	7.33E-23	56	44	INS	7.33E-23
9-18	9	17691449	17691449	NONE	gap called	17691449	17691449	89728	0.00E+00	52	0	INS	0.00E+00
9-19	9	18688461	18767137	NONE	gap called	18688461	18767137	10352	7.33E-23	44	37	INS	7.33E-23
9-27	9	21489334	21489334	NONE	gap called	21489334	21489334	37714	0.00E+00	84	0	INS	0.00E+00
9-30	9	21872482	21872482	NONE	gap called	21872482	21872482	14171	0.00E+00	85	0	INS	0.00E+00
9-50	9	33975418	33994993	NONE	gap called	33975418	33994993	5273	7.33E-23	97	81	INS	7.33E-23
9-57	9	36583180	36583180	NONE	gap called	36583180	36583180	20046	0.00E+00	52	0	INS	0.00E+00
9-74	9	42847485	42847485	NONE	gap called	42847485	42847485	17782	0.00E+00	60	0	INS	0.00E+00
9-76	9	43063644	43093414	NONE	gap called	43063644	43093414	16442	7.33E-23	44	37	INS	7.33E-23
9-79	9	43577040	43625687	NONE	gap called	43577040	43625687	5912	7.33E-23	65	45	INS	7.33E-23
9-85	9	48990498	49006428	NONE	gap called	48990498	49006428	8191	7.33E-23	76	57	INS	7.33E-23

9-90	9	50164796	50164796	NONE	gap called	50164796	50164796	10593	0.00E+00	47	0	INS	0.00E+00
9-93	9	52705505	52738677	NONE	gap called	52705505	52738677	6291	7.33E-23	69	60	INS	7.33E-23
9-94	9	52782747	52833515	NONE	gap called	52782747	52833515	5985	7.33E-23	64	52	INS	7.33E-23
9-99	9	55056046	55071185	NONE	gap called	55056046	55071185	7945	7.33E-23	57	47	INS	7.33E-23
9-103	9	56149597	56190180	NONE	gap called	56149597	56190180	5540	7.33E-23	56	42	INS	7.33E-23
9-105	9	56901345	56954562	NONE	gap called	56901345	56954562	6030	1.51E-20	58	40	INS	1.51E-20
9-111	9	59670880	59670880	NONE	gap called	59670880	59670880	7545	0.00E+00	61	0	INS	0.00E+00
9-127	9	67177843	67192685	NONE	gap called	67177843	67192685	8721	7.33E-23	84	67	INS	7.33E-23
9-136	9	70076185	70076185	NONE	gap called	70076185	70076185	12888	0.00E+00	57	0	INS	0.00E+00
9-142	9	71337510	71363585	NONE	gap called	71337510	71363585	6782	7.33E-23	52	43	INS	7.33E-23
9-155	9	75575632	75651117	NONE	gap called	75575632	75651117	7069	2.84E-07	18	18	INS	2.84E-07
9-159	9	76380115	76424998	NONE	gap called	76380115	76424998	7577	7.33E-23	62	45	INS	7.33E-23
9-168	9	80244780	80251217	NONE	gap called	80244780	80251217	13582	7.33E-23	69	59	INS	7.33E-23
9-172	9	81135082	81135082	NONE	gap called	81135082	81135082	10862	0.00E+00	73	0	INS	0.00E+00
9-174	9	81586458	81685431	NONE	gap called	81586458	81685431	10781	6.48E-12	28	22	INS	6.48E-12
9-190	9	87680798	87733239	NONE	gap called	87680798	87733239	3614	2.62E-11	70	57	INS	2.62E-11
9-192	9	87846509	87846509	NONE	gap called	87846509	87846509	11014	0.00E+00	89	0	INS	0.00E+00
9-199	9	90116594	90155560	NONE	gap called	90116594	90155560	7192	7.33E-23	71	49	INS	7.33E-23
9-214	9	93824913	93862623	NONE	gap called	93824913	93862623	2320	5.53E-08	71	52	INS	5.53E-08
9-215	9	94368403	94441119	NONE	gap called	94368403	94441119	7383	7.33E-23	65	57	INS	7.33E-23
9-217	9	94734593	94734593	NONE	gap called	94734593	94734593	76163	0.00E+00	70	0	INS	0.00E+00
9-219	9	95122824	95185806	NONE	gap called	95122824	95185806	17196	1.05E-22	31	25	INS	1.05E-22
9-222	9	95653884	95653884	NONE	gap called	95653884	95653884	8624	0.00E+00	76	0	INS	0.00E+00
9-225	9	96146734	96188706	NONE	gap called	96146734	96188706	11421	7.33E-23	80	61	INS	7.33E-23
9-228	9	96791153	96897003	NONE	gap called	96791153	96897003	20308	7.33E-23	40	34	INS	7.33E-23
9-230	9	97039773	97105392	NONE	gap called	97039773	97105392	11318	7.33E-23	37	36	INS	7.33E-23
9-235	9	98074237	98142822	NONE	gap called	98074237	98142822	6996	2.18E-17	48	40	INS	2.18E-17
9-238	9	98505389	98510518	NONE	gap called	98505389	98510518	4261	1.03E-22	62	39	INS	1.03E-22
9-246	9	103446339	103496639	NONE	gap called	103446339	103496639	11754	7.33E-23	52	40	INS	7.33E-23
9-247	9	103605663	103642148	NONE	gap called	103605663	103642148	5480	7.33E-23	47	39	INS	7.33E-23
9-248	9	103685281	103714255	NONE	gap called	103685281	103714255	5678	7.33E-23	40	31	INS	7.33E-23
9-249	9	106227695	106298259	NONE	gap called	106227695	106298259	7660	7.33E-23	64	48	INS	7.33E-23
9-257	9	113333171	113359889	NONE	gap called	113333171	113359889	4672	1.04E-22	67	52	INS	1.04E-22
9-263	9	114314861	114413230	NONE	gap called	114314861	114413230	16714	1.02E-22	26	19	INS	1.02E-22
9-270	9	119177159	119228639	NONE	gap called	119177159	119228639	14372	7.33E-23	51	42	INS	7.33E-23
9-272	9	119956340	119989145	NONE	gap called	119956340	119989145	6836	7.33E-23	47	40	INS	7.33E-23
9-282	9	131192772	131249785	NONE	gap called	131192772	131249785	20173	7.33E-23	19	15	INS	7.33E-23
9-297	9	135999055	136013153	NONE	gap called	135999055	136013153	7111	7.33E-23	62	49	INS	7.33E-23
9-298	9	136159869	136197783	NONE	gap called	136159869	136197783	10617	7.33E-23	72	53	INS	7.33E-23
9-299	9	136300377	136323187	NONE	gap called	136300377	136323187	6886	7.33E-23	85	61	INS	7.33E-23
9-302	9	137019431	137043720	NONE	gap called	137019431	137043720	5063	7.33E-23	70	46	INS	7.33E-23
9-309	9	138549921	138588573	NONE	gap called	138549921	138588573	7580	7.33E-23	38	29	INS	7.33E-23
9-313	9	139526353	139564244	NONE	gap called	139526353	139564244	7400	7.33E-23	57	49	INS	7.33E-23
9-319	9	141737080	141782681	NONE	gap called	141737080	141782681	26143	1.01E-22	41	29	INS	1.01E-22
9-327	9	144049258	144049258	NONE	gap called	144049258	144049258	56683	0.00E+00	55	0	INS	0.00E+00
9-348	9	150383836	150383836	NONE	gap called	150383836	150383836	68404	0.00E+00	67	0	INS	0.00E+00
10-3	10	582675	613836	NONE	gap called	582675	613836	8855	7.28E-23	87	63	INS	7.28E-23
10-6	10	943926	943926	NONE	gap called	943926	943926	8517	0.00E+00	94	0	INS	0.00E+00
10-11	10	3028862	3059192	NONE	gap called	3028862	3059192	4401	7.28E-23	78	58	INS	7.28E-23
10-15	10	4470979	4594188	NONE	gap called	4470979	4594188	17347	3.83E-20	28	22	INS	3.83E-20
10-22	10	9396295	9445431	NONE	gap called	9396295	9445431	10692	7.28E-23	65	60	INS	7.28E-23
10-25	10	9971488	9979947	NONE	gap called	9971488	9979947	5207	7.28E-23	86	67	INS	7.28E-23
10-29	10	10666176	10709109	NONE	gap called	10666176	10709109	8196	7.28E-23	62	53	INS	7.28E-23
10-35	10	16470286	16488835	NONE	gap called	16470286	16488835	6212	7.28E-23	55	44	INS	7.28E-23
10-39	10	17163413	17163413	NONE	gap called	17163413	17163413	20418	0.00E+00	76	0	INS	0.00E+00
10-41	10	18319332	18359693	NONE	gap called	18319332	18359693	3792	7.90E-14	49	40	INS	7.90E-14
10-43	10	18556000	18578234	NONE	gap called	18556000	18578234	13396	7.28E-23	45	33	INS	7.28E-23
10-45	10	18789616	18897046	NONE	gap called	18789616	18897046	5721	5.12E-04	31	24	INS	5.12E-04
10-47	10	19115115	19115115	NONE	gap called	19115115	19115115	128294	0.00E+00	42	0	INS	0.00E+00
10-53	10	24334091	24429877	NONE	gap called	24334091	24429877	17572	7.28E-23	28	19	INS	7.28E-23
10-55	10	25824615	25863072	NONE	gap called	25824615	25863072	7865	7.28E-23	77	62	INS	7.28E-23
10-71	10	32004394	32043509	NONE	gap called	32004394	32043509	7235	7.28E-23	74	54	INS	7.28E-23
10-75	10	35605312	35630696	NONE	gap called	35605312	35630696	6685	7.28E-23	46	35	INS	7.28E-23
10-76	10	36153339	36190009	NONE	gap called	36153339	36190009	11080	7.28E-23	31	25	INS	7.28E-23
10-82	10	41948163	41948163	NONE	gap called	41948163	41948163	7841	0.00E+00	59	0	INS	0.00E+00
10-86	10	42514907	42604437	NONE	gap called	42514907	42604437	10205	7.79E-22	58	38	INS	7.79E-22
10-91	10	46214504	46229883	NONE	gap called	46214504	46229883	6460	7.28E-23	52	44	INS	7.28E-23
10-92	10	46494545	46537748	NONE	gap called	46494545	46537748	22388	7.28E-23	48	36	INS	7.28E-23
10-94	10	46753895	46774942	NONE	gap called	46753895	46774942	7275	7.28E-23	54	44	INS	7.28E-23
10-105	10	53142434	53169455	NONE	gap called	53142434	53169455	9932	7.28E-23	49	34	INS	7.28E-23
10-108	10	53571055	53607697	NONE	gap called	53571055	53607697	11655	7.28E-23	48	40	INS	7.28E-23
10-110	10	54064292	54107756	NONE	gap called	54064292	54107756	10737	7.28E-23	58	42	INS	7.28E-23
10-131	10	61329910	61370540	NONE	gap called	61329910	61370540	8351	7.28E-23	64	48	INS	7.28E-23
10-152	10	72544635	72575873	NONE	gap called	72544635	72575873	7177	7.28E-23	41	34	INS	7.28E-23
10-154	10	73073263	73109913	NONE	gap called	73073263	73109913	6501	7.28E-23	68	59	INS	7.28E-23
10-157	10	73901539	73972356	NONE	gap called	73901539	73972356	7197	7.19E-10	30	21	INS	7.19E-10
10-159	10	74129629	74214062	NONE	gap called	74129629	74214062	15231	7.28E-23	49	32	INS	7.28E-23
10-162	10	75010539	75051999	NONE	gap called	75010539	75051999	8886	7.28E-23	41	33	INS	7.28E-23
10-163	10	75417885	75463249	NONE	gap called	75417885	75463249	5565	7.28E-23	56	50	INS	7.28E-23
10-165	10	75712214	75767290	NONE	gap called	75712214	75767290	11113	7.28E-23	68	46	INS	7.28E-23
10-168	10	76273228	76306402	NONE	gap called	76273228	76306402	8025	7.28E-23	48	33	INS	7.28E-23
10-172	10	77124202	77124202	NONE	gap called	77124202	77124202	35761	0.00E+00	38	0	INS	0.00E+00
10-173	10	77254326	77254326	NONE	gap called	77254326	77254326	27050	0.00E+00	54	0	INS	0.00E+00
10-185	10	83823289	83830304	NONE	gap called	83823289	83830304	6102	7.28E-23	36	26	INS	7.28E-23
10-193	10	86924238	86943962	NONE	gap called	86924238	86943962	7982	7.28E-23	53	43	INS	7.28E-23
10-197	10	90877689	90911096	NONE	gap called	90877689	90911096	9014	7.28E-23	47	37	INS	7.28E-23
10-199	10	92219357	92219357	NONE	gap called	92219357	92219357	33005	0.00E+00	81	0	INS	0.00E+00
10-206	10	97618054	97649884	NONE	gap called	97618054	97649884	11445	7.28E-23	56	42	INS	7.28E-23
10-211	10	99850435	99883100	NONE	gap called	99850435	99883100	8869	7.28E-23	40	31	INS	7.28E-23
10-214	10	101797578	101808506	NONE	gap called	101797578	101808506	6124	7.28E-23	89	78	INS	7.28E-23
10-218	10	103462709	103462709	NONE	gap called	103462709	103462709	119445	0.00E+00	45	0	INS	0.00E+00

10-219	10	103578560	103644561	NONE	gap called	103578560	103644561	12605	7.28E-23	56	51	INS	7.28E-23	
10-225	10	105047950	105047950	NONE	gap called	105047950	105047950	36910	0.00E+00	47	0	INS	0.00E+00	
10-227	10	105389485	105389485	NONE	gap called	105389485	105389485	77871	0.00E+00	67	0	INS	0.00E+00	
10-230	10	105727793	105827543	NONE	gap called	105727793	105827543	14284	7.28E-23	54	40	INS	7.28E-23	
10-233	10	106842418	106880595	NONE	gap called	106842418	106880595	7688	7.28E-23	59	41	INS	7.28E-23	
10-238	10	107559358	107587892	NONE	gap called	107559358	107587892	5226	7.28E-23	85	63	INS	7.28E-23	
10-239	10	107819309	107828188	NONE	gap called	107819309	107828188	8395	7.28E-23	77	56	INS	7.28E-23	
10-242	10	108349909	108381565	NONE	gap called	108349909	108381565	5945	7.28E-23	79	57	INS	7.28E-23	
10-243	10	108393396	108426567	NONE	gap called	108393396	108426567	7951	7.28E-23	77	54	INS	7.28E-23	
10-252	10	110434668	110447580	NONE	gap called	110434668	110447580	5836	7.28E-23	59	43	INS	7.28E-23	
10-253	10	110447580	110464324	NONE	gap called	110447580	110464324	5879	7.28E-23	56	45	INS	7.28E-23	
10-256	10	110766032	110766032	NONE	gap called	110766032	110766032	63198	0.00E+00	50	0	INS	0.00E+00	
10-263	10	112642883	112642883	NONE	gap called	112642883	112642883	18253	0.00E+00	70	0	INS	0.00E+00	
10-269	10	113403028	113428811	NONE	gap called	113403028	113428811	10313	7.28E-23	48	42	INS	7.28E-23	
10-272	10	113894230	113944586	NONE	gap called	113894230	113944586	16575	7.28E-23	49	38	INS	7.28E-23	
10-284	10	116598255	116598255	NONE	gap called	116598255	116598255	20598	0.00E+00	66	0	INS	0.00E+00	
10-290	10	117390093	117432823	NONE	gap called	117390093	117432823	12880	7.28E-23	68	53	INS	7.28E-23	
10-291	10	117690971	117702944	NONE	gap called	117690971	117702944	5022	7.28E-23	77	66	INS	7.28E-23	
10-293	10	117908569	117908569	NONE	gap called	117908569	117908569	15953	0.00E+00	59	0	INS	0.00E+00	
10-295	10	118193542	118205014	NONE	gap called	118193542	118205014	5027	7.28E-23	51	41	INS	7.28E-23	
10-300	10	120188710	120188710	NONE	gap called	120188710	120188710	76432	0.00E+00	54	0	INS	0.00E+00	
10-305	10	127078375	127118159	NONE	gap called	127078375	127118159	6301	7.28E-23	60	47	INS	7.28E-23	
10-308	10	127407526	127497548	NONE	gap called	127407526	127497548	15905	7.28E-23	41	30	INS	7.28E-23	
10-314	10	135864361	135924666	NONE	gap called	135864361	135924666	7945	7.28E-23	44	37	INS	7.28E-23	
10-319	10	137628275	137628275	NONE	gap called	137628275	137628275	40058	0.00E+00	60	0	INS	0.00E+00	
10-322	10	142251775	142291948	NONE	gap called	142251775	142291948	8830	7.28E-23	60	47	INS	7.28E-23	
10-328	10	149051997	149051997	NONE	gap called	149051997	149051997	7458	0.00E+00	64	0	INS	0.00E+00	
5-378	5	140171711	140200217	Misassembly	gap called	140171711	140200217	6111	7.30E-23	49	35	INS	7.30E-23	complex event
7-99	7	49411926	49435539	Misassembly	gap called	49411926	49435539	10862	1.08E-22	62	52	INS	1.08E-22	complex event - possible inversion
1-1	1	1933290	2008849	Misassembly	NONE	1933290	2008849	0				OTHER		Complex event
1-6	1	2693118	2825334	Misassembly	NONE	2693118	2825334	0				OTHER		insertion and rearrangement
1-10	1	5429636	5479603	Misassembly	NONE	5429636	5479603	0				OTHER		complex event
1-12	1	7312238	7376813	Misassembly	NONE	7312238	7376813	0				OTHER		complex event
1-13	1	7435795	7555989	Misassembly	NONE	7435795	7555989	0				OTHER		complex event
1-16	1	8263233	8426279	Misassembly	NONE	8263233	8426279	0				OTHER		possible inversion
1-21	1	8819988	8853281	Misassembly	NONE	8819988	8853281	0				OTHER		Complex event
1-23	1	9198019	9368760	Misassembly	NONE	9198019	9368760	0				OTHER		Insertion and complex event
1-30	1	10332683	10544151	Misassembly	NONE	10332683	10544151	0				OTHER		complex event
1-31	1	10622313	10767316	Misassembly	NONE	10622313	10767316	0				OTHER		complex event
1-33	1	11391442	11459014	Misassembly	NONE	11391442	11459014	0				OTHER		complex event
1-34	1	11715496	11760538	Misassembly	NONE	11715496	11760538	0				OTHER		complex event
1-43	1	12831282	13007173	Misassembly	NONE	12831282	13007173	0				OTHER		complex event
1-44	1	13072492	13287408	Misassembly	NONE	13072492	13287408	0				OTHER		complex event
1-47	1	13494371	13590640	Misassembly	NONE	13494371	13590640	0				OTHER		complex event possible inversion
1-48	1	13653475	13690277	Misassembly	NONE	13653475	13690277	0				OTHER		complex event
1-54	1	14430908	14597145	Misassembly	NONE	14430908	14597145	0				OTHER		complex event
1-58	1	15371796	15548474	Misassembly	NONE	15371796	15548474	0				OTHER		complex event
1-62	1	16225318	16436580	Misassembly	NONE	16225318	16436580	0				OTHER		complex event
1-65	1	16791695	16953906	Misassembly	NONE	16791695	16953906	0				OTHER		complex event
1-66	1	17060199	17350142	Misassembly	NONE	17060199	17350142	0				OTHER		complex event
1-71	1	18005003	18067612	Misassembly	NONE	18005003	18067612	0				OTHER		complex event
1-72	1	18102903	18267922	Misassembly	NONE	18102903	18267922	0				OTHER		complex event
1-74	1	18584003	18716231	Misassembly	NONE	18584003	18716231	0				OTHER		complex event
1-75	1	18902381	19012632	Misassembly	NONE	18902381	19012632	0				OTHER		complex event
1-78	1	23036941	23132267	Misassembly	NONE	23036941	23132267	0				OTHER		complex event possible inversion
1-79	1	23149366	23242592	Misassembly	NONE	23149366	23242592	0				OTHER		complex event
1-81	1	24486335	24568740	Misassembly	NONE	24486335	24568740	0				OTHER		complex event
1-82	1	28740973	28889944	Misassembly	NONE	28740973	28889944	0				OTHER		complex event
1-86	1	29478352	29534044	Misassembly	NONE	29478352	29534044	0				OTHER		complex event
1-88	1	31249867	31413432	Misassembly	NONE	31249867	31413432	0				OTHER		complex event
1-92	1	33992693	34147716	Misassembly	NONE	33992693	34147716	0				OTHER		complex event
1-93	1	34189304	34290760	Misassembly	NONE	34189304	34290760	0				OTHER		complex event
1-94	1	34412635	34703756	Misassembly	NONE	34412635	34703756	0				OTHER		complex event
1-95	1	34836292	34956014	Misassembly	NONE	34836292	34956014	0				OTHER		complex event
1-96	1	35114846	35172662	Misassembly	NONE	35114846	35172662	0				OTHER		complex event
1-97	1	35338500	35486733	Misassembly	NONE	35338500	35486733	0				OTHER		complex event
1-98	1	35536540	35639787	Misassembly	NONE	35536540	35639787	0				OTHER		complex event
1-101	1	36301873	36420071	Misassembly	NONE	36301873	36420071	0				OTHER		complex event
1-103	1	36561932	36736029	Misassembly	NONE	36561932	36736029	0				OTHER		complex event
1-107	1	37345881	37533048	Misassembly	NONE	37345881	37533048	0				OTHER		complex event
1-108	1	37748324	37867594	Misassembly	NONE	37748324	37867594	0				OTHER		complex event
1-109	1	37926819	38023473	Misassembly	NONE	37926819	38023473	0				OTHER		complex event
1-110	1	42350073	42367673	Misassembly	NONE	42350073	42367673	0				OTHER		complex event possible inversion
1-117	1	46132660	46264341	Misassembly	NONE	46132660	46264341	0				OTHER		complex event
1-119	1	46962608	47032992	Misassembly	NONE	46962608	47032992	0				OTHER		complex event
1-132	1	54428633	54586585	Misassembly	NONE	54428633	54586585	0				OTHER		complex event
1-133	1	54743210	54835350	Misassembly	NONE	54743210	54835350	0				OTHER		complex event
1-134	1	55250919	55469676	Misassembly	NONE	55250919	55469676	0				OTHER		complex event
1-136	1	55910779	55999209	Misassembly	NONE	55910779	55999209	0				OTHER		complex event
1-137	1	56075804	56271059	Misassembly	NONE	56075804	56271059	0				OTHER		complex event

1-138	1	56327842	56421589	Misassembly	NONE	56327842	56421589		0					OTHER		complex event
1-139	1	56620487	56825015	Misassembly	NONE	56620487	56825015		0					OTHER		complex event
1-147	1	57734742	57829183	Misassembly	NONE	57734742	57829183		0					OTHER		complex event
1-151	1	58348655	58366936	Misassembly	NONE	58348655	58366936		0					OTHER		complex event possible inversion
1-154	1	58560929	58810816	Misassembly	NONE	58560929	58810816		0					OTHER		complex event
1-155	1	59154850	59315548	Misassembly	NONE	59154850	59315548		0					OTHER		complex event
1-160	1	60026034	60113111	Misassembly	NONE	60026034	60113111		0					OTHER		complex event
1-161	1	60389185	60612187	Misassembly	NONE	60389185	60612187		0					OTHER		complex event
1-162	1	60654784	60991408	Misassembly	NONE	60654784	60991408		0					OTHER		complex event
1-163	1	61071278	61107362	Misassembly	NONE	61071278	61107362		0					OTHER		complex event
1-164	1	61224064	61340567	Misassembly	NONE	61224064	61340567		0					OTHER		complex event
1-172	1	62554868	63027265	Misassembly	NONE	62554868	63027265		0					OTHER		complex event
1-173	1	63176314	63395314	Misassembly	NONE	63176314	63395314		0					OTHER		complex event
1-180	1	64354710	64484292	Misassembly	NONE	64354710	64484292		0					OTHER		complex event
1-182	1	65916462	65989745	Misassembly	NONE	65916462	65989745		0					OTHER		complex event
1-184	1	66277144	66328752	Misassembly	NONE	66277144	66328752		0					OTHER		complex event possible inversion
1-187	1	69011478	69091823	Misassembly	NONE	69011478	69091823		0					OTHER		complex event
1-188	1	69240334	69311359	Misassembly	NONE	69240334	69311359		0					OTHER		complex event
1-189	1	69721950	69861541	Misassembly	NONE	69721950	69861541		0					OTHER		complex event
1-190	1	70038055	70099736	Misassembly	NONE	70038055	70099736		0					OTHER		complex event
1-191	1	71505542	71612977	Misassembly	NONE	71505542	71612977		0					OTHER		complex event
1-193	1	72196524	72279713	Misassembly	NONE	72196524	72279713		0					OTHER		complex event possible inversion
1-195	1	72367517	72423703	Misassembly	NONE	72367517	72423703		0					OTHER		complex event
1-196	1	72826227	72931300	Misassembly	NONE	72826227	72931300		0					OTHER		complex event
1-197	1	72994209	73027678	Misassembly	NONE	72994209	73027678		0					OTHER		complex event
1-198	1	73166303	73457298	Misassembly	NONE	73166303	73457298		0					OTHER		complex event
1-199	1	73540012	73766212	Misassembly	NONE	73540012	73766212		0					OTHER		complex event
1-200	1	74057928	74109471	Misassembly	NONE	74057928	74109471		0					OTHER		complex event
1-202	1	74388679	74531640	Misassembly	NONE	74388679	74531640		0					OTHER		complex event
1-203	1	74599439	74656137	Misassembly	NONE	74599439	74656137		0					OTHER		complex event possible inversion
1-206	1	74965341	75001976	Misassembly	NONE	74965341	75001976		0					OTHER		complex event
1-208	1	75267032	75402679	Misassembly	NONE	75267032	75402679		0					OTHER		complex event
1-209	1	75427796	75533898	Misassembly	NONE	75427796	75533898		0					OTHER		complex event
1-212	1	76033279	76214258	Misassembly	NONE	76033279	76214258		0					OTHER		complex event
1-219	1	79792930	79810016	Misassembly	NONE	79792930	79810016		0					OTHER		complex event
1-220	1	79979584	80204215	Misassembly	NONE	79979584	80204215		0					OTHER		complex event
1-221	1	80355939	80394928	Misassembly	NONE	80355939	80394928		0					OTHER		complex event
1-223	1	80587669	80662296	Misassembly	NONE	80587669	80662296		0					OTHER		complex event
1-226	1	80991993	81147108	Misassembly	NONE	80991993	81147108		0					OTHER		complex event
1-227	1	81408384	81468850	Misassembly	NONE	81408384	81468850		0					OTHER		complex event
1-228	1	81641589	81791158	Misassembly	NONE	81641589	81791158		0					OTHER		complex event
1-229	1	81828553	82110933	Misassembly	NONE	81828553	82110933		0					OTHER		complex event
1-231	1	82436384	82567732	Misassembly	NONE	82436384	82567732		0					OTHER		complex event
1-232	1	82654471	82951281	Misassembly	NONE	82654471	82951281		0					OTHER		complex event
1-235	1	84208966	84338152	Misassembly	NONE	84208966	84338152		0					OTHER		complex event
1-236	1	84424615	84452503	Misassembly	NONE	84424615	84452503		0					OTHER		complex event
1-237	1	84576767	84689129	Misassembly	NONE	84576767	84689129		0					OTHER		complex event
1-240	1	85778389	85873451	Misassembly	NONE	85778389	85873451		0					OTHER		complex event
1-241	1	87593507	87702146	Misassembly	NONE	87593507	87702146		0					OTHER		complex event
1-249	1	88857201	88935155	Misassembly	NONE	88857201	88935155		0					OTHER		complex event
1-250	1	88996398	89062571	Misassembly	NONE	88996398	89062571		0					OTHER		complex event
1-253	1	89312497	89383994	Misassembly	NONE	89312497	89383994		0					OTHER		complex event
1-258	1	90759036	91079531	Misassembly	NONE	90759036	91079531		0					OTHER		complex event
1-259	1	91141354	91198763	Misassembly	NONE	91141354	91198763		0					OTHER		complex event
1-260	1	91518315	91750254	Misassembly	NONE	91518315	91750254		0					OTHER		complex event
1-261	1	91803534	91907777	Misassembly	NONE	91803534	91907777		0					OTHER		complex event
1-262	1	91991312	92288561	Misassembly	NONE	91991312	92288561		0					OTHER		complex event
1-263	1	92316256	92543818	Misassembly	NONE	92316256	92543818		0					OTHER		complex event
1-264	1	92703188	92718690	Misassembly	NONE	92703188	92718690		0					OTHER		complex event
1-266	1	92953876	93090404	Misassembly	NONE	92953876	93090404		0					OTHER		complex event
1-270	1	94026590	94135349	Misassembly	NONE	94026590	94135349		0					OTHER		complex event
1-271	1	95301341	95406092	Misassembly	NONE	95301341	95406092		0					OTHER		complex event
1-277	1	96398217	96547165	Misassembly	NONE	96398217	96547165		0					OTHER		complex event
1-278	1	98105045	98352424	Misassembly	NONE	98105045	98352424		0					OTHER		complex event
1-279	1	98515689	98557112	Misassembly	NONE	98515689	98557112		0					OTHER		complex event
1-280	1	99416692	99656255	Misassembly	NONE	99416692	99656255		0					OTHER		complex event
1-281	1	99858342	99936676	Misassembly	NONE	99858342	99936676		0					OTHER		complex event
1-282	1	100265870	100357510	Misassembly	NONE	100265870	100357510		0					OTHER		complex event
1-286	1	100735770	100837212	Misassembly	NONE	100735770	100837212		0					OTHER		complex event
1-287	1	101025050	101162167	Misassembly	NONE	101025050	101162167		0					OTHER		complex event
1-289	1	101936749	102088892	Misassembly	NONE	101936749	102088892		0					OTHER		complex event
1-293	1	102545985	102650126	Misassembly	NONE	102545985	102650126		0					OTHER		complex event
1-300	1	103990324	104118585	Misassembly	NONE	103990324	104118585		0					OTHER		complex event
1-301	1	104174677	104193787	Misassembly	NONE	104174677	104193787		0					OTHER		complex event
1-312	1	105614983	105727653	Misassembly	NONE	105614983	105727653		0					OTHER		complex event
1-314	1	105800730	105897654	Misassembly	NONE	105800730	105897654		0					OTHER		complex event
1-315	1	106048381	106103251	Misassembly	NONE	106048381	106103251		0					OTHER		complex event
1-319	1	108172662	108275685	Misassembly	NONE	108172662	108275685		0					OTHER		complex event
1-321	1	108442659	108502327	Misassembly	NONE	108442659	108502327		0					OTHER		complex event
1-322	1	108710058	108726676	Misassembly	NONE	108710058	108726676		0					OTHER		complex event
1-323	1	109199614	109305823	Misassembly	NONE	109199614	109305823		0					OTHER		complex event
1-325	1	109773646	109932625	Misassembly	NONE	109773646	109932625		0					OTHER		complex event
1-330	1	110429330	110559940	Misassembly	NONE	110429330	110559940		0					OTHER		complex event

1-335	1	111444806	111540140	Misassembly	NONE	111444806	111540140		0					OTHER		complex event
1-341	1	112325908	112410332	Misassembly	NONE	112325908	112410332		0					OTHER		complex event
1-346	1	112971035	113141522	Misassembly	NONE	112971035	113141522		0					OTHER		complex event
1-348	1	113564661	113614043	Misassembly	NONE	113564661	113614043		0					OTHER		complex event
1-349	1	113805855	113933627	Misassembly	NONE	113805855	113933627		0					OTHER		complex event
1-352	1	114671347	114798369	Misassembly	NONE	114671347	114798369		0					OTHER		complex event
1-354	1	115000794	115117452	Misassembly	NONE	115000794	115117452		0					OTHER		complex event
1-356	1	117051116	117196918	Misassembly	NONE	117051116	117196918		0					OTHER		complex event
1-357	1	117534019	117710708	Misassembly	NONE	117534019	117710708		0					OTHER		complex event
1-361	1	118407473	118513164	Misassembly	NONE	118407473	118513164		0					OTHER		complex event
1-362	1	118838021	118896763	Misassembly	NONE	118838021	118896763		0					OTHER		complex event
1-364	1	119004682	119061334	Misassembly	NONE	119004682	119061334		0					OTHER		complex event
1-368	1	120142236	120219337	Misassembly	NONE	120142236	120219337		0					OTHER		complex event
1-369	1	120311824	120536033	Misassembly	NONE	120311824	120536033		0					OTHER		complex event
1-370	1	120846842	121100839	Misassembly	NONE	120846842	121100839		0					OTHER		complex event
1-373	1	121684962	121838077	Misassembly	NONE	121684962	121838077		0					OTHER		complex event
1-374	1	122198403	122331538	Misassembly	NONE	122198403	122331538		0					OTHER		complex event
1-375	1	122534416	122599017	Misassembly	NONE	122534416	122599017		0					OTHER		complex event
1-376	1	122808172	122906183	Misassembly	NONE	122808172	122906183		0					OTHER		complex event
1-377	1	123069184	123251623	Misassembly	NONE	123069184	123251623		0					OTHER		complex event
1-378	1	123490715	123834043	Misassembly	NONE	123490715	123834043		0					OTHER		complex event
1-380	1	123995718	124070758	Misassembly	NONE	123995718	124070758		0					OTHER		complex event
1-382	1	124470505	124554078	Misassembly	NONE	124470505	124554078		0					OTHER		complex event
1-383	1	124955551	125063686	Misassembly	NONE	124955551	125063686		0					OTHER		complex event
1-384	1	125409930	125515063	Misassembly	NONE	125409930	125515063		0					OTHER		complex event
1-385	1	125947562	126067323	Misassembly	NONE	125947562	126067323		0					OTHER		complex event with insertion
1-386	1	126355406	126414814	Misassembly	NONE	126355406	126414814		0					OTHER		complex event
1-387	1	126496919	126603904	Misassembly	NONE	126496919	126603904		0					OTHER		complex event
1-393	1	127432905	127625432	Misassembly	NONE	127432905	127625432		0					OTHER		complex event
1-394	1	127704626	127790026	Misassembly	NONE	127704626	127790026		0					OTHER		complex event with insertion
1-395	1	128349528	128690639	Misassembly	NONE	128349528	128690639		0					OTHER		complex event
1-396	1	128959198	129155825	Misassembly	NONE	128959198	129155825		0					OTHER		complex event
1-398	1	129905763	129995956	Misassembly	NONE	129905763	129995956		0					OTHER		complex event
1-401	1	130671191	130927190	Misassembly	NONE	130671191	130927190		0					OTHER		complex event
1-402	1	130972349	131041644	Misassembly	NONE	130972349	131041644		0					OTHER		complex event
1-406	1	134215577	134400044	Misassembly	NONE	134215577	134400044		0					OTHER		complex event
1-407	1	134703627	135105161	Misassembly	NONE	134703627	135105161		0					OTHER		complex event
1-408	1	135185406	135270503	Misassembly	NONE	135185406	135270503		0					OTHER		complex event possible inversion
1-411	1	135568026	135688759	Misassembly	NONE	135568026	135688759		0					OTHER		complex event
1-414	1	137819007	137871764	Misassembly	NONE	137819007	137871764		0					OTHER		complex event
1-416	1	139963805	140194573	Misassembly	NONE	139963805	140194573		0					OTHER		complex event
1-417	1	140245278	140337381	Misassembly	NONE	140245278	140337381		0					OTHER		complex event
1-422	1	141268622	141437306	Misassembly	NONE	141268622	141437306		0					OTHER		complex event
1-430	1	143630812	143704868	Misassembly	NONE	143630812	143704868		0					OTHER		complex event
1-432	1	143821533	144050456	Misassembly	NONE	143821533	144050456		0					OTHER		complex event
1-433	1	144314516	144427835	Misassembly	NONE	144314516	144427835		0					OTHER		complex event
1-436	1	145019076	145166074	Misassembly	NONE	145019076	145166074		0					OTHER		complex event
1-438	1	145617469	145753032	Misassembly	NONE	145617469	145753032		0					OTHER		complex event
1-439	1	145829732	145945163	Misassembly	NONE	145829732	145945163		0					OTHER		complex event
1-440	1	146329488	146431111	Misassembly	NONE	146329488	146431111		0					OTHER		complex event
1-444	1	149905606	149938835	Misassembly	NONE	149905606	149938835		0					OTHER		complex event
1-445	1	153147590	153209501	Misassembly	NONE	153147590	153209501		0					OTHER		complex event
1-446	1	153528908	153572953	Misassembly	NONE	153528908	153572953		0					OTHER		complex event
1-450	1	154234067	154326571	Misassembly	NONE	154234067	154326571		0					OTHER		complex event
1-453	1	154531256	154726330	Misassembly	NONE	154531256	154726330		0					OTHER		complex event
1-454	1	154886776	155128719	Misassembly	NONE	154886776	155128719		0					OTHER		complex event
1-455	1	155348285	155501976	Misassembly	NONE	155348285	155501976		0					OTHER		complex event with deletion
1-457	1	156148609	156209871	Misassembly	NONE	156148609	156209871		0					OTHER		complex event
1-460	1	156835411	156897179	Misassembly	NONE	156835411	156897179		0					OTHER		complex event
1-461	1	157339707	157390126	Misassembly	NONE	157339707	157390126		0					OTHER		complex event
1-463	1	157728114	157806099	Misassembly	NONE	157728114	157806099		0					OTHER		complex event
1-465	1	157966262	158041475	Misassembly	NONE	157966262	158041475		0					OTHER		complex event
1-467	1	158461827	158666265	Misassembly	NONE	158461827	158666265		0					OTHER		complex event
1-468	1	158759602	158794705	Misassembly	NONE	158759602	158794705		0					OTHER		complex event possible inversion
1-471	1	159726180	159778567	Misassembly	NONE	159726180	159778567		0					OTHER		complex event
1-475	1	160166630	160296689	Misassembly	NONE	160166630	160296689		0					OTHER		complex event
1-476	1	160325148	160535909	Misassembly	NONE	160325148	160535909		0					OTHER		complex event
1-477	1	160849240	160904810	Misassembly	NONE	160849240	160904810		0					OTHER		complex event
1-478	1	160961489	161048450	Misassembly	NONE	160961489	161048450		0					OTHER		complex event
1-481	1	161330274	161491607	Misassembly	NONE	161330274	161491607		0					OTHER		complex event with insertion
1-485	1	162967012	163016420	Misassembly	NONE	162967012	163016420		0					OTHER		complex event with deletion
1-488	1	163371112	163520854	Misassembly	NONE	163371112	163520854		0					OTHER		complex event
1-491	1	163960432	164134625	Misassembly	NONE	163960432	164134625		0					OTHER		complex event
1-493	1	164301217	164588997	Misassembly	NONE	164301217	164588997		0					OTHER		complex event
1-495	1	164957546	164994483	Misassembly	NONE	164957546	164994483		0					OTHER		complex event
1-496	1	165288153	165314573	Misassembly	NONE	165288153	165314573		0					OTHER		complex event
1-497	1	165575867	165760750	Misassembly	NONE	165575867	165760750		0					OTHER		complex event
1-501	1	166155002	166349870	Misassembly	NONE	166155002	166349870		0					OTHER		complex event
1-502	1	166451747	166566498	Misassembly	NONE	166451747	166566498		0					OTHER		complex event
1-504	1	166796102	166864156	Misassembly	NONE	166796102	166864156		0					OTHER		complex event
1-506	1	167206272	167470017	Misassembly	NONE	167206272	167470017		0					OTHER		complex event
1-507	1	167595235	167812398	Misassembly	NONE	167595235	167812398		0					OTHER		complex event

1-508	1	168020022	168113791	Misassembly	NONE	168020022	168113791		0					OTHER		complex event
1-510	1	168713351	168857990	Misassembly	NONE	168713351	168857990		0					OTHER		complex event with insertion
1-512	1	169338269	169535340	Misassembly	NONE	169338269	169535340		0					OTHER		complex event
1-514	1	169995439	170072895	Misassembly	NONE	169995439	170072895		0					OTHER		complex event
1-515	1	170478839	170945824	Misassembly	NONE	170478839	170945824		0					OTHER		complex event
1-518	1	171645811	171769633	Misassembly	NONE	171645811	171769633		0					OTHER		complex event
1-519	1	171824196	171862122	Misassembly	NONE	171824196	171862122		0					OTHER		complex event
1-520	1	172092860	172192805	Misassembly	NONE	172092860	172192805		0					OTHER		complex event
1-523	1	172561350	172730984	Misassembly	NONE	172561350	172730984		0					OTHER		complex event
1-525	1	173137784	173290833	Misassembly	NONE	173137784	173290833		0					OTHER		complex event
1-526	1	173349613	173587810	Misassembly	NONE	173349613	173587810		0					OTHER		complex event
1-527	1	173726605	173844280	Misassembly	NONE	173726605	173844280		0					OTHER		complex event
1-529	1	174183106	174225678	Misassembly	NONE	174183106	174225678		0					OTHER		complex event
1-530	1	174460723	174497770	Misassembly	NONE	174460723	174497770		0					OTHER		complex event
1-534	1	174953681	175061226	Misassembly	NONE	174953681	175061226		0					OTHER		complex event
1-536	1	176711080	176974755	Misassembly	NONE	176711080	176974755		0					OTHER		complex event
1-537	1	177008991	177095802	Misassembly	NONE	177008991	177095802		0					OTHER		complex event
1-539	1	178594928	178768342	Misassembly	NONE	178594928	178768342		0					OTHER		complex event
1-541	1	180187017	180302704	Misassembly	NONE	180187017	180302704		0					OTHER		complex event
1-542	1	180338959	180606195	Misassembly	NONE	180338959	180606195		0					OTHER		complex event
1-545	1	183046269	183119158	Misassembly	NONE	183046269	183119158		0					OTHER		complex event
1-546	1	183167280	183274083	Misassembly	NONE	183167280	183274083		0					OTHER		complex event
1-552	1	184417311	184508850	Misassembly	NONE	184417311	184508850		0					OTHER		complex event
1-553	1	184562720	184710610	Misassembly	NONE	184562720	184710610		0					OTHER		complex event
1-554	1	184934206	185036646	Misassembly	NONE	184934206	185036646		0					OTHER		complex event
1-555	1	185107191	185343365	Misassembly	NONE	185107191	185343365		0					OTHER		complex event
1-557	1	186107347	186234171	Misassembly	NONE	186107347	186234171		0					OTHER		complex event
1-558	1	186521057	186607601	Misassembly	NONE	186521057	186607601		0					OTHER		complex event
1-559	1	186737312	186854959	Misassembly	NONE	186737312	186854959		0					OTHER		complex event
1-561	1	189915113	189980104	Misassembly	NONE	189915113	189980104		0					OTHER		complex event
1-562	1	191434344	191674845	Misassembly	NONE	191434344	191674845		0					OTHER		complex event
1-563	1	191779303	191995116	Misassembly	NONE	191779303	191995116		0					OTHER		complex event
1-567	1	192661574	193093381	Misassembly	NONE	192661574	193093381		0					OTHER		complex event
1-568	1	193150341	193168373	Misassembly	NONE	193150341	193168373		0					OTHER		complex event
1-570	1	193991000	194165703	Misassembly	NONE	193991000	194165703		0					OTHER		complex event
1-572	1	194306798	194445268	Misassembly	NONE	194306798	194445268		0					OTHER		complex event
1-573	1	194513237	194604784	Misassembly	NONE	194513237	194604784		0					OTHER		complex event
1-574	1	194761059	194900019	Misassembly	NONE	194761059	194900019		0					OTHER		complex event
1-576	1	195001594	195189685	Misassembly	NONE	195001594	195189685		0					OTHER		complex event
1-578	1	195335115	195402600	Misassembly	NONE	195335115	195402600		0					OTHER		complex event
1-579	1	195685569	195743319	Misassembly	NONE	195685569	195743319		0					OTHER		complex event
1-581	1	196115766	196256092	Misassembly	NONE	196115766	196256092		0					OTHER		complex event
1-582	1	196336267	196397108	Misassembly	NONE	196336267	196397108		0					OTHER		complex event
1-584	1	196838299	196955486	Misassembly	NONE	196838299	196955486		0					OTHER		complex event
1-585	1	197014362	197119812	Misassembly	NONE	197014362	197119812		0					OTHER		complex event
1-589	1	197470717	197553377	Misassembly	NONE	197470717	197553377		0					OTHER		complex event
1-591	1	197822645	197965703	Misassembly	NONE	197822645	197965703		0					OTHER		complex event
1-594	1	198861310	198988618	Misassembly	NONE	198861310	198988618		0					OTHER		complex event
1-595	1	199014608	199073517	Misassembly	NONE	199014608	199073517		0					OTHER		complex event
1-596	1	199550971	199605316	Misassembly	NONE	199550971	199605316		0					OTHER		complex event
1-600	1	205141769	205218808	Misassembly	NONE	205141769	205218808		0					OTHER		complex event with deletion
1-602	1	205413663	205437859	Misassembly	NONE	205413663	205437859		0					OTHER		complex event
1-607	1	206303377	206418762	Misassembly	NONE	206303377	206418762		0					OTHER		complex event
1-610	1	207036087	207220886	Misassembly	NONE	207036087	207220886		0					OTHER		complex event
1-614	1	207951151	208073489	Misassembly	NONE	207951151	208073489		0					OTHER		complex event
1-615	1	208130551	208433276	Misassembly	NONE	208130551	208433276		0					OTHER		complex event
1-616	1	208567461	208801652	Misassembly	NONE	208567461	208801652		0					OTHER		complex event
1-617	1	208959893	209111495	Misassembly	NONE	208959893	209111495		0					OTHER		complex event
1-619	1	209652680	209755770	Misassembly	NONE	209652680	209755770		0					OTHER		complex event
1-620	1	210155461	210255961	Misassembly	NONE	210155461	210255961		0					OTHER		complex event
1-621	1	210403092	210812092	Misassembly	NONE	210403092	210812092		0					OTHER		complex event
1-622	1	211074885	211135800	Misassembly	NONE	211074885	211135800		0					OTHER		complex event
1-623	1	211188308	211248240	Misassembly	NONE	211188308	211248240		0					OTHER		complex event
1-627	1	211488626	211538515	Misassembly	NONE	211488626	211538515		0					OTHER		complex event
1-628	1	211646388	211685969	Misassembly	NONE	211646388	211685969		0					OTHER		complex event
1-629	1	211734079	211786623	Misassembly	NONE	211734079	211786623		0					OTHER		complex event
1-630	1	211951295	212152843	Misassembly	NONE	211951295	212152843		0					OTHER		complex event
1-631	1	212211440	212355818	Misassembly	NONE	212211440	212355818		0					OTHER		complex event
1-632	1	212432160	212620435	Misassembly	NONE	212432160	212620435		0					OTHER		complex event
1-633	1	212696778	212935888	Misassembly	NONE	212696778	212935888		0					OTHER		complex event
1-636	1	213134627	213467241	Misassembly	NONE	213134627	213467241		0					OTHER		complex event
1-637	1	213841987	213989373	Misassembly	NONE	213841987	213989373		0					OTHER		complex event
1-638	1	214359265	214772719	Misassembly	NONE	214359265	214772719		0					OTHER		complex event
1-639	1	214875035	214953276	Misassembly	NONE	214875035	214953276		0					OTHER		complex event
1-640	1	215363927	215483431	Misassembly	NONE	215363927	215483431		0					OTHER		complex event
1-641	1	215689170	215882470	Misassembly	NONE	215689170	215882470		0					OTHER		complex event
1-642	1	216259399	216331867	Misassembly	NONE	216259399	216331867		0					OTHER		complex event
1-643	1	216721653	216913342	Misassembly	NONE	216721653	216913342		0					OTHER		complex event
1-646	1	217376898	217637126	Misassembly	NONE	217376898	217637126		0					OTHER		complex event
1-647	1	217853128	218135161	Misassembly	NONE	217853128	218135161		0					OTHER		complex event
1-648	1	218217319	218423476	Misassembly	NONE	218217319	218423476		0					OTHER		complex event
1-649	1	218754281	218873670	Misassembly	NONE	218754281	218873670		0					OTHER		complex event
1-650	1	219200794	219259114	Misassembly	NONE	219200794	219259114		0					OTHER		complex event
1-653	1	220076536	220181337	Misassembly	NONE	220076536	220181337		0					OTHER		complex event
1-659	1	221974150	222078363	Misassembly	NONE	221974150	222078363		0					OTHER		complex event
1-663	1	224261761	224502476	Misassembly	NONE	224261761	224502476		0					OTHER		complex event
1-664	1	224536072	224605858	Misassembly	NONE	224536072	224605858		0					OTHER		complex event
1-666	1	224827677	224877988	Misassembly	NONE	224827677	224877988		0					OTHER		complex event
1-667	1	225065876	225616886	Misassembly	NONE	225065876	225616886		0					OTHER		complex event

1-668	1	225727721	225820970	Misassembly	NONE	225727721	225820970		0					OTHER		complex event
1-669	1	226043424	226176777	Misassembly	NONE	226043424	226176777		0					OTHER		complex event with insertion
1-671	1	226350563	226499943	Misassembly	NONE	226350563	226499943		0					OTHER		complex event
1-672	1	226617778	226782542	Misassembly	NONE	226617778	226782542		0					OTHER		complex event
1-677	1	227772594	228030085	Misassembly	NONE	227772594	228030085		0					OTHER		complex event
1-681	1	229580273	229724973	Misassembly	NONE	229580273	229724973		0					OTHER		complex event
1-683	1	230155297	230301086	Misassembly	NONE	230155297	230301086		0					OTHER		complex event
1-686	1	230671020	230930116	Misassembly	NONE	230671020	230930116		0					OTHER		complex event
1-691	1	231644354	231766007	Misassembly	NONE	231644354	231766007		0					OTHER		complex event
1-693	1	232012872	232188573	Misassembly	NONE	232012872	232188573		0					OTHER		complex event
1-694	1	232241573	232293615	Misassembly	NONE	232241573	232293615		0					OTHER		complex event
1-695	1	232427439	232535568	Misassembly	NONE	232427439	232535568		0					OTHER		complex event
1-696	1	232901141	233088871	Misassembly	NONE	232901141	233088871		0					OTHER		complex event
1-697	1	233182856	233292227	Misassembly	NONE	233182856	233292227		0					OTHER		complex event
1-698	1	233762652	234183333	Misassembly	NONE	233762652	234183333		0					OTHER		complex event with deletion
1-699	1	234252233	234549201	Misassembly	NONE	234252233	234549201		0					OTHER		
1-701	1	234926818	235054514	Misassembly	NONE	234926818	235054514		0					OTHER		complex event
1-703	1	235779480	235974956	Misassembly	NONE	235779480	235974956		0					OTHER		complex event
1-704	1	236105320	236149134	Misassembly	NONE	236105320	236149134		0					OTHER		complex event
1-705	1	237558097	237729885	Misassembly	NONE	237558097	237729885		0					OTHER		complex event
1-708	1	238325669	238388200	Misassembly	NONE	238325669	238388200		0					OTHER		complex event
1-710	1	241851700	241969755	Misassembly	NONE	241851700	241969755		0					OTHER		complex event
1-711	1	242105875	242184516	Misassembly	NONE	242105875	242184516		0					OTHER		complex event
1-712	1	242362469	242429176	Misassembly	NONE	242362469	242429176		0					OTHER		complex event
1-714	1	242910572	243009436	Misassembly	NONE	242910572	243009436		0					OTHER		complex event
1-715	1	245380305	245469706	Misassembly	NONE	245380305	245469706		0					OTHER		complex event
1-717	1	245883582	245944625	Misassembly	NONE	245883582	245944625		0					OTHER		complex event
1-721	1	246653376	246691753	Misassembly	NONE	246653376	246691753		0					OTHER		complex event
1-722	1	246740089	246797299	Misassembly	NONE	246740089	246797299		0					OTHER		complex event with deletion
1-726	1	247673385	247866561	Misassembly	NONE	247673385	247866561		0					OTHER		complex event
1-727	1	247982675	248201177	Misassembly	NONE	247982675	248201177		0					OTHER		complex event
1-729	1	251332266	251548014	Misassembly	NONE	251332266	251548014		0					OTHER		complex event
1-730	1	251754460	251870739	Misassembly	NONE	251754460	251870739		0					OTHER		complex event
1-731	1	252442831	252519859	Misassembly	NONE	252442831	252519859		0					OTHER		complex event
1-733	1	252721880	252850057	Misassembly	NONE	252721880	252850057		0					OTHER		complex event
1-734	1	253089104	253116431	Misassembly	NONE	253089104	253116431		0					OTHER		complex event
1-735	1	253319877	253414617	Misassembly	NONE	253319877	253414617		0					OTHER		complex event
1-736	1	253472655	253604672	Misassembly	NONE	253472655	253604672		0					OTHER		complex event
1-737	1	253775112	254213598	Misassembly	NONE	253775112	254213598		0					OTHER		complex event
1-738	1	254507195	254547550	Misassembly	NONE	254507195	254547550		0					OTHER		complex event
1-739	1	254595538	254685257	Misassembly	NONE	254595538	254685257		0					OTHER		complex event
1-740	1	254789390	254905308	Misassembly	NONE	254789390	254905308		0					OTHER		complex event
1-742	1	255225449	255406869	Misassembly	NONE	255225449	255406869		0					OTHER		complex event
1-745	1	260334429	260396502	Misassembly	NONE	260334429	260396502		0					OTHER		complex event
1-749	1	261138909	261242164	Misassembly	NONE	261138909	261242164		0					OTHER		complex event with deletion
1-750	1	261508896	261639868	Misassembly	NONE	261508896	261639868		0					OTHER		complex event
1-752	1	264850189	264916687	Misassembly	NONE	264850189	264916687		0					OTHER		complex event
1-753	1	265175350	265274635	Misassembly	NONE	265175350	265274635		0					OTHER		complex event
1-759	1	267116077	267330752	Misassembly	NONE	267116077	267330752		0					OTHER		complex event
1-765	1	270075800	270141149	Misassembly	NONE	270075800	270141149		0					OTHER		complex event
1-766	1	270334742	270531264	Misassembly	NONE	270334742	270531264		0					OTHER		complex event
1-768	1	271863227	271899084	Misassembly	NONE	271863227	271899084		0					OTHER		complex event
1-769	1	272119769	272193595	Misassembly	NONE	272119769	272193595		0					OTHER		complex event
1-771	1	272403779	272499208	Misassembly	NONE	272403779	272499208		0					OTHER		complex event
1-772	1	272598202	272621433	Misassembly	NONE	272598202	272621433		0					OTHER		complex event
1-773	1	272779416	272825693	Misassembly	NONE	272779416	272825693		0					OTHER		complex event
1-774	1	272904779	272964344	Misassembly	NONE	272904779	272964344		0					OTHER		complex event
1-775	1	273119240	273244688	Misassembly	NONE	273119240	273244688		0					OTHER		complex event
1-776	1	273269280	273362872	Misassembly	NONE	273269280	273362872		0					OTHER		complex event
1-778	1	273572425	273695741	Misassembly	NONE	273572425	273695741		0					OTHER		complex event
1-779	1	273727799	273877122	Misassembly	NONE	273727799	273877122		0					OTHER		complex event
1-781	1	275182629	275315316	Misassembly	NONE	275182629	275315316		0					OTHER		complex event
1-784	1	278246313	278335314	Misassembly	NONE	278246313	278335314		0					OTHER		complex event with deletion
1-786	1	278589705	278836319	Misassembly	NONE	278589705	278836319		0					OTHER		complex event
1-789	1	283500795	283830639	Misassembly	NONE	283500795	283830639		0					OTHER		complex event
1-790	1	284196770	284499252	Misassembly	NONE	284196770	284499252		0					OTHER		complex event
1-791	1	284573712	284665312	Misassembly	NONE	284573712	284665312		0					OTHER		complex event
1-792	1	284719491	284815796	Misassembly	NONE	284719491	284815796		0					OTHER		complex event
1-794	1	284980674	285180475	Misassembly	NONE	284980674	285180475		0					OTHER		complex event
1-796	1	286793203	286851441	Misassembly	NONE	286793203	286851441		0					OTHER		complex event
1-798	1	288644932	288782224	Misassembly	NONE	288644932	288782224		0					OTHER		complex event
1-799	1	289006375	289120954	Misassembly	NONE	289006375	289120954		0					OTHER		complex event
1-801	1	289477372	289575457	Misassembly	NONE	289477372	289575457		0					OTHER		complex event
1-804	1	290236145	290437845	Misassembly	NONE	290236145	290437845		0					OTHER		complex event
1-805	1	290584566	290639348	Misassembly	NONE	290584566	290639348		0					OTHER		complex event
1-806	1	290747934	290805278	Misassembly	NONE	290747934	290805278		0					OTHER		complex event
1-809	1	291376335	291543222	Misassembly	NONE	291376335	291543222		0					OTHER		complex event
1-810	1	291693501	291862915	Misassembly	NONE	291693501	291862915		0					OTHER		complex event
1-811	1	296848980	296943712	Misassembly	NONE	296848980	296943712		0					OTHER		complex event
1-812	1	297109220	297135786	Misassembly	NONE	297109220	297135786		0					OTHER		complex event
1-813	1	297364078	297452662	Misassembly	NONE	297364078	297452662		0					OTHER		complex event
1-817	1	299546828	299637647	Misassembly	NONE	299546828	299637647		0					OTHER		complex event
2-1	2	859753	963083	Misassembly	NONE	859753	963083		0					OTHER		complex event
2-2	2	1486958	1546539	Misassembly	NONE	1486958	1546539		0					OTHER		
2-5	2	4905528	5045210	Misassembly	NONE	4905528	5045210		0					OTHER		complex event
2-9	2	5592134	5769170	Misassembly	NONE	5592134	5769170		0					OTHER		complex event

2-13	2	6069129	6175933	Misassembly	NONE	6069129	6175933		0					OTHER		complex event
2-16	2	11996378	12245157	Misassembly	NONE	11996378	12245157		0					OTHER		complex event
2-18	2	12319431	12394245	Misassembly	NONE	12319431	12394245		0					OTHER		complex event
2-21	2	13075343	13223191	Misassembly	NONE	13075343	13223191		0					OTHER		complex event
2-23	2	13985412	14183700	Misassembly	NONE	13985412	14183700		0					OTHER		complex event
2-24	2	14485968	14703210	Misassembly	NONE	14485968	14703210		0					OTHER		complex event
2-25	2	14713154	15176472	Misassembly	NONE	14713154	15176472		0					OTHER		complex event
2-26	2	15285171	15417570	Misassembly	NONE	15285171	15417570		0					OTHER		complex event
2-27	2	15602597	15782390	Misassembly	NONE	15602597	15782390		0					OTHER		complex event
2-31	2	16796980	16882066	Misassembly	NONE	16796980	16882066		0					OTHER		complex event
2-32	2	17474464	17532482	Misassembly	NONE	17474464	17532482		0					OTHER		complex event
2-33	2	17662822	17692069	Misassembly	NONE	17662822	17692069		0					OTHER		
2-34	2	17834593	17943853	Misassembly	NONE	17834593	17943853		0					OTHER		complex event
2-35	2	17992557	18217667	Misassembly	NONE	17992557	18217667		0					OTHER		complex event
2-36	2	18747698	18857217	Misassembly	NONE	18747698	18857217		0					OTHER		complex event
2-39	2	19317127	19387950	Misassembly	NONE	19317127	19387950		0					OTHER		complex event
2-41	2	19912492	19992864	Misassembly	NONE	19912492	19992864		0					OTHER		complex event
2-42	2	20138692	20198397	Misassembly	NONE	20138692	20198397		0					OTHER		complex event
2-44	2	20531527	20836743	Misassembly	NONE	20531527	20836743		0					OTHER		complex event
2-47	2	21403361	21639734	Misassembly	NONE	21403361	21639734		0					OTHER		complex event
2-49	2	22200666	22368197	Misassembly	NONE	22200666	22368197		0					OTHER		complex event
2-50	2	22389712	22843343	Misassembly	NONE	22389712	22843343		0					OTHER		complex event
2-51	2	22878641	22919977	Misassembly	NONE	22878641	22919977		0					OTHER		complex event
2-52	2	23229187	23305678	Misassembly	NONE	23229187	23305678		0					OTHER		complex event
2-57	2	24044263	24156689	Misassembly	NONE	24044263	24156689		0					OTHER		complex event with insertion
2-59	2	24376159	24615012	Misassembly	NONE	24376159	24615012		0					OTHER		complex event
2-60	2	24757995	25231099	Misassembly	NONE	24757995	25231099		0					OTHER		complex event
2-61	2	25278856	25414783	Misassembly	NONE	25278856	25414783		0					OTHER		complex event
2-62	2	25455953	25629097	Misassembly	NONE	25455953	25629097		0					OTHER		complex event
2-63	2	25899233	26015662	Misassembly	NONE	25899233	26015662		0					OTHER		complex event
2-64	2	26136616	26299977	Misassembly	NONE	26136616	26299977		0					OTHER		complex event
2-65	2	26365534	26516862	Misassembly	NONE	26365534	26516862		0					OTHER		complex event
2-67	2	26654982	26751093	Misassembly	NONE	26654982	26751093		0					OTHER		complex event
2-69	2	27334837	27400634	Misassembly	NONE	27334837	27400634		0					OTHER		complex event
2-72	2	28014425	28086901	Misassembly	NONE	28014425	28086901		0					OTHER		complex event
2-73	2	28205372	28315732	Misassembly	NONE	28205372	28315732		0					OTHER		complex event
2-74	2	28335728	28437665	Misassembly	NONE	28335728	28437665		0					OTHER		complex event
2-75	2	28487714	28737176	Misassembly	NONE	28487714	28737176		0					OTHER		complex event
2-78	2	31511605	31632860	Misassembly	NONE	31511605	31632860		0					OTHER		complex event
2-79	2	31720972	31777202	Misassembly	NONE	31720972	31777202		0					OTHER		complex event
2-81	2	32052487	32126788	Misassembly	NONE	32052487	32126788		0					OTHER		complex event
2-82	2	32184744	32339756	Misassembly	NONE	32184744	32339756		0					OTHER		complex event
2-83	2	32497125	32570938	Misassembly	NONE	32497125	32570938		0					OTHER		complex event
2-85	2	32762974	32875515	Misassembly	NONE	32762974	32875515		0					OTHER		complex event
2-86	2	32896463	32919861	Misassembly	NONE	32896463	32919861		0					OTHER		complex event
2-87	2	33001648	33036625	Misassembly	NONE	33001648	33036625		0					OTHER		complex event
2-89	2	33366745	33420281	Misassembly	NONE	33366745	33420281		0					OTHER		complex event
2-90	2	33689815	33814426	Misassembly	NONE	33689815	33814426		0					OTHER		complex event
2-91	2	33920814	33959460	Misassembly	NONE	33920814	33959460		0					OTHER		complex event
2-94	2	36854729	36940351	Misassembly	NONE	36854729	36940351		0					OTHER		complex event
2-96	2	38707404	38827003	Misassembly	NONE	38707404	38827003		0					OTHER		complex event
2-97	2	38874153	38936995	Misassembly	NONE	38874153	38936995		0					OTHER		complex event
2-99	2	39107809	39210923	Misassembly	NONE	39107809	39210923		0					OTHER		complex event
2-103	2	40066631	40168241	Misassembly	NONE	40066631	40168241		0					OTHER		complex event
2-105	2	40392373	40433915	Misassembly	NONE	40392373	40433915		0					OTHER		complex event
2-106	2	42692842	42738237	Misassembly	NONE	42692842	42738237		0					OTHER		complex event
2-107	2	42979583	43083337	Misassembly	NONE	42979583	43083337		0					OTHER		complex event
2-108	2	43266629	43409820	Misassembly	NONE	43266629	43409820		0					OTHER		complex event
2-111	2	45147558	45214391	Misassembly	NONE	45147558	45214391		0					OTHER		complex event
2-112	2	45274828	45335267	Misassembly	NONE	45274828	45335267		0					OTHER		complex event
2-113	2	45444710	45619022	Misassembly	NONE	45444710	45619022		0					OTHER		complex event
2-121	2	46925999	47015172	Misassembly	NONE	46925999	47015172		0					OTHER		complex event
2-122	2	47262996	47318186	Misassembly	NONE	47262996	47318186		0					OTHER		complex event
2-123	2	47414818	47521784	Misassembly	NONE	47414818	47521784		0					OTHER		complex event
2-125	2	48018369	48130671	Misassembly	NONE	48018369	48130671		0					OTHER		complex event
2-126	2	48193209	48247213	Misassembly	NONE	48193209	48247213		0					OTHER		complex event
2-129	2	48797952	48951488	Misassembly	NONE	48797952	48951488		0					OTHER		complex event
2-130	2	49058058	49130048	Misassembly	NONE	49058058	49130048		0					OTHER		complex event
2-132	2	49270784	49351322	Misassembly	NONE	49270784	49351322		0					OTHER		complex event
2-133	2	49669474	49704109	Misassembly	NONE	49669474	49704109		0					OTHER		complex event
2-134	2	51353055	51464185	Misassembly	NONE	51353055	51464185		0					OTHER		complex event
2-135	2	52051705	52118187	Misassembly	NONE	52051705	52118187		0					OTHER		complex event
2-136	2	52204570	52364824	Misassembly	NONE	52204570	52364824		0					OTHER		complex event
2-137	2	52486051	52808659	Misassembly	NONE	52486051	52808659		0					OTHER		complex event
2-142	2	54305943	54353933	Misassembly	NONE	54305943	54353933		0					OTHER		complex event
2-145	2	58100235	58264910	Misassembly	NONE	58100235	58264910		0					OTHER		complex event
2-146	2	58703588	58802522	Misassembly	NONE	58703588	58802522		0					OTHER		complex event
2-148	2	60137265	60253407	Misassembly	NONE	60137265	60253407		0					OTHER		complex event
2-149	2	60734056	61038339	Misassembly	NONE	60734056	61038339		0					OTHER		complex event
2-150	2	61198154	61494703	Misassembly	NONE	61198154	61494703		0					OTHER		complex event
2-152	2	62126259	62166376	Misassembly	NONE	62126259	62166376		0					OTHER		complex event
2-153	2	62258110	62357206	Misassembly	NONE	62258110	62357206		0					OTHER		complex event
2-157	2	65807980	65907336	Misassembly	NONE	65807980	65907336		0					OTHER		complex event
2-159	2	66235049	66512838	Misassembly	NONE	66235049	66512838		0					OTHER		complex event
2-162	2	67031907	67128181	Misassembly	NONE	67031907	67128181		0					OTHER		complex event
2-164	2	67274008	67374553	Misassembly	NONE	67274008	67374553		0					OTHER		complex event
2-169	2	67891251	68029025	Misassembly	NONE	67891251	68029025		0					OTHER		complex event
2-170	2	68146205	68251446	Misassembly	NONE	68146205	68251446		0					OTHER		complex event
2-171	2	68392820	68470132	Misassembly	NONE	68392820	68470132		0					OTHER		complex event
2-173	2	68811726	68898680	Misassembly	NONE	68811726	68898680		0					OTHER		complex event

2-174	2	68922520	69022677	Misassembly	NONE	68922520	69022677		0					OTHER		complex event
2-175	2	69231754	69272410	Misassembly	NONE	69231754	69272410		0					OTHER		complex event
2-176	2	69335178	69443258	Misassembly	NONE	69335178	69443258		0					OTHER		complex event
2-177	2	69484100	69547744	Misassembly	NONE	69484100	69547744		0					OTHER		complex event
2-179	2	70151042	70398925	Misassembly	NONE	70151042	70398925		0					OTHER		complex event
2-181	2	70748475	71045915	Misassembly	NONE	70748475	71045915		0					OTHER		complex event
2-182	2	71099062	71252777	Misassembly	NONE	71099062	71252777		0					OTHER		
2-183	2	71385459	71691800	Misassembly	NONE	71385459	71691800		0					OTHER		complex event
2-185	2	72179709	72251771	Misassembly	NONE	72179709	72251771		0					OTHER		complex event
2-190	2	73264823	73352093	Misassembly	NONE	73264823	73352093		0					OTHER		complex event
2-191	2	73521247	73620835	Misassembly	NONE	73521247	73620835		0					OTHER		complex event
2-192	2	73765170	73969922	Misassembly	NONE	73765170	73969922		0					OTHER		complex event
2-193	2	74082393	74284546	Misassembly	NONE	74082393	74284546		0					OTHER		complex event
2-194	2	74470819	74520374	Misassembly	NONE	74470819	74520374		0					OTHER		complex event
2-196	2	75175686	75396379	Misassembly	NONE	75175686	75396379		0					OTHER		complex event
2-197	2	75490498	75525157	Misassembly	NONE	75490498	75525157		0					OTHER		complex event
2-198	2	75673517	75825372	Misassembly	NONE	75673517	75825372		0					OTHER		complex event with deletion
2-201	2	76224383	76306619	Misassembly	NONE	76224383	76306619		0					OTHER		complex event
2-203	2	76677009	76762136	Misassembly	NONE	76677009	76762136		0					OTHER		complex event
2-204	2	77063447	77126738	Misassembly	NONE	77063447	77126738		0					OTHER		complex event
2-206	2	79779039	80254000	Misassembly	NONE	79779039	80254000		0					OTHER		complex event
2-209	2	80986613	81269902	Misassembly	NONE	80986613	81269902		0					OTHER		complex event
2-210	2	81335749	81526386	Misassembly	NONE	81335749	81526386		0					OTHER		complex event
2-214	2	82166797	82272296	Misassembly	NONE	82166797	82272296		0					OTHER		complex event
2-218	2	83778340	83930910	Misassembly	NONE	83778340	83930910		0					OTHER		complex event
2-219	2	84135397	84318822	Misassembly	NONE	84135397	84318822		0					OTHER		complex event
2-220	2	84482521	84582143	Misassembly	NONE	84482521	84582143		0					OTHER		complex event
2-222	2	84908407	85041784	Misassembly	NONE	84908407	85041784		0					OTHER		complex event
2-224	2	85727558	86035333	Misassembly	NONE	85727558	86035333		0					OTHER		complex event
2-227	2	86641301	86774658	Misassembly	NONE	86641301	86774658		0					OTHER		complex event
2-230	2	87307453	87490237	Misassembly	NONE	87307453	87490237		0					OTHER		complex event
2-231	2	87895592	88081837	Misassembly	NONE	87895592	88081837		0					OTHER		complex event
2-233	2	88841514	88925641	Misassembly	NONE	88841514	88925641		0					OTHER		complex event
2-234	2	91227851	91269780	Misassembly	NONE	91227851	91269780		0					OTHER		complex event
2-235	2	91661596	92067913	Misassembly	NONE	91661596	92067913		0					OTHER		complex event
2-237	2	92352622	92590287	Misassembly	NONE	92352622	92590287		0					OTHER		complex event
2-239	2	92950520	93084434	Misassembly	NONE	92950520	93084434		0					OTHER		complex event
2-240	2	93283415	93337185	Misassembly	NONE	93283415	93337185		0					OTHER		complex event
2-247	2	94421906	94798350	Misassembly	NONE	94421906	94798350		0					OTHER		complex event
2-248	2	95036972	95234356	Misassembly	NONE	95036972	95234356		0					OTHER		complex event
2-249	2	95949846	95991314	Misassembly	NONE	95949846	95991314		0					OTHER		complex event
2-257	2	97765093	97924112	Misassembly	NONE	97765093	97924112		0					OTHER		complex event
2-258	2	97964821	97979959	Misassembly	NONE	97964821	97979959		0					OTHER		complex event
2-262	2	99026037	99178709	Misassembly	NONE	99026037	99178709		0					OTHER		complex event
2-263	2	99336380	99537733	Misassembly	NONE	99336380	99537733		0					OTHER		complex event
2-266	2	99901294	100004218	Misassembly	NONE	99901294	100004218		0					OTHER		complex event
2-268	2	100611804	100797783	Misassembly	NONE	100611804	100797783		0					OTHER		complex event
2-269	2	100922221	101106119	Misassembly	NONE	100922221	101106119		0					OTHER		complex event
2-270	2	101133473	101256625	Misassembly	NONE	101133473	101256625		0					OTHER		complex event
2-271	2	101256625	101640819	Misassembly	NONE	101256625	101640819		0					OTHER		complex event
2-272	2	102159211	102272738	Misassembly	NONE	102159211	102272738		0					OTHER		complex event
2-274	2	102660258	102944328	Misassembly	NONE	102660258	102944328		0					OTHER		complex event
2-275	2	103177484	103325749	Misassembly	NONE	103177484	103325749		0					OTHER		complex event
2-276	2	103641567	103717667	Misassembly	NONE	103641567	103717667		0					OTHER		complex event
2-277	2	103799081	103851893	Misassembly	NONE	103799081	103851893		0					OTHER		complex event
2-282	2	189363566	189497013	Misassembly	NONE	189363566	189497013		0					OTHER		complex event
2-284	2	194882406	194945119	Misassembly	NONE	194882406	194945119		0					OTHER		complex event
2-286	2	196939722	197373147	Misassembly	NONE	196939722	197373147		0					OTHER		complex event
2-288	2	197608130	197709195	Misassembly	NONE	197608130	197709195		0					OTHER		complex event
2-290	2	198984152	199177176	Misassembly	NONE	198984152	199177176		0					OTHER		complex event
2-293	2	199632458	199668839	Misassembly	NONE	199632458	199668839		0					OTHER		complex event
2-295	2	203197237	203280346	Misassembly	NONE	203197237	203280346		0					OTHER		complex event
2-296	2	203383129	203637844	Misassembly	NONE	203383129	203637844		0					OTHER		complex event
2-298	2	203738778	203763405	Misassembly	NONE	203738778	203763405		0					OTHER		complex event
2-299	2	204237239	204316337	Misassembly	NONE	204237239	204316337		0					OTHER		complex event
2-300	2	204539260	204681774	Misassembly	NONE	204539260	204681774		0					OTHER		complex event
2-304	2	208527394	208602594	Misassembly	NONE	208527394	208602594		0					OTHER		complex event
2-305	2	208655802	208674250	Misassembly	NONE	208655802	208674250		0					OTHER		complex event
2-308	2	209097763	209370926	Misassembly	NONE	209097763	209370926		0					OTHER		complex event
2-312	2	211311287	211411316	Misassembly	NONE	211311287	211411316		0					OTHER		complex event
2-314	2	211628613	211811746	Misassembly	NONE	211628613	211811746		0					OTHER		complex event
2-317	2	212057375	212278684	Misassembly	NONE	212057375	212278684		0					OTHER		complex event
2-319	2	216246044	216342531	Misassembly	NONE	216246044	216342531		0					OTHER		complex event
2-324	2	217110260	217266066	Misassembly	NONE	217110260	217266066		0					OTHER		complex event
2-325	2	218744567	218954475	Misassembly	NONE	218744567	218954475		0					OTHER		complex event
2-330	2	219762207	219972893	Misassembly	NONE	219762207	219972893		0					OTHER		complex event
2-331	2	220052739	220163952	Misassembly	NONE	220052739	220163952		0					OTHER		complex event
2-332	2	220259708	220481297	Misassembly	NONE	220259708	220481297		0					OTHER		complex event
2-340	2	225073128	225145798	Misassembly	NONE	225073128	225145798		0					OTHER		complex event
2-341	2	226475905	226525949	Misassembly	NONE	226475905	226525949		0					OTHER		complex event
2-345	2	228002699	228212397	Misassembly	NONE	228002699	228212397		0					OTHER		complex event with deletion
2-347	2	228829590	228963092	Misassembly	NONE	228829590	228963092		0					OTHER		complex event
2-350	2	229816274	229855859	Misassembly	NONE	229816274	229855859		0					OTHER		complex event
2-352	2	230348715	230403460	Misassembly	NONE	230348715	230403460		0					OTHER		complex event
2-356	2	231313101	231545743	Misassembly	NONE	231313101	231545743		0					OTHER		complex event
3-1	3	1593292	1696426	Misassembly	NONE	1593292	1696426		0					OTHER		complex event
3-3	3	2266310	2422606	Misassembly	NONE	2266310	2422606		0					OTHER		complex event
3-4	3	2604090	2786083	Misassembly	NONE	2604090	2786083		0					OTHER		complex event
3-5	3	2853219	3224723	Misassembly	NONE	2853219	3224723		0					OTHER		complex event

3-7	3	3682919	3768502	Misassembly	NONE	3682919	3768502		0					OTHER		complex event
3-9	3	7855691	8053897	Misassembly	NONE	7855691	8053897		0					OTHER		complex event
3-10	3	8349073	8472002	Misassembly	NONE	8349073	8472002		0					OTHER		complex event
3-14	3	16253133	16322026	Misassembly	NONE	16253133	16322026		0					OTHER		complex event
3-17	3	17790119	17941848	Misassembly	NONE	17790119	17941848		0					OTHER		complex event
3-19	3	18287511	18369665	Misassembly	NONE	18287511	18369665		0					OTHER		complex event
3-27	3	26605663	26629527	Misassembly	NONE	26605663	26629527		0					OTHER		complex event
3-29	3	27119752	27596188	Misassembly	NONE	27119752	27596188		0					OTHER		complex event
3-30	3	27735872	27774191	Misassembly	NONE	27735872	27774191		0					OTHER		complex event
3-31	3	27828933	27925092	Misassembly	NONE	27828933	27925092		0					OTHER		complex event
3-36	3	29259619	29287749	Misassembly	NONE	29259619	29287749		0					OTHER		complex event
3-37	3	29343416	29376462	Misassembly	NONE	29343416	29376462		0					OTHER		complex event
3-40	3	29896497	30315262	Misassembly	NONE	29896497	30315262		0					OTHER		complex event
3-44	3	31109943	31472455	Misassembly	NONE	31109943	31472455		0					OTHER		complex event
3-47	3	31887763	32174298	Misassembly	NONE	31887763	32174298		0					OTHER		complex event
3-48	3	32357631	32442281	Misassembly	NONE	32357631	32442281		0					OTHER		complex event
3-53	3	33672622	33809914	Misassembly	NONE	33672622	33809914		0					OTHER		complex event
3-58	3	35163784	35276645	Misassembly	NONE	35163784	35276645		0					OTHER		complex event
3-59	3	35327297	35841709	Misassembly	NONE	35327297	35841709		0					OTHER		complex event
3-60	3	35919432	35963612	Misassembly	NONE	35919432	35963612		0					OTHER		complex event
3-63	3	36401405	36424335	Misassembly	NONE	36401405	36424335		0					OTHER		complex event
3-67	3	37625143	37713508	Misassembly	NONE	37625143	37713508		0					OTHER		complex event
3-73	3	38368157	38455762	Misassembly	NONE	38368157	38455762		0					OTHER		complex event
3-74	3	38501238	38763425	Misassembly	NONE	38501238	38763425		0					OTHER		complex event with deletion
3-75	3	38873164	38983547	Misassembly	NONE	38873164	38983547		0					OTHER		complex event
3-76	3	39012849	39139473	Misassembly	NONE	39012849	39139473		0					OTHER		complex event
3-78	3	39832254	40066307	Misassembly	NONE	39832254	40066307		0					OTHER		complex event
3-79	3	40152923	40358847	Misassembly	NONE	40152923	40358847		0					OTHER		complex event
3-80	3	40437198	40581164	Misassembly	NONE	40437198	40581164		0					OTHER		complex event
3-81	3	40829369	40882876	Misassembly	NONE	40829369	40882876		0					OTHER		complex event
3-84	3	41080861	41416142	Misassembly	NONE	41080861	41416142		0					OTHER		complex event
3-86	3	41800914	41881728	Misassembly	NONE	41800914	41881728		0					OTHER		complex event
3-88	3	42170701	42391795	Misassembly	NONE	42170701	42391795		0					OTHER		complex event
3-91	3	42761491	42902811	Misassembly	NONE	42761491	42902811		0					OTHER		complex event
3-92	3	43066625	43181881	Misassembly	NONE	43066625	43181881		0					OTHER		complex event
3-93	3	43290446	43458537	Misassembly	NONE	43290446	43458537		0					OTHER		complex event
3-95	3	44004699	44061933	Misassembly	NONE	44004699	44061933		0					OTHER		complex event
3-96	3	44282043	44360752	Misassembly	NONE	44282043	44360752		0					OTHER		complex event
3-100	3	45375358	45460148	Misassembly	NONE	45375358	45460148		0					OTHER		complex event
3-102	3	45926283	46057623	Misassembly	NONE	45926283	46057623		0					OTHER		complex event
3-104	3	46323308	46375563	Misassembly	NONE	46323308	46375563		0					OTHER		complex event
3-107	3	46770254	46866970	Misassembly	NONE	46770254	46866970		0					OTHER		complex event with large insertion
3-108	3	47068725	47159695	Misassembly	NONE	47068725	47159695		0					OTHER		complex event
3-109	3	47213404	47323197	Misassembly	NONE	47213404	47323197		0					OTHER		complex event
3-112	3	47838519	47980813	Misassembly	NONE	47838519	47980813		0					OTHER		complex event
3-113	3	48179880	48419724	Misassembly	NONE	48179880	48419724		0					OTHER		complex event
3-115	3	48923281	49182246	Misassembly	NONE	48923281	49182246		0					OTHER		complex event
3-116	3	49318433	49457767	Misassembly	NONE	49318433	49457767		0					OTHER		complex event
3-119	3	50358801	50527882	Misassembly	NONE	50358801	50527882		0					OTHER		complex event
3-120	3	50580206	50634910	Misassembly	NONE	50580206	50634910		0					OTHER		complex event
3-124	3	51156101	51371879	Misassembly	NONE	51156101	51371879		0					OTHER		complex event
3-125	3	51418334	51486673	Misassembly	NONE	51418334	51486673		0					OTHER		complex event
3-126	3	51531867	51753993	Misassembly	NONE	51531867	51753993		0					OTHER		complex event
3-128	3	52009511	52107689	Misassembly	NONE	52009511	52107689		0					OTHER		complex event
3-129	3	52454030	52608513	Misassembly	NONE	52454030	52608513		0					OTHER		complex event
3-130	3	52855931	53002000	Misassembly	NONE	52855931	53002000		0					OTHER		complex event
3-133	3	53655560	53823846	Misassembly	NONE	53655560	53823846		0					OTHER		complex event
3-134	3	53958146	54015222	Misassembly	NONE	53958146	54015222		0					OTHER		complex event
3-137	3	54436659	54553527	Misassembly	NONE	54436659	54553527		0					OTHER		complex event
3-138	3	55344170	55465725	Misassembly	NONE	55344170	55465725		0					OTHER		complex event
3-140	3	55780016	55816909	Misassembly	NONE	55780016	55816909		0					OTHER		complex event
3-144	3	57300633	57468767	Misassembly	NONE	57300633	57468767		0					OTHER		complex event
3-146	3	57660889	57772933	Misassembly	NONE	57660889	57772933		0					OTHER		complex event
3-147	3	57803249	57869804	Misassembly	NONE	57803249	57869804		0					OTHER		complex event
3-149	3	60999559	61144481	Misassembly	NONE	60999559	61144481		0					OTHER		complex event
3-150	3	61543486	61622799	Misassembly	NONE	61543486	61622799		0					OTHER		complex event
3-151	3	62046449	62282226	Misassembly	NONE	62046449	62282226		0					OTHER		complex event
3-153	3	62830958	62861844	Misassembly	NONE	62830958	62861844		0					OTHER		complex event
3-154	3	63424550	63547937	Misassembly	NONE	63424550	63547937		0					OTHER		complex event
3-156	3	63873125	64044705	Misassembly	NONE	63873125	64044705		0					OTHER		complex event
3-160	3	64736118	64845239	Misassembly	NONE	64736118	64845239		0					OTHER		complex event
3-161	3	64861524	65153602	Misassembly	NONE	64861524	65153602		0					OTHER		complex event
3-162	3	65220389	65375669	Misassembly	NONE	65220389	65375669		0					OTHER		complex event
3-164	3	65767381	65846048	Misassembly	NONE	65767381	65846048		0					OTHER		complex event
3-167	3	66224552	66254558	Misassembly	NONE	66224552	66254558		0					OTHER		complex event
3-168	3	66313970	66392040	Misassembly	NONE	66313970	66392040		0					OTHER		complex event
3-169	3	66554563	66764510	Misassembly	NONE	66554563	66764510		0					OTHER		complex event
3-170	3	67113765	67253488	Misassembly	NONE	67113765	67253488		0					OTHER		complex event
3-171	3	67485095	67783990	Misassembly	NONE	67485095	67783990		0					OTHER		complex event
3-172	3	68355604	68420199	Misassembly	NONE	68355604	68420199		0					OTHER		complex event
3-173	3	68865299	68912400	Misassembly	NONE	68865299	68912400		0					OTHER		complex event
3-174	3	68941285	69024409	Misassembly	NONE	68941285	69024409		0					OTHER		complex event
3-175	3	70181570	70220097	Misassembly	NONE	70181570	70220097		0					OTHER		complex event
3-177	3	74232710	74645657	Misassembly	NONE	74232710	74645657		0					OTHER		complex event
3-178	3	74726472	74866266	Misassembly	NONE	74726472	74866266		0					OTHER		complex event
3-182	3	76717153	76755419	Misassembly	NONE	76717153	76755419		0					OTHER		complex event
3-187	3	77613598	77748762	Misassembly	NONE	77613598	77748762		0					OTHER		complex event
3-188	3	77941042	78056250	Misassembly	NONE	77941042	78056250		0					OTHER		complex event

3-194	3	79314114	79349545	Misassembly	NONE	79314114	79349545		0					OTHER		complex event
3-195	3	79526343	79609475	Misassembly	NONE	79526343	79609475		0					OTHER		complex event
3-197	3	79685711	80076506	Misassembly	NONE	79685711	80076506		0					OTHER		complex event
3-200	3	80625527	80710498	Misassembly	NONE	80625527	80710498		0					OTHER		complex event
3-201	3	80836884	81037154	Misassembly	NONE	80836884	81037154		0					OTHER		complex event
3-202	3	81609501	81649062	Misassembly	NONE	81609501	81649062		0					OTHER		complex event
3-207	3	83882751	83995199	Misassembly	NONE	83882751	83995199		0					OTHER		complex event
3-209	3	84746904	85136605	Misassembly	NONE	84746904	85136605		0					OTHER		complex event
3-210	3	85276812	85373826	Misassembly	NONE	85276812	85373826		0					OTHER		complex event
3-213	3	85692088	85774553	Misassembly	NONE	85692088	85774553		0					OTHER		complex event
3-214	3	86220565	86266471	Misassembly	NONE	86220565	86266471		0					OTHER		complex event
3-219	3	88318149	88407466	Misassembly	NONE	88318149	88407466		0					OTHER		complex event
3-220	3	89912799	89958385	Misassembly	NONE	89912799	89958385		0					OTHER		complex event
3-222	3	90374335	90535205	Misassembly	NONE	90374335	90535205		0					OTHER		complex event
3-224	3	91080362	91252077	Misassembly	NONE	91080362	91252077		0					OTHER		complex event
3-228	3	96505639	96569586	Misassembly	NONE	96505639	96569586		0					OTHER		complex event
3-230	3	97347490	97557580	Misassembly	NONE	97347490	97557580		0					OTHER		complex event
3-231	3	98171819	98286921	Misassembly	NONE	98171819	98286921		0					OTHER		complex event
3-232	3	98511950	98590592	Misassembly	NONE	98511950	98590592		0					OTHER		complex event
3-233	3	98811634	98917309	Misassembly	NONE	98811634	98917309		0					OTHER		complex event
3-235	3	99084988	99230248	Misassembly	NONE	99084988	99230248		0					OTHER		complex event
3-237	3	99389459	99505817	Misassembly	NONE	99389459	99505817		0					OTHER		complex event
3-240	3	99863817	99936268	Misassembly	NONE	99863817	99936268		0					OTHER		complex event
3-245	3	101978345	102092525	Misassembly	NONE	101978345	102092525		0					OTHER		complex event
3-247	3	102297115	102326588	Misassembly	NONE	102297115	102326588		0					OTHER		complex event
3-248	3	102580010	102749882	Misassembly	NONE	102580010	102749882		0					OTHER		complex event
3-249	3	103069013	103128072	Misassembly	NONE	103069013	103128072		0					OTHER		complex event
3-252	3	103578895	103797502	Misassembly	NONE	103578895	103797502		0					OTHER		complex event
3-255	3	104195085	104617256	Misassembly	NONE	104195085	104617256		0					OTHER		complex event
3-256	3	104618361	104886668	Misassembly	NONE	104618361	104886668		0					OTHER		complex event
3-257	3	105440406	105650759	Misassembly	NONE	105440406	105650759		0					OTHER		complex event
3-259	3	105836265	106087384	Misassembly	NONE	105836265	106087384		0					OTHER		complex event
3-261	3	106448641	106511660	Misassembly	NONE	106448641	106511660		0					OTHER		complex event
3-262	3	106647042	106891519	Misassembly	NONE	106647042	106891519		0					OTHER		complex event
3-263	3	107041220	107140100	Misassembly	NONE	107041220	107140100		0					OTHER		complex event
3-264	3	110767971	111001589	Misassembly	NONE	110767971	111001589		0					OTHER		complex event
3-265	3	111212780	111241033	Misassembly	NONE	111212780	111241033		0					OTHER		complex event
3-267	3	111595071	111701888	Misassembly	NONE	111595071	111701888		0					OTHER		complex event
3-268	3	111751650	111813806	Misassembly	NONE	111751650	111813806		0					OTHER		complex event
3-270	3	112158624	112258350	Misassembly	NONE	112158624	112258350		0					OTHER		complex event
3-277	3	114414156	114693636	Misassembly	NONE	114414156	114693636		0					OTHER		complex event
3-280	3	115197951	115301112	Misassembly	NONE	115197951	115301112		0					OTHER		complex event
3-283	3	116985216	117066645	Misassembly	NONE	116985216	117066645		0					OTHER		complex event
3-284	3	120932521	120974201	Misassembly	NONE	120932521	120974201		0					OTHER		complex event
3-286	3	121222467	121293078	Misassembly	NONE	121222467	121293078		0					OTHER		complex event
3-288	3	121412247	121589924	Misassembly	NONE	121412247	121589924		0					OTHER		complex event
3-289	3	121796122	121834150	Misassembly	NONE	121796122	121834150		0					OTHER		complex event
3-290	3	124005635	124078643	Misassembly	NONE	124005635	124078643		0					OTHER		complex event
3-293	3	125450089	125489873	Misassembly	NONE	125450089	125489873		0					OTHER		complex event
3-295	3	125756613	125887349	Misassembly	NONE	125756613	125887349		0					OTHER		complex event
3-309	3	135000279	135175175	Misassembly	NONE	135000279	135175175		0					OTHER		complex event
3-310	3	135503666	135609829	Misassembly	NONE	135503666	135609829		0					OTHER		complex event
3-313	3	136090283	136209572	Misassembly	NONE	136090283	136209572		0					OTHER		complex event
3-316	3	136778840	137011019	Misassembly	NONE	136778840	137011019		0					OTHER		complex event
3-317	3	137111297	137353098	Misassembly	NONE	137111297	137353098		0					OTHER		complex event
3-318	3	137449147	137504424	Misassembly	NONE	137449147	137504424		0					OTHER		complex event
3-319	3	137591323	137708855	Misassembly	NONE	137591323	137708855		0					OTHER		complex event
3-324	3	138631780	138858360	Misassembly	NONE	138631780	138858360		0					OTHER		complex event
3-325	3	138901006	139123824	Misassembly	NONE	138901006	139123824		0					OTHER		complex event
3-327	3	139313720	139351269	Misassembly	NONE	139313720	139351269		0					OTHER		complex event
3-328	3	139411950	139485951	Misassembly	NONE	139411950	139485951		0					OTHER		complex event
3-329	3	139576070	139639254	Misassembly	NONE	139576070	139639254		0					OTHER		complex event
3-331	3	139830820	139859980	Misassembly	NONE	139830820	139859980		0					OTHER		complex event
3-332	3	140167386	140328681	Misassembly	NONE	140167386	140328681		0					OTHER		complex event
3-334	3	140662625	140855174	Misassembly	NONE	140662625	140855174		0					OTHER		complex event
3-335	3	141091193	141264408	Misassembly	NONE	141091193	141264408		0					OTHER		complex event
3-337	3	141325361	141396640	Misassembly	NONE	141325361	141396640		0					OTHER		complex event
3-339	3	141922520	142046221	Misassembly	NONE	141922520	142046221		0					OTHER		complex event
3-340	3	142106391	142275647	Misassembly	NONE	142106391	142275647		0					OTHER		complex event
3-341	3	142459465	142638986	Misassembly	NONE	142459465	142638986		0					OTHER		complex event
3-344	3	144100784	144145230	Misassembly	NONE	144100784	144145230		0					OTHER		complex event
3-345	3	144528586	144840500	Misassembly	NONE	144528586	144840500		0					OTHER		complex event
3-346	3	144991941	145040293	Misassembly	NONE	144991941	145040293		0					OTHER		complex event
3-350	3	147117194	147217950	Misassembly	NONE	147117194	147217950		0					OTHER		complex event
3-358	3	149624871	149774817	Misassembly	NONE	149624871	149774817		0					OTHER		complex event
3-369	3	158116963	158203490	Misassembly	NONE	158116963	158203490		0					OTHER		complex event
3-370	3	158476799	158518926	Misassembly	NONE	158476799	158518926		0					OTHER		complex event
3-371	3	160459007	160527298	Misassembly	NONE	160459007	160527298		0					OTHER		complex event
3-375	3	161130234	161256293	Misassembly	NONE	161130234	161256293		0					OTHER		complex event
3-381	3	163343259	163421654	Misassembly	NONE	163343259	163421654		0					OTHER		complex event
3-382	3	163468048	163591920	Misassembly	NONE	163468048	163591920		0					OTHER		complex event
3-383	3	163641250	163825625	Misassembly	NONE	163641250	163825625		0					OTHER		complex event
3-386	3	164212012	164279115	Misassembly	NONE	164212012	164279115		0					OTHER		complex event
3-389	3	164885184	165229074	Misassembly	NONE	164885184	165229074		0					OTHER		complex event
3-390	3	167514831	167582027	Misassembly	NONE	167514831	167582027		0					OTHER		complex event
3-399	3	173949951	174013340	Misassembly	NONE	173949951	174013340		0					OTHER		complex event
3-402	3	174643659	174865202	Misassembly	NONE	174643659	174865202		0					OTHER		complex event
3-406	3	175838138	175877296	Misassembly	NONE	175838138	175877296		0					OTHER		complex event
3-407	3	176020493	176137313	Misassembly	NONE	176020493	176137313		0					OTHER		complex event
3-409	3	176492401	176571824	Misassembly	NONE	176492401	176571824		0					OTHER		complex event
3-410	3	179237238	179287784	Misassembly	NONE	179237238	179287784		0					OTHER		complex event

3-412	3	181948030	182005816	Misassembly	NONE	181948030	182005816		0					OTHER		complex event
3-415	3	182528068	182921363	Misassembly	NONE	182528068	182921363		0					OTHER		complex event
3-419	3	183623869	183952825	Misassembly	NONE	183623869	183952825		0					OTHER		complex event
3-420	3	184365138	184482654	Misassembly	NONE	184365138	184482654		0					OTHER		complex event
3-421	3	184569726	184666585	Misassembly	NONE	184569726	184666585		0					OTHER		complex event
3-423	3	184860293	184960811	Misassembly	NONE	184860293	184960811		0					OTHER		complex event
3-426	3	185125832	185295261	Misassembly	NONE	185125832	185295261		0					OTHER		complex event
3-427	3	185370070	185429129	Misassembly	NONE	185370070	185429129		0					OTHER		complex event
3-428	3	185508174	185636205	Misassembly	NONE	185508174	185636205		0					OTHER		complex event
3-429	3	185779209	185846551	Misassembly	NONE	185779209	185846551		0					OTHER		complex event
3-430	3	185933031	185974211	Misassembly	NONE	185933031	185974211		0					OTHER		complex event
3-432	3	186089679	186221338	Misassembly	NONE	186089679	186221338		0					OTHER		complex event
3-433	3	186285221	186531627	Misassembly	NONE	186285221	186531627		0					OTHER		complex event
3-434	3	186667732	186763055	Misassembly	NONE	186667732	186763055		0					OTHER		complex event
3-435	3	186893361	187150528	Misassembly	NONE	186893361	187150528		0					OTHER		complex event
3-440	3	187837173	187876042	Misassembly	NONE	187837173	187876042		0					OTHER		complex event
3-441	3	188280638	188533778	Misassembly	NONE	188280638	188533778		0					OTHER		complex event
3-442	3	188679766	188810748	Misassembly	NONE	188679766	188810748		0					OTHER		complex event
3-446	3	189268683	189304883	Misassembly	NONE	189268683	189304883		0					OTHER		complex event
3-447	3	189456003	189525293	Misassembly	NONE	189456003	189525293		0					OTHER		complex event
3-451	3	194481539	194555073	Misassembly	NONE	194481539	194555073		0					OTHER		complex event
3-452	3	194692495	194732652	Misassembly	NONE	194692495	194732652		0					OTHER		complex event
3-459	3	200332398	200398302	Misassembly	NONE	200332398	200398302		0					OTHER		complex event
3-463	3	201274434	201321526	Misassembly	NONE	201274434	201321526		0					OTHER		complex event
3-464	3	201544127	201698074	Misassembly	NONE	201544127	201698074		0					OTHER		complex event
3-468	3	202354024	202413407	Misassembly	NONE	202354024	202413407		0					OTHER		complex event
3-469	3	202501675	202645004	Misassembly	NONE	202501675	202645004		0					OTHER		complex event
3-470	3	202961735	203069617	Misassembly	NONE	202961735	203069617		0					OTHER		complex event
3-471	3	203106663	203294796	Misassembly	NONE	203106663	203294796		0					OTHER		complex event
3-474	3	203647768	203918862	Misassembly	NONE	203647768	203918862		0					OTHER		complex event
3-475	3	203951807	204016837	Misassembly	NONE	203951807	204016837		0					OTHER		complex event
3-476	3	204138688	204424782	Misassembly	NONE	204138688	204424782		0					OTHER		complex event
3-479	3	205087180	205301854	Misassembly	NONE	205087180	205301854		0					OTHER		complex event
3-480	3	205454973	205656258	Misassembly	NONE	205454973	205656258		0					OTHER		complex event
3-482	3	206552297	206629323	Misassembly	NONE	206552297	206629323		0					OTHER		complex event
3-484	3	207781801	207837685	Misassembly	NONE	207781801	207837685		0					OTHER		
3-485	3	208032242	208068827	Misassembly	NONE	208032242	208068827		0					OTHER		complex event
3-489	3	208403760	208460943	Misassembly	NONE	208403760	208460943		0					OTHER		complex event
3-490	3	208836850	208960240	Misassembly	NONE	208836850	208960240		0					OTHER		complex event
3-491	3	209071288	209141208	Misassembly	NONE	209071288	209141208		0					OTHER		complex event
3-492	3	209469995	209718913	Misassembly	NONE	209469995	209718913		0					OTHER		complex event
3-493	3	209832021	209940571	Misassembly	NONE	209832021	209940571		0					OTHER		complex event
3-496	3	210455041	210556761	Misassembly	NONE	210455041	210556761		0					OTHER		complex event
3-497	3	210662657	210721540	Misassembly	NONE	210662657	210721540		0					OTHER		complex event
3-498	3	210895850	210984650	Misassembly	NONE	210895850	210984650		0					OTHER		complex event
3-501	3	211842163	212261359	Misassembly	NONE	211842163	212261359		0					OTHER		complex event
3-502	3	212535172	212676410	Misassembly	NONE	212535172	212676410		0					OTHER		complex event
3-504	3	212870026	213123517	Misassembly	NONE	212870026	213123517		0					OTHER		complex event
3-505	3	213640370	213818257	Misassembly	NONE	213640370	213818257		0					OTHER		complex event
3-507	3	214138443	214219809	Misassembly	NONE	214138443	214219809		0					OTHER		complex event
3-508	3	214339581	214431521	Misassembly	NONE	214339581	214431521		0					OTHER		complex event
3-512	3	215171364	215270865	Misassembly	NONE	215171364	215270865		0					OTHER		complex event
3-513	3	215417345	215453590	Misassembly	NONE	215417345	215453590		0					OTHER		complex event
3-517	3	215890552	216067688	Misassembly	NONE	215890552	216067688		0					OTHER		complex event
3-519	3	216697652	216808694	Misassembly	NONE	216697652	216808694		0					OTHER		complex event
3-520	3	216855534	216901974	Misassembly	NONE	216855534	216901974		0					OTHER		complex event
3-522	3	217689253	217813452	Misassembly	NONE	217689253	217813452		0					OTHER		complex event
3-523	3	217851335	218030682	Misassembly	NONE	217851335	218030682		0					OTHER		complex event
3-525	3	218764554	219061747	Misassembly	NONE	218764554	219061747		0					OTHER		complex event
3-527	3	219352691	219514109	Misassembly	NONE	219352691	219514109		0					OTHER		complex event
3-531	3	220421161	220599780	Misassembly	NONE	220421161	220599780		0					OTHER		complex event
3-537	3	224005560	224076930	Misassembly	NONE	224005560	224076930		0					OTHER		complex event
3-538	3	224951190	225179460	Misassembly	NONE	224951190	225179460		0					OTHER		complex event
3-539	3	225566359	225599083	Misassembly	NONE	225566359	225599083		0					OTHER		complex event
3-540	3	225702344	225734642	Misassembly	NONE	225702344	225734642		0					OTHER		complex event
3-541	3	226249340	226336612	Misassembly	NONE	226249340	226336612		0					OTHER		complex event
3-544	3	226601998	226722657	Misassembly	NONE	226601998	226722657		0					OTHER		complex event
3-546	3	227100028	227243918	Misassembly	NONE	227100028	227243918		0					OTHER		complex event
3-548	3	227891732	228173262	Misassembly	NONE	227891732	228173262		0					OTHER		complex event
3-549	3	228198451	228447041	Misassembly	NONE	228198451	228447041		0					OTHER		complex event
3-553	3	228911061	229019615	Misassembly	NONE	228911061	229019615		0					OTHER		complex event
4-2	4	5121548	5403592	Misassembly	NONE	5121548	5403592		0					OTHER		complex event
4-4	4	5475537	5599723	Misassembly	NONE	5475537	5599723		0					OTHER		complex event
4-8	4	6233455	6290809	Misassembly	NONE	6233455	6290809		0					OTHER		complex event
4-10	4	6557744	6586837	Misassembly	NONE	6557744	6586837		0					OTHER		complex event
4-12	4	6722284	6837219	Misassembly	NONE	6722284	6837219		0					OTHER		complex event
4-13	4	6938790	7029034	Misassembly	NONE	6938790	7029034		0					OTHER		complex event
4-14	4	7087553	7147173	Misassembly	NONE	7087553	7147173		0					OTHER		complex event
4-15	4	7186732	7289072	Misassembly	NONE	7186732	7289072		0					OTHER		complex event
4-16	4	7358969	7416409	Misassembly	NONE	7358969	7416409		0					OTHER		complex event
4-17	4	7437851	7522123	Misassembly	NONE	7437851	7522123		0					OTHER		complex event
4-18	4	7576204	7842642	Misassembly	NONE	7576204	7842642		0					OTHER		complex event
4-21	4	8261179	8501011	Misassembly	NONE	8261179	8501011		0					OTHER		complex event
4-22	4	8532506	8757876	Misassembly	NONE	8532506	8757876		0					OTHER		complex event
4-23	4	8931173	9238630	Misassembly	NONE	8931173	9238630		0					OTHER		complex event
4-25	4	9379709	9554182	Misassembly	NONE	9379709	9554182		0					OTHER		complex event
4-29	4	10920829	11147614	Misassembly	NONE	10920829	11147614		0					OTHER		complex event
4-31	4	11387661	11483901	Misassembly	NONE	11387661	11483901		0					OTHER		complex event
4-32	4	11529706	11649342	Misassembly	NONE	11529706	11649342		0					OTHER		complex event
4-33	4	11876514	11961435	Misassembly	NONE	11876514	11961435		0					OTHER		complex event
4-34	4	12027123	12194184	Misassembly	NONE	12027123	12194184		0					OTHER		complex event

4-35	4	12257425	12349527	Misassembly	NONE	12257425	12349527		0					OTHER		complex event
4-38	4	13870346	13944280	Misassembly	NONE	13870346	13944280		0					OTHER		complex event
4-39	4	14921834	15088744	Misassembly	NONE	14921834	15088744		0					OTHER		complex event
4-40	4	15217136	15337805	Misassembly	NONE	15217136	15337805		0					OTHER		complex event
4-42	4	18009224	18135633	Misassembly	NONE	18009224	18135633		0					OTHER		complex event
4-45	4	18331112	18361091	Misassembly	NONE	18331112	18361091		0					OTHER		complex event
4-47	4	18463596	18567566	Misassembly	NONE	18463596	18567566		0					OTHER		complex event
4-50	4	19112396	19219888	Misassembly	NONE	19112396	19219888		0					OTHER		complex event
4-51	4	19533832	19808218	Misassembly	NONE	19533832	19808218		0					OTHER		complex event
4-54	4	20755155	21068126	Misassembly	NONE	20755155	21068126		0					OTHER		complex event
4-56	4	23216321	23380709	Misassembly	NONE	23216321	23380709		0					OTHER		complex event
4-58	4	25618267	25650814	Misassembly	NONE	25618267	25650814		0					OTHER		complex event
4-65	4	29949803	30028188	Misassembly	NONE	29949803	30028188		0					OTHER		complex event
4-66	4	30153892	30427841	Misassembly	NONE	30153892	30427841		0					OTHER		complex event
4-67	4	30467041	30611541	Misassembly	NONE	30467041	30611541		0					OTHER		complex event
4-68	4	30664837	30708383	Misassembly	NONE	30664837	30708383		0					OTHER		complex event
4-69	4	30755835	30856394	Misassembly	NONE	30755835	30856394		0					OTHER		complex event
4-71	4	32435164	32493715	Misassembly	NONE	32435164	32493715		0					OTHER		complex event
4-72	4	32553173	32694750	Misassembly	NONE	32553173	32694750		0					OTHER		complex event
4-73	4	36128992	36172265	Misassembly	NONE	36128992	36172265		0					OTHER		complex event
4-74	4	36372570	36650050	Misassembly	NONE	36372570	36650050		0					OTHER		complex event
4-77	4	37060935	37258191	Misassembly	NONE	37060935	37258191		0					OTHER		complex event
4-80	4	37802274	37851808	Misassembly	NONE	37802274	37851808		0					OTHER		complex event
4-81	4	38029597	38117642	Misassembly	NONE	38029597	38117642		0					OTHER		complex event
4-85	4	38882684	38969375	Misassembly	NONE	38882684	38969375		0					OTHER		complex event
4-86	4	39583674	39674402	Misassembly	NONE	39583674	39674402		0					OTHER		complex event
4-88	4	41439318	41483502	Misassembly	NONE	41439318	41483502		0					OTHER		complex event
4-90	4	41807233	41883137	Misassembly	NONE	41807233	41883137		0					OTHER		complex event
4-94	4	42166813	42217384	Misassembly	NONE	42166813	42217384		0					OTHER		complex event
4-95	4	42241651	42314714	Misassembly	NONE	42241651	42314714		0					OTHER		complex event
4-97	4	42588815	42733964	Misassembly	NONE	42588815	42733964		0					OTHER		complex event
4-98	4	42925556	42995697	Misassembly	NONE	42925556	42995697		0					OTHER		complex event
4-99	4	43170641	43221152	Misassembly	NONE	43170641	43221152		0					OTHER		complex event
4-102	4	43474995	43499457	Misassembly	NONE	43474995	43499457		0					OTHER		complex event
4-103	4	43565708	43599205	Misassembly	NONE	43565708	43599205		0					OTHER		complex event
4-105	4	46199929	46491101	Misassembly	NONE	46199929	46491101		0					OTHER		complex event
4-106	4	47715614	47807720	Misassembly	NONE	47715614	47807720		0					OTHER		complex event
4-113	4	49745056	49920728	Misassembly	NONE	49745056	49920728		0					OTHER		complex event
4-117	4	51502508	51943790	Misassembly	NONE	51502508	51943790		0					OTHER		complex event
4-118	4	52200586	52271628	Misassembly	NONE	52200586	52271628		0					OTHER		complex event
4-120	4	52410510	52496881	Misassembly	NONE	52410510	52496881		0					OTHER		complex event
4-124	4	53521804	53824675	Misassembly	NONE	53521804	53824675		0					OTHER		complex event
4-126	4	54105397	54199752	Misassembly	NONE	54105397	54199752		0					OTHER		complex event
4-128	4	54397015	54744064	Misassembly	NONE	54397015	54744064		0					OTHER		complex event
4-129	4	55193875	55311226	Misassembly	NONE	55193875	55311226		0					OTHER		complex event
4-133	4	60309434	60406440	Misassembly	NONE	60309434	60406440		0					OTHER		complex event
4-135	4	60590438	60750093	Misassembly	NONE	60590438	60750093		0					OTHER		complex event
4-137	4	60793943	60857347	Misassembly	NONE	60793943	60857347		0					OTHER		complex event
4-138	4	61548157	61621476	Misassembly	NONE	61548157	61621476		0					OTHER		complex event
4-139	4	61695419	62055429	Misassembly	NONE	61695419	62055429		0					OTHER		complex event
4-141	4	62192814	62685708	Misassembly	NONE	62192814	62685708		0					OTHER		complex event
4-143	4	62985649	63725889	Misassembly	NONE	62985649	63725889		0					OTHER		complex event
4-148	4	64484843	64564368	Misassembly	NONE	64484843	64564368		0					OTHER		complex event
4-152	4	67689203	67726196	Misassembly	NONE	67689203	67726196		0					OTHER		complex event
4-153	4	67847073	68092211	Misassembly	NONE	67847073	68092211		0					OTHER		complex event
4-155	4	68632027	68723990	Misassembly	NONE	68632027	68723990		0					OTHER		complex event
4-156	4	68840637	68890307	Misassembly	NONE	68840637	68890307		0					OTHER		complex event
4-160	4	69370536	69443116	Misassembly	NONE	69370536	69443116		0					OTHER		complex event
4-161	4	69479017	69601523	Misassembly	NONE	69479017	69601523		0					OTHER		complex event
4-162	4	69650375	69736165	Misassembly	NONE	69650375	69736165		0					OTHER		complex event
4-163	4	70227728	70567357	Misassembly	NONE	70227728	70567357		0					OTHER		complex event
4-164	4	70653496	70730527	Misassembly	NONE	70653496	70730527		0					OTHER		complex event
4-165	4	70836008	71160792	Misassembly	NONE	70836008	71160792		0					OTHER		complex event
4-166	4	71270792	71302407	Misassembly	NONE	71270792	71302407		0					OTHER		complex event
4-167	4	71362438	71467068	Misassembly	NONE	71362438	71467068		0					OTHER		complex event
4-168	4	71548950	71915285	Misassembly	NONE	71548950	71915285		0					OTHER		complex event
4-170	4	72371925	72511336	Misassembly	NONE	72371925	72511336		0					OTHER		complex event
4-171	4	72619940	72728627	Misassembly	NONE	72619940	72728627		0					OTHER		complex event
4-172	4	72769012	72911400	Misassembly	NONE	72769012	72911400		0					OTHER		complex event
4-173	4	73078570	73192422	Misassembly	NONE	73078570	73192422		0					OTHER		complex event
4-175	4	73546079	73820514	Misassembly	NONE	73546079	73820514		0					OTHER		complex event
4-178	4	76115569	76191069	Misassembly	NONE	76115569	76191069		0					OTHER		complex event
4-180	4	76576519	76742304	Misassembly	NONE	76576519	76742304		0					OTHER		complex event
4-181	4	76866229	77126264	Misassembly	NONE	76866229	77126264		0					OTHER		complex event
4-183	4	77677088	77767910	Misassembly	NONE	77677088	77767910		0					OTHER		complex event
4-184	4	77843280	77896792	Misassembly	NONE	77843280	77896792		0					OTHER		complex event
4-185	4	79345191	79469473	Misassembly	NONE	79345191	79469473		0					OTHER		complex event
4-186	4	79747073	79777050	Misassembly	NONE	79747073	79777050		0					OTHER		complex event<br>possible inversion
4-188	4	80089192	80187682	Misassembly	NONE	80089192	80187682		0					OTHER		complex event
4-189	4	80298948	80447945	Misassembly	NONE	80298948	80447945		0					OTHER		complex event
4-191	4	80658093	80729437	Misassembly	NONE	80658093	80729437		0					OTHER		complex event
4-194	4	81214317	81263110	Misassembly	NONE	81214317	81263110		0					OTHER		complex event
4-195	4	81392625	81513155	Misassembly	NONE	81392625	81513155		0					OTHER		complex event
4-198	4	82230527	82297094	Misassembly	NONE	82230527	82297094		0					OTHER		complex event
4-199	4	82392308	82455405	Misassembly	NONE	82392308	82455405		0					OTHER		complex event
4-203	4	83252987	83328920	Misassembly	NONE	83252987	83328920		0					OTHER		complex event
4-204	4	83669346	83717531	Misassembly	NONE	83669346	83717531		0					OTHER		complex event
4-205	4	83856638	84105038	Misassembly	NONE	83856638	84105038		0					OTHER		complex event
4-209	4	84910852	85318502	Misassembly	NONE	84910852	85318502		0					OTHER		complex event

4-210	4	85454721	85576081	Misassembly	NONE	85454721	85576081		0					OTHER		complex event
4-212	4	85950643	86032463	Misassembly	NONE	85950643	86032463		0					OTHER		complex event
4-214	4	86121473	86238880	Misassembly	NONE	86121473	86238880		0					OTHER		complex event
4-217	4	86743271	86820412	Misassembly	NONE	86743271	86820412		0					OTHER		complex event
4-218	4	86871807	86972395	Misassembly	NONE	86871807	86972395		0					OTHER		complex event
4-220	4	87323390	87506860	Misassembly	NONE	87323390	87506860		0					OTHER		complex event
4-221	4	87860209	87941275	Misassembly	NONE	87860209	87941275		0					OTHER		complex event
4-225	4	88390093	88503467	Misassembly	NONE	88390093	88503467		0					OTHER		complex event
4-226	4	88733983	88766084	Misassembly	NONE	88733983	88766084		0					OTHER		complex event
4-227	4	88924279	88998205	Misassembly	NONE	88924279	88998205		0					OTHER		complex event
4-229	4	89316824	89512480	Misassembly	NONE	89316824	89512480		0					OTHER		complex event
4-230	4	89710529	89792133	Misassembly	NONE	89710529	89792133		0					OTHER		complex event
4-231	4	89962534	90306575	Misassembly	NONE	89962534	90306575		0					OTHER		complex event
4-232	4	90707505	90753246	Misassembly	NONE	90707505	90753246		0					OTHER		complex event
4-233	4	90959045	91125449	Misassembly	NONE	90959045	91125449		0					OTHER		complex event
4-235	4	91771992	91921298	Misassembly	NONE	91771992	91921298		0					OTHER		complex event
4-238	4	92389508	92519189	Misassembly	NONE	92389508	92519189		0					OTHER		complex event
4-239	4	92612614	92658192	Misassembly	NONE	92612614	92658192		0					OTHER		complex event
4-240	4	93133806	93208216	Misassembly	NONE	93133806	93208216		0					OTHER		complex event
4-245	4	94737948	94774009	Misassembly	NONE	94737948	94774009		0					OTHER		complex event
4-246	4	94844510	94986480	Misassembly	NONE	94844510	94986480		0					OTHER		complex event
4-247	4	95121160	95381832	Misassembly	NONE	95121160	95381832		0					OTHER		complex event
4-253	4	96580176	96645878	Misassembly	NONE	96580176	96645878		0					OTHER		complex event
4-254	4	96964426	97080227	Misassembly	NONE	96964426	97080227		0					OTHER		complex event
4-256	4	98024002	98147612	Misassembly	NONE	98024002	98147612		0					OTHER		complex event with deletion
4-257	4	98271179	98288494	Misassembly	NONE	98271179	98288494		0					OTHER		complex event
4-258	4	98780135	98842872	Misassembly	NONE	98780135	98842872		0					OTHER		complex event
4-261	4	100145647	100400771	Misassembly	NONE	100145647	100400771		0					OTHER		complex event
4-263	4	100766983	100883249	Misassembly	NONE	100766983	100883249		0					OTHER		complex event
4-268	4	101276575	101483289	Misassembly	NONE	101276575	101483289		0					OTHER		complex event
4-269	4	101558210	101696454	Misassembly	NONE	101558210	101696454		0					OTHER		complex event
4-270	4	101951322	102289189	Misassembly	NONE	101951322	102289189		0					OTHER		complex event
4-271	4	102505685	102704174	Misassembly	NONE	102505685	102704174		0					OTHER		complex event
4-273	4	103180352	103238914	Misassembly	NONE	103180352	103238914		0					OTHER		complex event
4-278	4	106926303	107023410	Misassembly	NONE	106926303	107023410		0					OTHER		complex event
4-279	4	107592267	107729303	Misassembly	NONE	107592267	107729303		0					OTHER		complex event
4-281	4	109593734	109656363	Misassembly	NONE	109593734	109656363		0					OTHER		complex event
4-283	4	110785052	110934656	Misassembly	NONE	110785052	110934656		0					OTHER		complex event
4-286	4	111418413	111543906	Misassembly	NONE	111418413	111543906		0					OTHER		complex event
4-287	4	111719496	111923580	Misassembly	NONE	111719496	111923580		0					OTHER		complex event
4-288	4	112351588	112546313	Misassembly	NONE	112351588	112546313		0					OTHER		complex event
4-289	4	112773218	112979193	Misassembly	NONE	112773218	112979193		0					OTHER		complex event
4-291	4	113197177	113373089	Misassembly	NONE	113197177	113373089		0					OTHER		complex event
4-292	4	113626696	113825360	Misassembly	NONE	113626696	113825360		0					OTHER		complex event
4-294	4	114124126	114260152	Misassembly	NONE	114124126	114260152		0					OTHER		complex event
4-295	4	114339313	114386018	Misassembly	NONE	114339313	114386018		0					OTHER		complex event
4-297	4	114944202	115090960	Misassembly	NONE	114944202	115090960		0					OTHER		complex event
4-300	4	116473291	116554509	Misassembly	NONE	116473291	116554509		0					OTHER		complex event
4-302	4	117020938	117185339	Misassembly	NONE	117020938	117185339		0					OTHER		complex event
4-303	4	117810170	117886196	Misassembly	NONE	117810170	117886196		0					OTHER		complex event
4-304	4	118026846	118115338	Misassembly	NONE	118026846	118115338		0					OTHER		complex event
4-305	4	118131558	118196294	Misassembly	NONE	118131558	118196294		0					OTHER		complex event
4-306	4	118333069	118524906	Misassembly	NONE	118333069	118524906		0					OTHER		complex event
4-307	4	119544268	119874813	Misassembly	NONE	119544268	119874813		0					OTHER		complex event
4-308	4	119994459	120060817	Misassembly	NONE	119994459	120060817		0					OTHER		complex event
4-310	4	120270843	120382042	Misassembly	NONE	120270843	120382042		0					OTHER		complex event
4-311	4	120478040	120558884	Misassembly	NONE	120478040	120558884		0					OTHER		complex event
4-312	4	120627623	120813136	Misassembly	NONE	120627623	120813136		0					OTHER		complex event
4-316	4	121763645	121842422	Misassembly	NONE	121763645	121842422		0					OTHER		complex event
4-317	4	122312108	122548199	Misassembly	NONE	122312108	122548199		0					OTHER		complex event
4-318	4	122567825	122773942	Misassembly	NONE	122567825	122773942		0					OTHER		complex event
4-320	4	122940654	123280121	Misassembly	NONE	122940654	123280121		0					OTHER		complex event
4-324	4	124061190	124269865	Misassembly	NONE	124061190	124269865		0					OTHER		complex event
4-328	4	133850556	133901667	Misassembly	NONE	133850556	133901667		0					OTHER		complex event
4-329	4	133924666	134066769	Misassembly	NONE	133924666	134066769		0					OTHER		complex event
4-331	4	134602336	135001497	Misassembly	NONE	134602336	135001497		0					OTHER		complex event
4-332	4	135347546	135390185	Misassembly	NONE	135347546	135390185		0					OTHER		complex event
4-334	4	135684515	135923411	Misassembly	NONE	135684515	135923411		0					OTHER		complex event
4-335	4	136045159	136117465	Misassembly	NONE	136045159	136117465		0					OTHER		complex event
4-336	4	136181751	136229899	Misassembly	NONE	136181751	136229899		0					OTHER		complex event
4-338	4	136478083	136626609	Misassembly	NONE	136478083	136626609		0					OTHER		complex event
4-339	4	136846899	136917821	Misassembly	NONE	136846899	136917821		0					OTHER		complex event
4-345	4	138473964	138543462	Misassembly	NONE	138473964	138543462		0					OTHER		complex event
4-346	4	139478996	139776450	Misassembly	NONE	139478996	139776450		0					OTHER		complex event
4-347	4	139922802	140068022	Misassembly	NONE	139922802	140068022		0					OTHER		complex event
4-350	4	140418037	140485729	Misassembly	NONE	140418037	140485729		0					OTHER		complex event
4-351	4	140645838	140927017	Misassembly	NONE	140645838	140927017		0					OTHER		complex event
4-352	4	141058632	141146212	Misassembly	NONE	141058632	141146212		0					OTHER		complex event
4-353	4	141276446	141346012	Misassembly	NONE	141276446	141346012		0					OTHER		complex event
4-355	4	142128085	142165896	Misassembly	NONE	142128085	142165896		0					OTHER		complex event
4-357	4	142578966	142918864	Misassembly	NONE	142578966	142918864		0					OTHER		complex event
4-358	4	143331734	143496167	Misassembly	NONE	143331734	143496167		0					OTHER		complex event
4-359	4	143558589	143597756	Misassembly	NONE	143558589	143597756		0					OTHER		complex event possible inversion
4-361	4	143922501	144100330	Misassembly	NONE	143922501	144100330		0					OTHER		complex event
4-362	4	144246402	144460886	Misassembly	NONE	144246402	144460886		0					OTHER		complex event
4-363	4	144806496	144908407	Misassembly	NONE	144806496	144908407		0					OTHER		complex event
4-364	4	145488791	145651071	Misassembly	NONE	145488791	145651071		0					OTHER		complex event
4-366	4	145836221	145932663	Misassembly	NONE	145836221	145932663		0					OTHER		complex event

4-368	4	146048090	146181648	Misassembly	NONE	146048090	146181648		0					OTHER		complex event possible inversion
4-369	4	146279955	146406756	Misassembly	NONE	146279955	146406756		0					OTHER		complex event
4-374	4	150571867	150760897	Misassembly	NONE	150571867	150760897		0					OTHER		complex event
4-377	4	152994357	153294607	Misassembly	NONE	152994357	153294607		0					OTHER		complex event
4-382	4	155501687	155738684	Misassembly	NONE	155501687	155738684		0					OTHER		complex event
4-383	4	155839302	155990156	Misassembly	NONE	155839302	155990156		0					OTHER		complex event
4-384	4	156017965	156054365	Misassembly	NONE	156017965	156054365		0					OTHER		complex event
4-385	4	156124293	156223108	Misassembly	NONE	156124293	156223108		0					OTHER		complex event
4-387	4	156762999	156839854	Misassembly	NONE	156762999	156839854		0					OTHER		complex event
4-389	4	156965413	157038299	Misassembly	NONE	156965413	157038299		0					OTHER		complex event
4-390	4	157174700	157445814	Misassembly	NONE	157174700	157445814		0					OTHER		complex event
4-392	4	157753538	157853953	Misassembly	NONE	157753538	157853953		0					OTHER		complex event
4-395	4	158392455	158581180	Misassembly	NONE	158392455	158581180		0					OTHER		complex event
4-397	4	158839114	158932700	Misassembly	NONE	158839114	158932700		0					OTHER		complex event
4-398	4	158974688	159108885	Misassembly	NONE	158974688	159108885		0					OTHER		complex event
4-400	4	159208355	159275698	Misassembly	NONE	159208355	159275698		0					OTHER		complex event
4-403	4	159773379	159965098	Misassembly	NONE	159773379	159965098		0					OTHER		complex event
4-404	4	159997238	160040344	Misassembly	NONE	159997238	160040344		0					OTHER		complex event
4-407	4	160253646	160364627	Misassembly	NONE	160253646	160364627		0					OTHER		complex event
4-408	4	160587124	160644868	Misassembly	NONE	160587124	160644868		0					OTHER		complex event
4-409	4	160843544	161043058	Misassembly	NONE	160843544	161043058		0					OTHER		complex event
4-411	4	161230472	161518783	Misassembly	NONE	161230472	161518783		0					OTHER		complex event
4-412	4	161759126	161826624	Misassembly	NONE	161759126	161826624		0					OTHER		complex event
4-414	4	161992434	162192107	Misassembly	NONE	161992434	162192107		0					OTHER		complex event
4-416	4	162304571	162341482	Misassembly	NONE	162304571	162341482		0					OTHER		complex event
4-418	4	162528029	162631181	Misassembly	NONE	162528029	162631181		0					OTHER		complex event
4-420	4	163213989	163351393	Misassembly	NONE	163213989	163351393		0					OTHER		complex event
4-421	4	163459565	163677598	Misassembly	NONE	163459565	163677598		0					OTHER		complex event
4-422	4	163785639	163828241	Misassembly	NONE	163785639	163828241		0					OTHER		complex event
4-424	4	164077581	164124612	Misassembly	NONE	164077581	164124612		0					OTHER		complex event
4-426	4	164500431	164706314	Misassembly	NONE	164500431	164706314		0					OTHER		complex event
4-428	4	165161878	165273233	Misassembly	NONE	165161878	165273233		0					OTHER		complex event
4-429	4	165533682	165610933	Misassembly	NONE	165533682	165610933		0					OTHER		complex event
4-430	4	166247167	166274995	Misassembly	NONE	166247167	166274995		0					OTHER		complex event
4-431	4	166318864	166447169	Misassembly	NONE	166318864	166447169		0					OTHER		complex event
4-434	4	166961134	167097218	Misassembly	NONE	166961134	167097218		0					OTHER		complex event
4-435	4	167151123	167254778	Misassembly	NONE	167151123	167254778		0					OTHER		complex event
4-436	4	167743296	167817981	Misassembly	NONE	167743296	167817981		0					OTHER		complex event
4-437	4	167951865	168072920	Misassembly	NONE	167951865	168072920		0					OTHER		complex event
4-442	4	168696189	168803351	Misassembly	NONE	168696189	168803351		0					OTHER		complex event
4-443	4	168852003	168999957	Misassembly	NONE	168852003	168999957		0					OTHER		complex event
4-444	4	169287162	169334011	Misassembly	NONE	169287162	169334011		0					OTHER		complex event
4-446	4	169549557	169597150	Misassembly	NONE	169549557	169597150		0					OTHER		complex event
4-447	4	169717370	169872615	Misassembly	NONE	169717370	169872615		0					OTHER		complex event
4-448	4	170161495	170286552	Misassembly	NONE	170161495	170286552		0					OTHER		complex event
4-452	4	172765783	172868437	Misassembly	NONE	172765783	172868437		0					OTHER		complex event
4-453	4	173144057	173204622	Misassembly	NONE	173144057	173204622		0					OTHER		complex event
4-454	4	173310552	173679824	Misassembly	NONE	173310552	173679824		0					OTHER		complex event
4-455	4	173707462	173769956	Misassembly	NONE	173707462	173769956		0					OTHER		complex event
4-457	4	174060033	174194722	Misassembly	NONE	174060033	174194722		0					OTHER		complex event
4-459	4	174517601	174568328	Misassembly	NONE	174517601	174568328		0					OTHER		complex event
4-463	4	174997804	175049057	Misassembly	NONE	174997804	175049057		0					OTHER		complex event
4-465	4	175245386	175547956	Misassembly	NONE	175245386	175547956		0					OTHER		complex event
4-467	4	175985523	176019917	Misassembly	NONE	175985523	176019917		0					OTHER		complex event
4-470	4	181808894	181869926	Misassembly	NONE	181808894	181869926		0					OTHER		complex event
4-472	4	182505879	182573432	Misassembly	NONE	182505879	182573432		0					OTHER		complex event
4-473	4	182610146	182795912	Misassembly	NONE	182610146	182795912		0					OTHER		complex event
4-474	4	182870899	182994282	Misassembly	NONE	182870899	182994282		0					OTHER		complex event
4-476	4	183557381	183597631	Misassembly	NONE	183557381	183597631		0					OTHER		complex event
4-477	4	183641250	183731938	Misassembly	NONE	183641250	183731938		0					OTHER		complex event
4-479	4	185787654	185915266	Misassembly	NONE	185787654	185915266		0					OTHER		complex event
4-480	4	185957783	186030559	Misassembly	NONE	185957783	186030559		0					OTHER		complex event
4-483	4	186742700	186911676	Misassembly	NONE	186742700	186911676		0					OTHER		complex event
4-485	4	188067010	188143665	Misassembly	NONE	188067010	188143665		0					OTHER		complex event
4-487	4	189525776	189614309	Misassembly	NONE	189525776	189614309		0					OTHER		complex event
4-488	4	189667012	189764007	Misassembly	NONE	189667012	189764007		0					OTHER		complex event
4-491	4	190420076	190535941	Misassembly	NONE	190420076	190535941		0					OTHER		complex event
4-492	4	190743297	190830867	Misassembly	NONE	190743297	190830867		0					OTHER		complex event
4-493	4	191139349	191227931	Misassembly	NONE	191139349	191227931		0					OTHER		complex event
4-494	4	191579148	191632309	Misassembly	NONE	191579148	191632309		0					OTHER		complex event
4-496	4	191944044	192134389	Misassembly	NONE	191944044	192134389		0					OTHER		complex event
4-497	4	192189455	192272138	Misassembly	NONE	192189455	192272138		0					OTHER		complex event
4-498	4	192465564	192555518	Misassembly	NONE	192465564	192555518		0					OTHER		complex event
4-499	4	192718449	192824416	Misassembly	NONE	192718449	192824416		0					OTHER		complex event
4-501	4	196165878	196279043	Misassembly	NONE	196165878	196279043		0					OTHER		complex event
4-503	4	197953949	198008255	Misassembly	NONE	197953949	198008255		0					OTHER		complex event
4-504	4	198045462	198172305	Misassembly	NONE	198045462	198172305		0					OTHER		complex event
4-507	4	198705962	198767183	Misassembly	NONE	198705962	198767183		0					OTHER		complex event
4-508	4	198812179	198925830	Misassembly	NONE	198812179	198925830		0					OTHER		complex event
4-509	4	199293569	199501176	Misassembly	NONE	199293569	199501176		0					OTHER		complex event
4-510	4	199531821	200006804	Misassembly	NONE	199531821	200006804		0					OTHER		complex event
4-513	4	200870246	200955728	Misassembly	NONE	200870246	200955728		0					OTHER		complex event
4-514	4	201121593	201264389	Misassembly	NONE	201121593	201264389		0					OTHER		complex event
4-515	4	201360959	201510402	Misassembly	NONE	201360959	201510402		0					OTHER		complex event
4-517	4	201921743	202007231	Misassembly	NONE	201921743	202007231		0					OTHER		complex event
4-519	4	202289452	202528888	Misassembly	NONE	202289452	202528888		0					OTHER		complex event
4-521	4	202811477	203025326	Misassembly	NONE	202811477	203025326		0					OTHER		complex event
4-524	4	203556120	203644400	Misassembly	NONE	203556120	203644400		0					OTHER		complex event
4-525	4	203721535	203753011	Misassembly	NONE	203721535	203753011		0					OTHER		complex event

4-527	4	203965981	204207261	Misassembly	NONE	203965981	204207261		0					OTHER		complex event
4-528	4	204280277	204476221	Misassembly	NONE	204280277	204476221		0					OTHER		complex event
4-529	4	204545880	204601813	Misassembly	NONE	204545880	204601813		0					OTHER		complex event
4-530	4	204601813	204676170	Misassembly	NONE	204601813	204676170		0					OTHER		complex event
4-531	4	205055066	205108244	Misassembly	NONE	205055066	205108244		0					OTHER		complex event
4-533	4	205391316	205476815	Misassembly	NONE	205391316	205476815		0					OTHER		complex event
4-535	4	205642862	205690571	Misassembly	NONE	205642862	205690571		0					OTHER		complex event
4-536	4	205803852	205840891	Misassembly	NONE	205803852	205840891		0					OTHER		complex event
4-541	4	206816588	207202753	Misassembly	NONE	206816588	207202753		0					OTHER		complex event
4-542	4	207305689	207492095	Misassembly	NONE	207305689	207492095		0					OTHER		complex event
4-546	4	208402557	208480679	Misassembly	NONE	208402557	208480679		0					OTHER		complex event
4-547	4	208609833	208875887	Misassembly	NONE	208609833	208875887		0					OTHER		complex event
4-549	4	209236777	209300285	Misassembly	NONE	209236777	209300285		0					OTHER		complex event
4-550	4	209601068	209673926	Misassembly	NONE	209601068	209673926		0					OTHER		complex event
4-551	4	210193631	210294646	Misassembly	NONE	210193631	210294646		0					OTHER		complex event
4-552	4	210363059	210532353	Misassembly	NONE	210363059	210532353		0					OTHER		complex event
4-553	4	210706397	210830787	Misassembly	NONE	210706397	210830787		0					OTHER		complex event
4-554	4	210949403	211044092	Misassembly	NONE	210949403	211044092		0					OTHER		complex event
4-555	4	211112319	211350527	Misassembly	NONE	211112319	211350527		0					OTHER		complex event
4-556	4	211720284	211984445	Misassembly	NONE	211720284	211984445		0					OTHER		complex event
4-557	4	212087707	212141790	Misassembly	NONE	212087707	212141790		0					OTHER		complex event
4-559	4	212661939	212737869	Misassembly	NONE	212661939	212737869		0					OTHER		complex event
4-561	4	213053643	213190266	Misassembly	NONE	213053643	213190266		0					OTHER		complex event
4-563	4	213437362	213482730	Misassembly	NONE	213437362	213482730		0					OTHER		complex event
4-564	4	213840505	213977313	Misassembly	NONE	213840505	213977313		0					OTHER		complex event
4-565	4	214043509	214166842	Misassembly	NONE	214043509	214166842		0					OTHER		complex event
4-566	4	214293223	214422704	Misassembly	NONE	214293223	214422704		0					OTHER		complex event
4-567	4	214501403	214864319	Misassembly	NONE	214501403	214864319		0					OTHER		complex event
4-569	4	215203950	215424514	Misassembly	NONE	215203950	215424514		0					OTHER		complex event
4-571	4	215604881	215686180	Misassembly	NONE	215604881	215686180		0					OTHER		complex event
4-572	4	216333791	216412580	Misassembly	NONE	216333791	216412580		0					OTHER		complex event
4-573	4	216490220	216564958	Misassembly	NONE	216490220	216564958		0					OTHER		complex event
4-576	4	216994211	217075649	Misassembly	NONE	216994211	217075649		0					OTHER		complex event
4-577	4	217134655	217208436	Misassembly	NONE	217134655	217208436		0					OTHER		complex event
4-580	4	218984462	219036543	Misassembly	NONE	218984462	219036543		0					OTHER		complex event
4-583	4	220116118	220506375	Misassembly	NONE	220116118	220506375		0					OTHER		complex event
4-588	4	221789088	221894072	Misassembly	NONE	221789088	221894072		0					OTHER		complex event
4-591	4	222257955	222419193	Misassembly	NONE	222257955	222419193		0					OTHER		complex event
4-595	4	222901096	222990751	Misassembly	NONE	222901096	222990751		0					OTHER		complex event
4-597	4	223419900	223524129	Misassembly	NONE	223419900	223524129		0					OTHER		complex event
4-603	4	224140852	224176650	Misassembly	NONE	224140852	224176650		0					OTHER		complex event
4-604	4	224511933	224599894	Misassembly	NONE	224511933	224599894		0					OTHER		complex event
4-611	4	225320840	225458653	Misassembly	NONE	225320840	225458653		0					OTHER		complex event
4-613	4	225575358	225791563	Misassembly	NONE	225575358	225791563		0					OTHER		complex event
4-616	4	226267628	226349235	Misassembly	NONE	226267628	226349235		0					OTHER		complex event
4-623	4	227011116	227142769	Misassembly	NONE	227011116	227142769		0					OTHER		complex event
4-624	4	227379917	227586653	Misassembly	NONE	227379917	227586653		0					OTHER		complex event
4-626	4	227854471	227950686	Misassembly	NONE	227854471	227950686		0					OTHER		complex event
4-629	4	231287375	231397754	Misassembly	NONE	231287375	231397754		0					OTHER		complex event
4-630	4	231557236	231681499	Misassembly	NONE	231557236	231681499		0					OTHER		complex event
4-631	4	231833733	231897826	Misassembly	NONE	231833733	231897826		0					OTHER		complex event
4-632	4	232040034	232095150	Misassembly	NONE	232040034	232095150		0					OTHER		complex event
4-633	4	232375717	232468077	Misassembly	NONE	232375717	232468077		0					OTHER		complex event
4-634	4	232510763	232658486	Misassembly	NONE	232510763	232658486		0					OTHER		complex event
4-638	4	233440415	233562996	Misassembly	NONE	233440415	233562996		0					OTHER		complex event
4-639	4	233599099	233707817	Misassembly	NONE	233599099	233707817		0					OTHER		complex event
4-640	4	233821577	233932570	Misassembly	NONE	233821577	233932570		0					OTHER		complex event
4-644	4	234416799	234466355	Misassembly	NONE	234416799	234466355		0					OTHER		complex event
4-645	4	234501070	234699982	Misassembly	NONE	234501070	234699982		0					OTHER		complex event
4-647	4	235053066	235306394	Misassembly	NONE	235053066	235306394		0					OTHER		complex event
4-648	4	235342184	235410855	Misassembly	NONE	235342184	235410855		0					OTHER		complex event
4-650	4	235618226	235677562	Misassembly	NONE	235618226	235677562		0					OTHER		complex event
4-651	4	235677562	235829618	Misassembly	NONE	235677562	235829618		0					OTHER		complex event
4-653	4	236853687	237043051	Misassembly	NONE	236853687	237043051		0					OTHER		complex event
4-654	4	237318931	237576942	Misassembly	NONE	237318931	237576942		0					OTHER		complex event
4-655	4	237795270	237902410	Misassembly	NONE	237795270	237902410		0					OTHER		complex event
4-657	4	238751597	238796651	Misassembly	NONE	238751597	238796651		0					OTHER		complex event
4-658	4	239217899	239411325	Misassembly	NONE	239217899	239411325		0					OTHER		complex event
4-659	4	239538265	239711539	Misassembly	NONE	239538265	239711539		0					OTHER		complex event
4-660	4	239780930	239905132	Misassembly	NONE	239780930	239905132		0					OTHER		complex event
4-662	4	242569386	242651369	Misassembly	NONE	242569386	242651369		0					OTHER		complex event
4-664	4	243071385	243097083	Misassembly	NONE	243071385	243097083		0					OTHER		complex event
4-665	4	243346228	243368769	Misassembly	NONE	243346228	243368769		0					OTHER		complex event
4-666	4	243473677	243700733	Misassembly	NONE	243473677	243700733		0					OTHER		complex event
4-669	4	244268079	244369844	Misassembly	NONE	244268079	244369844		0					OTHER		complex event
4-670	4	244446003	244484709	Misassembly	NONE	244446003	244484709		0					OTHER		complex event
4-671	4	244565862	244890781	Misassembly	NONE	244565862	244890781		0					OTHER		complex event
4-672	4	244970560	245020425	Misassembly	NONE	244970560	245020425		0					OTHER		complex event
5-1	5	675017	750713	Misassembly	NONE	675017	750713		0					OTHER		complex event
5-2	5	817865	873794	Misassembly	NONE	817865	873794		0					OTHER		complex event
5-3	5	990299	1432877	Misassembly	NONE	990299	1432877		0					OTHER		complex event
5-5	5	1628737	1702020	Misassembly	NONE	1628737	1702020		0					OTHER		complex event
5-7	5	1917066	2086918	Misassembly	NONE	1917066	2086918		0					OTHER		complex event
5-9	5	2404985	2472226	Misassembly	NONE	2404985	2472226		0					OTHER		complex event
5-10	5	2628193	2733466	Misassembly	NONE	2628193	2733466		0					OTHER		complex event
5-11	5	2833386	2940293	Misassembly	NONE	2833386	2940293		0					OTHER		complex event
5-12	5	3005457	3034495	Misassembly	NONE	3005457	3034495		0					OTHER		complex event - possible inversion
5-13	5	3062763	3154273	Misassembly	NONE	3062763	3154273		0					OTHER		complex event
5-14	5	3440951	3645534	Misassembly	NONE	3440951	3645534		0					OTHER		complex event

5-15	5	3798596	3914267	Misassembly	NONE	3798596	3914267		0					OTHER		complex event
5-17	5	4366555	4390911	Misassembly	NONE	4366555	4390911		0					OTHER		complex event
5-18	5	4466582	4562364	Misassembly	NONE	4466582	4562364		0					OTHER		complex event - part 1 of 2
5-19	5	4562364	4678814	Misassembly	NONE	4562364	4678814		0					OTHER		complex event - part 2 of 2
5-23	5	5241344	5306379	Misassembly	NONE	5241344	5306379		0					OTHER		complex event - possible inversion
5-26	5	5894810	6136813	Misassembly	NONE	5894810	6136813		0					OTHER		complex event
5-28	5	7129593	7285709	Misassembly	NONE	7129593	7285709		0					OTHER		complex event
5-29	5	7858118	7932627	Misassembly	NONE	7858118	7932627		0					OTHER		complex event
5-31	5	8187323	8314120	Misassembly	NONE	8187323	8314120		0					OTHER		complex event
5-32	5	8453132	8611152	Misassembly	NONE	8453132	8611152		0					OTHER		complex event - possible inversion
5-33	5	8611152	8740503	Misassembly	NONE	8611152	8740503		0					OTHER		complex event
5-34	5	8797406	8850597	Misassembly	NONE	8797406	8850597		0					OTHER		complex event
5-35	5	9047202	9097523	Misassembly	NONE	9047202	9097523		0					OTHER		complex event
5-37	5	9288582	9474780	Misassembly	NONE	9288582	9474780		0					OTHER		complex event
5-39	5	9935572	10009819	Misassembly	NONE	9935572	10009819		0					OTHER		complex event
5-41	5	10258558	10405656	Misassembly	NONE	10258558	10405656		0					OTHER		complex event
5-42	5	10489852	10748239	Misassembly	NONE	10489852	10748239		0					OTHER		complex event
5-44	5	11181371	11683914	Misassembly	NONE	11181371	11683914		0					OTHER		complex event
5-45	5	11995509	12140700	Misassembly	NONE	11995509	12140700		0					OTHER		complex event
5-46	5	12252182	12589515	Misassembly	NONE	12252182	12589515		0					OTHER		complex event
5-47	5	12819623	12941486	Misassembly	NONE	12819623	12941486		0					OTHER		complex event
5-48	5	13048592	13281128	Misassembly	NONE	13048592	13281128		0					OTHER		complex event
5-50	5	16845892	16932198	Misassembly	NONE	16845892	16932198		0					OTHER		complex event
5-51	5	18057346	18112741	Misassembly	NONE	18057346	18112741		0					OTHER		complex event
5-53	5	18714847	18840340	Misassembly	NONE	18714847	18840340		0					OTHER		complex event
5-54	5	19163197	19304523	Misassembly	NONE	19163197	19304523		0					OTHER		complex event
5-55	5	19640408	19697063	Misassembly	NONE	19640408	19697063		0					OTHER		complex event - possible inversion
5-57	5	23486879	23646704	Misassembly	NONE	23486879	23646704		0					OTHER		complex event
5-58	5	25685666	25820070	Misassembly	NONE	25685666	25820070		0					OTHER		complex event
5-59	5	25893142	25936379	Misassembly	NONE	25893142	25936379		0					OTHER		complex event
5-60	5	29124522	29211561	Misassembly	NONE	29124522	29211561		0					OTHER		complex event
5-61	5	30216673	30317764	Misassembly	NONE	30216673	30317764		0					OTHER		complex event
5-62	5	30464929	30550161	Misassembly	NONE	30464929	30550161		0					OTHER		complex event
5-64	5	31239702	31470356	Misassembly	NONE	31239702	31470356		0					OTHER		complex event
5-65	5	31533114	31792021	Misassembly	NONE	31533114	31792021		0					OTHER		complex event
5-66	5	31851812	31940749	Misassembly	NONE	31851812	31940749		0					OTHER		complex event
5-67	5	32009511	32173180	Misassembly	NONE	32009511	32173180		0					OTHER		complex event
5-68	5	32226146	32370711	Misassembly	NONE	32226146	32370711		0					OTHER		complex event
5-70	5	32628007	32718375	Misassembly	NONE	32628007	32718375		0					OTHER		complex event - possible inversion
5-71	5	32768071	33202356	Misassembly	NONE	32768071	33202356		0					OTHER		complex event
5-73	5	33856761	33956125	Misassembly	NONE	33856761	33956125		0					OTHER		complex event
5-74	5	34015560	34078326	Misassembly	NONE	34015560	34078326		0					OTHER		complex event
5-77	5	34391813	34403336	Misassembly	NONE	34391813	34403336		0					OTHER		complex event
5-78	5	34436496	34641654	Misassembly	NONE	34436496	34641654		0					OTHER		complex event
5-79	5	34971623	35017738	Misassembly	NONE	34971623	35017738		0					OTHER		complex event
5-80	5	35094991	35228282	Misassembly	NONE	35094991	35228282		0					OTHER		complex event
5-81	5	35592421	35718972	Misassembly	NONE	35592421	35718972		0					OTHER		complex event
5-83	5	35973312	36056159	Misassembly	NONE	35973312	36056159		0					OTHER		complex event
5-84	5	36624830	36691459	Misassembly	NONE	36624830	36691459		0					OTHER		complex event
5-85	5	36793888	37032542	Misassembly	NONE	36793888	37032542		0					OTHER		complex event
5-86	5	37279512	37464457	Misassembly	NONE	37279512	37464457		0					OTHER		complex event
5-87	5	37657014	37872290	Misassembly	NONE	37657014	37872290		0					OTHER		complex event
5-90	5	38820932	38931579	Misassembly	NONE	38820932	38931579		0					OTHER		complex event
5-91	5	39132039	39170900	Misassembly	NONE	39132039	39170900		0					OTHER		complex event
5-93	5	39749757	39892095	Misassembly	NONE	39749757	39892095		0					OTHER		complex event
5-96	5	40482844	40599362	Misassembly	NONE	40482844	40599362		0					OTHER		complex event
5-97	5	40928608	40981033	Misassembly	NONE	40928608	40981033		0					OTHER		complex event
5-98	5	41492590	41659256	Misassembly	NONE	41492590	41659256		0					OTHER		complex event
5-99	5	41959918	42021050	Misassembly	NONE	41959918	42021050		0					OTHER		complex event
5-101	5	42553472	42807028	Misassembly	NONE	42553472	42807028		0					OTHER		complex event
5-102	5	43389953	43416279	Misassembly	NONE	43389953	43416279		0					OTHER		complex event - possible inversion
5-106	5	44855518	44888513	Misassembly	NONE	44855518	44888513		0					OTHER		complex event - possible inversion
5-109	5	45891646	46110445	Misassembly	NONE	45891646	46110445		0					OTHER		complex event
5-111	5	47978642	48097422	Misassembly	NONE	47978642	48097422		0					OTHER		complex event
5-113	5	48429842	48550078	Misassembly	NONE	48429842	48550078		0					OTHER		complex event
5-114	5	48735173	48829074	Misassembly	NONE	48735173	48829074		0					OTHER		complex event
5-115	5	48876937	49135774	Misassembly	NONE	48876937	49135774		0					OTHER		complex event
5-116	5	49247883	49327168	Misassembly	NONE	49247883	49327168		0					OTHER		complex event
5-117	5	49585506	49838908	Misassembly	NONE	49585506	49838908		0					OTHER		complex event
5-118	5	50189074	50566613	Misassembly	NONE	50189074	50566613		0					OTHER		complex event
5-119	5	50636963	50652384	Misassembly	NONE	50636963	50652384		0					OTHER		complex event
5-120	5	50888147	51173543	Misassembly	NONE	50888147	51173543		0					OTHER		complex event
5-122	5	51498573	51528966	Misassembly	NONE	51498573	51528966		0					OTHER		complex event
5-123	5	51617923	51695156	Misassembly	NONE	51617923	51695156		0					OTHER		complex event
5-125	5	52142377	52274350	Misassembly	NONE	52142377	52274350		0					OTHER		complex event
5-127	5	52575373	52775755	Misassembly	NONE	52575373	52775755		0					OTHER		complex event
5-128	5	53085231	53505613	Misassembly	NONE	53085231	53505613		0					OTHER		complex event

5-129	5	53661035	53933450	Misassembly	NONE	53661035	53933450		0					OTHER		complex event
5-130	5	53996326	54105564	Misassembly	NONE	53996326	54105564		0					OTHER		complex event
5-131	5	54555455	54641120	Misassembly	NONE	54555455	54641120		0					OTHER		complex event
5-132	5	54841463	55033348	Misassembly	NONE	54841463	55033348		0					OTHER		complex event
5-134	5	55142850	55463966	Misassembly	NONE	55142850	55463966		0					OTHER		complex event
5-135	5	55629023	55717449	Misassembly	NONE	55629023	55717449		0					OTHER		complex event
5-137	5	55779595	55915644	Misassembly	NONE	55779595	55915644		0					OTHER		complex event - possible inversion
5-139	5	56318571	56484997	Misassembly	NONE	56318571	56484997		0					OTHER		complex event
5-140	5	56561299	56671007	Misassembly	NONE	56561299	56671007		0					OTHER		complex event
5-142	5	56746206	56840869	Misassembly	NONE	56746206	56840869		0					OTHER		complex event
5-143	5	56884588	56964125	Misassembly	NONE	56884588	56964125		0					OTHER		complex event
5-148	5	57711377	57745743	Misassembly	NONE	57711377	57745743		0					OTHER		complex event - possible inversion
5-149	5	57858075	58139901	Misassembly	NONE	57858075	58139901		0					OTHER		complex event
5-150	5	58446811	58554150	Misassembly	NONE	58446811	58554150		0					OTHER		complex event
5-151	5	58903963	59212170	Misassembly	NONE	58903963	59212170		0					OTHER		complex event
5-152	5	59687504	60000646	Misassembly	NONE	59687504	60000646		0					OTHER		complex event
5-157	5	64612523	64701380	Misassembly	NONE	64612523	64701380		0					OTHER		complex event
5-165	5	67086935	67176408	Misassembly	NONE	67086935	67176408		0					OTHER		complex event
5-166	5	67215545	67314361	Misassembly	NONE	67215545	67314361		0					OTHER		complex event
5-168	5	69509864	69607770	Misassembly	NONE	69509864	69607770		0					OTHER		complex event
5-169	5	69847503	69930808	Misassembly	NONE	69847503	69930808		0					OTHER		complex event
5-170	5	70096215	70238305	Misassembly	NONE	70096215	70238305		0					OTHER		complex event
5-171	5	70277246	70429234	Misassembly	NONE	70277246	70429234		0					OTHER		complex event
5-172	5	70528231	70591285	Misassembly	NONE	70528231	70591285		0					OTHER		complex event
5-173	5	70813271	71182609	Misassembly	NONE	70813271	71182609		0					OTHER		complex event
5-174	5	71247779	71339671	Misassembly	NONE	71247779	71339671		0					OTHER		complex event
5-175	5	71530756	71825517	Misassembly	NONE	71530756	71825517		0					OTHER		complex event
5-176	5	71995612	72194579	Misassembly	NONE	71995612	72194579		0					OTHER		complex event
5-177	5	72241196	72286190	Misassembly	NONE	72241196	72286190		0					OTHER		complex event
5-181	5	72823402	73130792	Misassembly	NONE	72823402	73130792		0					OTHER		complex event
5-182	5	73431175	73600499	Misassembly	NONE	73431175	73600499		0					OTHER		complex event
5-183	5	73717928	73770010	Misassembly	NONE	73717928	73770010		0					OTHER		complex event
5-184	5	73821045	73880610	Misassembly	NONE	73821045	73880610		0					OTHER		complex event
5-189	5	74574264	74659523	Misassembly	NONE	74574264	74659523		0					OTHER		complex event
5-190	5	74721646	74999133	Misassembly	NONE	74721646	74999133		0					OTHER		complex event
5-191	5	75120471	75171814	Misassembly	NONE	75120471	75171814		0					OTHER		complex event
5-192	5	75440379	75688825	Misassembly	NONE	75440379	75688825		0					OTHER		complex event
5-194	5	75854346	75942265	Misassembly	NONE	75854346	75942265		0					OTHER		complex event
5-196	5	76081333	76184747	Misassembly	NONE	76081333	76184747		0					OTHER		complex event
5-197	5	76287716	76384780	Misassembly	NONE	76287716	76384780		0					OTHER		complex event
5-198	5	76444966	76581654	Misassembly	NONE	76444966	76581654		0					OTHER		complex event
5-199	5	76742051	76826065	Misassembly	NONE	76742051	76826065		0					OTHER		complex event
5-200	5	76959629	77144333	Misassembly	NONE	76959629	77144333		0					OTHER		complex event
5-201	5	77348848	77688144	Misassembly	NONE	77348848	77688144		0					OTHER		complex event
5-203	5	78264708	78480448	Misassembly	NONE	78264708	78480448		0					OTHER		complex event
5-204	5	78643596	78707838	Misassembly	NONE	78643596	78707838		0					OTHER		complex event
5-205	5	78945354	79095498	Misassembly	NONE	78945354	79095498		0					OTHER		complex event
5-207	5	80645119	80799556	Misassembly	NONE	80645119	80799556		0					OTHER		complex event
5-208	5	80829870	80970664	Misassembly	NONE	80829870	80970664		0					OTHER		complex event
5-210	5	83546901	83770556	Misassembly	NONE	83546901	83770556		0					OTHER		complex event
5-211	5	83829407	83909554	Misassembly	NONE	83829407	83909554		0					OTHER		complex event
5-213	5	84255970	84432838	Misassembly	NONE	84255970	84432838		0					OTHER		complex event
5-214	5	84558824	84610781	Misassembly	NONE	84558824	84610781		0					OTHER		complex event - possible inversion
5-216	5	85214334	85537488	Misassembly	NONE	85214334	85537488		0					OTHER		complex event
5-217	5	85796523	85994271	Misassembly	NONE	85796523	85994271		0					OTHER		complex event
5-218	5	86158149	86199936	Misassembly	NONE	86158149	86199936		0					OTHER		complex event
5-219	5	86310757	86389903	Misassembly	NONE	86310757	86389903		0					OTHER		complex event
5-220	5	86560045	86619980	Misassembly	NONE	86560045	86619980		0					OTHER		complex event
5-221	5	86686634	86745082	Misassembly	NONE	86686634	86745082		0					OTHER		complex event
5-222	5	86892108	87025277	Misassembly	NONE	86892108	87025277		0					OTHER		complex event
5-223	5	87198450	87295798	Misassembly	NONE	87198450	87295798		0					OTHER		complex event
5-224	5	87385391	87631261	Misassembly	NONE	87385391	87631261		0					OTHER		complex event
5-225	5	87698174	87815470	Misassembly	NONE	87698174	87815470		0					OTHER		complex event
5-226	5	87864104	87926079	Misassembly	NONE	87864104	87926079		0					OTHER		complex event - possible inversion
5-227	5	88048144	88158351	Misassembly	NONE	88048144	88158351		0					OTHER		complex event
5-229	5	88552092	88753068	Misassembly	NONE	88552092	88753068		0					OTHER		complex event
5-231	5	89021027	89284128	Misassembly	NONE	89021027	89284128		0					OTHER		complex event
5-232	5	89473483	89523535	Misassembly	NONE	89473483	89523535		0					OTHER		complex event
5-233	5	89670736	89861586	Misassembly	NONE	89670736	89861586		0					OTHER		complex event
5-234	5	90308334	90411669	Misassembly	NONE	90308334	90411669		0					OTHER		complex event
5-235	5	90545396	90619224	Misassembly	NONE	90545396	90619224		0					OTHER		complex event
5-236	5	90663976	91056310	Misassembly	NONE	90663976	91056310		0					OTHER		complex event
5-242	5	92486493	92572731	Misassembly	NONE	92486493	92572731		0					OTHER		complex event
5-243	5	92791509	92845196	Misassembly	NONE	92791509	92845196		0					OTHER		complex event
5-245	5	94048789	94203788	Misassembly	NONE	94048789	94203788		0					OTHER		complex event
5-246	5	94453640	94638979	Misassembly	NONE	94453640	94638979		0					OTHER		complex event
5-248	5	95298389	95459927	Misassembly	NONE	95298389	95459927		0					OTHER		complex event
5-249	5	95592797	95843266	Misassembly	NONE	95592797	95843266		0					OTHER		complex event
5-250	5	96010746	96177376	Misassembly	NONE	96010746	96177376		0					OTHER		complex event
5-251	5	96319671	96563869	Misassembly	NONE	96319671	96563869		0					OTHER		complex event
5-252	5	96835463	96891661	Misassembly	NONE	96835463	96891661		0					OTHER		complex event
5-254	5	97113340	97386708	Misassembly	NONE	97113340	97386708		0					OTHER		complex event
5-255	5	97600227	97689392	Misassembly	NONE	97600227	97689392		0					OTHER		complex event

5-258	5	98033645	98092413	Misassembly	NONE	98033645	98092413		0					OTHER		complex event
5-259	5	98215471	98366038	Misassembly	NONE	98215471	98366038		0					OTHER		complex event
5-261	5	98668819	98791450	Misassembly	NONE	98668819	98791450		0					OTHER		complex event
5-262	5	99274136	99307968	Misassembly	NONE	99274136	99307968		0					OTHER		complex event
5-263	5	99384495	99625602	Misassembly	NONE	99384495	99625602		0					OTHER		complex event
5-264	5	99760886	99803447	Misassembly	NONE	99760886	99803447		0					OTHER		complex event
5-266	5	100310237	100394292	Misassembly	NONE	100310237	100394292		0					OTHER		complex event
5-267	5	100731461	100892472	Misassembly	NONE	100731461	100892472		0					OTHER		complex event
5-269	5	104173927	104255758	Misassembly	NONE	104173927	104255758		0					OTHER		complex event
5-273	5	106067593	106428958	Misassembly	NONE	106067593	106428958		0					OTHER		complex event
5-274	5	107173955	107294567	Misassembly	NONE	107173955	107294567		0					OTHER		complex event
5-275	5	107435703	107499362	Misassembly	NONE	107435703	107499362		0					OTHER		complex event
5-276	5	107550674	107632905	Misassembly	NONE	107550674	107632905		0					OTHER		complex event
5-277	5	107660708	107896844	Misassembly	NONE	107660708	107896844		0					OTHER		complex event
5-278	5	107941435	107990028	Misassembly	NONE	107941435	107990028		0					OTHER		complex event
5-279	5	108037014	108115660	Misassembly	NONE	108037014	108115660		0					OTHER		complex event
5-280	5	108263014	108492241	Misassembly	NONE	108263014	108492241		0					OTHER		complex event
5-281	5	108644661	108695385	Misassembly	NONE	108644661	108695385		0					OTHER		complex event
5-282	5	108753991	108825083	Misassembly	NONE	108753991	108825083		0					OTHER		complex event
5-284	5	108952143	109048723	Misassembly	NONE	108952143	109048723		0					OTHER		complex event
5-285	5	109272733	109401097	Misassembly	NONE	109272733	109401097		0					OTHER		complex event
5-286	5	109683530	109906411	Misassembly	NONE	109683530	109906411		0					OTHER		complex event
5-287	5	109971524	110062982	Misassembly	NONE	109971524	110062982		0					OTHER		complex event - possible translocation
5-291	5	110803796	111096635	Misassembly	NONE	110803796	111096635		0					OTHER		complex event
5-292	5	111365049	111853963	Misassembly	NONE	111365049	111853963		0					OTHER		complex event
5-293	5	111995220	112405114	Misassembly	NONE	111995220	112405114		0					OTHER		complex event
5-294	5	112443149	112532242	Misassembly	NONE	112443149	112532242		0					OTHER		complex event
5-297	5	114055842	114103321	Misassembly	NONE	114055842	114103321		0					OTHER		complex event
5-300	5	116441539	116632706	Misassembly	NONE	116441539	116632706		0					OTHER		complex event
5-301	5	116786137	116835280	Misassembly	NONE	116786137	116835280		0					OTHER		complex event
5-302	5	117136301	117326719	Misassembly	NONE	117136301	117326719		0					OTHER		complex event
5-304	5	118662410	118709520	Misassembly	NONE	118662410	118709520		0					OTHER		complex event - possible inversion
5-306	5	119514297	119669369	Misassembly	NONE	119514297	119669369		0					OTHER		complex event - possible inversion
5-307	5	120075904	120164004	Misassembly	NONE	120075904	120164004		0					OTHER		complex event
5-309	5	120381322	120475178	Misassembly	NONE	120381322	120475178		0					OTHER		complex event
5-310	5	120606824	120825849	Misassembly	NONE	120606824	120825849		0					OTHER		complex event
5-314	5	121572829	121666784	Misassembly	NONE	121572829	121666784		0					OTHER		complex event
5-317	5	122633060	122791947	Misassembly	NONE	122633060	122791947		0					OTHER		complex event
5-318	5	122933705	123161347	Misassembly	NONE	122933705	123161347		0					OTHER		complex event
5-319	5	123226407	123438093	Misassembly	NONE	123226407	123438093		0					OTHER		complex event
5-321	5	123664300	123803851	Misassembly	NONE	123664300	123803851		0					OTHER		complex event
5-322	5	123921630	123967469	Misassembly	NONE	123921630	123967469		0					OTHER		complex event
5-324	5	124216359	124296706	Misassembly	NONE	124216359	124296706		0					OTHER		complex event
5-326	5	124502148	124590547	Misassembly	NONE	124502148	124590547		0					OTHER		complex event
5-330	5	125341318	125403423	Misassembly	NONE	125341318	125403423		0					OTHER		complex event
5-331	5	125523210	125816524	Misassembly	NONE	125523210	125816524		0					OTHER		complex event
5-332	5	125843978	125975243	Misassembly	NONE	125843978	125975243		0					OTHER		complex event
5-333	5	126216668	126328392	Misassembly	NONE	126216668	126328392		0					OTHER		complex event
5-335	5	126562113	126724214	Misassembly	NONE	126562113	126724214		0					OTHER		complex event
5-336	5	127138703	127212486	Misassembly	NONE	127138703	127212486		0					OTHER		complex event - possible inversion
5-337	5	127333251	127369951	Misassembly	NONE	127333251	127369951		0					OTHER		complex event
5-339	5	130367572	130722014	Misassembly	NONE	130367572	130722014		0					OTHER		complex event
5-341	5	131233836	131335050	Misassembly	NONE	131233836	131335050		0					OTHER		complex event
5-342	5	131495612	131743373	Misassembly	NONE	131495612	131743373		0					OTHER		complex event
5-343	5	131789638	131817354	Misassembly	NONE	131789638	131817354		0					OTHER		complex event - possible inversion
5-344	5	131851428	131912659	Misassembly	NONE	131851428	131912659		0					OTHER		complex event - possible inversion
5-345	5	131954585	131997331	Misassembly	NONE	131954585	131997331		0					OTHER		complex event
5-346	5	132102156	132422878	Misassembly	NONE	132102156	132422878		0					OTHER		complex event
5-349	5	132911431	132945248	Misassembly	NONE	132911431	132945248		0					OTHER		complex event
5-350	5	133177064	133276428	Misassembly	NONE	133177064	133276428		0					OTHER		complex event
5-351	5	133337323	133987315	Misassembly	NONE	133337323	133987315		0					OTHER		complex event
5-352	5	134133664	134276839	Misassembly	NONE	134133664	134276839		0					OTHER		complex event
5-355	5	134569027	134692689	Misassembly	NONE	134569027	134692689		0					OTHER		complex event
5-356	5	134859510	134936273	Misassembly	NONE	134859510	134936273		0					OTHER		complex event
5-358	5	135557036	135615588	Misassembly	NONE	135557036	135615588		0					OTHER		complex event
5-361	5	136071556	136103843	Misassembly	NONE	136071556	136103843		0					OTHER		complex event
5-364	5	136762257	136939357	Misassembly	NONE	136762257	136939357		0					OTHER		complex event
5-365	5	137123283	137233196	Misassembly	NONE	137123283	137233196		0					OTHER		complex event
5-368	5	137672053	137756245	Misassembly	NONE	137672053	137756245		0					OTHER		complex event
5-369	5	137895958	138013380	Misassembly	NONE	137895958	138013380		0					OTHER		complex event
5-372	5	138523837	138682031	Misassembly	NONE	138523837	138682031		0					OTHER		complex event
5-373	5	138798125	138831657	Misassembly	NONE	138798125	138831657		0					OTHER		complex event
5-374	5	138982732	139064767	Misassembly	NONE	138982732	139064767		0					OTHER		complex event
5-375	5	139440751	139533743	Misassembly	NONE	139440751	139533743		0					OTHER		complex event
5-380	5	140949612	141286449	Misassembly	NONE	140949612	141286449		0					OTHER		complex event
5-381	5	141465727	142008575	Misassembly	NONE	141465727	142008575		0					OTHER		complex event
5-382	5	142092908	142118332	Misassembly	NONE	142092908	142118332		0					OTHER		complex event
5-384	5	142866559	142980032	Misassembly	NONE	142866559	142980032		0					OTHER		complex event
5-385	5	143038648	143208156	Misassembly	NONE	143038648	143208156		0					OTHER		complex event

5-386	5	143461197	143534685	Misassembly	NONE	143461197	143534685		0					OTHER		complex event
5-387	5	143585737	143685804	Misassembly	NONE	143585737	143685804		0					OTHER		complex event
5-388	5	144205694	144382931	Misassembly	NONE	144205694	144382931		0					OTHER		complex event
5-390	5	144547651	144740073	Misassembly	NONE	144547651	144740073		0					OTHER		complex event
5-392	5	145778086	145905415	Misassembly	NONE	145778086	145905415		0					OTHER		complex event
5-395	5	146166835	146227725	Misassembly	NONE	146166835	146227725		0					OTHER		complex event
5-396	5	146265281	146324094	Misassembly	NONE	146265281	146324094		0					OTHER		complex event
5-399	5	148903986	149021004	Misassembly	NONE	148903986	149021004		0					OTHER		complex event
5-400	5	149171576	149208200	Misassembly	NONE	149171576	149208200		0					OTHER		complex event - possible insertion
5-401	5	150984707	151161715	Misassembly	NONE	150984707	151161715		0					OTHER		complex event
5-402	5	153033572	153130799	Misassembly	NONE	153033572	153130799		0					OTHER		complex event
5-403	5	153179438	153277598	Misassembly	NONE	153179438	153277598		0					OTHER		complex event
5-404	5	153713732	153803621	Misassembly	NONE	153713732	153803621		0					OTHER		complex event
5-405	5	153887377	153991233	Misassembly	NONE	153887377	153991233		0					OTHER		complex event
5-406	5	154036802	154383838	Misassembly	NONE	154036802	154383838		0					OTHER		complex event
5-407	5	154621573	154714696	Misassembly	NONE	154621573	154714696		0					OTHER		complex event
5-408	5	154932503	155122418	Misassembly	NONE	154932503	155122418		0					OTHER		complex event
5-409	5	155278873	155351834	Misassembly	NONE	155278873	155351834		0					OTHER		complex event
5-410	5	155375787	155532050	Misassembly	NONE	155375787	155532050		0					OTHER		complex event
5-411	5	155765604	156030706	Misassembly	NONE	155765604	156030706		0					OTHER		complex event
5-412	5	156083509	156273986	Misassembly	NONE	156083509	156273986		0					OTHER		complex event
5-413	5	156392147	156600393	Misassembly	NONE	156392147	156600393		0					OTHER		complex event
5-414	5	156767592	156903044	Misassembly	NONE	156767592	156903044		0					OTHER		complex event
5-416	5	157229819	157467720	Misassembly	NONE	157229819	157467720		0					OTHER		complex event
5-418	5	158085264	158250331	Misassembly	NONE	158085264	158250331		0					OTHER		complex event
5-419	5	158589511	158683407	Misassembly	NONE	158589511	158683407		0					OTHER		complex event
5-420	5	159010878	159136761	Misassembly	NONE	159010878	159136761		0					OTHER		complex event
5-421	5	159329910	159662490	Misassembly	NONE	159329910	159662490		0					OTHER		complex event
5-424	5	159964777	160100512	Misassembly	NONE	159964777	160100512		0					OTHER		complex event
5-427	5	162466768	162571595	Misassembly	NONE	162466768	162571595		0					OTHER		complex event
5-429	5	162838177	163035850	Misassembly	NONE	162838177	163035850		0					OTHER		complex event
5-431	5	163591371	163649463	Misassembly	NONE	163591371	163649463		0					OTHER		complex event - possible inversion
5-432	5	163700866	163788294	Misassembly	NONE	163700866	163788294		0					OTHER		complex event
5-433	5	163853307	163912130	Misassembly	NONE	163853307	163912130		0					OTHER		complex event
5-436	5	168253755	168466159	Misassembly	NONE	168253755	168466159		0					OTHER		complex event
5-438	5	168907011	169229945	Misassembly	NONE	168907011	169229945		0					OTHER		complex event
5-439	5	169500744	169625929	Misassembly	NONE	169500744	169625929		0					OTHER		complex event
5-440	5	169724478	169943714	Misassembly	NONE	169724478	169943714		0					OTHER		complex event
5-441	5	170080864	170347044	Misassembly	NONE	170080864	170347044		0					OTHER		complex event
5-443	5	170657656	170901728	Misassembly	NONE	170657656	170901728		0					OTHER		complex event
5-445	5	171224650	171477030	Misassembly	NONE	171224650	171477030		0					OTHER		complex event
5-446	5	171858051	172065621	Misassembly	NONE	171858051	172065621		0					OTHER		complex event
5-447	5	174531198	174649389	Misassembly	NONE	174531198	174649389		0					OTHER		complex event
5-448	5	174735028	174815147	Misassembly	NONE	174735028	174815147		0					OTHER		complex event
5-451	5	180805179	181036791	Misassembly	NONE	180805179	181036791		0					OTHER		complex event
5-452	5	181076655	181153653	Misassembly	NONE	181076655	181153653		0					OTHER		complex event
5-453	5	181250661	181335110	Misassembly	NONE	181250661	181335110		0					OTHER		complex event
5-454	5	181389434	181402956	Misassembly	NONE	181389434	181402956		0					OTHER		complex event - possible inversion
5-457	5	184433692	184556448	Misassembly	NONE	184433692	184556448		0					OTHER		complex event
5-459	5	184752847	184774789	Misassembly	NONE	184752847	184774789		0					OTHER		complex event
5-462	5	185986602	186047454	Misassembly	NONE	185986602	186047454		0					OTHER		complex event
5-463	5	186166707	186262754	Misassembly	NONE	186166707	186262754		0					OTHER		complex event
5-464	5	186389416	186423785	Misassembly	NONE	186389416	186423785		0					OTHER		complex event
5-467	5	186668394	186866718	Misassembly	NONE	186668394	186866718		0					OTHER		complex event
5-469	5	187486239	187799683	Misassembly	NONE	187486239	187799683		0					OTHER		complex event
5-470	5	187838153	187869664	Misassembly	NONE	187838153	187869664		0					OTHER		complex event
5-471	5	187950813	188039245	Misassembly	NONE	187950813	188039245		0					OTHER		complex event
5-472	5	188369037	188601187	Misassembly	NONE	188369037	188601187		0					OTHER		complex event
5-473	5	190317012	190384545	Misassembly	NONE	190317012	190384545		0					OTHER		complex event
5-476	5	195207790	195308755	Misassembly	NONE	195207790	195308755		0					OTHER		complex event
5-478	5	195536954	195669905	Misassembly	NONE	195536954	195669905		0					OTHER		complex event
5-480	5	195986880	196454338	Misassembly	NONE	195986880	196454338		0					OTHER		complex event
5-481	5	197111615	197366278	Misassembly	NONE	197111615	197366278		0					OTHER		complex event
5-483	5	197760297	197808802	Misassembly	NONE	197760297	197808802		0					OTHER		complex event
5-484	5	197841204	197922562	Misassembly	NONE	197841204	197922562		0					OTHER		complex event
5-485	5	198091794	198219106	Misassembly	NONE	198091794	198219106		0					OTHER		complex event
5-486	5	198327239	198526701	Misassembly	NONE	198327239	198526701		0					OTHER		complex event
5-487	5	198570322	198671525	Misassembly	NONE	198570322	198671525		0					OTHER		complex event
5-489	5	198832310	198997728	Misassembly	NONE	198832310	198997728		0					OTHER		complex event
5-491	5	201979064	202053146	Misassembly	NONE	201979064	202053146		0					OTHER		complex event
5-492	5	202382565	202449662	Misassembly	NONE	202382565	202449662		0					OTHER		complex event
5-496	5	212353780	212432549	Misassembly	NONE	212353780	212432549		0					OTHER		complex event
5-497	5	212922401	213179148	Misassembly	NONE	212922401	213179148		0					OTHER		complex event
5-498	5	213346200	213432096	Misassembly	NONE	213346200	213432096		0					OTHER		complex event
5-499	5	213499415	213537654	Misassembly	NONE	213499415	213537654		0					OTHER		complex event
5-500	5	213762818	213880579	Misassembly	NONE	213762818	213880579		0					OTHER		complex event
5-502	5	214087669	214424033	Misassembly	NONE	214087669	214424033		0					OTHER		complex event
5-503	5	214699892	214812438	Misassembly	NONE	214699892	214812438		0					OTHER		complex event
5-504	5	214850565	215004884	Misassembly	NONE	214850565	215004884		0					OTHER		complex event
5-505	5	215259018	215437392	Misassembly	NONE	215259018	215437392		0					OTHER		complex event
5-507	5	215989570	216018414	Misassembly	NONE	215989570	216018414		0					OTHER		complex event - possible inversion
6-5	6	3387374	3493470	Misassembly	NONE	3387374	3493470		0					OTHER		complex event
6-6	6	5713204	5806392	Misassembly	NONE	5713204	5806392		0					OTHER		complex event

6-8	6	9571703	9676613	Misassembly	NONE	9571703	9676613		0					OTHER		complex event
6-10	6	11930273	11958290	Misassembly	NONE	11930273	11958290		0					OTHER		complex event
6-12	6	12415833	12542339	Misassembly	NONE	12415833	12542339		0					OTHER		complex event
6-14	6	18915172	19029728	Misassembly	NONE	18915172	19029728		0					OTHER		complex event
6-15	6	19249093	19279997	Misassembly	NONE	19249093	19279997		0					OTHER		complex event - possible inversion
6-18	6	19829880	19972380	Misassembly	NONE	19829880	19972380		0					OTHER		complex event
6-20	6	20401458	20479664	Misassembly	NONE	20401458	20479664		0					OTHER		complex event
6-21	6	20543396	20715621	Misassembly	NONE	20543396	20715621		0					OTHER		complex event
6-24	6	21985073	22120717	Misassembly	NONE	21985073	22120717		0					OTHER		complex event
6-26	6	29694726	29886792	Misassembly	NONE	29694726	29886792		0					OTHER		complex event
6-27	6	30872256	31224465	Misassembly	NONE	30872256	31224465		0					OTHER		complex event
6-28	6	31325148	31393267	Misassembly	NONE	31325148	31393267		0					OTHER		complex event
6-29	6	31692447	31761408	Misassembly	NONE	31692447	31761408		0					OTHER		complex event
6-30	6	32108697	32213817	Misassembly	NONE	32108697	32213817		0					OTHER		complex event
6-31	6	32252234	32295606	Misassembly	NONE	32252234	32295606		0					OTHER		complex event
6-32	6	32447571	32634975	Misassembly	NONE	32447571	32634975		0					OTHER		complex event
6-33	6	32906228	33007257	Misassembly	NONE	32906228	33007257		0					OTHER		complex event
6-35	6	33341332	33390155	Misassembly	NONE	33341332	33390155		0					OTHER		complex event
6-36	6	33524960	33644807	Misassembly	NONE	33524960	33644807		0					OTHER		complex event
6-38	6	33821931	33912466	Misassembly	NONE	33821931	33912466		0					OTHER		complex event
6-39	6	34040179	34129685	Misassembly	NONE	34040179	34129685		0					OTHER		complex event
6-40	6	34308671	34358904	Misassembly	NONE	34308671	34358904		0					OTHER		complex event
6-41	6	34431871	34543624	Misassembly	NONE	34431871	34543624		0					OTHER		complex event
6-43	6	34642065	34910716	Misassembly	NONE	34642065	34910716		0					OTHER		complex event
6-44	6	35395616	35531567	Misassembly	NONE	35395616	35531567		0					OTHER		complex event
6-45	6	35848754	36094308	Misassembly	NONE	35848754	36094308		0					OTHER		complex event
6-46	6	36256908	36359903	Misassembly	NONE	36256908	36359903		0					OTHER		complex event
6-49	6	37241635	37405690	Misassembly	NONE	37241635	37405690		0					OTHER		complex event
6-52	6	37754889	37890530	Misassembly	NONE	37754889	37890530		0					OTHER		complex event
6-53	6	38019373	38234043	Misassembly	NONE	38019373	38234043		0					OTHER		complex event
6-54	6	38507375	38573044	Misassembly	NONE	38507375	38573044		0					OTHER		complex event
6-55	6	38671118	38703851	Misassembly	NONE	38671118	38703851		0					OTHER		complex event
6-56	6	38834582	39055707	Misassembly	NONE	38834582	39055707		0					OTHER		complex event
6-57	6	39253926	39287851	Misassembly	NONE	39253926	39287851		0					OTHER		complex event
6-58	6	39623333	39692608	Misassembly	NONE	39623333	39692608		0					OTHER		complex event
6-59	6	39825367	39959278	Misassembly	NONE	39825367	39959278		0					OTHER		complex event
6-60	6	40111860	40145092	Misassembly	NONE	40111860	40145092		0					OTHER		complex event
6-63	6	40670938	40716868	Misassembly	NONE	40670938	40716868		0					OTHER		complex event - possible inversion
6-65	6	41004939	41037575	Misassembly	NONE	41004939	41037575		0					OTHER		complex event
6-66	6	41267998	41389085	Misassembly	NONE	41267998	41389085		0					OTHER		complex event
6-67	6	41744651	41894744	Misassembly	NONE	41744651	41894744		0					OTHER		complex event
6-70	6	46771761	47143181	Misassembly	NONE	46771761	47143181		0					OTHER		complex event
6-71	6	47181882	47307557	Misassembly	NONE	47181882	47307557		0					OTHER		complex event
6-73	6	50575242	50602408	Misassembly	NONE	50575242	50602408		0					OTHER		complex event
6-74	6	50784897	50853464	Misassembly	NONE	50784897	50853464		0					OTHER		complex event
6-75	6	51210641	51283273	Misassembly	NONE	51210641	51283273		0					OTHER		complex event
6-77	6	51708681	51939301	Misassembly	NONE	51708681	51939301		0					OTHER		complex event
6-79	6	52341161	52544971	Misassembly	NONE	52341161	52544971		0					OTHER		complex event
6-81	6	52791831	52848605	Misassembly	NONE	52791831	52848605		0					OTHER		complex event
6-83	6	53074418	53233057	Misassembly	NONE	53074418	53233057		0					OTHER		complex event
6-85	6	53461217	53726734	Misassembly	NONE	53461217	53726734		0					OTHER		complex event
6-86	6	53893341	54114101	Misassembly	NONE	53893341	54114101		0					OTHER		complex event
6-87	6	54203941	54276029	Misassembly	NONE	54203941	54276029		0					OTHER		complex event
6-88	6	54406918	54622953	Misassembly	NONE	54406918	54622953		0					OTHER		complex event
6-89	6	54798793	54856141	Misassembly	NONE	54798793	54856141		0					OTHER		complex event
6-92	6	55225917	55401240	Misassembly	NONE	55225917	55401240		0					OTHER		complex event
6-93	6	55440596	55781577	Misassembly	NONE	55440596	55781577		0					OTHER		complex event
6-100	6	56950473	57025309	Misassembly	NONE	56950473	57025309		0					OTHER		complex event
6-101	6	57162151	57248916	Misassembly	NONE	57162151	57248916		0					OTHER		complex event
6-102	6	57581973	57825625	Misassembly	NONE	57581973	57825625		0					OTHER		complex event
6-104	6	60722895	60838678	Misassembly	NONE	60722895	60838678		0					OTHER		complex event
6-105	6	60921960	61059481	Misassembly	NONE	60921960	61059481		0					OTHER		complex event
6-107	6	61390413	61478137	Misassembly	NONE	61390413	61478137		0					OTHER		complex event
6-108	6	61527010	61619067	Misassembly	NONE	61527010	61619067		0					OTHER		complex event
6-110	6	61952283	62038510	Misassembly	NONE	61952283	62038510		0					OTHER		complex event
6-111	6	62354496	62438625	Misassembly	NONE	62354496	62438625		0					OTHER		complex event
6-113	6	62593368	62666740	Misassembly	NONE	62593368	62666740		0					OTHER		complex event
6-114	6	62951200	63124022	Misassembly	NONE	62951200	63124022		0					OTHER		complex event
6-115	6	64002884	64114660	Misassembly	NONE	64002884	64114660		0					OTHER		complex event - possible inversion within
6-116	6	64170084	64184217	Misassembly	NONE	64170084	64184217		0					OTHER		complex event - possible inversion
6-117	6	64418762	64449550	Misassembly	NONE	64418762	64449550		0					OTHER		complex event
6-120	6	64934432	65157108	Misassembly	NONE	64934432	65157108		0					OTHER		complex event
6-121	6	65187074	65222437	Misassembly	NONE	65187074	65222437		0					OTHER		complex event
6-122	6	65333453	65346039	Misassembly	NONE	65333453	65346039		0					OTHER		complex event
6-124	6	65584303	65727395	Misassembly	NONE	65584303	65727395		0					OTHER		complex event
6-125	6	65911268	65947223	Misassembly	NONE	65911268	65947223		0					OTHER		complex event
6-126	6	66126755	66214859	Misassembly	NONE	66126755	66214859		0					OTHER		complex event
6-128	6	66445001	66668003	Misassembly	NONE	66445001	66668003		0					OTHER		complex event
6-129	6	66717889	66746258	Misassembly	NONE	66717889	66746258		0					OTHER		complex event
6-132	6	67551988	67638372	Misassembly	NONE	67551988	67638372		0					OTHER		complex event
6-139	6	70498872	70546320	Misassembly	NONE	70498872	70546320		0					OTHER		complex event

6-143	6	74397839	74444318	Misassembly	NONE	74397839	74444318		0					OTHER		complex event - possible inversion
6-144	6	74477428	74612253	Misassembly	NONE	74477428	74612253		0					OTHER		complex event
6-145	6	74824837	75031884	Misassembly	NONE	74824837	75031884		0					OTHER		complex event
6-146	6	75061866	75509541	Misassembly	NONE	75061866	75509541		0					OTHER		complex event
6-147	6	75700006	75832402	Misassembly	NONE	75700006	75832402		0					OTHER		complex event
6-148	6	76017041	76125878	Misassembly	NONE	76017041	76125878		0					OTHER		complex event
6-149	6	76411944	76534813	Misassembly	NONE	76411944	76534813		0					OTHER		complex event
6-151	6	77342950	77515710	Misassembly	NONE	77342950	77515710		0					OTHER		complex event
6-152	6	77893309	77994640	Misassembly	NONE	77893309	77994640		0					OTHER		complex event
6-153	6	78022895	78201066	Misassembly	NONE	78022895	78201066		0					OTHER		complex event
6-155	6	78402800	78549950	Misassembly	NONE	78402800	78549950		0					OTHER		complex event
6-156	6	78682405	78803707	Misassembly	NONE	78682405	78803707		0					OTHER		complex event
6-158	6	79060829	79193000	Misassembly	NONE	79060829	79193000		0					OTHER		complex event
6-161	6	79965960	80057658	Misassembly	NONE	79965960	80057658		0					OTHER		complex event
6-162	6	80295734	80373055	Misassembly	NONE	80295734	80373055		0					OTHER		complex event
6-163	6	80763939	80966632	Misassembly	NONE	80763939	80966632		0					OTHER		complex event
6-164	6	81292871	81608042	Misassembly	NONE	81292871	81608042		0					OTHER		complex event
6-165	6	83322451	83381448	Misassembly	NONE	83322451	83381448		0					OTHER		complex event
6-167	6	88617285	88715573	Misassembly	NONE	88617285	88715573		0					OTHER		complex event
6-171	6	92751976	92793000	Misassembly	NONE	92751976	92793000		0					OTHER		complex event
6-172	6	97389201	97674995	Misassembly	NONE	97389201	97674995		0					OTHER		complex event
6-174	6	138527094	138598576	Misassembly	NONE	138527094	138598576		0					OTHER		complex event
7-1	7	1411593	1514361	Misassembly	NONE	1411593	1514361		0					OTHER		complex event
7-5	7	6341973	6444024	Misassembly	NONE	6341973	6444024		0					OTHER		complex event
7-8	7	6870804	6929713	Misassembly	NONE	6870804	6929713		0					OTHER		complex event - possible inversion
7-9	7	7104285	7153350	Misassembly	NONE	7104285	7153350		0					OTHER		complex event
7-11	7	7267662	7342490	Misassembly	NONE	7267662	7342490		0					OTHER		complex event
7-12	7	7407470	7557689	Misassembly	NONE	7407470	7557689		0					OTHER		complex event
7-13	7	7621466	7796058	Misassembly	NONE	7621466	7796058		0					OTHER		complex event
7-15	7	7880710	7992690	Misassembly	NONE	7880710	7992690		0					OTHER		complex event
7-16	7	8199108	8540794	Misassembly	NONE	8199108	8540794		0					OTHER		complex event
7-19	7	8890849	9060553	Misassembly	NONE	8890849	9060553		0					OTHER		complex event
7-21	7	9692677	9789579	Misassembly	NONE	9692677	9789579		0					OTHER		complex event - possible inversion
7-23	7	10078708	10282718	Misassembly	NONE	10078708	10282718		0					OTHER		complex event
7-29	7	15431515	15697592	Misassembly	NONE	15431515	15697592		0					OTHER		complex event
7-31	7	16119669	16220901	Misassembly	NONE	16119669	16220901		0					OTHER		complex event
7-32	7	16817452	17019045	Misassembly	NONE	16817452	17019045		0					OTHER		complex event
7-33	7	17117454	17437884	Misassembly	NONE	17117454	17437884		0					OTHER		complex event
7-34	7	17633132	17910998	Misassembly	NONE	17633132	17910998		0					OTHER		complex event
7-35	7	18098736	18475240	Misassembly	NONE	18098736	18475240		0					OTHER		complex event
7-40	7	23216189	23287500	Misassembly	NONE	23216189	23287500		0					OTHER		complex event
7-42	7	24613296	24675554	Misassembly	NONE	24613296	24675554		0					OTHER		complex event
7-43	7	25930185	26130874	Misassembly	NONE	25930185	26130874		0					OTHER		complex event
7-46	7	28271624	28397718	Misassembly	NONE	28271624	28397718		0					OTHER		complex event
7-50	7	32327012	32434358	Misassembly	NONE	32327012	32434358		0					OTHER		complex event
7-52	7	34525179	34693031	Misassembly	NONE	34525179	34693031		0					OTHER		complex event
7-56	7	36030879	36089748	Misassembly	NONE	36030879	36089748		0					OTHER		complex event - possible inversion
7-57	7	36293140	36478317	Misassembly	NONE	36293140	36478317		0					OTHER		complex event
7-58	7	36646570	36740106	Misassembly	NONE	36646570	36740106		0					OTHER		complex event
7-59	7	37362009	37464118	Misassembly	NONE	37362009	37464118		0					OTHER		complex event - possible inversion
7-60	7	37753664	37960516	Misassembly	NONE	37753664	37960516		0					OTHER		complex event
7-63	7	38521452	38601005	Misassembly	NONE	38521452	38601005		0					OTHER		complex event
7-65	7	38878383	39013828	Misassembly	NONE	38878383	39013828		0					OTHER		complex event
7-66	7	39061059	39159895	Misassembly	NONE	39061059	39159895		0					OTHER		complex event
7-67	7	39212817	39577856	Misassembly	NONE	39212817	39577856		0					OTHER		complex event
7-68	7	39877230	39942693	Misassembly	NONE	39877230	39942693		0					OTHER		complex event
7-72	7	40680207	40748248	Misassembly	NONE	40680207	40748248		0					OTHER		complex event
7-73	7	40869248	40970312	Misassembly	NONE	40869248	40970312		0					OTHER		complex event
7-74	7	41113896	41350012	Misassembly	NONE	41113896	41350012		0					OTHER		complex event
7-76	7	42129627	42198612	Misassembly	NONE	42129627	42198612		0					OTHER		complex event
7-77	7	42394619	42535561	Misassembly	NONE	42394619	42535561		0					OTHER		complex event
7-78	7	44464871	44533406	Misassembly	NONE	44464871	44533406		0					OTHER		complex event
7-83	7	45075959	45256468	Misassembly	NONE	45075959	45256468		0					OTHER		complex event
7-84	7	45312447	45387592	Misassembly	NONE	45312447	45387592		0					OTHER		complex event
7-85	7	45677984	46060428	Misassembly	NONE	45677984	46060428		0					OTHER		complex event
7-86	7	46383946	46422143	Misassembly	NONE	46383946	46422143		0					OTHER		complex event
7-87	7	47028885	47145236	Misassembly	NONE	47028885	47145236		0					OTHER		complex event
7-88	7	47189605	47425255	Misassembly	NONE	47189605	47425255		0					OTHER		complex event
7-89	7	47652538	47692042	Misassembly	NONE	47652538	47692042		0					OTHER		complex event
7-91	7	47878866	47946946	Misassembly	NONE	47878866	47946946		0					OTHER		complex event
7-93	7	48158196	48187789	Misassembly	NONE	48158196	48187789		0					OTHER		complex event
7-95	7	48295883	48520640	Misassembly	NONE	48295883	48520640		0					OTHER		complex event
7-98	7	48858876	48887773	Misassembly	NONE	48858876	48887773		0					OTHER		complex event
7-100	7	49675116	49774335	Misassembly	NONE	49675116	49774335		0					OTHER		complex event
7-101	7	49843127	50091914	Misassembly	NONE	49843127	50091914		0					OTHER		complex event
7-103	7	50249976	50553867	Misassembly	NONE	50249976	50553867		0					OTHER		complex event
7-105	7	51074697	51244293	Misassembly	NONE	51074697	51244293		0					OTHER		complex event
7-107	7	54101531	54162035	Misassembly	NONE	54101531	54162035		0					OTHER		complex event
7-108	7	54325225	54451306	Misassembly	NONE	54325225	54451306		0					OTHER		complex event
7-109	7	54797098	55053087	Misassembly	NONE	54797098	55053087		0					OTHER		complex event
7-117	7	59691766	59762948	Misassembly	NONE	59691766	59762948		0					OTHER		complex event

7-120	7	60316911	60442049	Misassembly	NONE	60316911	60442049		0					OTHER		complex event
7-121	7	60594259	60682552	Misassembly	NONE	60594259	60682552		0					OTHER		complex event
7-122	7	61025830	61103924	Misassembly	NONE	61025830	61103924		0					OTHER		complex event
7-123	7	61377464	61699254	Misassembly	NONE	61377464	61699254		0					OTHER		complex event
7-127	7	62568383	62604383	Misassembly	NONE	62568383	62604383		0					OTHER		complex event
7-128	7	62891750	63063372	Misassembly	NONE	62891750	63063372		0					OTHER		complex event
7-130	7	63312723	63396933	Misassembly	NONE	63312723	63396933		0					OTHER		complex event
7-131	7	63951913	63979529	Misassembly	NONE	63951913	63979529		0					OTHER		complex event
7-132	7	64069081	64148354	Misassembly	NONE	64069081	64148354		0					OTHER		complex event
7-134	7	64268107	64382821	Misassembly	NONE	64268107	64382821		0					OTHER		complex event
7-135	7	64474532	64639669	Misassembly	NONE	64474532	64639669		0					OTHER		complex event
7-136	7	64841533	64972315	Misassembly	NONE	64841533	64972315		0					OTHER		complex event
7-137	7	65529461	65587101	Misassembly	NONE	65529461	65587101		0					OTHER		complex event
7-138	7	65822613	66021564	Misassembly	NONE	65822613	66021564		0					OTHER		complex event
7-142	7	66751831	66938587	Misassembly	NONE	66751831	66938587		0					OTHER		complex event
7-143	7	67601472	67631075	Misassembly	NONE	67601472	67631075		0					OTHER		complex event
7-144	7	67899483	67950876	Misassembly	NONE	67899483	67950876		0					OTHER		complex event
7-145	7	68030614	68099421	Misassembly	NONE	68030614	68099421		0					OTHER		complex event
7-148	7	69427818	69670170	Misassembly	NONE	69427818	69670170		0					OTHER		complex event
7-149	7	70229123	70287966	Misassembly	NONE	70229123	70287966		0					OTHER		complex event - possible inversion
7-150	7	70449322	70730456	Misassembly	NONE	70449322	70730456		0					OTHER		complex event
7-151	7	70810275	71025630	Misassembly	NONE	70810275	71025630		0					OTHER		complex event
7-152	7	71056306	71291964	Misassembly	NONE	71056306	71291964		0					OTHER		complex event
7-153	7	71368077	71656743	Misassembly	NONE	71368077	71656743		0					OTHER		complex event
7-155	7	71840295	72050760	Misassembly	NONE	71840295	72050760		0					OTHER		complex event
7-157	7	72375722	72507809	Misassembly	NONE	72375722	72507809		0					OTHER		complex event
7-158	7	72792769	72932723	Misassembly	NONE	72792769	72932723		0					OTHER		complex event
7-159	7	73471967	73518010	Misassembly	NONE	73471967	73518010		0					OTHER		complex event
7-160	7	73622492	73795869	Misassembly	NONE	73622492	73795869		0					OTHER		complex event
7-161	7	74011616	74072050	Misassembly	NONE	74011616	74072050		0					OTHER		complex event
7-162	7	74217723	74592159	Misassembly	NONE	74217723	74592159		0					OTHER		complex event
7-163	7	74832840	75012450	Misassembly	NONE	74832840	75012450		0					OTHER		complex event
7-164	7	75317036	75351858	Misassembly	NONE	75317036	75351858		0					OTHER		complex event
7-165	7	75549107	75636028	Misassembly	NONE	75549107	75636028		0					OTHER		complex event
7-166	7	75661314	75863891	Misassembly	NONE	75661314	75863891		0					OTHER		complex event
7-167	7	76194092	76472268	Misassembly	NONE	76194092	76472268		0					OTHER		complex event
7-169	7	76867729	76982233	Misassembly	NONE	76867729	76982233		0					OTHER		complex event - possible inversion
7-171	7	77340490	77458796	Misassembly	NONE	77340490	77458796		0					OTHER		complex event
7-172	7	77864854	77959552	Misassembly	NONE	77864854	77959552		0					OTHER		complex event
7-173	7	77991492	78128768	Misassembly	NONE	77991492	78128768		0					OTHER		complex event
7-175	7	78396656	78528717	Misassembly	NONE	78396656	78528717		0					OTHER		complex event
7-176	7	78752664	78819772	Misassembly	NONE	78752664	78819772		0					OTHER		complex event - possible inversion
7-178	7	79738751	79810110	Misassembly	NONE	79738751	79810110		0					OTHER		complex event
7-180	7	80268880	80500696	Misassembly	NONE	80268880	80500696		0					OTHER		complex event
7-181	7	80608202	80802543	Misassembly	NONE	80608202	80802543		0					OTHER		complex event
7-183	7	83350363	83456964	Misassembly	NONE	83350363	83456964		0					OTHER		
7-186	7	84890783	85054670	Misassembly	NONE	84890783	85054670		0					OTHER		complex event
7-187	7	85343982	85432474	Misassembly	NONE	85343982	85432474		0					OTHER		complex event
7-188	7	85616709	85738391	Misassembly	NONE	85616709	85738391		0					OTHER		complex event
7-189	7	85771977	85859852	Misassembly	NONE	85771977	85859852		0					OTHER		complex event
7-192	7	89409328	89498040	Misassembly	NONE	89409328	89498040		0					OTHER		
7-193	7	89544607	89603187	Misassembly	NONE	89544607	89603187		0					OTHER		complex event
7-194	7	89640264	89729115	Misassembly	NONE	89640264	89729115		0					OTHER		complex event
7-195	7	89894376	90028953	Misassembly	NONE	89894376	90028953		0					OTHER		complex event
7-197	7	90315442	90408953	Misassembly	NONE	90315442	90408953		0					OTHER		complex event
7-199	7	90652475	90669639	Misassembly	NONE	90652475	90669639		0					OTHER		complex event
7-200	7	90807915	91014670	Misassembly	NONE	90807915	91014670		0					OTHER		complex event
7-201	7	91209093	91531240	Misassembly	NONE	91209093	91531240		0					OTHER		complex event
7-202	7	91660513	91939580	Misassembly	NONE	91660513	91939580		0					OTHER		complex event
7-203	7	91985397	92058492	Misassembly	NONE	91985397	92058492		0					OTHER		complex event - possible inversion
7-204	7	92372914	92688325	Misassembly	NONE	92372914	92688325		0					OTHER		complex event
7-206	7	93707294	93835917	Misassembly	NONE	93707294	93835917		0					OTHER		complex event
7-209	7	94223564	94352987	Misassembly	NONE	94223564	94352987		0					OTHER		complex event
7-211	7	94691710	94834784	Misassembly	NONE	94691710	94834784		0					OTHER		complex event
7-212	7	94895891	94966746	Misassembly	NONE	94895891	94966746		0					OTHER		complex event
7-216	7	98138167	98187078	Misassembly	NONE	98138167	98187078		0					OTHER		complex event
7-218	7	98593925	98846258	Misassembly	NONE	98593925	98846258		0					OTHER		complex event
7-219	7	98957308	99011113	Misassembly	NONE	98957308	99011113		0					OTHER		complex event
7-222	7	99376746	99460185	Misassembly	NONE	99376746	99460185		0					OTHER		complex event
7-223	7	99668482	99822603	Misassembly	NONE	99668482	99822603		0					OTHER		complex event
7-224	7	99960154	100055911	Misassembly	NONE	99960154	100055911		0					OTHER		complex event
7-225	7	100350017	100489235	Misassembly	NONE	100350017	100489235		0					OTHER		complex event
7-226	7	100541550	100683543	Misassembly	NONE	100541550	100683543		0					OTHER		complex event
7-227	7	100772800	100844357	Misassembly	NONE	100772800	100844357		0					OTHER		complex event
7-229	7	101410755	101579553	Misassembly	NONE	101410755	101579553		0					OTHER		complex event
7-231	7	101843846	101882409	Misassembly	NONE	101843846	101882409		0					OTHER		complex event
7-232	7	105935666	106190394	Misassembly	NONE	105935666	106190394		0					OTHER		complex event
7-234	7	106615501	106725810	Misassembly	NONE	106615501	106725810		0					OTHER		complex event
7-235	7	106817515	106845516	Misassembly	NONE	106817515	106845516		0					OTHER		complex event - possible inversion
7-236	7	106891693	107014786	Misassembly	NONE	106891693	107014786		0					OTHER		complex event
7-237	7	107072687	107154663	Misassembly	NONE	107072687	107154663		0					OTHER		complex event

7-240	7	108432672	108540688	Misassembly	NONE	108432672	108540688		0					OTHER		complex event
7-242	7	110364834	110463861	Misassembly	NONE	110364834	110463861		0					OTHER		complex event
7-244	7	113211376	113264599	Misassembly	NONE	113211376	113264599		0					OTHER		complex event
7-248	7	113810436	113957512	Misassembly	NONE	113810436	113957512		0					OTHER		complex event
7-249	7	114101706	114332695	Misassembly	NONE	114101706	114332695		0					OTHER		complex event
7-250	7	114548145	114660281	Misassembly	NONE	114548145	114660281		0					OTHER		complex event
7-251	7	114808667	114850499	Misassembly	NONE	114808667	114850499		0					OTHER		complex event
7-252	7	114996493	115048615	Misassembly	NONE	114996493	115048615		0					OTHER		complex event
7-254	7	115290391	115364619	Misassembly	NONE	115290391	115364619		0					OTHER		complex event
7-256	7	117388650	117502701	Misassembly	NONE	117388650	117502701		0					OTHER		complex event
7-257	7	117562338	117680317	Misassembly	NONE	117562338	117680317		0					OTHER		complex event
7-262	7	120453446	120545406	Misassembly	NONE	120453446	120545406		0					OTHER		complex event
7-263	7	120721097	120810151	Misassembly	NONE	120721097	120810151		0					OTHER		complex event
7-266	7	127077514	127258626	Misassembly	NONE	127077514	127258626		0					OTHER		complex event
7-267	7	127289941	127369157	Misassembly	NONE	127289941	127369157		0					OTHER		complex event
7-268	7	131802388	131867281	Misassembly	NONE	131802388	131867281		0					OTHER		complex event
7-269	7	131911107	132087951	Misassembly	NONE	131911107	132087951		0					OTHER		complex event
7-270	7	133250150	133325359	Misassembly	NONE	133250150	133325359		0					OTHER		complex event
7-271	7	133416821	133507764	Misassembly	NONE	133416821	133507764		0					OTHER		complex event - possible inversion
7-273	7	135320870	135580962	Misassembly	NONE	135320870	135580962		0					OTHER		complex event
7-274	7	135645104	135846744	Misassembly	NONE	135645104	135846744		0					OTHER		complex event
7-275	7	135943606	136210736	Misassembly	NONE	135943606	136210736		0					OTHER		complex event
7-276	7	136416544	136755647	Misassembly	NONE	136416544	136755647		0					OTHER		complex event
7-277	7	136962526	137212336	Misassembly	NONE	136962526	137212336		0					OTHER		complex event
7-278	7	137568386	137651894	Misassembly	NONE	137568386	137651894		0					OTHER		complex event
7-279	7	137692441	137760985	Misassembly	NONE	137692441	137760985		0					OTHER		complex event
7-280	7	137866217	137914699	Misassembly	NONE	137866217	137914699		0					OTHER		complex event
7-281	7	137964908	137995183	Misassembly	NONE	137964908	137995183		0					OTHER		complex event
7-282	7	138105567	138212999	Misassembly	NONE	138105567	138212999		0					OTHER		complex event
7-284	7	138407599	138476173	Misassembly	NONE	138407599	138476173		0					OTHER		complex event - possible inversion
7-286	7	139094846	139214552	Misassembly	NONE	139094846	139214552		0					OTHER		complex event
7-287	7	139470764	139516930	Misassembly	NONE	139470764	139516930		0					OTHER		complex event
7-289	7	139795174	139841216	Misassembly	NONE	139795174	139841216		0					OTHER		complex event
7-290	7	139895818	140175349	Misassembly	NONE	139895818	140175349		0					OTHER		complex event
7-294	7	144287662	144340952	Misassembly	NONE	144287662	144340952		0					OTHER		complex event - possible inversion
7-297	7	146640377	146655590	Misassembly	NONE	146640377	146655590		0					OTHER		complex event
7-298	7	146817042	146901201	Misassembly	NONE	146817042	146901201		0					OTHER		complex event
7-299	7	146991170	147138484	Misassembly	NONE	146991170	147138484		0					OTHER		complex event
7-300	7	152598442	152735421	Misassembly	NONE	152598442	152735421		0					OTHER		complex event
7-301	7	152778858	152874537	Misassembly	NONE	152778858	152874537		0					OTHER		complex event - possible inversion
7-304	7	153617478	153736394	Misassembly	NONE	153617478	153736394		0					OTHER		complex event
7-306	7	155540150	155618578	Misassembly	NONE	155540150	155618578		0					OTHER		complex event
7-307	7	159259249	159341439	Misassembly	NONE	159259249	159341439		0					OTHER		complex event
7-309	7	160499301	160647938	Misassembly	NONE	160499301	160647938		0					OTHER		
7-310	7	163137644	163211755	Misassembly	NONE	163137644	163211755		0					OTHER		complex event
7-311	7	164583411	164651928	Misassembly	NONE	164583411	164651928		0					OTHER		complex event
7-312	7	164699437	164781219	Misassembly	NONE	164699437	164781219		0					OTHER		complex event
7-313	7	165169015	165217033	Misassembly	NONE	165169015	165217033		0					OTHER		complex event
7-314	7	165290991	165308326	Misassembly	NONE	165290991	165308326		0					OTHER		complex event - possible inversion
7-315	7	168485694	168647825	Misassembly	NONE	168485694	168647825		0					OTHER		complex event
7-316	7	169072113	169187691	Misassembly	NONE	169072113	169187691		0					OTHER		
7-318	7	170615439	170682997	Misassembly	NONE	170615439	170682997		0					OTHER		complex event
8-1	8	1546664	1625343	Misassembly	NONE	1546664	1625343		0					OTHER		complex event
8-2	8	1873856	1974568	Misassembly	NONE	1873856	1974568		0					OTHER		complex event
8-10	8	5641863	5700338	Misassembly	NONE	5641863	5700338		0					OTHER		complex event
8-12	8	5901850	6044753	Misassembly	NONE	5901850	6044753		0					OTHER		complex event
8-15	8	8335683	8524160	Misassembly	NONE	8335683	8524160		0					OTHER		complex event
8-17	8	8707739	8845545	Misassembly	NONE	8707739	8845545		0					OTHER		complex event
8-20	8	9127586	9177599	Misassembly	NONE	9127586	9177599		0					OTHER		complex event
8-21	8	9416135	9554601	Misassembly	NONE	9416135	9554601		0					OTHER		complex event
8-22	8	9700405	9891959	Misassembly	NONE	9700405	9891959		0					OTHER		complex event
8-23	8	9912264	10027695	Misassembly	NONE	9912264	10027695		0					OTHER		complex event
8-24	8	10164863	10187616	Misassembly	NONE	10164863	10187616		0					OTHER		complex event
8-25	8	10419059	10508175	Misassembly	NONE	10419059	10508175		0					OTHER		complex event
8-26	8	10625597	10909982	Misassembly	NONE	10625597	10909982		0					OTHER		complex event
8-27	8	11177456	11266758	Misassembly	NONE	11177456	11266758		0					OTHER		complex event
8-31	8	11768769	11835247	Misassembly	NONE	11768769	11835247		0					OTHER		complex event
8-32	8	11916875	11966228	Misassembly	NONE	11916875	11966228		0					OTHER		complex event
8-33	8	12089132	12167103	Misassembly	NONE	12089132	12167103		0					OTHER		complex event
8-36	8	12302214	12393910	Misassembly	NONE	12302214	12393910		0					OTHER		complex event
8-37	8	12503115	12661018	Misassembly	NONE	12503115	12661018		0					OTHER		complex event
8-39	8	13118851	13378192	Misassembly	NONE	13118851	13378192		0					OTHER		complex event
8-40	8	13429954	13650507	Misassembly	NONE	13429954	13650507		0					OTHER		complex event
8-44	8	15666199	15713290	Misassembly	NONE	15666199	15713290		0					OTHER		complex event
8-47	8	16165488	16228598	Misassembly	NONE	16165488	16228598		0					OTHER		complex event
8-50	8	16491595	16657269	Misassembly	NONE	16491595	16657269		0					OTHER		complex event
8-53	8	17277375	17399013	Misassembly	NONE	17277375	17399013		0					OTHER		complex event
8-55	8	18493597	18666466	Misassembly	NONE	18493597	18666466		0					OTHER		complex event
8-61	8	20458733	20696114	Misassembly	NONE	20458733	20696114		0					OTHER		complex event
8-62	8	20762772	20845782	Misassembly	NONE	20762772	20845782		0					OTHER		complex event
8-67	8	21591450	21650934	Misassembly	NONE	21591450	21650934		0					OTHER		complex event

8-74	8	24564124	24620214	Misassembly	NONE	24564124	24620214		0					OTHER		complex event
8-76	8	25020752	25125151	Misassembly	NONE	25020752	25125151		0					OTHER		complex event
8-77	8	25239875	25257284	Misassembly	NONE	25239875	25257284		0					OTHER		complex event
8-79	8	25486245	25648260	Misassembly	NONE	25486245	25648260		0					OTHER		complex event
8-80	8	26312466	26417814	Misassembly	NONE	26312466	26417814		0					OTHER		complex event
8-82	8	26563633	26616757	Misassembly	NONE	26563633	26616757		0					OTHER		complex event
8-87	8	27375035	27400248	Misassembly	NONE	27375035	27400248		0					OTHER		complex event
8-88	8	27519008	27623242	Misassembly	NONE	27519008	27623242		0					OTHER		complex event
8-89	8	27663202	27749353	Misassembly	NONE	27663202	27749353		0					OTHER		complex event
8-90	8	27793269	27986647	Misassembly	NONE	27793269	27986647		0					OTHER		complex event
8-91	8	28014932	28092350	Misassembly	NONE	28014932	28092350		0					OTHER		complex event
8-92	8	28691890	28909937	Misassembly	NONE	28691890	28909937		0					OTHER		complex event
8-94	8	29393265	29496424	Misassembly	NONE	29393265	29496424		0					OTHER		complex event
8-96	8	29983370	30203893	Misassembly	NONE	29983370	30203893		0					OTHER		complex event
8-97	8	30387959	30458955	Misassembly	NONE	30387959	30458955		0					OTHER		complex event
8-99	8	32893584	32991675	Misassembly	NONE	32893584	32991675		0					OTHER		complex event
8-106	8	34822977	34933151	Misassembly	NONE	34822977	34933151		0					OTHER		complex event
8-107	8	35152867	35287267	Misassembly	NONE	35152867	35287267		0					OTHER		complex event
8-110	8	35631335	35704888	Misassembly	NONE	35631335	35704888		0					OTHER		complex event - possible inversion
8-111	8	35779863	36000150	Misassembly	NONE	35779863	36000150		0					OTHER		complex event - possible inversion
8-113	8	37520274	37642351	Misassembly	NONE	37520274	37642351		0					OTHER		complex event
8-115	8	37875816	37926185	Misassembly	NONE	37875816	37926185		0					OTHER		complex event
8-117	8	38244307	38525698	Misassembly	NONE	38244307	38525698		0					OTHER		complex event
8-120	8	38895649	39016148	Misassembly	NONE	38895649	39016148		0					OTHER		complex event
8-121	8	39076920	39167523	Misassembly	NONE	39076920	39167523		0					OTHER		complex event
8-126	8	40380870	40431506	Misassembly	NONE	40380870	40431506		0					OTHER		complex event
8-128	8	40867008	40932322	Misassembly	NONE	40867008	40932322		0					OTHER		complex event
8-129	8	40987906	41318106	Misassembly	NONE	40987906	41318106		0					OTHER		complex event
8-130	8	41796826	41825724	Misassembly	NONE	41796826	41825724		0					OTHER		complex event
8-131	8	42205358	42330002	Misassembly	NONE	42205358	42330002		0					OTHER		complex event
8-132	8	42450853	42599312	Misassembly	NONE	42450853	42599312		0					OTHER		complex event
8-133	8	42653608	42823670	Misassembly	NONE	42653608	42823670		0					OTHER		complex event
8-136	8	43203149	43430416	Misassembly	NONE	43203149	43430416		0					OTHER		complex event
8-138	8	43761151	43962026	Misassembly	NONE	43761151	43962026		0					OTHER		complex event
8-139	8	44306510	44372854	Misassembly	NONE	44306510	44372854		0					OTHER		complex event
8-141	8	44527912	44695718	Misassembly	NONE	44527912	44695718		0					OTHER		complex event
8-143	8	44914699	44995206	Misassembly	NONE	44914699	44995206		0					OTHER		complex event
8-145	8	50036780	50080912	Misassembly	NONE	50036780	50080912		0					OTHER		complex event - possible inversion
8-148	8	50742709	50875245	Misassembly	NONE	50742709	50875245		0					OTHER		complex event
8-149	8	50934291	51302170	Misassembly	NONE	50934291	51302170		0					OTHER		complex event
8-150	8	51595451	51786232	Misassembly	NONE	51595451	51786232		0					OTHER		complex event
8-155	8	54094165	54264010	Misassembly	NONE	54094165	54264010		0					OTHER		complex event
8-156	8	54503493	54593852	Misassembly	NONE	54503493	54593852		0					OTHER		complex event
8-160	8	55432077	55537885	Misassembly	NONE	55432077	55537885		0					OTHER		complex event
8-162	8	55679873	55706471	Misassembly	NONE	55679873	55706471		0					OTHER		complex event
8-166	8	56216677	56353853	Misassembly	NONE	56216677	56353853		0					OTHER		complex event
8-167	8	59199087	59324070	Misassembly	NONE	59199087	59324070		0					OTHER		complex event
8-168	8	59400646	59503989	Misassembly	NONE	59400646	59503989		0					OTHER		complex event
8-173	8	60876580	61052430	Misassembly	NONE	60876580	61052430		0					OTHER		complex event
8-174	8	61186016	61351827	Misassembly	NONE	61186016	61351827		0					OTHER		complex event
8-176	8	63507856	63555898	Misassembly	NONE	63507856	63555898		0					OTHER		complex event
8-177	8	63868349	63898028	Misassembly	NONE	63868349	63898028		0					OTHER		complex event
8-182	8	64245258	64406256	Misassembly	NONE	64245258	64406256		0					OTHER		complex event
8-183	8	64454855	64499774	Misassembly	NONE	64454855	64499774		0					OTHER		complex event
8-184	8	64627628	64727713	Misassembly	NONE	64627628	64727713		0					OTHER		complex event
8-186	8	65010503	65354778	Misassembly	NONE	65010503	65354778		0					OTHER		complex event
8-187	8	65441697	65547469	Misassembly	NONE	65441697	65547469		0					OTHER		complex event
8-189	8	66056913	66357997	Misassembly	NONE	66056913	66357997		0					OTHER		complex event
8-190	8	66507421	66711617	Misassembly	NONE	66507421	66711617		0					OTHER		complex event
8-192	8	68592631	68724398	Misassembly	NONE	68592631	68724398		0					OTHER		complex event
8-193	8	68750782	68880884	Misassembly	NONE	68750782	68880884		0					OTHER		complex event
8-194	8	69113334	69204189	Misassembly	NONE	69113334	69204189		0					OTHER		complex event
8-197	8	69943730	70056646	Misassembly	NONE	69943730	70056646		0					OTHER		complex event
8-203	8	71616287	71930268	Misassembly	NONE	71616287	71930268		0					OTHER		complex event
8-204	8	72033747	72342910	Misassembly	NONE	72033747	72342910		0					OTHER		complex event
8-205	8	72480209	72534774	Misassembly	NONE	72480209	72534774		0					OTHER		complex event
8-206	8	72614266	72721865	Misassembly	NONE	72614266	72721865		0					OTHER		complex event
8-207	8	73074004	73773360	Misassembly	NONE	73074004	73773360		0					OTHER		complex event
8-208	8	73883976	73920205	Misassembly	NONE	73883976	73920205		0					OTHER		complex event
8-211	8	74652532	74817358	Misassembly	NONE	74652532	74817358		0					OTHER		complex event
8-212	8	75261454	75455621	Misassembly	NONE	75261454	75455621		0					OTHER		complex event
8-213	8	75486221	75571789	Misassembly	NONE	75486221	75571789		0					OTHER		complex event
8-214	8	75596977	75863424	Misassembly	NONE	75596977	75863424		0					OTHER		complex event
8-217	8	76325466	76425016	Misassembly	NONE	76325466	76425016		0					OTHER		complex event
8-218	8	76624934	76773208	Misassembly	NONE	76624934	76773208		0					OTHER		complex event
8-220	8	76901631	76968560	Misassembly	NONE	76901631	76968560		0					OTHER		complex event
8-222	8	78877658	78946918	Misassembly	NONE	78877658	78946918		0					OTHER		complex event
8-223	8	79021564	79120254	Misassembly	NONE	79021564	79120254		0					OTHER		complex event
8-224	8	79237341	79290338	Misassembly	NONE	79237341	79290338		0					OTHER		complex event
8-225	8	79369710	79589296	Misassembly	NONE	79369710	79589296		0					OTHER		complex event
8-226	8	79646979	79671068	Misassembly	NONE	79646979	79671068		0					OTHER		complex event - possible inversion
8-227	8	79745883	79877734	Misassembly	NONE	79745883	79877734		0					OTHER		complex event

8-228	8	79986934	80005497	Misassembly	NONE	79986934	80005497		0					OTHER		complex event - possible inversion
8-230	8	80303664	80453384	Misassembly	NONE	80303664	80453384		0					OTHER		complex event
8-231	8	80585004	80705381	Misassembly	NONE	80585004	80705381		0					OTHER		complex event
8-232	8	82190058	82323073	Misassembly	NONE	82190058	82323073		0					OTHER		complex event
8-235	8	82735368	82952848	Misassembly	NONE	82735368	82952848		0					OTHER		complex event
8-236	8	83051487	83132891	Misassembly	NONE	83051487	83132891		0					OTHER		complex event
8-237	8	83888402	83986506	Misassembly	NONE	83888402	83986506		0					OTHER		complex event
8-238	8	84158703	84224203	Misassembly	NONE	84158703	84224203		0					OTHER		complex event
8-240	8	86106736	86180833	Misassembly	NONE	86106736	86180833		0					OTHER		complex event
8-241	8	89208534	89327727	Misassembly	NONE	89208534	89327727		0					OTHER		complex event
8-243	8	89819053	89904772	Misassembly	NONE	89819053	89904772		0					OTHER		complex event
8-244	8	90107114	90228575	Misassembly	NONE	90107114	90228575		0					OTHER		complex event
8-245	8	91729824	91828978	Misassembly	NONE	91729824	91828978		0					OTHER		complex event
8-246	8	92037930	92353978	Misassembly	NONE	92037930	92353978		0					OTHER		complex event
8-247	8	92458013	92582824	Misassembly	NONE	92458013	92582824		0					OTHER		complex event
8-249	8	93028602	93172020	Misassembly	NONE	93028602	93172020		0					OTHER		complex event
8-250	8	93338591	93513015	Misassembly	NONE	93338591	93513015		0					OTHER		complex event
8-251	8	93552015	93677783	Misassembly	NONE	93552015	93677783		0					OTHER		complex event
8-252	8	97151118	97401067	Misassembly	NONE	97151118	97401067		0					OTHER		complex event
8-255	8	99975973	100027679	Misassembly	NONE	99975973	100027679		0					OTHER		complex event
8-257	8	100218316	100311767	Misassembly	NONE	100218316	100311767		0					OTHER		complex event
8-258	8	103839497	103977290	Misassembly	NONE	103839497	103977290		0					OTHER		complex event
8-260	8	107210377	107364564	Misassembly	NONE	107210377	107364564		0					OTHER		complex event
8-261	8	108647559	108841978	Misassembly	NONE	108647559	108841978		0					OTHER		complex event
8-264	8	110721961	110909589	Misassembly	NONE	110721961	110909589		0					OTHER		complex event
8-266	8	112616432	112717359	Misassembly	NONE	112616432	112717359		0					OTHER		complex event
8-267	8	112905384	113045842	Misassembly	NONE	112905384	113045842		0					OTHER		complex event
8-271	8	113689896	113805544	Misassembly	NONE	113689896	113805544		0					OTHER		complex event
8-275	8	118126613	118348472	Misassembly	NONE	118126613	118348472		0					OTHER		complex event
8-279	8	119149831	119262712	Misassembly	NONE	119149831	119262712		0					OTHER		complex event
8-282	8	119700332	119789456	Misassembly	NONE	119700332	119789456		0					OTHER		complex event
8-283	8	119881340	120003652	Misassembly	NONE	119881340	120003652		0					OTHER		complex event
8-284	8	120061980	120325757	Misassembly	NONE	120061980	120325757		0					OTHER		complex event
8-287	8	127340318	127385614	Misassembly	NONE	127340318	127385614		0					OTHER		complex event
8-290	8	128009368	128141938	Misassembly	NONE	128009368	128141938		0					OTHER		complex event
8-291	8	128192032	128291844	Misassembly	NONE	128192032	128291844		0					OTHER		complex event
8-292	8	128413277	128835356	Misassembly	NONE	128413277	128835356		0					OTHER		complex event
8-294	8	129020063	129087064	Misassembly	NONE	129020063	129087064		0					OTHER		complex event
8-297	8	129292858	129457157	Misassembly	NONE	129292858	129457157		0					OTHER		complex event
8-300	8	130024642	130091276	Misassembly	NONE	130024642	130091276		0					OTHER		complex event
8-301	8	130304038	130433662	Misassembly	NONE	130304038	130433662		0					OTHER		complex event
8-305	8	130892046	130923907	Misassembly	NONE	130892046	130923907		0					OTHER		complex event - possible inversion
8-307	8	131186583	131259339	Misassembly	NONE	131186583	131259339		0					OTHER		complex event
8-310	8	134264585	134348531	Misassembly	NONE	134264585	134348531		0					OTHER		complex event
8-312	8	134700127	134895153	Misassembly	NONE	134700127	134895153		0					OTHER		complex event
8-313	8	135022932	135084756	Misassembly	NONE	135022932	135084756		0					OTHER		complex event
8-315	8	135543385	135580206	Misassembly	NONE	135543385	135580206		0					OTHER		complex event
8-316	8	135719943	136005211	Misassembly	NONE	135719943	136005211		0					OTHER		complex event
8-317	8	136035527	136063870	Misassembly	NONE	136035527	136063870		0					OTHER		complex event - possible inversion
8-318	8	136096410	136156214	Misassembly	NONE	136096410	136156214		0					OTHER		complex event
8-321	8	136447780	136493647	Misassembly	NONE	136447780	136493647		0					OTHER		complex event
8-323	8	136600695	136676462	Misassembly	NONE	136600695	136676462		0					OTHER		complex event
8-329	8	144689711	144807141	Misassembly	NONE	144689711	144807141		0					OTHER		complex event - possible inversion
8-331	8	145364784	145376587	Misassembly	NONE	145364784	145376587		0					OTHER		complex event - possible inversion
8-335	8	148027291	148366181	Misassembly	NONE	148027291	148366181		0					OTHER		complex event
8-339	8	148925129	149088815	Misassembly	NONE	148925129	149088815		0					OTHER		complex event
8-340	8	149220481	149404270	Misassembly	NONE	149220481	149404270		0					OTHER		complex event
8-341	8	149452076	149497032	Misassembly	NONE	149452076	149497032		0					OTHER		complex event
8-342	8	149593117	149713181	Misassembly	NONE	149593117	149713181		0					OTHER		complex event
8-347	8	150279794	150391119	Misassembly	NONE	150279794	150391119		0					OTHER		complex event
8-348	8	150541951	150605335	Misassembly	NONE	150541951	150605335		0					OTHER		complex event
8-349	8	150638246	150983874	Misassembly	NONE	150638246	150983874		0					OTHER		complex event
8-352	8	158232843	158398200	Misassembly	NONE	158232843	158398200		0					OTHER		complex event
8-353	8	158777932	159098014	Misassembly	NONE	158777932	159098014		0					OTHER		complex event
8-354	8	159320170	159381669	Misassembly	NONE	159320170	159381669		0					OTHER		complex event
8-355	8	159469311	159576277	Misassembly	NONE	159469311	159576277		0					OTHER		complex event
8-356	8	159672624	159845231	Misassembly	NONE	159672624	159845231		0					OTHER		complex event
8-358	8	160052778	160176034	Misassembly	NONE	160052778	160176034		0					OTHER		complex event
8-359	8	160327809	160358425	Misassembly	NONE	160327809	160358425		0					OTHER		complex event - possible inversion
8-360	8	160704395	160770742	Misassembly	NONE	160704395	160770742		0					OTHER		complex event
8-361	8	160832428	160943608	Misassembly	NONE	160832428	160943608		0					OTHER		complex event - possible inversion
8-364	8	161609977	161737472	Misassembly	NONE	161609977	161737472		0					OTHER		complex event
8-365	8	162013864	162051449	Misassembly	NONE	162013864	162051449		0					OTHER		complex event
8-374	8	170778665	170886796	Misassembly	NONE	170778665	170886796		0					OTHER		complex event
8-379	8	171552398	171616634	Misassembly	NONE	171552398	171616634		0					OTHER		complex event

8-380	8	172728726	172785782	Misassembly	NONE	172728726	172785782		0					OTHER		complex event - possible inversion
8-381	8	172853476	172951076	Misassembly	NONE	172853476	172951076		0					OTHER		complex event - possible inversion
8-382	8	173075203	173241632	Misassembly	NONE	173075203	173241632		0					OTHER		complex event
8-383	8	173614491	173761824	Misassembly	NONE	173614491	173761824		0					OTHER		complex event
9-3	9	4395934	4454559	Misassembly	NONE	4395934	4454559		0					OTHER		complex event
9-5	9	4613504	4704681	Misassembly	NONE	4613504	4704681		0					OTHER		complex event
9-6	9	5180212	5395851	Misassembly	NONE	5180212	5395851		0					OTHER		complex event
9-7	9	5428415	5488470	Misassembly	NONE	5428415	5488470		0					OTHER		complex event
9-8	9	5531399	5612394	Misassembly	NONE	5531399	5612394		0					OTHER		complex event
9-10	9	5829773	5940504	Misassembly	NONE	5829773	5940504		0					OTHER		complex event
9-13	9	9738712	9845008	Misassembly	NONE	9738712	9845008		0					OTHER		complex event
9-16	9	16171472	16263425	Misassembly	NONE	16171472	16263425		0					OTHER		complex event
9-23	9	20410766	20582389	Misassembly	NONE	20410766	20582389		0					OTHER		complex event
9-24	9	20872949	21006721	Misassembly	NONE	20872949	21006721		0					OTHER		complex event
9-26	9	21380045	21441792	Misassembly	NONE	21380045	21441792		0					OTHER		complex event
9-28	9	21543234	21736528	Misassembly	NONE	21543234	21736528		0					OTHER		complex event
9-32	9	24943326	25005845	Misassembly	NONE	24943326	25005845		0					OTHER		complex event
9-34	9	25238171	25273963	Misassembly	NONE	25238171	25273963		0					OTHER		complex event
9-35	9	25806418	25916299	Misassembly	NONE	25806418	25916299		0					OTHER		complex event
9-36	9	25981113	26073069	Misassembly	NONE	25981113	26073069		0					OTHER		complex event - possible inversion
9-37	9	26237588	26596613	Misassembly	NONE	26237588	26596613		0					OTHER		complex event
9-38	9	26629249	26726618	Misassembly	NONE	26629249	26726618		0					OTHER		complex event
9-39	9	26870201	26980870	Misassembly	NONE	26870201	26980870		0					OTHER		complex event
9-40	9	29460411	29564619	Misassembly	NONE	29460411	29564619		0					OTHER		complex event
9-43	9	30805976	30926946	Misassembly	NONE	30805976	30926946		0					OTHER		complex event
9-44	9	32173954	32289039	Misassembly	NONE	32173954	32289039		0					OTHER		
9-45	9	32390505	32457660	Misassembly	NONE	32390505	32457660		0					OTHER		complex event
9-46	9	32626500	33111308	Misassembly	NONE	32626500	33111308		0					OTHER		complex event
9-48	9	33255397	33277999	Misassembly	NONE	33255397	33277999		0					OTHER		complex event
9-49	9	33493530	33582450	Misassembly	NONE	33493530	33582450		0					OTHER		complex event
9-51	9	34117078	34206159	Misassembly	NONE	34117078	34206159		0					OTHER		complex event
9-52	9	34400646	34476830	Misassembly	NONE	34400646	34476830		0					OTHER		complex event
9-53	9	34688108	34948173	Misassembly	NONE	34688108	34948173		0					OTHER		complex event
9-54	9	35485306	35777830	Misassembly	NONE	35485306	35777830		0					OTHER		complex event
9-55	9	35998671	36106414	Misassembly	NONE	35998671	36106414		0					OTHER		complex event
9-56	9	36171653	36486181	Misassembly	NONE	36171653	36486181		0					OTHER		complex event
9-58	9	37035408	37188585	Misassembly	NONE	37035408	37188585		0					OTHER		complex event
9-59	9	39117710	39171967	Misassembly	NONE	39117710	39171967		0					OTHER		complex event
9-61	9	39719287	39887673	Misassembly	NONE	39719287	39887673		0					OTHER		complex event
9-62	9	39975058	40040151	Misassembly	NONE	39975058	40040151		0					OTHER		complex event - possible inversion
9-64	9	40225889	40346332	Misassembly	NONE	40225889	40346332		0					OTHER		complex event
9-65	9	40396092	40668316	Misassembly	NONE	40396092	40668316		0					OTHER		complex event
9-67	9	40855002	40936279	Misassembly	NONE	40855002	40936279		0					OTHER		complex event
9-68	9	41006742	41211457	Misassembly	NONE	41006742	41211457		0					OTHER		complex event
9-69	9	41369551	41574641	Misassembly	NONE	41369551	41574641		0					OTHER		complex event
9-70	9	41603580	41649191	Misassembly	NONE	41603580	41649191		0					OTHER		complex event - possible inversion
9-71	9	41868165	41987613	Misassembly	NONE	41868165	41987613		0					OTHER		complex event
9-72	9	42083514	42236411	Misassembly	NONE	42083514	42236411		0					OTHER		complex event
9-73	9	42288859	42429477	Misassembly	NONE	42288859	42429477		0					OTHER		complex event
9-77	9	43160504	43212082	Misassembly	NONE	43160504	43212082		0					OTHER		complex event
9-78	9	43241793	43290050	Misassembly	NONE	43241793	43290050		0					OTHER		complex event
9-80	9	43682764	43738822	Misassembly	NONE	43682764	43738822		0					OTHER		complex event
9-81	9	43861812	44034745	Misassembly	NONE	43861812	44034745		0					OTHER		complex event
9-82	9	47881938	47980900	Misassembly	NONE	47881938	47980900		0					OTHER		complex event
9-83	9	48247512	48363088	Misassembly	NONE	48247512	48363088		0					OTHER		complex event
9-89	9	49989756	50086512	Misassembly	NONE	49989756	50086512		0					OTHER		complex event
9-91	9	50198240	50403332	Misassembly	NONE	50198240	50403332		0					OTHER		complex event
9-92	9	50550768	50661736	Misassembly	NONE	50550768	50661736		0					OTHER		complex event
9-95	9	52847985	53104250	Misassembly	NONE	52847985	53104250		0					OTHER		complex event
9-97	9	54631541	54669926	Misassembly	NONE	54631541	54669926		0					OTHER		complex event
9-100	9	55289410	55518958	Misassembly	NONE	55289410	55518958		0					OTHER		complex event
9-101	9	55606300	55722929	Misassembly	NONE	55606300	55722929		0					OTHER		complex event
9-108	9	58490112	58597222	Misassembly	NONE	58490112	58597222		0					OTHER		complex event
9-109	9	58786241	58887195	Misassembly	NONE	58786241	58887195		0					OTHER		complex event
9-112	9	59743933	59820600	Misassembly	NONE	59743933	59820600		0					OTHER		complex event
9-114	9	60848127	61054391	Misassembly	NONE	60848127	61054391		0					OTHER		complex event
9-115	9	61152177	61490537	Misassembly	NONE	61152177	61490537		0					OTHER		complex event
9-116	9	61660588	61761484	Misassembly	NONE	61660588	61761484		0					OTHER		complex event
9-117	9	63296793	63906095	Misassembly	NONE	63296793	63906095		0					OTHER		complex event
9-119	9	64824135	64901943	Misassembly	NONE	64824135	64901943		0					OTHER		complex event
9-121	9	65171383	65368081	Misassembly	NONE	65171383	65368081		0					OTHER		complex event
9-122	9	65471003	65628019	Misassembly	NONE	65471003	65628019		0					OTHER		complex event
9-123	9	65652865	65713742	Misassembly	NONE	65652865	65713742		0					OTHER		complex event - possible inversion
9-124	9	65806164	65950096	Misassembly	NONE	65806164	65950096		0					OTHER		complex event
9-125	9	66567183	66636200	Misassembly	NONE	66567183	66636200		0					OTHER		complex event
9-128	9	67284698	67344902	Misassembly	NONE	67284698	67344902		0					OTHER		complex event
9-130	9	67534539	67752935	Misassembly	NONE	67534539	67752935		0					OTHER		complex event
9-131	9	67851467	68069406	Misassembly	NONE	67851467	68069406		0					OTHER		complex event
9-133	9	68560015	68639558	Misassembly	NONE	68560015	68639558		0					OTHER		complex event

9-137	9	70183003	70219497	Misassembly	NONE	70183003	70219497		0					OTHER		complex event
9-138	9	70279133	70369198	Misassembly	NONE	70279133	70369198		0					OTHER		complex event
9-139	9	70416261	70763633	Misassembly	NONE	70416261	70763633		0					OTHER		complex event
9-141	9	71141124	71206753	Misassembly	NONE	71141124	71206753		0					OTHER		complex event - possible inversion
9-143	9	71486472	71638573	Misassembly	NONE	71486472	71638573		0					OTHER		complex event
9-144	9	71717732	71827740	Misassembly	NONE	71717732	71827740		0					OTHER		complex event
9-146	9	72132256	72319453	Misassembly	NONE	72132256	72319453		0					OTHER		complex event
9-148	9	72839205	72912378	Misassembly	NONE	72839205	72912378		0					OTHER		complex event
9-151	9	73759576	74156823	Misassembly	NONE	73759576	74156823		0					OTHER		complex event
9-152	9	74328703	74501584	Misassembly	NONE	74328703	74501584		0					OTHER		complex event
9-153	9	74836676	75017819	Misassembly	NONE	74836676	75017819		0					OTHER		complex event
9-154	9	75260627	75360278	Misassembly	NONE	75260627	75360278		0					OTHER		complex event
9-156	9	75959211	76040744	Misassembly	NONE	75959211	76040744		0					OTHER		complex event
9-160	9	76580334	76697163	Misassembly	NONE	76580334	76697163		0					OTHER		complex event
9-162	9	76857108	77157494	Misassembly	NONE	76857108	77157494		0					OTHER		complex event
9-163	9	77387151	77740344	Misassembly	NONE	77387151	77740344		0					OTHER		complex event
9-164	9	77861026	78073904	Misassembly	NONE	77861026	78073904		0					OTHER		complex event
9-165	9	78956514	79022524	Misassembly	NONE	78956514	79022524		0					OTHER		complex event
9-169	9	80465753	80569055	Misassembly	NONE	80465753	80569055		0					OTHER		complex event - possible translocation
9-170	9	80729606	80798357	Misassembly	NONE	80729606	80798357		0					OTHER		complex event
9-171	9	80964604	81026755	Misassembly	NONE	80964604	81026755		0					OTHER		complex event
9-173	9	81232111	81448986	Misassembly	NONE	81232111	81448986		0					OTHER		complex event
9-175	9	81703264	81907584	Misassembly	NONE	81703264	81907584		0					OTHER		complex event
9-176	9	82372712	82473945	Misassembly	NONE	82372712	82473945		0					OTHER		complex event
9-177	9	82662545	82935992	Misassembly	NONE	82662545	82935992		0					OTHER		complex event
9-179	9	84706079	85050494	Misassembly	NONE	84706079	85050494		0					OTHER		complex event
9-180	9	85117727	85271090	Misassembly	NONE	85117727	85271090		0					OTHER		complex event
9-181	9	85549795	85605629	Misassembly	NONE	85549795	85605629		0					OTHER		complex event - possible translocation
9-182	9	85740368	85848879	Misassembly	NONE	85740368	85848879		0					OTHER		complex event
9-183	9	85980854	86072499	Misassembly	NONE	85980854	86072499		0					OTHER		complex event
9-184	9	86121691	86326824	Misassembly	NONE	86121691	86326824		0					OTHER		complex event
9-186	9	86481732	86607029	Misassembly	NONE	86481732	86607029		0					OTHER		complex event
9-187	9	86951762	86984810	Misassembly	NONE	86951762	86984810		0					OTHER		complex event - possible inversion
9-188	9	87086831	87282151	Misassembly	NONE	87086831	87282151		0					OTHER		complex event
9-189	9	87467201	87517398	Misassembly	NONE	87467201	87517398		0					OTHER		complex event
9-191	9	87752256	87816328	Misassembly	NONE	87752256	87816328		0					OTHER		complex event
9-193	9	87888329	87915927	Misassembly	NONE	87888329	87915927		0					OTHER		complex event - possible inversion
9-194	9	88028857	88093739	Misassembly	NONE	88028857	88093739		0					OTHER		complex event
9-195	9	88648732	88792650	Misassembly	NONE	88648732	88792650		0					OTHER		complex event
9-196	9	88855538	89499307	Misassembly	NONE	88855538	89499307		0					OTHER		complex event
9-197	9	89499307	89630806	Misassembly	NONE	89499307	89630806		0					OTHER		complex event
9-198	9	89770093	89827736	Misassembly	NONE	89770093	89827736		0					OTHER		complex event
9-201	9	90286372	90314652	Misassembly	NONE	90286372	90314652		0					OTHER		complex event - possible inversion
9-202	9	90528031	90621150	Misassembly	NONE	90528031	90621150		0					OTHER		complex event
9-204	9	91147929	91225939	Misassembly	NONE	91147929	91225939		0					OTHER		complex event
9-205	9	91285573	91403782	Misassembly	NONE	91285573	91403782		0					OTHER		complex event
9-206	9	91606621	91723093	Misassembly	NONE	91606621	91723093		0					OTHER		complex event
9-207	9	91819248	91909852	Misassembly	NONE	91819248	91909852		0					OTHER		complex event
9-208	9	92003594	92119618	Misassembly	NONE	92003594	92119618		0					OTHER		complex event
9-209	9	92191644	92502199	Misassembly	NONE	92191644	92502199		0					OTHER		complex event
9-211	9	92698481	92727690	Misassembly	NONE	92698481	92727690		0					OTHER		complex event
9-212	9	92791449	93022315	Misassembly	NONE	92791449	93022315		0					OTHER		complex event
9-213	9	93292351	93694309	Misassembly	NONE	93292351	93694309		0					OTHER		complex event
9-218	9	94917982	95039784	Misassembly	NONE	94917982	95039784		0					OTHER		complex event
9-221	9	95342217	95399850	Misassembly	NONE	95342217	95399850		0					OTHER		complex event
9-223	9	95823669	95886396	Misassembly	NONE	95823669	95886396		0					OTHER		complex event
9-224	9	95922042	96049150	Misassembly	NONE	95922042	96049150		0					OTHER		complex event
9-226	9	96314115	96379230	Misassembly	NONE	96314115	96379230		0					OTHER		complex event
9-232	9	97489527	97600886	Misassembly	NONE	97489527	97600886		0					OTHER		complex event
9-236	9	98190308	98351193	Misassembly	NONE	98190308	98351193		0					OTHER		complex event
9-237	9	98376736	98471693	Misassembly	NONE	98376736	98471693		0					OTHER		complex event
9-241	9	98983101	99176106	Misassembly	NONE	98983101	99176106		0					OTHER		complex event
9-242	9	99360885	99516481	Misassembly	NONE	99360885	99516481		0					OTHER		complex event
9-243	9	99685600	99886500	Misassembly	NONE	99685600	99886500		0					OTHER		complex event
9-245	9	102195390	102330447	Misassembly	NONE	102195390	102330447		0					OTHER		complex event
9-252	9	109969544	110108809	Misassembly	NONE	109969544	110108809		0					OTHER		complex event - possible inversion
9-253	9	111447671	111496012	Misassembly	NONE	111447671	111496012		0					OTHER		complex event
9-255	9	112693475	112923549	Misassembly	NONE	112693475	112923549		0					OTHER		complex event
9-258	9	113501757	113627025	Misassembly	NONE	113501757	113627025		0					OTHER		complex event
9-259	9	113759197	113812279	Misassembly	NONE	113759197	113812279		0					OTHER		complex event
9-269	9	116947683	117006546	Misassembly	NONE	116947683	117006546		0					OTHER		complex event
9-271	9	119412537	119561710	Misassembly	NONE	119412537	119561710		0					OTHER		complex event
9-273	9	120015733	120097964	Misassembly	NONE	120015733	120097964		0					OTHER		complex event
9-275	9	120704657	120959635	Misassembly	NONE	120704657	120959635		0					OTHER		complex event
9-276	9	123035873	123128739	Misassembly	NONE	123035873	123128739		0					OTHER		complex event
9-278	9	124218980	124408380	Misassembly	NONE	124218980	124408380		0					OTHER		complex event
9-279	9	124605421	124902532	Misassembly	NONE	124605421	124902532		0					OTHER		complex event

9-285	9	132929515	132984439	Misassembly	NONE	132929515	132984439		0					OTHER		complex event
9-286	9	133055754	133139254	Misassembly	NONE	133055754	133139254		0					OTHER		complex event
9-287	9	133280558	133539391	Misassembly	NONE	133280558	133539391		0					OTHER		complex event
9-288	9	133656178	133859528	Misassembly	NONE	133656178	133859528		0					OTHER		complex event
9-290	9	134392409	134484018	Misassembly	NONE	134392409	134484018		0					OTHER		complex event
9-292	9	134681115	135007981	Misassembly	NONE	134681115	135007981		0					OTHER		complex event
9-293	9	135058361	135141527	Misassembly	NONE	135058361	135141527		0					OTHER		complex event
9-295	9	135366115	135545931	Misassembly	NONE	135366115	135545931		0					OTHER		complex event
9-296	9	135717509	135791985	Misassembly	NONE	135717509	135791985		0					OTHER		complex event
9-300	9	136487106	136560788	Misassembly	NONE	136487106	136560788		0					OTHER		complex event
9-301	9	136769763	136880609	Misassembly	NONE	136769763	136880609		0					OTHER		complex event
9-304	9	137197006	137235621	Misassembly	NONE	137197006	137235621		0					OTHER		complex event
9-305	9	137350463	137445185	Misassembly	NONE	137350463	137445185		0					OTHER		complex event
9-306	9	137606890	137848908	Misassembly	NONE	137606890	137848908		0					OTHER		complex event
9-307	9	137908165	138193059	Misassembly	NONE	137908165	138193059		0					OTHER		complex event
9-308	9	138386499	138449363	Misassembly	NONE	138386499	138449363		0					OTHER		complex event
9-311	9	138839364	139031018	Misassembly	NONE	138839364	139031018		0					OTHER		complex event
9-312	9	139058230	139468857	Misassembly	NONE	139058230	139468857		0					OTHER		complex event
9-314	9	139578867	139710182	Misassembly	NONE	139578867	139710182		0					OTHER		complex event
9-315	9	139751602	139856865	Misassembly	NONE	139751602	139856865		0					OTHER		complex event
9-316	9	140407791	140561151	Misassembly	NONE	140407791	140561151		0					OTHER		complex event
9-318	9	141296837	141407535	Misassembly	NONE	141296837	141407535		0					OTHER		complex event
9-320	9	141824047	141878535	Misassembly	NONE	141824047	141878535		0					OTHER		complex event
9-321	9	141957030	142334894	Misassembly	NONE	141957030	142334894		0					OTHER		complex event
9-322	9	142384868	142622569	Misassembly	NONE	142384868	142622569		0					OTHER		complex event
9-323	9	143121427	143230802	Misassembly	NONE	143121427	143230802		0					OTHER		complex event
9-325	9	143670742	143763980	Misassembly	NONE	143670742	143763980		0					OTHER		complex event
9-326	9	143910969	143985788	Misassembly	NONE	143910969	143985788		0					OTHER		complex event
9-329	9	144187670	144386854	Misassembly	NONE	144187670	144386854		0					OTHER		complex event
9-333	9	145158803	145353710	Misassembly	NONE	145158803	145353710		0					OTHER		complex event
9-334	9	145379863	145487643	Misassembly	NONE	145379863	145487643		0					OTHER		complex event
9-335	9	145695079	145806852	Misassembly	NONE	145695079	145806852		0					OTHER		complex event
9-336	9	146043481	146120345	Misassembly	NONE	146043481	146120345		0					OTHER		complex event
9-337	9	146304764	146438079	Misassembly	NONE	146304764	146438079		0					OTHER		complex event
9-338	9	146544109	146598431	Misassembly	NONE	146544109	146598431		0					OTHER		complex event
9-340	9	146805776	146862748	Misassembly	NONE	146805776	146862748		0					OTHER		complex event
9-342	9	147416641	147667260	Misassembly	NONE	147416641	147667260		0					OTHER		complex event
9-343	9	147770691	148065582	Misassembly	NONE	147770691	148065582		0					OTHER		complex event
9-344	9	148473497	148906274	Misassembly	NONE	148473497	148906274		0					OTHER		complex event
9-345	9	149311753	149477093	Misassembly	NONE	149311753	149477093		0					OTHER		complex event
9-346	9	149831741	149994099	Misassembly	NONE	149831741	149994099		0					OTHER		complex event
9-349	9	150414117	150546560	Misassembly	NONE	150414117	150546560		0					OTHER		complex event
10-1	10	307952	346182	Misassembly	NONE	307952	346182		0					OTHER		complex event
10-7	10	996489	1468531	Misassembly	NONE	996489	1468531		0					OTHER		complex event
10-8	10	1722863	1894356	Misassembly	NONE	1722863	1894356		0					OTHER		complex event
10-9	10	2163422	2458511	Misassembly	NONE	2163422	2458511		0					OTHER		complex event
10-10	10	2458511	2735099	Misassembly	NONE	2458511	2735099		0					OTHER		complex event
10-12	10	3137189	3198447	Misassembly	NONE	3137189	3198447		0					OTHER		complex event
10-13	10	3367150	3549507	Misassembly	NONE	3367150	3549507		0					OTHER		complex event
10-14	10	3593484	3845811	Misassembly	NONE	3593484	3845811		0					OTHER		complex event
10-18	10	8276974	8362125	Misassembly	NONE	8276974	8362125		0					OTHER		complex event
10-19	10	8403918	8585805	Misassembly	NONE	8403918	8585805		0					OTHER		complex event
10-20	10	8965512	9073015	Misassembly	NONE	8965512	9073015		0					OTHER		complex event
10-21	10	9142353	9341981	Misassembly	NONE	9142353	9341981		0					OTHER		complex event
10-23	10	9527410	9582753	Misassembly	NONE	9527410	9582753		0					OTHER		compex event
10-26	10	10030758	10224510	Misassembly	NONE	10030758	10224510		0					OTHER		complex event
10-28	10	10393710	10436650	Misassembly	NONE	10393710	10436650		0					OTHER		complex event
10-31	10	15507279	15543751	Misassembly	NONE	15507279	15543751		0					OTHER		complex event
10-33	10	15656544	16135726	Misassembly	NONE	15656544	16135726		0					OTHER		complex event
10-36	10	16657318	16740724	Misassembly	NONE	16657318	16740724		0					OTHER		complex event
10-37	10	16834742	16911909	Misassembly	NONE	16834742	16911909		0					OTHER		complex event
10-38	10	17051280	17107847	Misassembly	NONE	17051280	17107847		0					OTHER		complex event - possible inversion
10-40	10	17195554	17276036	Misassembly	NONE	17195554	17276036		0					OTHER		complex event
10-42	10	18456599	18495050	Misassembly	NONE	18456599	18495050		0					OTHER		complex event
10-44	10	18642036	18745630	Misassembly	NONE	18642036	18745630		0					OTHER		complex event
10-46	10	18984995	19050423	Misassembly	NONE	18984995	19050423		0					OTHER		complex event
10-48	10	19180716	19232469	Misassembly	NONE	19180716	19232469		0					OTHER		complex event - possible deletion
10-49	10	23131633	23225626	Misassembly	NONE	23131633	23225626		0					OTHER		complex event
10-51	10	23679142	23950047	Misassembly	NONE	23679142	23950047		0					OTHER		complex event
10-54	10	25189125	25560653	Misassembly	NONE	25189125	25560653		0					OTHER		complex event
10-56	10	25923999	26100613	Misassembly	NONE	25923999	26100613		0					OTHER		complex event
10-57	10	26313495	26341381	Misassembly	NONE	26313495	26341381		0					OTHER		complex event
10-59	10	28383041	28853832	Misassembly	NONE	28383041	28853832		0					OTHER		complex event
10-60	10	29443662	29512177	Misassembly	NONE	29443662	29512177		0					OTHER		complex event
10-61	10	29568715	29592886	Misassembly	NONE	29568715	29592886		0					OTHER		complex event
10-62	10	29630067	29937741	Misassembly	NONE	29630067	29937741		0					OTHER		complex event
10-65	10	30327519	30449156	Misassembly	NONE	30327519	30449156		0					OTHER		complex event
10-66	10	30669132	30726513	Misassembly	NONE	30669132	30726513		0					OTHER		complex event
10-67	10	30836736	30950292	Misassembly	NONE	30836736	30950292		0					OTHER		complex event
10-68	10	30990973	31045003	Misassembly	NONE	30990973	31045003		0					OTHER		complex event
10-69	10	31196365	31450568	Misassembly	NONE	31196365	31450568		0					OTHER		complex event
10-70	10	31609790	31656694	Misassembly	NONE	31609790	31656694		0					OTHER		complex event - possible inversion
10-72	10	32125893	32275434	Misassembly	NONE	32125893	32275434		0					OTHER		complex event

10-73	10	33931462	34057452	Misassembly	NONE	33931462	34057452		0					OTHER		complex event - possible translocation
10-74	10	34185293	34334527	Misassembly	NONE	34185293	34334527		0					OTHER		complex event
10-78	10	40683569	40761763	Misassembly	NONE	40683569	40761763		0					OTHER		complex event
10-79	10	40863977	41074022	Misassembly	NONE	40863977	41074022		0					OTHER		complex event
10-80	10	41245374	41334848	Misassembly	NONE	41245374	41334848		0					OTHER		complex event
10-81	10	41625734	41737289	Misassembly	NONE	41625734	41737289		0					OTHER		complex event
10-83	10	42032923	42231728	Misassembly	NONE	42032923	42231728		0					OTHER		complex event
10-85	10	42398520	42471183	Misassembly	NONE	42398520	42471183		0					OTHER		complex event
10-87	10	45256115	45351504	Misassembly	NONE	45256115	45351504		0					OTHER		complex event
10-88	10	45545943	45593784	Misassembly	NONE	45545943	45593784		0					OTHER		complex event
10-89	10	45798555	45884029	Misassembly	NONE	45798555	45884029		0					OTHER		complex event - possible insertion
10-90	10	46071497	46131863	Misassembly	NONE	46071497	46131863		0					OTHER		complex event
10-95	10	46844784	46912646	Misassembly	NONE	46844784	46912646		0					OTHER		complex event - possible inversion
10-96	10	47360639	47448890	Misassembly	NONE	47360639	47448890		0					OTHER		complex event - possible inversion
10-98	10	47928873	48110920	Misassembly	NONE	47928873	48110920		0					OTHER		complex event
10-99	10	48110920	48625940	Misassembly	NONE	48110920	48625940		0					OTHER		complex event
10-100	10	48688151	48728924	Misassembly	NONE	48688151	48728924		0					OTHER		complex event - possible inversion
10-101	10	51314244	51559150	Misassembly	NONE	51314244	51559150		0					OTHER		complex event
10-102	10	51721097	51779483	Misassembly	NONE	51721097	51779483		0					OTHER		complex event - possible inversion
10-103	10	51813427	51926872	Misassembly	NONE	51813427	51926872		0					OTHER		complex event
10-111	10	54230073	54501854	Misassembly	NONE	54230073	54501854		0					OTHER		complex event
10-112	10	54756676	54918883	Misassembly	NONE	54756676	54918883		0					OTHER		complex event
10-113	10	54966864	55109428	Misassembly	NONE	54966864	55109428		0					OTHER		complex event - possible inversion
10-114	10	55265843	55410199	Misassembly	NONE	55265843	55410199		0					OTHER		complex event
10-115	10	55477757	55589065	Misassembly	NONE	55477757	55589065		0					OTHER		complex event
10-119	10	56770244	56912847	Misassembly	NONE	56770244	56912847		0					OTHER		complex event
10-120	10	57084867	57232814	Misassembly	NONE	57084867	57232814		0					OTHER		complex event
10-121	10	57843691	58208215	Misassembly	NONE	57843691	58208215		0					OTHER		complex event
10-122	10	58287663	58542763	Misassembly	NONE	58287663	58542763		0					OTHER		complex event
10-124	10	59017324	59086611	Misassembly	NONE	59017324	59086611		0					OTHER		complex event
10-129	10	61107959	61190175	Misassembly	NONE	61107959	61190175		0					OTHER		complex event
10-130	10	61210336	61292263	Misassembly	NONE	61210336	61292263		0					OTHER		complex event
10-132	10	61607704	61630953	Misassembly	NONE	61607704	61630953		0					OTHER		complex event
10-133	10	61752440	61844538	Misassembly	NONE	61752440	61844538		0					OTHER		complex event
10-134	10	62169203	62273028	Misassembly	NONE	62169203	62273028		0					OTHER		complex event
10-135	10	62328800	62400418	Misassembly	NONE	62328800	62400418		0					OTHER		complex event - possible inversion
10-137	10	62609807	62670649	Misassembly	NONE	62609807	62670649		0					OTHER		complex event
10-139	10	67910529	68000794	Misassembly	NONE	67910529	68000794		0					OTHER		complex event
10-140	10	68262845	68455627	Misassembly	NONE	68262845	68455627		0					OTHER		complex event
10-144	10	69373016	69459989	Misassembly	NONE	69373016	69459989		0					OTHER		complex event
10-146	10	69671313	69826990	Misassembly	NONE	69671313	69826990		0					OTHER		complex event
10-149	10	71571045	71623855	Misassembly	NONE	71571045	71623855		0					OTHER		complex event
10-150	10	72058272	72138991	Misassembly	NONE	72058272	72138991		0					OTHER		complex event - possible inversion
10-151	10	72205770	72264232	Misassembly	NONE	72205770	72264232		0					OTHER		complex event
10-153	10	72792519	72911300	Misassembly	NONE	72792519	72911300		0					OTHER		complex event
10-155	10	73276597	73351903	Misassembly	NONE	73276597	73351903		0					OTHER		complex event
10-156	10	73414193	73684485	Misassembly	NONE	73414193	73684485		0					OTHER		complex event
10-160	10	74499238	74592089	Misassembly	NONE	74499238	74592089		0					OTHER		complex event
10-166	10	75872438	75962596	Misassembly	NONE	75872438	75962596		0					OTHER		complex event
10-167	10	76096580	76148646	Misassembly	NONE	76096580	76148646		0					OTHER		complex event
10-169	10	76621177	76655428	Misassembly	NONE	76621177	76655428		0					OTHER		complex event
10-170	10	76783451	76819309	Misassembly	NONE	76783451	76819309		0					OTHER		complex event
10-176	10	79539151	79653389	Misassembly	NONE	79539151	79653389		0					OTHER		complex event
10-181	10	83030653	83076438	Misassembly	NONE	83030653	83076438		0					OTHER		complex event
10-182	10	83155352	83237982	Misassembly	NONE	83155352	83237982		0					OTHER		complex event
10-183	10	83350762	83526394	Misassembly	NONE	83350762	83526394		0					OTHER		
10-190	10	86465518	86568988	Misassembly	NONE	86465518	86568988		0					OTHER		complex event
10-192	10	86730854	86859936	Misassembly	NONE	86730854	86859936		0					OTHER		complex event
10-195	10	87338101	87568468	Misassembly	NONE	87338101	87568468		0					OTHER		complex event
10-198	10	91998421	92048916	Misassembly	NONE	91998421	92048916		0					OTHER		complex event
10-200	10	92280688	92366237	Misassembly	NONE	92280688	92366237		0					OTHER		complex event
10-203	10	92888624	93064459	Misassembly	NONE	92888624	93064459		0					OTHER		complex event
10-208	10	97856213	98003015	Misassembly	NONE	97856213	98003015		0					OTHER		complex event
10-210	10	98446537	98555651	Misassembly	NONE	98446537	98555651		0					OTHER		complex event
10-213	10	101601915	101720961	Misassembly	NONE	101601915	101720961		0					OTHER		complex event
10-215	10	102386925	102592166	Misassembly	NONE	102386925	102592166		0					OTHER		complex event
10-216	10	102883245	103114669	Misassembly	NONE	102883245	103114669		0					OTHER		complex event
10-220	10	103685788	103807649	Misassembly	NONE	103685788	103807649		0					OTHER		complex event
10-222	10	104141804	104204446	Misassembly	NONE	104141804	104204446		0					OTHER		complex event
10-223	10	104340763	104551995	Misassembly	NONE	104340763	104551995		0					OTHER		complex event
10-229	10	105565675	105631898	Misassembly	NONE	105565675	105631898		0					OTHER		complex event
10-232	10	106470287	106728768	Misassembly	NONE	106470287	106728768		0					OTHER		complex event
10-237	10	107384061	107493729	Misassembly	NONE	107384061	107493729		0					OTHER		complex event

10-240	10	108145530	108247989	Misassembly	NONE	108145530	108247989		0					OTHER		complex event
10-244	10	108456033	108614170	Misassembly	NONE	108456033	108614170		0					OTHER		complex event
10-245	10	108703960	108740408	Misassembly	NONE	108703960	108740408		0					OTHER		complex event
10-246	10	108904594	109191387	Misassembly	NONE	108904594	109191387		0					OTHER		complex event
10-247	10	109274655	109336942	Misassembly	NONE	109274655	109336942		0					OTHER		complex event - possible inversion
10-248	10	109426918	109486737	Misassembly	NONE	109426918	109486737		0					OTHER		complex event - possible inversion
10-250	10	109755240	109791940	Misassembly	NONE	109755240	109791940		0					OTHER		complex event
10-251	10	109814900	109915263	Misassembly	NONE	109814900	109915263		0					OTHER		complex event
10-259	10	111948038	112119243	Misassembly	NONE	111948038	112119243		0					OTHER		complex event
10-261	10	112220085	112347158	Misassembly	NONE	112220085	112347158		0					OTHER		complex event
10-262	10	112443834	112552696	Misassembly	NONE	112443834	112552696		0					OTHER		complex event
10-266	10	112928266	113014218	Misassembly	NONE	112928266	113014218		0					OTHER		complex event
10-267	10	113112970	113209502	Misassembly	NONE	113112970	113209502		0					OTHER		complex event
10-268	10	113382233	113394669	Misassembly	NONE	113382233	113394669		0					OTHER		complex event
10-273	10	114199372	114250980	Misassembly	NONE	114199372	114250980		0					OTHER		complex event
10-274	10	114314590	114412501	Misassembly	NONE	114314590	114412501		0					OTHER		complex event
10-275	10	114600865	114706824	Misassembly	NONE	114600865	114706824		0					OTHER		complex event
10-278	10	114861558	114925311	Misassembly	NONE	114861558	114925311		0					OTHER		complex event
10-280	10	115484138	115830565	Misassembly	NONE	115484138	115830565		0					OTHER		complex event
10-281	10	115961621	116077310	Misassembly	NONE	115961621	116077310		0					OTHER		complex event
10-285	10	116675847	116716465	Misassembly	NONE	116675847	116716465		0					OTHER		complex event - possible inversion
10-288	10	117098471	117216827	Misassembly	NONE	117098471	117216827		0					OTHER		complex event
10-292	10	117833705	117886791	Misassembly	NONE	117833705	117886791		0					OTHER		complex event
10-296	10	118696611	119006146	Misassembly	NONE	118696611	119006146		0					OTHER		complex event
10-297	10	119217437	119268535	Misassembly	NONE	119217437	119268535		0					OTHER		complex event
10-299	10	119931472	120135215	Misassembly	NONE	119931472	120135215		0					OTHER		complex event
10-301	10	124277267	124367994	Misassembly	NONE	124277267	124367994		0					OTHER		complex event
10-302	10	124555054	124734012	Misassembly	NONE	124555054	124734012		0					OTHER		complex event
10-304	10	125041743	125118494	Misassembly	NONE	125041743	125118494		0					OTHER		complex event
10-309	10	127567314	127851168	Misassembly	NONE	127567314	127851168		0					OTHER		complex event
10-310	10	130397158	130503577	Misassembly	NONE	130397158	130503577		0					OTHER		complex event - possible translocation
10-311	10	130583734	130644542	Misassembly	NONE	130583734	130644542		0					OTHER		complex event - possible inversion
10-318	10	137344609	137442036	Misassembly	NONE	137344609	137442036		0					OTHER		complex event
10-320	10	141835158	142005167	Misassembly	NONE	141835158	142005167		0					OTHER		complex event
10-323	10	142384702	142424478	Misassembly	NONE	142384702	142424478		0					OTHER		complex event
10-326	10	142865914	142947166	Misassembly	NONE	142865914	142947166		0					OTHER		complex event
10-327	10	148503687	148646617	Misassembly	NONE	148503687	148646617		0					OTHER		complex event

Note: EC = Extra Cut, MC= Missing Cut, INS = Insertion, DEL = Deletion, and OTHER = Multiple Events
