# Supplementary material for: A Single Molecule Scaffold for the Maize Genome
Source: PLoS Genet. 2009 Nov 20;5(11):e1000711. doi: 10.1371/journal.pgen.1000711 (PMC2774507; doi:10.1371/journal.pgen.1000711)
Supplement: Table S3 — FPC gap estimations based on map alignments between optical maps and the in silico maps of FPC contig sequence pseudomolecules and B73 RefGen_v1 reference chromosomes. (0.12 MB PDF) [file pgen.1000711.s004.pdf]

FPC gap estimations based on map alignments between optical maps and the *in silico* maps of FPC contig sequence pseudomolecules and B73 RefGen_v1 reference chromosomes

	FPC Contigs on AGP Pseudomolecule			Aligned Map Segment on AGP Pseudomolecule		Aligned Map Segment on Optical Map				
Chr. No.	Contig A	Contig B	Aligned Optical Map Contig	Left pseudomolecule coordinate (bp)	Right pseudomolecule coordinate (bp)	Left optical coordinate (bp)	Right optical coordinate (bp)	Gap size (kb)	Difference (%)	Comments
1	ctg1	ctg2	OMcontig_15	2,296,436	2,340,298	47,492,540	47,452,820	2.36	5.38	
1	ctg2	ctg3	OMcontig_15	3,158,020	4,932,978	46,639,860	44,897,400	-32.50	1.83	
1	ctg3	ctg4	OMcontig_15	5,701,470	7,069,304	44,201,740	42,869,790	-35.88	2.62	
1	ctg4	ctg5	OMcontig_15	8,363,233	10,767,316	41,729,520	39,198,190	127.25	5.29	
1	ctg5	ctg6	OMcontig_15	13,072,492	13,091,007	36,866,810	36,723,500	124.80	674.02	
1	ctg8	ctg9	OMcontig_15	23,361,536	24,061,519	26,918,510	26,330,930	-112.40	16.06	
1	ctg9	ctg10	OMcontig_15	29,507,634	30,159,601	21,014,080	20,408,450	-46.34	7.11	
1	ctg10	ctg12	OMcontig_15	47,372,113	48,929,557	3,763,240	2,162,490	43.31	2.78	
1	ctg14	ctg16	OMcontig_3	64,946,214	65,771,847	70,918,130	68,003,030	2089.47	253.07	
1	ctg16	ctg474	OMcontig_3	67,086,454	67,932,233	66,684,270	68,274,340	see comment		ctg474 is inconsistent with optical map
1	ctg474	ctg17	OMcontig_3	68,290,142	68,474,832	68,603,720	65,900,220	see comment		ctg474 is inconsistent with optical map
1	ctg17	ctg18	OMcontig_3	70,009,936	71,051,557	64,370,290	63,255,930	72.74	6.98	
1	ctg18	ctg19	OMcontig_3	73,505,043	73,719,210	60,781,400	60,545,540	21.69	10.13	
1	ctg19	ctg20	OMcontig_3	78,226,617	78,371,392	55,998,200	55,618,020	235.41	162.60	
1	ctg20	ctg22	OMcontig_3	84,549,950	84,598,481	49,548,420	49,355,580	144.31	297.35	
1	ctg22	ctg24	OMcontig_3	85,910,394	87,407,337	48,129,210	42,067,180	see comment		
1	ctg24	ctg23	OMcontig_3	90,006,601	90,254,799	39,455,040	47,310,750	see comment		ctg23 is inconsistent with optical map
1	ctg23	ctg26	OMcontig_3	94,205,616	95,014,665	43,587,330	38,881,040	see comment		ctg23 is inconsistent with optical map
1	ctg26	ctg27	OMcontig_3	100,685,532	100,806,270	33,259,950	32,872,340	266.87	221.03	
1	ctg27	ctg28	OMcontig_3	107,622,378	107,631,375	26,333,660	26,269,730	54.93	610.57	
1	ctg28	ctg29	OMcontig_3	116,091,195	116,647,876	17,893,820	17,275,340	61.80	11.10	
1	ctg29	ctg471	OMcontig_3	123,417,449	123,640,410	10,528,810	10,018,740	287.11	128.77	
1	ctg471	ctg433	OMcontig_3	125,926,280	126,016,224	7,908,150	7,651,360	166.85	185.50	
1	ctg433	ctg437	OMcontig_3	131,169,897	131,182,847	2,626,860	2,585,350	28.56	220.54	
1	ctg437	ctg445	OMcontig_0/3					see comment		ctg445 is inconsistent with optical map
1	ctg445	ctg31	OMcontig_3/0					see comment		ctg445 is inconsistent with optical map
1	ctg31	ctg32	OMcontig_0	154,886,776	155,128,719	83,629,080	83,375,750	11.39	4.71	
1	ctg32	ctg480	OMcontig_0	160,166,630	160,535,909	78,394,270	78,031,930	-6.94	1.88	
1	ctg480	ctg33	OMcontig_0	160,961,489	161,491,607	77,609,870	76,801,920	277.83	52.41	
1	ctg33	ctg36	OMcontig_0	168,713,351	168,857,990	69,536,570	69,335,470	56.46	39.04	
1	ctg36	ctg37	OMcontig_0	177,382,595	177,824,910	60,865,480	60,351,220	71.95	16.27	
1	ctg37	ctg15	OMcontig_0	183,618,165	183,665,204	54,653,140	54,596,700	9.40	19.99	
1	ctg15	ctg38	OMcontig_0	184,203,771	184,710,610	54,012,150	53,496,370	8.94	1.76	
1	ctg38	ctg39	OMcontig_0	190,118,999	190,873,890	48,183,660	47,406,170	22.60	2.99	
1	ctg39	ctg40	OMcontig_0	192,428,206	192,466,073	45,824,670	45,717,850	68.95	182.09	
1	ctg40	ctg41	OMcontig_0	194,251,740	194,287,472	43,940,870	43,911,150	-6.01	16.83	
1	ctg41	ctg42	OMcontig_0	202,447,503	202,615,135	35,837,060	35,033,030	636.40	379.64	
1	ctg42	ctg43	OMcontig_0	204,027,614	204,173,298	33,610,600	33,473,130	-8.21	5.64	inversion
1	ctg43	ctg44	OMcontig_0	207,346,383	207,439,651	30,327,730	30,127,830	106.63	114.33	
1	ctg44	ctg45	OMcontig_0	221,151,978	221,310,128	16,427,940	15,951,600	318.19	201.20	
1	ctg45	ctg492	OMcontig_0					see comment		ctg492 was inverted
1	ctg492	ctg46	OMcontig_0					see comment		ctg492 was inverted
1	ctg46	ctg48	OMcontig_0	233,762,652	234,533,045	3,148,900	2,558,290	-179.78	23.34	
1	ctg52	ctg51	OMcontig_19					see comment		ctg52 was inverted
1	ctg51	ctg54	OMcontig_19	253,775,112	254,020,733	5,240,610	5,487,970	1.74	0.71	
1	ctg54	ctg56	OMcontig_19	258,082,862	260,196,536	9,570,280	11,786,800	102.85	4.87	
1	ctg56	ctg59	OMcontig_19	278,375,203	278,390,617	29,896,490	30,015,660	103.76	673.13	
1	ctg59	ctg60	OMcontig_19	278,589,705	278,689,484	30,213,350	30,374,860	61.73	61.87	
1	ctg60	ctg61	OMcontig_19	279,359,325	280,928,008	30,980,910	32,518,500	-31.09	1.98	

1	ctg61	ctg62	OMcontig_19	281,863,348	282,937,292	33,461,360	34,622,340	87.04	8.10	
1	ctg62	ctg63	OMcontig_19	286,793,203	286,851,441	38,473,460	38,549,590	17.89	30.72	
1	ctg63	ctg67	OMcontig_61	297,479,462	298,811,031	2,948,030	1,491,980	124.48	9.35	
2	ctg68	ctg69	OMcontig_2	2,092,811	4,357,312	1,907,080	4,102,580	-69.00	3.05	
2	ctg69	ctg70	OMcontig_2	6,262,688	8,201,318	5,962,440	8,156,490	255.42	13.18	
2	ctg70	ctg71	OMcontig_2	10,663,599	11,735,460	10,582,570	11,738,630	84.20	7.86	
2	ctg71	ctg72	OMcontig_2	13,973,666	14,183,700	13,968,430	14,326,050	147.59	70.27	
2	ctg72	ctg74	OMcontig_2	16,428,574	16,686,014	16,462,520	17,503,250	783.29	304.26	
2	ctg76	ctg77	OMcontig_2	34,002,462	34,756,082	34,130,320	34,909,750	25.81	3.42	
2	ctg77	ctg78	OMcontig_2	40,957,334	41,371,378	41,063,030	41,604,130	127.06	30.69	
2	ctg78	ctg79	OMcontig_2	49,755,442	50,255,967	50,111,630	51,009,590	397.44	79.40	
2	ctg79	ctg80	OMcontig_2	56,923,859	57,496,341	57,605,180	58,281,560	103.90	18.15	
2	ctg80	ctg81	OMcontig_2	63,234,086	63,340,766	64,005,800	64,316,280	203.80	191.04	
2	ctg81	ctg451	OMcontig_2	65,242,695	65,401,186	66,149,390	66,340,140	32.26	20.35	
2	ctg451	ctg82	OMcontig_2	67,693,641	67,839,845	68,607,750	68,744,870	-9.08	6.21	
2	ctg82	ctg302	OMcontig_2	78,670,254	78,823,152	79,625,990	79,785,450	6.56	4.29	
2	ctg302	ctg83	OMcontig_2	80,821,918	80,832,731	81,767,660	81,781,920	3.45	31.88	
2	ctg83	ctg85	OMcontig_2	82,441,603	82,490,012	83,324,160	84,320,440	947.87	1958.05	
2	ctg85	ctg87	OMcontig_2	83,163,664	83,180,187	85,034,480	87,701,310	2650.31	16040.11	
2	ctg449	ctg86	OMcontig_41	94,427,027	94,770,430	9,993,490	9,652,900	-2.81	0.82	
2	ctg86	ctg426	OMcontig_5/2					see comment		ctg426 is inconsistent with optical map
2	ctg426	ctg467	OMcontig_2/5					see comment		ctg426 is inconsistent with optical map
2	ctg467	ctg89	OMcontig_5	115,145,811	115,449,469	10,016,440	10,341,390	21.29	7.01	
2	ctg89	ctg90	OMcontig_5	133,784,864	133,911,579	28,325,120	28,490,320	38.49	30.37	
2	ctg90	ctg92	OMcontig_5	168,521,694	168,605,913	63,135,180	63,254,610	35.21	41.81	
2	ctg92	ctg88	OMcontig_5	172,156,479	172,496,182	66,783,310	67,414,490	291.48	85.80	
2	ctg88	ctg95	OMcontig_5	172,569,243	172,766,384	67,483,690	67,695,090	14.26	7.23	
2	ctg95	ctg96	OMcontig_5	174,242,688	174,485,114	69,234,940	69,589,120	111.75	46.10	
2	ctg96	ctg97	OMcontig_5	180,479,460	180,649,665	75,543,180	75,715,990	2.61	1.53	
2	ctg97	ctg98	OMcontig_5	181,368,840	181,584,567	76,439,910	76,695,330	39.69	18.40	
2	ctg99	ctg100	OMcontig_26	189,880,662	190,476,307	24,973,810	24,287,740	90.43	15.18	
2	ctg103	ctg104	OMcontig_26	201,147,049	202,798,345	13,196,570	11,325,120	220.15	13.33	
2	ctg105	ctg107	OMcontig_66	216,668,720	216,860,391	1,518,681	1,345,161	-18.15	9.47	
2	ctg108	ctg109	OMcontig_39	228,006,919	228,212,397	6,222,470	6,172,360	-155.37	75.61	
2	ctg109	ctg110	OMcontig_39	230,963,162	232,984,713	3,446,940	1,152,587	272.80	13.49	
3	ctg111	ctg112	OMcontig_13	7,855,691	9,680,999	47,231,330	45,363,460	42.56	2.33	
3	ctg112	ctg113	OMcontig_13	11,724,869	11,800,886	43,308,690	43,221,510	11.16	14.68	
3	ctg113	ctg115	OMcontig_13	18,586,901	18,632,798	36,400,060	36,349,210	4.95	10.79	
3	ctg115	ctg117	OMcontig_13	27,119,752	27,525,107	27,898,010	27,445,420	47.24	11.65	
3	ctg117	ctg118	OMcontig_13	46,471,424	46,495,468	8,893,650	8,839,840	29.77	123.80	
3	ctg118	ctg119	OMcontig_46	55,889,527	56,330,899	1,508,570	1,956,150	6.21	1.41	
3	ctg119	ctg120	OMcontig_46	63,742,207	63,751,015	9,811,740	9,845,790	25.24	286.58	
3	ctg255	ctg121	OMcontig_28					see comment		ctg121 was inverted, and both ctg255 and ctg121 probably should be moved to chr5
3	ctg121	ctg122	OMcontig_12	109,416,224	109,902,009	53,563,907	53,127,047	-48.93	10.07	
3	ctg122	ctg124	OMcontig_12	121,222,467	121,293,078	41,948,057	41,883,347	-5.90	8.36	
3	ctg124	ctg126	OMcontig_12	137,449,147	137,504,424	25,733,657	25,603,757	74.62	135.00	
3	ctg126	ctg128	OMcontig_12	143,318,664	143,423,711	19,669,747	19,409,567	155.13	147.68	
3	ctg128	ctg129	OMcontig_12	147,632,217	149,220,626	15,126,577	13,521,757	16.41	1.03	
3	ctg129	ctg130	OMcontig_12/2					see comment		ctg130 is inconsistent with optical map and it should be placed on chr2
3	ctg130	ctg131	OMcontig_2/12					see comment		ctg130 is inconsistent with optical map and it should be placed on chr2
3	ctg131	ctg134	OMcontig_11	172,616,049	172,660,261	46,478,220	46,424,270	9.74	22.03	
3	ctg134	ctg135	OMcontig_11	175,045,593	175,128,780	43,846,040	43,601,470	161.38	194.00	
3	ctg135	ctg136	OMcontig_11	177,119,332	177,735,296	41,557,850	40,892,550	49.34	8.01	
3	ctg136	ctg138	OMcontig_11	179,389,429	181,383,820	39,222,320	37,252,730	-24.80	1.24	
3	ctg138	ctg140	OMcontig_11	190,017,142	193,712,706	28,733,280	25,044,140	-6.42	0.17	
3	ctg140	ctg141	OMcontig_11	194,481,539	194,558,718	24,315,740	24,150,500	88.06	114.10	

3	ctg141	ctg142	OMcontig_11	198,101,045	199,164,107	20,629,640	19,516,210	50.37	4.74	
3	ctg142	ctg143	OMcontig_11	200,703,948	200,732,449	18,010,570	17,926,740	55.33	194.13	
3	ctg143	ctg144	OMcontig_11	202,501,675	202,650,104	16,231,510	16,058,910	24.17	16.28	
3	ctg144	ctg145	OMcontig_11	202,961,735	204,383,362	15,753,910	14,252,850	79.43	5.59	
3	ctg145	ctg147	OMcontig_11	212,610,737	212,676,410	6,271,200	6,113,770	91.76	139.72	
3	ctg147	ctg149	OMcontig_11	215,513,986	215,565,584	3,470,030	3,392,080	26.35	51.07	
3	ctg149	ctg150	OMcontig_47	219,143,241	219,189,266	932,960	1,005,530	26.55	57.68	
3	ctg150	ctg151	OMcontig_47	224,179,631	224,643,809	5,993,210	6,539,160	81.77	17.62	
3	ctg151	ctg152	OMcontig_47	228,911,061	229,019,615	10,649,220	11,164,160	406.39	374.36	
4	ctg156	ctg157	OMcontig_44	11,195,836	11,299,399	5,579,280	5,432,920	42.80	41.32	
4	ctg158	ctg159	OMcontig_44	14,080,312	14,627,603	2,791,010	1,846,670	397.05	72.55	
4	ctg160	ctg531	OMcontig_24	21,284,969	22,765,583	5,477,060	7,104,460	146.79	9.91	
4	ctg531	ctg162	OMcontig_24	23,498,024	25,165,597	7,835,250	9,586,050	83.23	4.99	
4	ctg162	ctg163	OMcontig_24	25,939,539	27,207,385	10,352,710	11,542,650	-77.91	6.14	
4	ctg163	ctg164	OMcontig_24	31,295,087	32,122,023	15,534,980	16,351,830	-10.09	1.22	
4	ctg435	ctg167	OMcontig_9/40					see comment		ctg435 is inconsistent with optical map
4	ctg190	ctg165	OMcontig_9	62,689,571	62,771,817	52,379,546	52,274,276	23.02	27.99	
4	ctg165	ctg166	OMcontig_9	69,370,536	69,443,116	45,661,166	45,580,766	7.82	10.77	
4	ctg166	ctg172	OMcontig_9	74,962,898	75,024,205	40,059,166	39,940,106	57.75	94.20	
4	ctg172	ctg174	OMcontig_9	84,910,852	85,235,127	30,016,816	29,653,356	39.19	12.08	
4	ctg174	ctg168	OMcontig_9	88,230,589	88,269,671	26,660,406	26,508,836	112.49	287.83	
4	ctg168	ctg444	OMcontig_9	90,959,045	91,169,866	23,766,706	23,495,416	60.47	28.68	
4	ctg444	ctg169	OMcontig_9	92,389,508	92,519,189	22,224,696	22,007,606	87.41	67.40	
4	ctg170	ctg171	OMcontig_29	112,830,572	112,979,193	9,244,360	9,437,260	44.28	29.79	
4	ctg173	ctg175	OMcontig_1	131,026,444	132,034,586	93,873,480	93,002,190	-136.85	13.57	
4	ctg175	ctg176	OMcontig_1	132,258,626	133,494,003	92,781,080	91,644,620	-98.92	8.01	
4	ctg176	ctg179	OMcontig_1	140,847,675	140,927,017	84,435,550	84,307,370	48.84	61.55	
4	ctg179	ctg246	OMcontig_1	148,569,067	148,717,110	76,766,490	76,561,190	57.26	38.68	
4	ctg246	ctg181	OMcontig_1	151,072,530	151,206,230	74,142,200	74,057,890	-49.39	36.94	
4	ctg181	ctg182	OMcontig_1	158,772,110	158,932,700	66,797,000	66,617,040	19.37	12.06	
4	ctg182	ctg184	OMcontig_1	180,895,632	181,328,361	45,190,480	44,342,740	415.01	95.91	
4	ctg184	ctg183	OMcontig_1/9					see comment		ctg183 is inconsistent with optical map, and should be moved to between ctg169 and ctg171
4	ctg183	ctg185	OMcontig_9/1					see comment		ctg183 is inconsistent with optical map, and should be moved to between ctg169 and ctg171
4	ctg185	ctg187	OMcontig_1	193,466,648	194,077,875	38,870,700	37,601,190	658.28	107.70	containing two FPC gaps
4	ctg187	ctg188	OMcontig_1	195,703,159	198,132,364	36,016,580	33,621,140	-33.77	1.39	
4	ctg188	ctg191	OMcontig_1	204,545,880	204,676,170	27,308,490	27,171,010	7.19	5.52	
4	ctg191	ctg194	OMcontig_1	205,875,738	205,926,049	25,966,980	25,840,980	75.69	150.44	
4	ctg194	ctg193	OMcontig_1	208,397,832	208,495,946	23,467,420	23,276,290	93.02	94.80	
4	ctg193	ctg469	OMcontig_1	214,501,403	214,864,319	17,195,900	16,813,060	19.92	5.49	
4	ctg469	ctg192	OMcontig_1	216,994,211	217,063,033	14,547,480	13,951,200	527.46	766.41	
4	ctg192	ctg195	OMcontig_1	218,424,730	218,555,943	12,638,860	12,473,950	33.70	25.68	
4	ctg195	ctg127	OMcontig_1	220,116,118	220,589,622	10,953,410	10,449,110	30.80	6.50	
4	ctg127	ctg196	OMcontig_1	224,516,935	224,599,894	6,492,450	6,310,330	99.16	119.53	
4	ctg199	ctg198	OMcontig_38	236,512,282	236,625,418	6,131,320	6,285,160	40.70	35.98	
4	ctg198	ctg200	OMcontig_38	238,426,899	238,434,708	8,084,860	8,106,070	13.40	171.61	
4	ctg200	ctg201	OMcontig_38	240,959,668	241,014,588	10,628,400	10,712,290	28.97	52.75	
4	ctg201	ctg202	OMcontig_38	244,050,843	244,075,845	13,775,720	13,852,420	51.70	206.78	
4	ctg202	ctg203	OMcontig_38	245,685,047	246,033,540	15,432,800	15,880,400	99.11	28.44	
5	ctg204	ctg205	OMcontig_7	5,241,344	5,501,379	5,644,920	5,958,740	53.79	20.68	
5	ctg205	ctg206	OMcontig_7	5,894,810	6,136,813	6,359,020	6,677,410	76.39	31.56	
5	ctg206	ctg207	OMcontig_7	6,329,384	7,932,627	6,879,191	8,559,590	77.16	4.81	
5	ctg207	ctg209	OMcontig_7	11,181,371	11,704,297	11,697,110	12,327,950	107.91	20.64	
5	ctg209	ctg210	OMcontig_7	13,561,031	16,317,555	14,086,880	17,054,890	211.49	7.67	
5	ctg211	ctg212	OMcontig_7	20,250,693	21,605,532	21,095,380	22,499,960	49.74	3.67	
5	ctg212	ctg215	OMcontig_7	28,064,412	28,996,835	29,012,630	30,679,610	734.56	78.78	
5	ctg215	ctg216	OMcontig_7	30,464,929	30,550,161	32,174,410	32,322,040	62.40	73.21	

5	ctg216	ctg217	OMcontig_7	31,229,224	31,239,702	32,985,080	33,026,930	31.37	299.41	
5	ctg217	ctg218	OMcontig_7	37,657,014	37,872,290	39,444,800	39,720,820	60.74	28.22	
5	ctg218	ctg486	OMcontig_7	45,027,210	45,569,841	46,836,950	47,487,940	108.36	19.97	
5	ctg486	ctg219	OMcontig_7	45,694,265	45,720,961	47,608,230	47,645,990	11.06	41.44	
5	ctg219	ctg220	OMcontig_7	54,555,455	54,641,120	56,416,590	56,559,150	56.90	66.42	
5	ctg220	ctg221	OMcontig_7	61,347,898	62,768,236	63,253,840	64,724,900	50.72	3.57	
5	ctg221	ctg223	OMcontig_7	62,886,523	64,221,427	64,831,580	66,236,260	69.78	5.23	
5	ctg223	ctg225	OMcontig_22	76,887,098	77,689,306	28,229,360	27,446,830	-19.68	2.45	
5	ctg225	ctg227	OMcontig_22	86,686,634	89,284,128	18,631,260	16,056,360	-22.59	0.87	
5	ctg227	ctg228	OMcontig_22	94,388,047	94,638,979	10,935,320	10,637,200	47.19	18.81	
5	ctg228	ctg180	OMcontig_22	99,384,495	99,625,602	6,048,360	5,799,140	8.11	3.36	
5	ctg231	ctg230	OMcontig_14	121,329,069	121,366,997	2,349,500	2,413,650	26.22	69.14	
5	ctg230	ctg494	OMcontig_14	123,495,332	123,551,107	4,519,840	4,587,740	12.13	21.74	
5	ctg494	ctg233	OMcontig_14	126,515,455	126,724,214	7,520,790	7,856,670	127.12	60.89	
5	ctg233	ctg234	OMcontig_14	129,744,256	130,128,229	10,878,780	11,280,360	17.61	4.59	
5	ctg234	ctg237	OMcontig_14	160,246,834	161,015,609	40,986,060	41,818,850	64.02	8.33	
5	ctg237	ctg238	OMcontig_14	163,302,080	163,317,808	43,968,150	44,002,500	18.62	118.40	
5	ctg238	ctg241	OMcontig_25					see comment		ctg241 was inverted
5	ctg241	ctg242	OMcontig_25					see comment		ctg241 was inverted
5	ctg242	ctg244	OMcontig_25	182,077,315	184,266,280	15,787,600	17,925,270	-51.30	2.34	
5	ctg244	ctg245	OMcontig_25	185,986,602	186,693,022	19,724,700	20,549,470	118.35	16.75	
5	ctg245	ctg247	OMcontig_25	188,898,824	190,173,796	22,742,460	24,112,280	94.85	7.44	across two FPC gaps
5	ctg249	ctg250	OMcontig_33	195,765,167	196,454,338	21,512,820	20,793,700	29.95	4.35	
5	ctg250	ctg251	OMcontig_33	202,382,565	204,705,646	14,878,610	12,178,570	376.96	16.23	
5	ctg251	ctg253	OMcontig_33	206,721,054	208,100,861	10,131,520	9,024,470	-272.76	19.77	across two FPC gaps
5	ctg253	ctg254	OMcontig_33	212,424,733	212,432,549	4,824,490	4,705,530	111.14	1422.01	
6	ctg256	ctg257	OMcontig_48	677,441	2,680,267	892,250	2,867,480	-27.60	1.38	
6	ctg257	ctg259	OMcontig_48	4,857,743	5,499,125	5,065,210	6,423,320	716.73	111.75	
6	ctg259	ctg260	OMcontig_48	5,992,909	8,182,429	5,971,730	8,668,310	507.06	23.16	
6	ctg261	ctg262	OMcontig_30	13,272,950	13,728,613	3,631,070	4,120,240	33.51	7.35	
6	ctg262	ctg438	OMcontig_30/67					see comment		ctg438 is inconsistent with optical map, and should be close to ctg263
6	ctg438	ctg268	OMcontig_67/30					see comment		ctg438 is inconsistent with optical map, and should be close to ctg263
6	ctg268	ctg267	OMcontig_30	31,722,729	31,761,408	18,901,660	18,981,960	41.62	107.61	
6	ctg267	ctg266	OMcontig_60					see comment		ctg266 was inverted
6	ctg265	ctg269	OMcontig_36	63,738,835	63,854,627	6,515,020	5,015,440	1383.79	1195.06	
6	ctg269	ctg270	OMcontig_34	75,309,432	75,445,366	14,739,240	14,553,900	49.41	36.35	
6	ctg270	ctg271	OMcontig_34	83,525,829	84,672,272	6,249,540	5,489,520	-386.42	33.71	
6	ctg271	ctg442	OMcontig_53/36					see comment		ctg442 does not belong here
6	ctg442	ctg272	OMcontig_36/53					see comment		ctg442 does not belong here
6	ctg272	ctg273	OMcontig_53	94,772,875	96,138,568	3,705,720	5,224,400	152.99	11.20	
6	ctg274	ctg276	OMcontig_6	102,836,731	104,113,351	3,575,800	4,845,090	-7.33	0.57	
6	ctg280	ctg281	OMcontig_6	111,438,793	111,959,926	11,568,200	13,021,660	932.33	178.90	
6	ctg281	ctg282	OMcontig_6	129,103,243	129,337,413	29,803,990	30,090,450	52.29	22.33	
6	ctg282	ctg283	OMcontig_6	131,492,867	132,489,966	32,257,940	33,288,980	33.94	3.40	
6	ctg283	ctg284	OMcontig_6	140,067,667	140,116,466	41,044,880	41,140,640	46.96	96.23	
6	ctg284	ctg285	OMcontig_6	141,884,936	142,759,446	42,879,120	43,705,390	-48.24	5.52	
6	ctg285	ctg286	OMcontig_6	149,890,187	149,933,248	50,779,370	50,839,200	16.77	38.94	
6	ctg286	ctg287	OMcontig_6	150,594,349	150,815,584	51,506,240	51,722,950	-4.53	2.05	
6	ctg287	ctg289	OMcontig_6	164,004,029	164,209,396	65,259,160	65,454,420	-10.11	4.92	
6	ctg289	ctg291	OMcontig_6					see comment		ctg291 was inverted
7	ctg487	ctg296	OMcontig_20	6,146,406	6,186,478	6,457,260	6,507,280	9.95	24.83	
7	ctg296	ctg297	OMcontig_20	14,298,481	15,083,190	14,608,990	15,471,860	78.16	9.96	
7	ctg297	ctg298	OMcontig_20	18,878,117	19,689,853	19,358,820	20,446,840	276.28	34.04	
7	ctg298	ctg299	OMcontig_20/57					see comment		ctg299 is chimeric, and
7	ctg299	ctg300	OMcontig_20	32,685,673	33,853,584	31,042,100	32,270,490	60.48	5.18	
7	ctg300	ctg301	OMcontig_20	41,113,896	41,350,012	39,559,900	39,892,430	96.41	40.83	
7	ctg301	ctg456	OMcontig_43	52,438,573	53,945,650	8,436,320	11,582,660	1639.26	108.77	

7	ctg456	ctg303	OMcontig_43					see comment		ctg303 should be moved to between ctg301 and ctg456
7	ctg459	ctg470	OMcontig_10	60,316,911	60,442,049	55,448,078	55,218,978	103.96	83.08	
7	ctg470	ctg304	OMcontig_10	67,899,483	67,950,876	47,627,718	47,461,398	114.93	223.62	
7	ctg304	ctg306	OMcontig_10	74,217,723	74,592,159	41,146,318	40,885,798	-113.92	30.42	across two FPC gaps
7	ctg307	ctg430	OMcontig_10/40					see comment		ctg430 does not belong here
7	ctg430	ctg309	OMcontig_40/10					see comment		ctg430 does not belong here
7	ctg309	ctg311	OMcontig_10	103,055,213	105,508,652	18,063,678	15,594,248	15.99	0.65	
7	ctg311	ctg312	OMcontig_10	107,670,617	108,243,012	13,431,148	12,798,638	60.12	10.50	
7	ctg312	ctg313	OMcontig_10	111,277,679	113,068,923	9,767,688	7,555,098	421.35	23.52	
7	ctg313	ctg315	OMcontig_10	115,167,515	115,364,619	5,423,598	5,122,398	104.10	52.81	
7	ctg315	ctg316	OMcontig_10	117,908,711	118,861,024	2,616,738	1,553,418	111.01	11.66	
7	ctg317	ctg318	OMcontig_27	121,768,417	122,395,154	23,663,430	22,932,460	104.23	16.63	
7	ctg318	ctg320	OMcontig_27	132,333,743	133,147,706	15,520,590	12,088,690	2617.94	321.63	
7	ctg322	ctg324	OMcontig_31	156,530,582	156,554,984	9,188,580	9,232,950	19.97	81.83	
7	ctg324	ctg325	OMcontig_31	156,596,731	157,297,750	9,273,020	10,023,040	49.00	6.99	
7	ctg325	ctg501	OMcontig_31	169,591,070	170,682,997	22,162,270	23,316,330	62.13	5.69	
8	ctg326	ctg327	OMcontig_16	16,267,813	16,299,772	16,516,960	16,628,040	79.12	247.57	
8	ctg327	ctg328	OMcontig_16					see comment		ctg328 was inverted
8	ctg328	ctg329	OMcontig_16					see comment		ctg328 was inverted
8	ctg329	ctg330	OMcontig_16	25,809,425	26,618,156	26,177,990	28,224,690	1237.97	153.08	
8	ctg331	ctg334	OMcontig_16	37,520,274	39,179,273	38,027,290	39,699,690	13.40	0.81	
8	ctg332	ctg343	OMcontig_4	51,995,210	53,199,821	80,133,560	78,886,100	42.85	3.56	
8	ctg343	ctg333	OMcontig_4	56,216,677	56,353,853	75,890,720	75,783,150	-29.61	21.58	
8	ctg333	ctg457	OMcontig_4/16					see comment		ctg457 is inconsistent with optical map and it should be moved to between ctg334 and ctg332
8	ctg457	ctg335	OMcontig_16/4					see comment		ctg457 is inconsistent with optical map and it should be moved to between ctg334 and ctg332
8	ctg335	ctg336	OMcontig_4	62,753,948	62,998,937	71,680,210	70,815,730	619.49	252.86	
8	ctg336	ctg339	OMcontig_4	71,064,976	71,154,696	62,578,740	62,449,520	39.50	44.03	
8	ctg339	ctg428	OMcontig_4/No					see comment		ctg428 does not belong here
8	ctg428	ctg344	OMcontig_NO/4					see comment		ctg428 does not belong here
8	ctg344	ctg338	OMcontig_4	80,303,664	80,585,004	53,806,960	53,455,520	70.10	24.92	
8	ctg338	ctg337	OMcontig_4					see comment		ctg337 was inverted
8	ctg337	ctg340	OMcontig_4					see comment		ctg337 was inverted
8	ctg340	ctg345	OMcontig_4	95,445,064	97,295,447	38,906,760	36,761,180	295.20	15.95	
8	ctg347	ctg348	OMcontig_4	104,065,712	105,670,974	29,712,390	27,995,280	111.85	6.97	
8	ctg348	ctg349	OMcontig_4	107,564,799	108,548,041	26,054,170	24,866,380	204.55	20.80	
8	ctg349	ctg350	OMcontig_4	114,551,771	116,160,698	18,873,480	17,172,900	91.65	5.70	
8	ctg350	ctg352	OMcontig_4	119,881,340	120,269,717	13,459,180	12,965,950	104.85	27.00	
8	ctg352	ctg353	OMcontig_4	120,505,374	122,779,391	12,728,170	10,226,010	228.14	10.03	
8	ctg353	ctg354	OMcontig_4	124,113,549	124,240,886	8,862,800	8,369,160	366.30	287.66	
8	ctg354	ctg355	OMcontig_32	137,340,404	137,440,908	4,948,720	5,094,330	45.11	44.88	
8	ctg355	ctg356	OMcontig_32	138,104,869	139,763,786	5,763,660	7,534,660	112.08	6.76	
8	ctg356	ctg358	OMcontig_32	141,144,343	142,876,261	8,924,710	10,679,860	23.23	1.34	
8	ctg360	ctg362	OMcontig_35	159,320,170	159,381,669	15,491,120	15,347,760	81.86	133.11	
8	ctg362	ctg363	OMcontig_35	163,161,486	163,561,404	11,628,890	11,062,630	166.34	41.59	
8	ctg363	ctg364	OMcontig_35	167,418,574	168,784,759	7,115,000	5,807,280	-58.47	4.28	
8	ctg364	ctg365	OMcontig_35	170,464,570	170,517,291	4,165,030	4,081,170	31.14	59.06	
8	ctg365	ctg366	OMcontig_35	170,765,840	170,905,719	3,827,710	3,653,520	34.31	24.53	
9	ctg441	ctg368	OMcontig_51					see comment		ctg441 was inverted
9	ctg368	ctg369	OMcontig_51					see comment		ctg369 was inverted
9	ctg369	ctg370	OMcontig_51					see comment		ctg369 was inverted
9	ctg370	ctg373	OMcontig_18	12,595,568	15,965,905	44,378,870	40,784,230	224.30	6.66	across three FPC gaps

9	ctg381	ctg377	OMcontig_18	31,461,709	31,735,930	25,039,730	23,652,480	1113.03	405.89	
9	ctg378	ctg367	OMcontig_18	37,425,767	38,660,929	17,942,790	16,723,470	-15.84	1.28	
9	ctg367	ctg374	OMcontig_18	43,160,504	43,290,050	12,196,300	12,008,760	57.99	44.77	
9	ctg374	ctg375	OMcontig_18	44,909,784	47,593,781	10,338,420	7,643,040	11.38	0.42	
9	ctg375	ctg214	OMcontig_18					see comment		ctg214 was inverted
9	ctg106	ctg432	OMcontig_58	60,378,434	60,400,617	3,242,282	1,730,798	1489.30	6713.70	
9	ctg432	ctg448	OMcontig_55	64,952,653	65,016,516	5,004,000	4,174,380	765.76	1199.06	
9	ctg197	ctg491	OMcontig_17/58					see comment		ctg491 should be moved to between ctg106 and ctg432
9	ctg491	ctg431	OMcontig_58/17					see comment		ctg491 should be moved to between ctg106 and ctg432
9	ctg431	ctg376	OMcontig_17	80,132,348	80,172,031	10,293,140	10,327,270	-5.55	13.99	
9	ctg380	ctg473	OMcontig_17					see comment		ctg473 was inverted
9	ctg473	ctg382	OMcontig_17					see comment		ctg473 was inverted
9	ctg382	ctg383	OMcontig_17	110,996,910	111,155,183	41,399,910	41,610,250	52.07	32.90	
9	ctg383	ctg384	OMcontig_23	117,182,224	117,756,572	747,060	2,207,150	885.74	154.22	
9	ctg384	ctg385	OMcontig_23	118,664,751	119,034,882	3,114,100	3,557,540	73.31	19.81	
9	ctg385	ctg386	OMcontig_23	125,539,604	129,171,668	10,145,070	13,909,510	132.38	3.64	
9	ctg386	ctg387	OMcontig_23	129,403,145	129,600,608	14,134,020	14,391,070	59.59	30.18	
9	ctg387	ctg388	OMcontig_23	134,681,115	135,753,752	19,498,640	20,580,380	9.10	0.85	
9	ctg388	ctg389	OMcontig_23	136,159,869	136,216,994	20,981,379	21,044,560	6.06	10.60	
9	ctg389	ctg390	OMcontig_23	141,203,299	142,622,569	26,120,050	27,635,000	95.68	6.74	
9	ctg390	ctg391	OMcontig_23	143,910,969	144,049,258	28,895,760	29,098,230	64.18	46.41	
10	ctg392	ctg393	OMcontig_21	4,745,285	8,519,898	35,534,990	31,613,110	147.27	3.90	
10	ctg393	ctg394	OMcontig_21	10,857,911	12,116,659	29,290,290	28,531,770	-500.23	39.74	across two FPC gaps
10	ctg394	ctg395	OMcontig_21	14,420,385	15,013,830	26,236,030	25,484,460	158.13	26.65	
10	ctg395	ctg397	OMcontig_21	23,781,743	23,950,047	16,562,910	16,314,910	79.70	47.35	
10	ctg397	ctg398	OMcontig_21	26,468,740	28,853,832	13,850,790	11,420,800	44.90	1.88	
10	ctg398	ctg399	OMcontig_21/37					see comment		ctg399 does not belong here, and should be moved to between ctg84 and ctg400
10	ctg399	ctg84	OMcontig_37/21					see comment		ctg399 does not belong here, and should be moved to between ctg84 and ctg400
10	ctg400	ctg401	OMcontig_37	69,087,249	69,252,028	2,403,560	2,332,250	-93.47	56.72	
10	ctg401	ctg402	OMcontig_45	75,921,541	75,962,596	4,535,370	4,702,680	126.26	307.53	
10	ctg402	ctg403	OMcontig_45	76,757,144	79,654,502	5,479,910	8,380,380	3.11	0.11	
10	ctg403	ctg404	OMcontig_45	79,876,504	82,747,576	8,605,390	11,682,530	206.07	7.18	
10	ctg405	ctg406	OMcontig_8	84,656,175	85,867,808	65,163,620	63,884,590	67.40	5.56	
10	ctg406	ctg408	OMcontig_8	87,977,999	89,277,835	61,778,520	60,486,610	-7.93	0.61	
10	ctg408	ctg409	OMcontig_8	91,113,824	91,902,335	58,642,300	57,801,990	51.80	6.57	
10	ctg409	ctg410	OMcontig_8	97,775,874	98,003,015	51,824,320	51,581,980	15.20	6.69	
10	ctg410	ctg411	OMcontig_8	99,850,435	99,883,100	49,656,340	49,614,340	9.34	28.58	
10	ctg411	ctg721	OMcontig_8	112,928,266	113,014,218	36,512,760	36,392,270	34.54	40.18	
10	ctg721	ctg412	OMcontig_8	113,303,726	113,428,811	36,101,420	35,876,270	100.07	80.00	
10	ctg412	ctg413	OMcontig_8	120,399,486	123,951,414	28,832,650	25,298,550	-17.83	0.50	
10	ctg413	ctg414	OMcontig_8	130,841,449	132,724,232	18,406,510	16,502,860	20.87	1.11	
10	ctg419	ctg420	OMcontig_8	147,451,237	148,311,710	2,211,110	1,386,210	-35.57	4.13	

***Note:** negative gap size means that there are extra sequences included in the sequence pseudomolecules; Difference (%) = [gap size/(|AlignedSeqFlank1-AlignedSeqFlank2|/1000)]*100%, and we usually allow <= 10% optical sizing error.
